# Exploiting directional long range secondary forces for regulating electrostatics-dominated noncovalent interactions†
†Electronic supplementary information (ESI) available: Tables S1–S9, Fig. S1–S16, and *xyz* coordinates of optimized geometries for all of the structures considered in the study are provided here. See DOI: 10.1039/c6sc03642b



**DOI:** 10.1039/c6sc03642b

**Published:** 2016-10-11

**Authors:** Mrityunjay K. Tiwari, Kumar Vanka

**Affiliations:** a Physical and Material Chemistry Division , CSIR-National Chemical Laboratory , Dr. Homi Bhabha Road, Pashan , Pune-411008 , Maharashtra , India . Email: k.vanka@ncl.res.in

## Abstract

It has been well established that long range secondary electrostatic interactions (SEIs) have a significant effect on the stability of supramolecular complexes. However, general rules for exploiting SEIs in the rational design of diverse supramolecular complexes have been difficult to obtain. In this work, we outline a quantum chemical approach for understanding the strength of electrostatic interactions. This approach is seen to provide excellent correlation between the electrostatic force and the binding energy between two partners in hydrogen-bonded complexes, as well as that between two ions in ion-pair complexes. Furthermore, we illustrate how the understanding of the binding allows for the rational design of new complexes where the association constant between the two partners can be increased or decreased, as desired, by several orders of magnitude. Hence, the current work showcases a general, simple and powerful method of understanding and exploiting long range secondary electrostatic interactions.

## Abstract 2

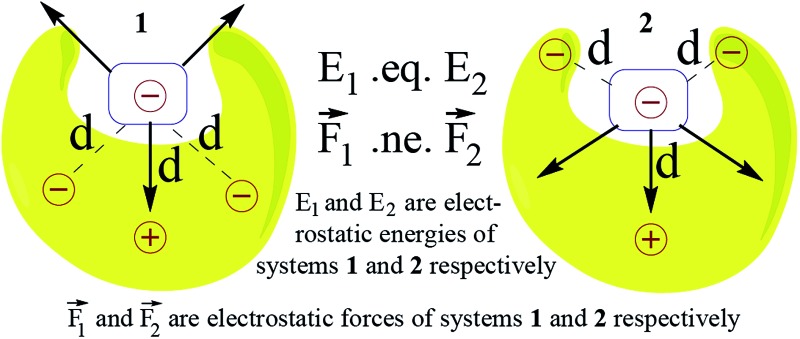
It has been well established that long range secondary electrostatic interactions (SEIs) have a significant effect on the stability of supramolecular complexes.

## Introduction

Noncovalent interactions are of great significance in several varied and important areas of chemistry and biology. Given their significance, there has been a conscious effort in recent times to exploit such interactions in order to achieve specifically designed goals, in areas as diverse as those of asymmetric catalysis,^1–4^ supramolecular chemistry,^1,5,6^ crystal engineering,^7–10^ polymer chemistry,^6,11,12^ peptido-mimetic chemistry,^4,13,14^ and molecular medicine.^15–18^ However, in order to fully unlock and exploit the potential of such noncovalent interactions, it is necessary to properly understand the factors that determine their strength. Some of these interactions are *de facto* dominated by electrostatics (*e.g.* XH–Y H-bonds),^19^ whereas some are dominated by dispersion and other factors (*e.g.* CH–π,^20^ π–π^21^). In this computational and theoretical study, our focus has been on the development of an understanding of systems where electrostatic noncovalent interactions are the principal influencing factor.

**Fig. 1:**
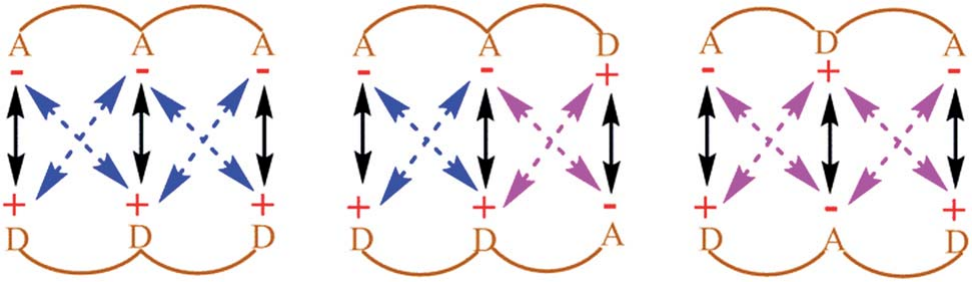
Electrostatic interactions between H-bond acceptors and donors in triply H-bonded complexes as proposed by Jorgensen *et al*.; A and D symbolize hydrogen bond acceptor and donor atoms respectively; black, blue and pink arrows represent attractive primary, attractive secondary and repulsive secondary interactions, respectively; the association constant decreases with an increasing number of repulsive secondary interactions.

In such systems, the fundamental question is that of understanding the extent of long-range electrostatic interactions in determining important properties of the system, such as the binding behavior of two partners into a single complex. A typical family of complexes that has generated great interest in this regard is that of the planar hydrogen-bonded complexes. Jorgensen *et al.* in their seminal theoretical study on triply hydrogen-bonded nitrogenous bases explained that it is inadequate to consider only primary electrostatic interactions in determining the association constant for a system with two partners held together by hydrogen bonding.^22^ They concluded that the electrostatic interactions between the immediate non-hydrogen bonded donors and acceptors, which they defined as secondary electrostatic interactions (SEI), also contribute significantly to the binding (Fig. 1). This has been supported by a plethora of experimental studies.^23–30^ However, the results of some subsequent studies^23,24,31^ have brought this hypothesis into question. Popelier *et al.* in their comprehensive QTAIM (Quantum Theory of Atoms-in-Molecules) study on 28 base pairs complexes have shown that the electrostatic energy of interaction between many remote atom pairs across a hydrogen bond is also influential to the binding, and hence the consideration of the electrostatic interactions of all the atoms of one partner with all the atoms of the other may be necessary in order to get the proper picture of the long range electrostatic influence on the binding.^32^

**Fig. 2:**
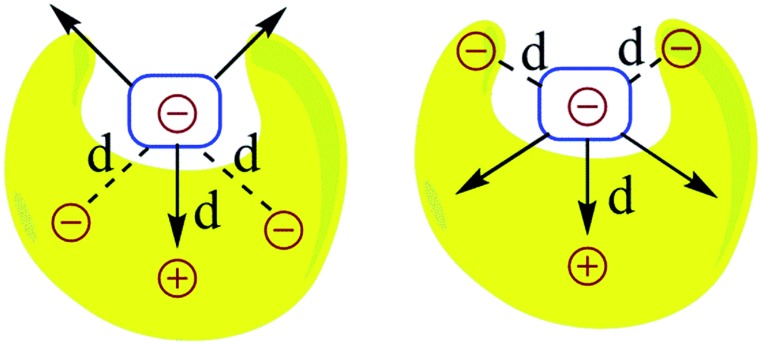
The representation of electrostatic forces between two molecular segments bonded by noncovalent interactions and having different charge distributions in three-dimensional space; *d* is the distance between the two charges. This model considers both primary and secondary interactions.

The question that has sparked the current investigation is this: since long range SEIs have been demonstrated to be significant, should not the electrostatic *force*, rather than the electrostatic energy of interaction, be the more important property that needs to be evaluated in order to get proper understanding and insight into such systems? For example, in structures such as those of proteins and DNA, hydrogen bonds are surrounded by atoms from every side in a three-dimensional framework. In such a situation, the electrostatic energy cannot define the strength of the interaction unambiguously. Electrostatic force, which has directionality, thus becomes a more significant factor. This point is illustrated by a simple model shown in Fig. 2. If all of the individual distances between the charges on the smaller subunit and the charges on the larger subunit in Fig. 2 are equal in the two complexes, the intermolecular electrostatic potential of both the systems would be the same. However, the electrostatic force of interaction will vary: the structure on the right will be more tightly held. One can also see from Fig. 2 that the line of approach of the two species is of significance. Therefore, it is more important to consider the electrostatic force of interaction rather than the electrostatic energy.

**Fig. 3:**
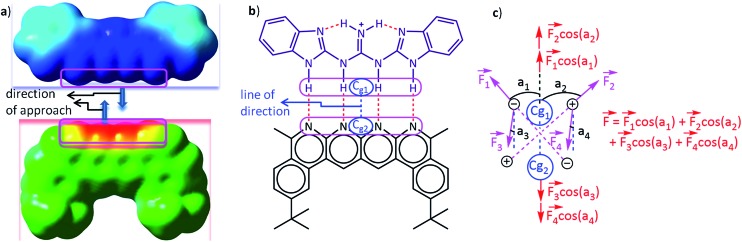
A schematic representation of (a) the direction of approach of two partners represented through their molecular surface electrostatic potentials, (b) the center of geometry of frontier atoms on two partners describing the line of direction and (c) the electrostatic force vector acting on one partner due to the charges on the other in a two dimensional planar model complex. The molecular surface electrostatic potential was computed in Hartrees on the 0.0004 au contour of the electron density using the GaussView software at the CPCM(CHCl_3_)/M062X/6-31G** level of theory. The blue colour on the potential surfaces indicates a positive electrostatic potential and the red colour indicates a negative electrostatic potential, whereas the intermediate colours indicate intermediate electrostatic potentials. The colour scale was kept uniform for both partners while constructing the potential surface. *C*_g1_ and *C*_g2_ in (b) and (c) are the geometric centers of the frontiers atoms of partners 1 (upper) and 2 (lower), respectively. The line joining *C*_g1_ and *C*_g2_ is defined to be the line of direction, which is the line along which the two partners will approach and interact with each other, as described by the nature of the electrostatic potential surfaces of the two partners in (a). *F*_1_, *F*_2_, *F*_3_ and *F*_4_ in (c) are the electrostatic forces acting on the atoms in one partner due to the charges on the atoms of the complementary partner. *a*_1_, *a*_2_, *a*_3_, and *a*_4_ are angles subtended by the forces *F*_1_, *F*_2_, *F*_3_ and *F*_4_, respectively, along the line of direction. Therefore, *F*_1_ cos(*a*_1_), *F*_2_ cos(*a*_2_), *F*_3_ cos(*a*_3_), and *F*_4_ cos(*a*_4_) are the components of the forces *F*_1_, *F*_2_, *F*_3_ and *F*_4_, respectively, along the line of direction. *F* is the net electrostatic force acting along the line of direction.

Several methods such as EDA (energy decomposition analysis), NCE (natural Coulomb electrostatics) and QTAIM have been developed and are being practised regularly for segregating and quantifying the electrostatic contribution in the interaction between two partners or fragments in a system. However, all of these methods rely on the computation of the electrostatic energy rather than the force. In the current work, we propose the determination and understanding of the electrostatic forces (EFs) as a viable alternative to account for the strength and nature of electrostatic interactions. The forces have been calculated by employing Coulomb's law, with the atoms being considered as point charges. The charges on the atoms have been determined from quantum chemical calculations, and the net electrostatic force of interaction between two partners has been determined in a particular direction assigned after careful analysis of the molecular structures (Fig. 3). This method has been discussed further in the Computational details and background theory section below. A flow chart of the FORTRAN code being implemented to calculate the forces is provided in Fig. S1 and S2.†

The current investigation focuses on two completely different families of complexes where the electrostatic interaction has been known to be the significant contributing factor: (i) the near-planar hydrogen bonded molecular complexes that have been studied extensively by Leigh and co-workers, as well as others, that are models for biological systems (Fig. S3 and S4†),^23,25–27^ and (ii) contact ion-pairs that are very significant in homogeneous olefin polymerization,^33–36^ which have been modeled by binding the cationic zirconocene, Cp_2_ZrMe^+^ with several different counterions (Fig. S5†). These two illustrative examples have been specifically chosen in order to highlight the efficacy of the current approach, because they represent two completely different challenges in their structure and function: while the goal of researchers working with the class of molecules in (i) has been to obtain as strong a binding as possible between the two partners, the objective in the field of homogeneous olefin polymerization (case (ii)) is to make the interaction between the cation and the counterion as weak as possible. The current approach allows each of these objectives to be realized, showing its general versatility and usefulness, and allowing for the rational design of new systems that are significantly better than the state-of-the art in the different fields. Also discussed is the scope of the work, thereby underlining its significance in several different areas of chemistry.

## Computational details and background theory

All the DFT calculations, unless mentioned specifically, were carried out using the Turbomole 6.4 suite of quantum-chemical programs.^37^ Geometry optimizations were performed using the PBE^38^ functional in the solvent phase using the Conductor-like Screening Model (COSMO)^39^ employing chloroform (epsilon = 4.81) as the solvent. The electronic configuration of the atoms was described by a triple-zeta basis set augmented by a polarization function (TURBOMOLE basis set TZVP). The resolution of identity (RI),^40^ and the multipole accelerated resolution of identity for *J* (MARI-J)^41^ approximations were employed for an accurate and efficient treatment of the electronic Coulomb term in the density functional calculations. The option “disp” provided in the Turbomole package (DFT-D2, a general, empirical dispersion correction proposed by Stefan Grimme for density functional calculations) was used for dispersion-corrected DFT calculations for all the calculations with Turbomole.^42^ Only the electronic energies were considered in calculating the binding and interaction energies. The free energies of binding were calculated to determine the association constant of specific systems wherever mentioned in the manuscript. The binding energy (*E*_b_) between two noncovalently bonded fragments was calculated using the following formula:

1 *E*_b_ = *E*_comp_ – (*E*_o,frag1_ + *E*_o,frag2_)

where *E*_comp_ is the energy of the noncovalently bonded complex, and *E*_o,frag1_ and *E*_o,frag2_ are the energies of two independently optimized fragments involved in the weak interactions. The interaction energy (*E*_i_) between two noncovalently bonded fragments was calculated as:

2 *E*_i_ = *E*_comp_ – (*E*_frag1_ + *E*_frag2_)

where *E*_comp_ is the energy of the noncovalently bonded complex, and *E*_frag1_ and *E*_frag2_ are the single-point energies of two fragments being separated from an optimized complex.

The energy decomposition analysis (EDA) was carried out using Turbomole 7.0 at the same level of theory and under the same conditions of solvent and dispersion that have been used for geometry optimizations using Turbomole version 6.4 (the EDA implementation is not available with Turbomole 6.4).

The Gaussian geometries were optimized at the M06-2X/6-31G** level of theory^43^ using the Gaussian 09 suite of quantum-chemical programs.^44^ The solvent effect was added through the Conductor-like Polarization Continuum Model (CPCM) using chloroform as a common solvent for all the geometries considered.^45^

The Mulliken^46^ and NBO^47^ charges have been used to calculate electrostatic forces on each fragment of every complex along a particular direction that described the intermolecular interactions most suitably (*i.e.* along the line of direction, see the Fig. 3a and b). To compute the magnitude of the net force of binding, *i.e.*, the binding force, the vector sum of the electrostatic forces experienced by each fragment along the aforementioned direction has been considered. A flow chart revealing the algorithm of the code that was employed for computing the forces is shown in Fig. S1 and S2.† Löwdin^48^ and CHelpG^49^ (Charges from Electrostatic Potentials using a Grid based method) charge analyses have also been employed for calculating the forces and for providing further validation of our method for a representative set of 16 planar hydrogen-bonded structures.

It is to be noted that interactions involving the hydrogen bond acceptors and donors that are directly involved in hydrogen bonding with each other are referred to as primary interactions. The electrostatic interactions between any pair of atoms between two partners that are not directly involved in hydrogen bonding have been defined as long range secondary electrostatic interactions. These interactions also include the Jogensen's type of secondary electrostatic interactions (SEIs).

A careful inspection of molecular structure is necessary in order to determine the direction to compute the net intermolecular electrostatic force of interaction. A thorough analysis reveals that the line joining the center of geometry of the frontier atoms in hydrogen bonded complexes describes the interaction forces most appropriately, because this is the line along which the two partners that will hydrogen bond would approach and bind with each other as revealed by the molecular surface electrostatic potential map in Fig. 3a. The frontier atoms of a hydrogen bonding partner are atoms that are nearest to the complementary partner and are directly involved in the hydrogen bonding (Fig. 3b). The center of geometry of the frontier atoms of a partner is the geometrical center of the frontier atoms. The *x*, *y* and *z* coordinates of the geometrical center of a particular partner were calculated by taking the average of the corresponding coordinates of all the frontier atoms. It has been assumed here that the two partners will approach each other along the line connecting the center of geometries of the frontier atoms. This hypothesis has been tested by a thorough analysis where we have calculated the binding forces along lines making angles of 0°, 30°, 60°, 90°, and so on up to 360° for a sample set of 16 representative complexes of the planar hydrogen bond family using Mulliken charges (Table S1†). The net forces of binding were also calculated along these lines. The magnitude of the total binding force was found to be most favourable (negative) along the originally chosen reference line of direction (*i.e.*, at 0° and 360°) for 10 out of the total 16 structures. A further analysis using NBO charges gives 15 structures having the most favourable binding along this line, and analysis using Löwdin charges gives 13 structures with maximum binding along this line of reference (please see Table S1† for details). Therefore, the line joining the center of geometries of the two hydrogen bonding partners was found to be the most appropriate, common line for the line of direction calculations for the family of planar hydrogen bonded complexes (Fig. 3b). However, in the case of the olefin polymerization catalysts, the line joining the central (metal) atom of the cation and the central atom of the anion was found to be the most appropriate line of direction for portraying the intermolecular electrostatic force of interaction, as the magnitude of the electrostatic force of binding was found to be greater (with Mulliken and NBO charges) along this line in comparison to the line parallel to the Zr–F bond. All the forces on one partner due to the presence of the other were calculated along this “line of direction” but in the direction of mutual approach as illustrated in Fig. 3c below by a simple two dimensional (2D) model complex. It should be noted here that Fig. 3c is a 2D model chosen for the purpose of simplicity and clarity. The complexes considered in this study are three dimensional (3D). Suitable measures have been taken in order to calculate the component of the forces along the line of direction by employing vector algebra.

The idea behind choosing a particular line as a line of direction has been derived from the following logic: when the electrostatic interaction is the dominant factor in the binding, the two binding partners, in general, would approach each other along a direction that is most favorable electrostatically, *i.e*., along the direction that maximizes the favourable electrostatic force of interaction. This implies that the magnitude of the electrostatic force of interaction must be the highest (with negative sign) along this direction, which we define here as the “line of direction”. This point can be understood by looking at the potential energy surface of the individual partners (please see Fig. 3a). Having hypothesized that there exists a direction (or a line) along which the favourable electrostatic force of interaction between two partners in a hydrogen bonded complex is the greatest, we have tried to deduce this direction by looking at the molecular geometries of the individual partners in the complex, and by employing the force analysis. With a simple approximation that all the hydrogen bonds in the complex are similar in strength, we can reach the conclusion that the line joining the center of geometries of the individual partners would be the best line to generalize as a line of direction, as also suggested by our force analysis results.

**Fig. 4:**
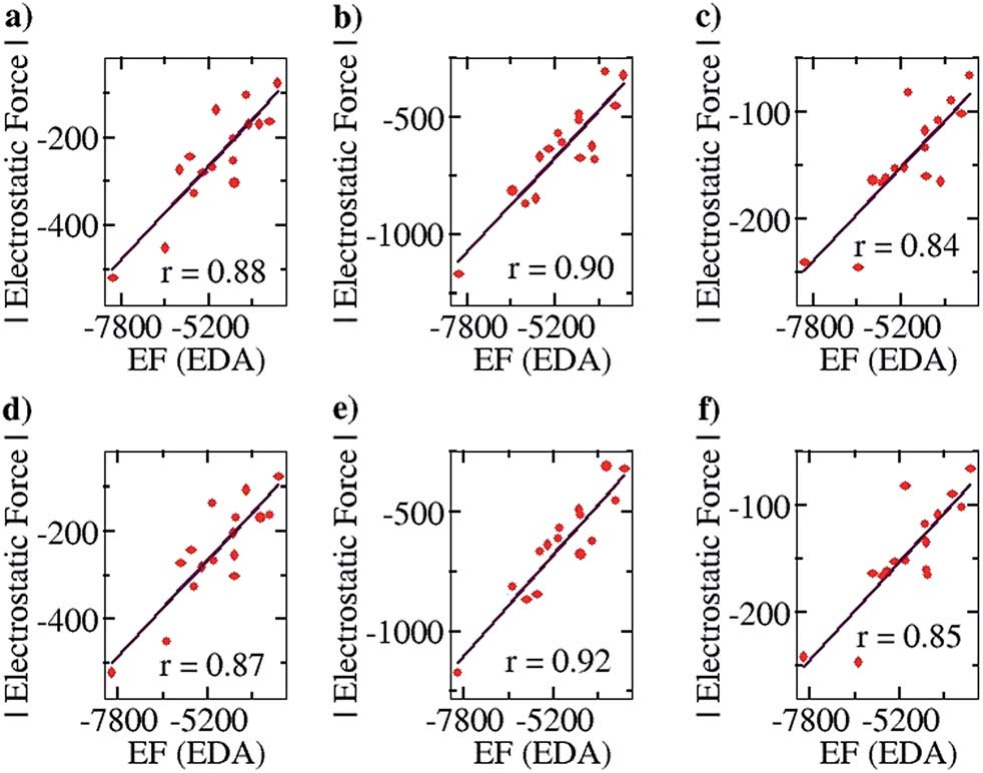
Pearson correlation graphs for planar hydrogen bonded molecules. EF (EDA) represents the EF (electrostatic force) obtained by calculating the negative of the gradient of the electrostatic energies of interaction between two points, calculated by the energy decomposition analysis (EDA) method. One of the two points considered for each complex is the optimized geometry, and the other point is the geometry obtained after translating one partner of each complex by 0.1 Å away from the corresponding partner along the line of direction. (a), (b) and (c) represent EF *vs.* EF (EDA) plots where the EF was calculated using Mulliken, NBO and Löwdin population analyses, respectively, and the EF (EDA) was calculated by constrained geometry optimization (keeping the frontier atoms fixed) for the complexes obtained after translation. (d), (e) and (f) are EF *vs.* EF (EDA) plots obtained when the EF was calculated using Mulliken, NBO and Löwdin population analyses, respectively, and the EF (EDA) was calculated by single point energy calculation of the complexes obtained after translation.

After ensuring a particular line as the line of direction, we have corroborated our electrostatic force analysis method with the electrostatic forces obtained with the help of the EDA analysis method, which we have termed “EF (EDA)”. EF (EDA) was calculated as a finite difference force obtained by taking the spatial derivative of the electrostatic energies (EEs), *i.e.*, it was calculated by taking the negative gradient of electrostatic energies between the two points: –{(EE_2_ – EE_1_)/(0.1)}. One of the two points considered is the optimized geometry, with the corresponding EDA obtained energy: EE_1_, and the other is the geometry optimized after translating one partner by 0.1 Å away from the other partner along the line of direction, with the corresponding EDA obtained energy: EE_2_. It should be noted that these newly obtained geometries for each complex case were optimized *via* a constrained geometry optimization, where the frontier atoms of each complex were frozen in order to preserve the center of geometry and maintain the additional distance of 0.1 Å between the centers of geometry in the two partners. The EF (EDA) thus obtained was compared with the EF obtained by our charge analysis approach. Linear plots with correlation coefficients of 0.88, 0.90 and 0.84 were obtained for the Mulliken (Fig. 4a), NBO (Fig. 4b) and Löwdin (Fig. 4c) charge analyses methods. Furthermore, since a change of 0.1 Å in the distance between the two centers of geometries should not lead to any appreciable change in the geometries, it is expected that single point calculations with the translated geometries, followed by the EDA analysis, will also provide the same results. This was indeed found to be the case: plots of EF *vs.* the EF (EDA) obtained by our approach showed a similar correlation of 0.87, 0.92 and 0.85 for the Mulliken (Fig. 4d), NBO (Fig. 4e) and Löwdin (Fig. 4f) charge analyses methods. These results suggest that the EFs obtained by employing our point charge analysis correlates excellently and linearly with the EFs obtained from the EDA analysis, and thereby validates our approach. Hence, in the subsequent studies with planar hydrogen bonded and contact ion-pair complexes that are discussed below, we have calculated and made use of the EFs that were obtained by our outlined charge analysis approach.

Credit for the pioneering contributions to the electrostatic force analysis should also be given to Berlin, who partitioned diatomic molecules (for example, a covalently linked H_2_ molecule) into binding and nonbinding regions on the basis of a binding force function *f*(*r*) obtained by the actual computed electron density under the Born–Oppenheimer approximation.^50^ Many attempts were made to extend Berlin's approach to analyze covalent bonds in polyatomic molecules by Bader *et al.*,^51^ Johnsen,^52^ Koga *et al.*,^53^ and other authors.^54^ However, this approach had also been criticized by Epstein,^55^ Koga *et al.* ^53^ and Silberbach^56^ for different reasons. Overall, the concepts of a binding and a nonbinding region become unrealistic when applied to real-life chemistry, specifically in polyatomic molecules.

The noted alternative method to obtain directionality in noncovalent interactions is provided by the Buckingham–Fowler model, according to which directionality in noncovalent bonds can be achieved as a function of relative orientation of interacting partners by putting them in van der Waals contact with each other and then by allowing one of them to roll over the other in search of the minimum electrostatic energy (till the global minimum is achieved).^57^

A recent review by Clark, Politzer, Murray and others states that even the polarization/induction and covalent (or donor–acceptor charge transfer) factors in the noncovalent bonds are essentially electrostatic in nature.^19^ According to the Feynman interpretation, even the dispersion interaction is electrostatic in nature.^58^ The Born–Oppenheimer approximation reveals molecules to be a collection of point charge nuclei and a cloud of indistinguishable electrons described by electron density. To distinguish a molecule in the form of atoms and bonds in a point charge analysis, the electron density is divided on the criteria of basis sets, *i.e.*, the atomic orbitals. When a noncovalent bonding partner is kept in the electric field of another partner, the induction causes a change in the local electron density of the first and *vice versa* as well. This is reflected in the point charge calculation as a modification in the charges of the atoms when the charges are calculated for partners in the complex in comparison to the charges when calculated with the two partners infinitely separated. The point charges that are calculated for the atoms in a complex will, therefore, also include the polarization and donor–acceptor charge transfer in an approximate way. This kind of analysis has been done by other groups as well to account for the effect of polarization and donor–acceptor contributions.^59^ Recently, noncovalent interactions between covalently bonded atoms of groups IV–VII and a negative site (*e.g.* a Lewis base) have been discovered, and are commonly referred to as σ-hole bonding, because of the presence of a positive cap on the electrostatic potential surface on the opposite side of one of the covalent bonds of the atom, labeled as a σ-hole.^60–62^ One of the most common known cases of σ-hole bonding is halogen bonding. The σ-hole arises due to anisotropy of the atomic charge distribution, and can be visualized through an anisotropic electrostatic potential around the atom. This results in unusual behavior of atoms that have σ-holes. Such atoms can have regions of both positive and negative electrostatic potential on their surfaces, and they can thus interact attractively with both negative and positive sites respectively, in different directions.^60,62^ Assigning a single atomic charge to such atoms in molecular complexes will fail to describe σ-hole bonding. More recently, methods have been developed in molecular dynamics (MD) simulations to address such behavior in a more accurate way. Force fields have been developed where the positive region of halogen atoms are represented as an extra point of charge in a way so that the net formal charge and the electrostatic potential assigned to the atom remain the same.^63,64^ The results of this method have also been corroborated by high level quantum chemical calculations.^63^ Therefore, assigning one extra-point charge to every positive hole in a molecule/complex was found to give a good reasonable approximation for modulating other electrostatic properties. However, σ-hole bonding is not found to be universally manifested by all the elements of groups IV–VII in every chemical composition. In general, σ-hole bonding is not exhibited by fluorines and is insignificant in other elements of the same period, particularly when they are not covalently linked to more electronegative atoms.^63,65^ This can be justified by looking at the molecular electrostatic potential surface map of the corresponding compounds. Molecules that do not possess σ-holes may not require additional treatment (of assigning extra point charges for representing σ-holes) to accurately address the electrostatic properties. To further confirm whether the individual noncovalently bonded partners considered in this study possess σ-holes on their electrostatic potential surfaces, we have constructed the molecular surface electrostatic potential map of a set of representative noncovalent partners containing nitrogen and fluorine atoms at their interactive sites. The obtained electrostatic potential surfaces reveal that none of the moieties that have been considered in this study possess σ-holes on their surface. Please refer to Fig. S10† for details. It is, therefore, correct to conclude that the point charge calculations employed in the current work provide a good approach to estimating the net electrostatic interactions, especially when determined from charge calculations at a high level of theory. However, additional caution should be taken for molecules containing σ-holes.

## Results and discussion

In order to determine the EF existing between two partners, the approach that has been adopted has been to determine the coulombic force between each pair of atoms, with the atoms being chosen from the different fragments, and then summing up the forces. This provides the net EF of interaction between the two fragments. As mentioned in the Introduction, the point charge approximation has been employed for this purpose, and each atom has been considered to have a charge, determined by the NBO and/or the Mulliken charge analysis. Since force has direction, the Coulombic interaction has been considered along a certain common line: the “line of direction” for the given molecule (see Fig. 3). Therefore, only the component of the force along that given line has been considered. The algorithm for the code that incorporates this approach has been provided in the ESI (Fig. S1 and S2†).

### (i) The planar hydrogen bonded structures case

**Fig. 5:**
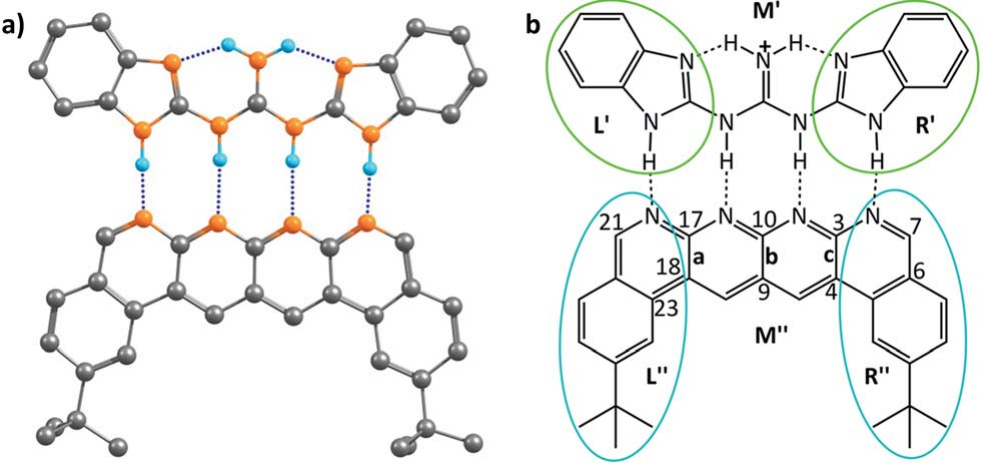
(a) The optimized geometry of the cationic complex reported by Leigh *et al*.; black, cyan and orange colours represent carbon, hydrogen and nitrogen atoms, respectively; dotted lines represent the hydrogen bonding interactions. Hydrogen atoms other than those involved in hydrogen bonding interactions have been deleted for clarity. (b) A schematic picture of the cationic complex showing the three regions into which each of partners was divided for the electrostatic force analysis.

This family of complexes involves two planar fragments interacting through X–H···Y hydrogen bonded interactions, with the number of such hydrogen bonds varying from two to four. The significance of such complexes lies in the fact that they can serve as model structures for investigating and understanding multipoint hydrogen bonding, which is highly relevant to biological systems as well as to multifunctional materials and supramolecular polymers.^23,25–27,66^ Hence, there have been a large number of reports in recent times that have discussed different planar structures belonging to this family. A typical optimized structure is shown in Fig. 5a, with four N–H···N hydrogen bonds connecting two fragments.

**Fig. 6:**
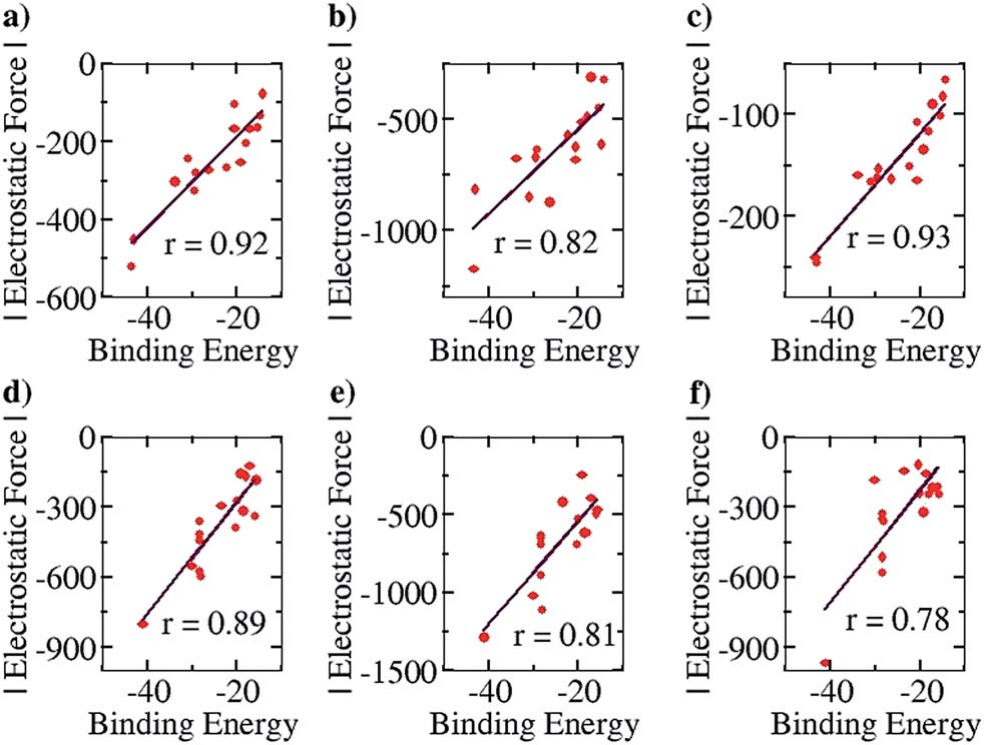
Pearson correlation graphs for planar hydrogen bonded molecules: (a) EF *vs. E*_b_ for Mulliken charges at the COSMO/PBE/TZVP Turbomole 6.4 geometries, (b) EF *vs. E*_b_ for NBO charges at the COSMO(CHCl_3_)/PBE/TZVP Turbomole 6.4 geometries, (c) EF *vs. E*_b_ for Löwdin charges at the COSMO/PBE/TZVP Turbomole 6.4 geometries, (d) EF *vs. E*_b_ for Mulliken charges at the CPCM/M06-2X/6-31G** Gaussian 09 geometries, (e) EF *vs. E*_b_ for NBO charges at the CPCM(CHCl_3_)/M06-2X/6-31G** Gaussian 09 geometries and (f) EF *vs. E*_b_ for ChelpG charges at the CPCM(CHCl_3_)/M06-2X/6-31G** Gaussian 09 geometries.

This family of complexes is an ideal choice for testing our approach because it has been shown that SEIs are very important for determining the stability of these complexes,^23–30^ which has led experimentalists to the design principle of having all the hydrogen bond acceptor groups in one fragment and all the hydrogen bond donor groups in the other.^22^ We have therefore taken a sample set of sixteen different representative complexes from this family, optimized the structures (with Turbomole 6.4 and the COSMO(CHCl_3_)/PBE/TZVP level of theory, Fig. S3†) and obtained the total force of electrostatic interaction between the two fragments for each case. The line of direction along which the force had been considered is the “center of geometry” of the frontier atoms of the two fragments, because this is the line along which the two partners that will hydrogen bond would approach and bind with each other. Furthermore, we have obtained the energy of binding of the two fragments for each case. A graph with the EF (with the charges obtained from a Mulliken charge analysis) on the *y* axis and the corresponding binding energies on the *x* axis is shown in Fig. 6a below. Gratifyingly, we find a near linear correlation (*r* = 0.92) between the two quantities for the sixteen cases considered. In order to show that the result is not an artifact of the method of charge analysis, we have repeated the EF calculations by taking the charges from the NBO analysis and have obtained a graph of comparable linear correlation (*r* = 0.82), as shown in Fig. 6b below. It is believed that systems dominated by polarization are better represented by NBO charges in comparison to the Mulliken approach.^47^ Since the long range electrostatic interactions between atoms in the two hydrogen bonding partners are unlikely to be influenced by polarization (as the atoms are from the first and second periods of the Periodic Table), this helps explain why Mulliken charges give better results than NBO, especially since the calculations have been done with good basis sets. In addition to this, in order to show that similar results would be obtained by other population calculation approaches, we have done the analysis with another (conceptually different) charge analysis method, Löwdin, and obtained a linear correlation (*r* = 0.93, see Fig. 6c) for this as well. Furthermore, in order to show that the results are not dependent on the choice of basis set and functional, we have repeated the optimization calculations at the CPCM(CHCl_3_)/M06-2X/6-31G** level of theory with Gaussian 09, with a slightly more diverse group of sixteen structures (Fig. S4†), and have found a similar correlation between the total EF of interaction and binding energies for both the Mulliken (*r* = 0.89, Fig. 6d) and NBO charge (*r* = 0.81, Fig. 6e) analysis cases. A further analysis with the charges obtained from the CHelpG method for the same set of 16 molecules also gave a satisfactory correlation constant of *r* = 0.78 (Fig. 6f). It is to be noted that charges assigned to the atoms by the CHelpG method account for the electrostatic potential around each atom, and therefore employing such charges for the force calculations in our method can be considered a means of accounting for the electron density around each atom.^49^

**Fig. 7:**
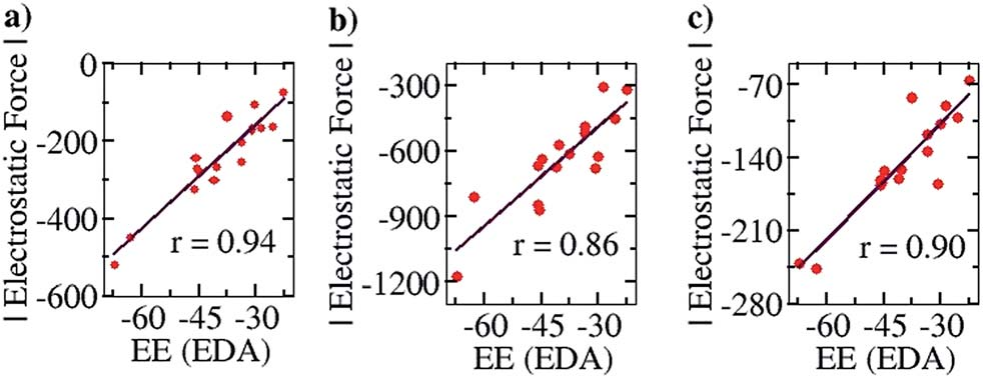
Pearson correlation graphs for planar hydrogen bonded molecules: (a) EF *vs.* EE (EDA) for Mulliken charges at the COSMO/PBE/TZVP Turbomole 6.4 geometries, (b) EF *vs.* EE (EDA) for NBO charges at the COSMO(CHCl_3_)/PBE/TZVP Turbomole 6.4 level of theory and (c) EF *vs.* EE (EDA) for Löwdin charges at the COSMO(CHCl_3_)/PBE/TZVP Turbomole 6.4 level of theory. EE (EDA) represents the electrostatic component of the binding energy obtained from the Energy Decomposition Analysis (EDA) method implemented in Turbomole 7.0.

To further corroborate our results with the electrostatic component of the binding energy, we did an energy decomposition analysis (EDA) using Turbomole 7.0. A graph with the EF on the *y* axis and the corresponding electrostatic component of binding energies obtained from the EDA method, “EE (EDA)”, on the *x* axis, is shown in Fig. 7. We found an improved correlation between the two quantities for the sixteen cases considered in the Mulliken (*r* = 0.94, Fig. 7a) and NBO (*r* = 0.86, Fig. 7b) cases, compared to the EF *vs. E*_b_ plots (Fig. 6a and b) shown earlier. This further shows that our approach correctly captures the electrostatic interaction between the two partners. Likewise, a good correlation was also obtained for the Löwdin population analysis (*r* = 0.90, Fig. 7c) case.

In order to further understand why the electrostatic force correlates so well with the binding energy, additional calculations have been done with a simple model system that is shown in Fig. S6 in the ESI.† The figure shows two hydrogen bonding partners that each possess two charged centres that are equidistant from their oppositely charged counterparts (see Fig. S6†). When the charges are all negative or all positive, the net force is repulsive (as shown in the figure), but when the charges in one partner are positive and the charges in the other partner are negative, then the net force would be attractive (not shown in the figure). The net electrostatic force (along with the direction obtained) was calculated for each charge combination. Furthermore, for each combination, the net electrostatic energy was also calculated. This was done for different sets of values for the charges. The set of thirty electrostatic force values thus obtained was correlated with the thirty corresponding electrostatic energy values, and an exact correlation was obtained (see Fig. S6c†). When an angle 180° different from the resultant line of force was taken, an exact negative correlation was obtained (see Fig. S6d†). This indicates that at a given distance and for a particular arrangement of atoms, the electrostatic force of binding (when considered along the direction of approach and along the line of direction) correlates perfectly with the electrostatic energy, *i.e.*, the greater the magnitude of the force, the greater the electrostatic binding energy. In other words, the electrostatic force and the electrostatic energy change by the same proportion when charges on atoms are changed in a complex while the partners remain at a fixed separation. This explains why good linear correlation has been obtained between the EF and binding energy within the chosen family of complexes in this study: the major influencing factor within each family (when we move from one complex to the other in the family) is the charges on the atoms, and not the distances between the atoms, as the relative atomic arrangement of the atoms within each family of complexes is nearly the same. Furthermore, the reason why the total electrostatic force of interaction is seen to correlate so well with the binding energy is because the greater electrostatic interaction between the two partners allows them to overcome the Pauli repulsion force to a greater extent, thereby allowing them to bind more strongly in their equilibrium structures. Therefore, determining the electrostatic force of interaction along the line of direction, which is the line along which the two partners approach and bind, and the line along which the interaction between the partners is the greatest, is shown to be the correct approach for understanding the binding between the two partners.

Therefore, the results showcase the validity of our approach, and also illustrate the importance of taking the SEIs due to *all* the atoms in each fragment into consideration, rather than the SEIs for only the frontier atoms, as has been the traditional view.^22–30^ Indeed, a plot of the EF obtained by considering only the frontier atoms *versus* the binding energy shows a poorer correlation (*r* = 0.75, for the Mulliken charge analysis case), as opposed to *r* = 0.92 when all atoms are taken into account. It is to be noted here that the interactions of the frontier atoms in quadruply hydrogen-bonded complexes also include the secondary interactions between diagonal atoms, which were not taken into account in Jorgesen's hypothesis^22^ (Fig. 1). Furthermore, when the EF was calculated by calculating the forces (for all the atoms) along a line of direction perpendicular to the line employed in the calculations (the line connecting the centers of geometries of the frontier atoms, as stated earlier), we observed a *negative* correlation of the EF with the binding energy, with *r* = 0.85 (Mulliken charge analysis case), with the net EFs for each of the sixteen structures now found to be positive (see Fig. S7†). This further shows the significance of taking the direction of the electrostatic interaction into account. It is to be noted that Berlin in the 1950s and Bader and coworkers in the 1960s had attempted to understand covalent bonding in compounds by dividing a molecule into “binding” and “non-binding” regions based on electron density calculations (see the “Computational Details and Background Theory” section above for more details and references). In effect, our current work shows that the line of direction is analogous to the “binding” and “non-binding” regions that had been discussed by Berlin and Bader earlier, with a further important distinction that the line of direction is now applied to non-covalently bonded supramolecular complexes.

**Fig. 8:**
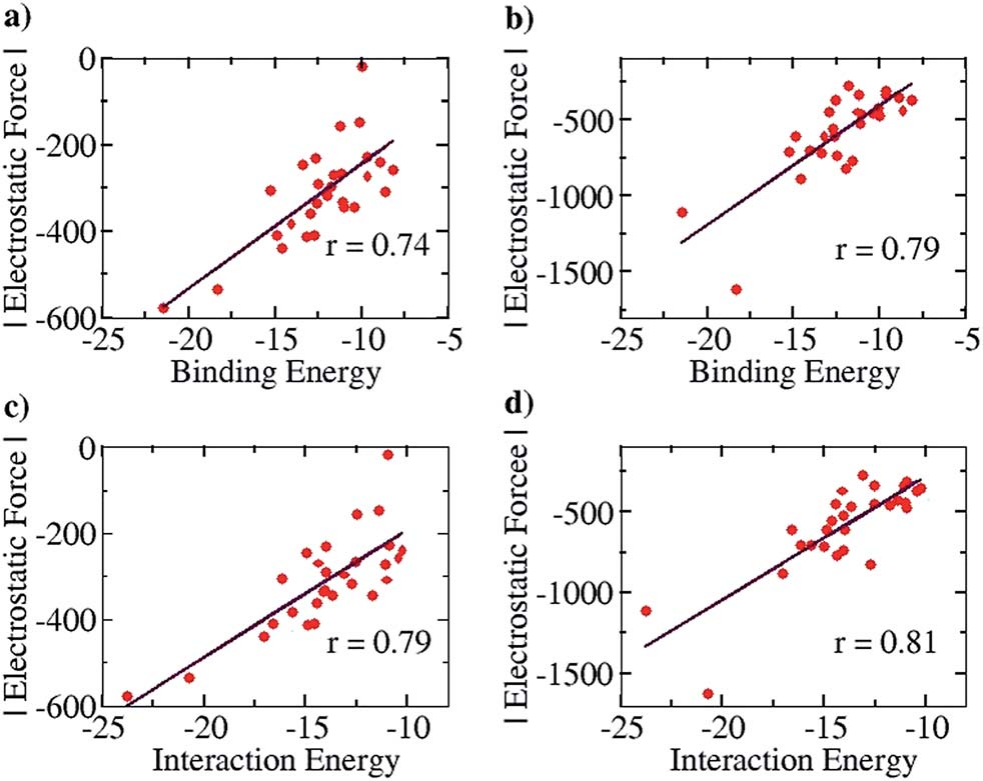
Pearson correlation graphs for nitrogen base pairs: (a) EF *vs. E*_b_ for Mulliken charges, (b) EF *vs. E*_I_ for NBO charges, (c) EF *vs.* IE for Mulliken charges and (d) EF *vs. E*_i_ for NBO charges.

We further note here that Popelier *et al.* had also suggested that including only the frontier atom SEIs leads to erroneous results for different hydrogen bonded cases.^32^ They pointed out that taking frontier atom SEIs as an indicator and a design principle was flawed, because the efficacy of frontier atom SEIs was only limited to specific cases. We have employed our approach (structures optimized at the COSMO(CHCl_3_)/PBE/TZVP level of theory, Fig. S8†) to determine the correlation between the total EF (including all the atoms, between the two base pair fragments) and the binding energy (between the two base pair fragments), for the 28 base pair cases (including the uracil-diaminopyridine: U-DAP interaction, see Fig. S8†) that Popelier *et al.* had studied. The line of direction was taken, as before, to be the center of geometry of the frontier hydrogen bonding atoms. The results indicate significant correlation: *r* = 0.74 with the Mulliken charge analysis (Fig. 8a), and *r* = 0.79 with the NBO charge analysis (Fig. 8b). It is to be noted that the EF was calculated between the hydrogen bonding partners obtained after geometry optimization of the complexes, and the binding energies (*E*_b_) of the complexes were obtained with respect to the infinitely separated partners. In order to look into the effect of geometrical variance in the presence and absence of the hydrogen-bonded partner, we have also done Pearson correlation analysis for the EF *vs.* the interaction energy (*E*_i_) plot (please see the Computational Details section for the description of how the *E*_b_ and the *E*_i_ have been calculated). A marginally improved correlation coefficient was obtained for both Mulliken (*r* = 0.79, Fig. 8c) and NBO (*r* = 0.81, Fig. 8d) charge analyses, indicating only a minor change in the geometry of the molecular fragments while optimized independently, mainly due to their rigid aromatic framework. Hence, the calculations of the EF with our approach for the 28 base pair cases also shows good correlation with the binding energy, indicating that our method works for different families of hydrogen-bonded complexes.

However, the advantage of employing the Jorgensen approach – that of looking at only the frontier atoms to understand the strength of interaction between the two interacting partners – was that it provided a simple means of designing systems that would bind more strongly and effectively. While the current work shows the limitations of that approach, does it then also provide a superior means of design that would lead to more strongly binding systems? It does indeed do so: since the code determines the specific force of interaction between each atom in a given partner and all the atoms of the other partner, one can write down the value for each such interaction in an output file, and then look at the output to determine which specific interactions are the strongest. As an example, we consider the structure shown in Fig. 5, which has been shown to have the strongest binding,^23^ among all the planar hydrogen bonded structures that have been studied to date. Indeed, we obtained the binding energy for the two partners in this case to be –43.4 kcal mol^–1^, which is the highest among all the cases considered in this study. We aimed to improve the binding energy of the system by changing the non-frontier atoms (which were not considered in the Jorgensens theory) in order to ascertain the impact of long range secondary interactions. We first calculated the net interaction forces experienced by every non-frontier atom on the acceptor partner due to all the atoms on the donor partner and *vice versa*. A perusal of the output files shows that both the strongest attractive and repulsive interactions are experienced by atoms from the middle region **M′′** (see Fig. 5b) of the acceptor partner. The atoms 3 and 17 face the most attractive interactions followed by atoms 10, 7 and 21, whereas the most repulsive interactions experienced by atoms other than H are 9 and 4 followed by 23, 18 and 6 (see Fig. 5b). An examination of individual atom–atom interactions between the rear atoms of one partner and each atom of the other partner also suggest the same trend in the electrostatic forces, as atoms 3 and 17 face the most attractive and 9 and 4 face the most repulsive interactions, all belonging to the middle region of the acceptor segments. Interestingly, as revealed from the output files, the magnitude of the attractive interactions is greater than the magnitude of the repulsive interactions. Based on this result, in a further analysis, we divided each donor and acceptor molecule into three regions (left, middle and right), as shown in Fig. 5b, and calculated the net force experienced by each region due to all the atoms on the complementary partner. The output further suggests that the middle region of the acceptor contributes the most to the net attractive interaction between the two partners.

Based on this analysis, in order to enhance the net attractive EF between the two partners, we first replaced bond **a** with the isoelectronic B–N bond to obtain a new structure. Another structure was obtained by doing the same with bond **b**. Then, a new structure was obtained by replacing bonds **a** and **c** together. Finally, all of the bonds **a**, **b** and **c** were substituted at once with the isoelectronic B–N bonds, as shown in Fig. S9.† The newly designed AAAA–DDDD cationic systems were obtained with improved binding energies of –49.6, –49.2, –55.7 and –60.2 kcal mol^–1^, respectively (see Table S6†). This translates to association constants that are orders of magnitude greater than those obtained for the best-known case to date, for which the binding energy is –43.4 kcal mol^–1^. An electrostatic force analysis reveals that the increase in binding affinity occurs mainly due to more favorable interactions between the frontier atoms of the newly designed complex, caused by the altered electronics of the frontier atoms due to modification at the remote sites. An electrostatic potential surface map also suggests accumulation of a more negative electrostatic potential near the frontier atoms in the newly designed acceptor partner with respect to the originally synthesized molecule (see Fig. S10† for details). It is to be noted here that the frontier atom interactions in quadruply hydrogen-bonded complexes also include the secondary interactions between diagonal atoms, which have been overlooked in Jorgesen's analysis^22^ (Fig. 1). However, the contributions from the remote atoms cannot be neglected.

This modification has been extended to other similar molecules that have been considered in this study (Fig. S13†). In all cases, the binding energy of the newly obtained complexes was found to be increased (Table S7†). Recent reports suggest that replacements of C–C bonds of aromatic systems with isoelectronic B–N moieties is possible with specified strategies;^67^ hence the synthesis of such proposed compounds would be quite feasible.

Furthermore, it has been shown in the literature that electrostatic interactions are weak in CH–π interactions, where the attractive dispersion interaction is the more dominant.^20^ Since our current work shows the significance of interactions in atoms far away from the frontier hydrogen bonding region, another simple recipe for improving the bonding would be to add phenyl rings in one of the two partners, as shown in Fig. S11.† The primary electrostatic impact would be marginal, and all the EFs between the phenyl ring atoms and the atoms of the partner would have a very small component along the line of direction. Therefore, the electrostatic effect of adding the phenyl rings would be small, while the system would benefit from attractive dispersion interactions, thereby improving the binding strength. This is indeed seen to be true: as shown in Fig. S11,† structures S11(B-1) and S11(B-2), which are modifications of the structure in Fig. 5b with phenyl rings at the end of the frontier line, have binding energies of –50.2 and –50.8 kcal mol^–1^ respectively, *i.e.*, about 7.0 kcal mol^–1^ stronger than the binding energy obtained for the Fig. 5b structure (Table S6†). To further examine the effect of non-directional attractive dispersion forces on the binding strength of complex 5a, we next substituted frontier non-hydrogen-bonded hydrogen atoms on acceptor and donor partners with sterically less demanding substituents –CH_3_, I, Br and –OCH_3_ as shown in Fig. S14.† Improved binding energies were obtained with respect to the parent AAAA–DDDD complex (Table S8†), which suggests that the binding is benefited by dispersion interactions in these designed complexes. However, dispersion can be closely counteracted by sterics, which works towards destabilizing the complex by weakening the existing hydrogen bonds. Hence, the unhelpful steric effect of adding new groups should also be kept in mind when designing such systems.

Now, taking a hypothetical “best case” design improvement of the Fig. 5b structure (Fig. S9A-4†), one can put three B–N pairs in the place of the C–C bonds, and put *ortho* and *para* methyl-substituted (so as to increase dispersion interactions) phenyl rings at each end, in order to get a new structure (see Fig. S12†). Such a structure would be expected to have the best binding energy. This is indeed seen to be the case: we find that the binding energy of this complex is as high as –69.6 kcal mol^–1^, *i.e.* 60.4% greater binding than the strongest-binding structure known to date! Such a complex would have a association constant that would be as much as 1.9 × 10^15^ times greater than the association constant of the “best” known complex of this family, which shows the great power of understanding and exploiting SEIs by the current approach.

We note that there might be individual cases in the considered families where the electrostatic force would be seen to not correlate with the binding energy. A reason for such a deviation is the selection of the common “line of direction” (line joining the centers of geometries of the frontier atoms in each complex) for all the complexes of a family. The best line of direction may vary slightly for specific complexes in the family, thus leading to the possibility of having two complexes with similar binding energies but slightly different electrostatic forces of binding. However, for a given family of complexes of sufficient sample size, the electrostatic force of binding will fairly represent the binding strength of the electrostatics-dominated noncovalent bonds, and thus it can be exploited for the design of new, superior systems by tuning the strength of the noncovalents bonds.

### (ii) The contact ion-pair case

**Fig. 9:**
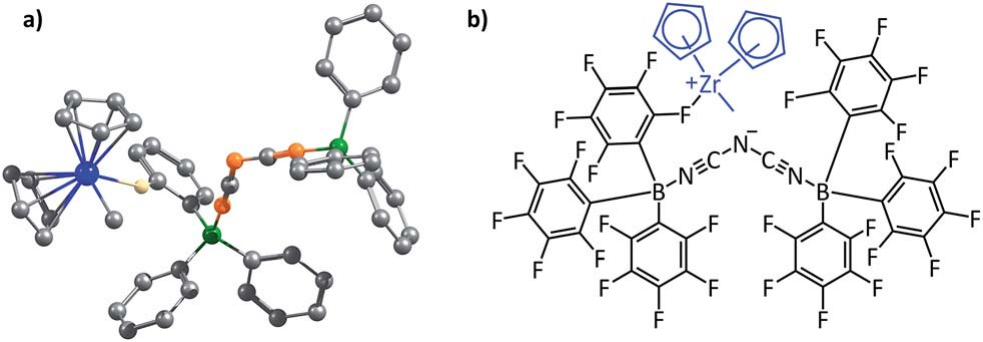
(a) The optimized geometry of a non-coordinating dinuclear anion and the cationic zirconocene complex; black, orange, green, blue, and white colours represent carbon, nitrogen, boron, zirconium and fluorine atoms, respectively. All the hydrogens of the zirconocene and the fluorides of the anion other than the one involved in coordination with the cation have been deleted for clarity. (b) A schematic picture of the non-coordinating dinuclear anion and the cationic zirconocene complex.

The second family of structures that we have considered is that of the contact ion-pair catalysts employed in homogeneous olefin polymerization. This is an important area of research, beginning with the pioneering work done by Kaminsky *et al.* in the 1980s.^68^ The active catalyst in these systems is the cationic species. One of the major foci of investigation in these systems has been to make the counterion as weakly coordinating as possible (several hundreds of papers have been produced in this pursuit). This is because the reduced interaction with the cation would allow the counterion to be displaced easily when the olefin substrate approaches the cationic metal center. A large variety of counterions have been employed over the years, with one of the most successful being the “BARF anion”: B(C_6_F_5_)_4_^–^.^33^ More recent counterions that achieve weaker interaction with the cation even in comparison to B(C_6_F_5_)_4_^–^ are the dinuclear counterions that have been proposed by Bochmann and coworkers.^36^ The optimized structures of the counterions are shown in Fig. S15,† and they will henceforth be referred to as the “Bochmann anions”. We decided to investigate the contact ion-pair catalyst systems by focusing on how one could rationally design better counterions, *i.e.*, ones that would interact more weakly with the counterions even in comparison to the Bochmann anions. The zirconocene cation Cp_2_ZrMe^+^, was employed as the model cation in these studies, and the total electrostatic interaction of this cation has been considered for different contact ion-pair cases, with a range of different counterions considered. Fig. 9 below shows the optimized ion-pair structure of the zirconocene cation with best of the three Bochmann anions.

Since the two binding partners of the ion-pair complex are charged, the dominant noncovalent interactions would be likely to be electrostatic in nature, which makes this family of complexes appropriate for investigation with our approach. It is also to be noted that the sum total of the long range secondary EF in the complexes studied was found to be approximately half that of the primary EF, which is the same ratio that had been found for the planar hydrogen bonded structures studied in (i).

**Fig. 10:**
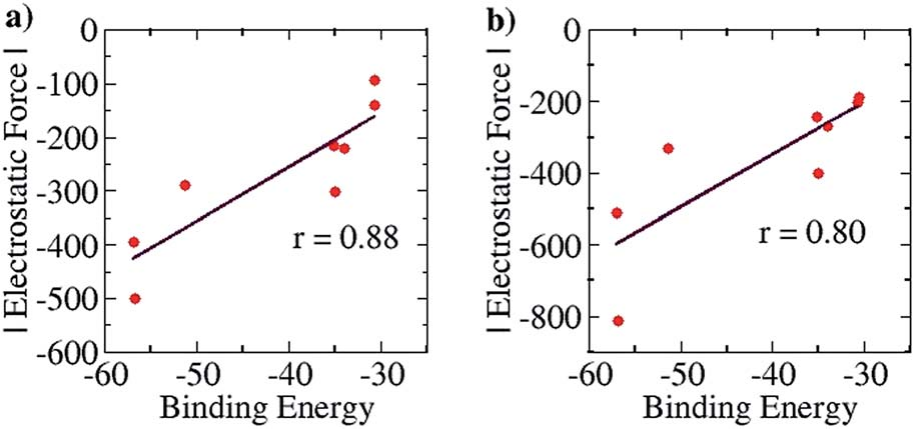
Pearson correlation graphs for the olefin polymerization catalyst: (a) EF *vs. E*_b_ for Mulliken charges and (b) EF *vs. E*_b_ for NBO charges.

For the purpose of analyzing the interaction by our approach, the line of direction chosen was the line connecting the zirconium atom in the cation and the central atom in the anion. The results of our calculations (with Turbomole 6.4, at the COSMO(CHCl_3_)PBE/TZVP level of theory) are shown in Fig. 10 below. Like for the planar hydrogen-bonded complexes, we observed a high amount of correlation between the attractive EF between the two ions and the binding energy. This was true for both the Mulliken (*r* = 0.88, Fig. 10a) and the NBO (*r* = 0.80, Fig. 10b) charge analyses. This result further showcases the viability of our approach. Also, as for case (i), we were interested in exploiting our approach for designing new anions that would serve as better counterions than the state-of-the-art, thereby improving the efficiency of the homogeneous olefin polymerization systems. Now, however, unlike in case (i), the focus was upon *reducing* the binding between the two interacting partners. For doing so, we analyzed the nature of the interaction between the two ions for the case where the binding energy had been seen to be the weakest: for the [Cp_2_ZrMe–N{CNB(C_6_F_5_)_3_}_2_] case – the structure shown in Fig. 9b. For this case, the analysis revealed that the –C_6_F_5_ ring that is in direct contact with the cation had the greatest contribution to the EF (–90.5 pN), followed by the central N of the anion (–56.4 pN) (see Table S9†). Based on this information, it became clear that increasing the distance of the zirconium center from the central nitrogen atom in the Fig. 9b structure and/or decreasing the electronic density from the ring directly associated to the cation would lead to a decrease in the EF between the two species. We therefore propose the counterion shown Fig. S16a,† where the fluorides at the *ortho* and *para* positions of the phenyl ring of the counterion have been replaced by CF_3_ groups. The distance between the zirconium and nitrogen atoms in the new complex is seen to have increased: from 5.008 Å in the Fig. 9b structure to 6.538 Å. The EF was seen to be decreased (from –94.1 pN to –88.5 pN for the Mulliken charge analysis case, and from –185.7 pN to –137.1 pN with the NBO charge analysis) and the binding energy was seen to have reduced from –30.7 kcal mol^–1^ to –28.7 kcal mol^–1^, thus indicating that the newly proposed anion would be better than the best counterion for homogeneous olefin polymerization systems. In a further attempt, in order to reduce the attractive interaction of the phenyl ring of the anion that is connected to the cation, we replaced all C_6_F_5_ groups of the Bochmann's anion with the C_4_(CF_3_)_4_N^–^ group (Fig. S16c†), which led to even weaker binding: –23.8 kcal mol^–1^, which is as much as 6.9 kcal weaker than the best Bochmann's anion considered in this study. The computed association constant of this ion-pair system would be 2.2 × 10^6^ times less than the state-of-the-art for this family of complexes, again illustrating the power of the present method to significantly improve upon existing systems. An electrostatic potential map of this anion shows weaker negative potential on its surface than Bochmann's best anion (Fig. S10†), which further corroborates our results.

## Scope of the work

The sections above have demonstrated how the current approach can be effective for two completely diverse families of complexes, which shows its general applicability. It is likely, therefore, that the approach can be useful in providing deeper insights into many other areas of chemistry as well. Biomolecules, for instance, are primarily governed by hydrogen bonds that are dominated by long range electrostatic interactions. Furthermore, the unfolding behavior of proteins in acidic and basic media, as well as in certain salt solutions, can be better understood with the current approach. The mobility of ions in a liquid is significantly governed by long range solute–solvent electrostatic interactions. The current approach would also help in understanding the behavior of hydrogen-bonded solvents – this is because long range SEI have been found^22–32^ to be impactful in determining the H-bond strength. The importance of hydrogen bonding and electrostatic interactions has also been seen in foldamer chemistry.^4,13,14^ It is also to be noted that the exciting and rapidly developing field of ionic liquids would be highly benefited by an understanding of long range electrostatic interactions, as the behavior and solvation properties of solutes in ionic liquids will depend on their SEI. Furthermore, noncovalent interactions have recently been exploited in stereochemical induction, where the approach of prochiral substrates to the chiral catalysts was allowed only from a specific direction.^1–4,69^ Our EF analysis approach would provide meaningful insights into the mechanistic understanding of such reactions, and that would help in tuning such systems for superior catalytic performance. This model could also be useful in understanding the behavior of ionic crystals.

## Conclusions

The current work showcases a simple method based on evaluating the electrostatic force (EF) of interaction between two partners in molecular complexes where the noncovalent electrostatic interaction is the dominant factor. Excellent correlation is seen between the EF and the binding energy of two partners, for two completely different families of complexes. The significance of the work lies in the fact that such an approach provides insight into the nature of the bonding in the different systems studied, and can be exploited to design new systems with significantly increased or decreased binding, as desired, in comparison to the state-of-the-art. More importantly, the current work emphasizes the significance of long range secondary interactions between all atoms of one binding partner with all atoms of the other. Multifold increase and decrease in binding energies has been obtained by altering distant atoms in the noncovalently bonded partners. The work also shows that the consideration of directionality in defining such interactions is important. Given the diverse areas of chemistry where long range electrostatic interactions play a significant role in determining the strength of interaction, the current approach is of significant relevance.
