# Supplementary material for: Exploiting directional long range secondary forces for regulating electrostatics-dominated noncovalent interactions
Source: Chem Sci. 2016 Oct 11;8(2):1378–90. doi: 10.1039/c6sc03642b (PMC5361874; doi:10.1039/c6sc03642b)
Supplement: Supplementary file 1 [file SC-008-C6SC03642B-s001.pdf]

Electronic Supplementary Material (ESI) for Chemical Science.
This journal is © The Royal Society of Chemistry 2016

Supplementary Information

Exploiting Directional Long Range Secondary Forces for Regulating Electrostatics Dominated Noncovalent Interactions

Mrityunjay K. Tiwari and Kumar Vanka*

Physical Chemistry Division, CSIR-National Chemical Laboratory, Pune-411008, India

E-mail: k.vanka@ncl.res.in

Table of Contents

Table S1. Electrostatic Forces along the lines making angles 0°, 30°, 60°, 90°, up to 360° from the line connecting center of geometries of frontier atoms of hydrogen bonding partners for a sample set of 16 representative complexes of the planar hydrogen bond family ……………………………….4

Table S2. Binding Energies and Electrostatic Forces for planar hydrogen bonded complexes optimized at the COSMO($CHCl_3$)/PBE/TZVP level of theory using Turbomole 6.4. …………………….5

Table S3. Binding Energies and Electrostatic Forces for planar hydrogen bonded complexes optimized at the CPCM($CHCl_3$)/M06-2X/6-31G** level of theory using Gaussian 09. ……………….6

Table S4. Binding Energy, Interaction Energy and Electrostatic Forces for Nitrogenous Base Complexes optimized at the COSMO($CHCl_3$)/PBE/TZVP level of theory using Turbomole 6.4. ……7

Table S5. Binding Energies and Electrostatic Forces for contact ion-pairs at the COSMO($CHCl_3$)/PBE/TZVP level of theory using Turbomole 6.4. ……………………………………………..8

Table S6. Binding Energies and Electrostatic Forces for hypothetical newly designed cationic AAAA-DDDD complexes obtained at the COSMO($CHCl_3$)/PBE/TZVP level of theory using Turbomole 6.4. ………9

Table S7. Binding Energies for hypothetical newly designed planer hydrogen bonded complexes obtained at the COSMO($CHCl_3$)/PBE/TZVP level of theory using Turbomole 6.4. ……………………10

Table S8. Binding Energies for hypothetical newly designed planer hydrogen bonded complexes obtained at the COSMO/PBE/TZVP level of theory using Turbomole 6.4. ………………………………11

Table S9. Electrostatic Forces analysis for zirconocne complex with best Bochmann's anion on Mulliken charge analysis for the geometry obtained at the COSMO/PBE/TZVP level of theory using Turbomole 6.4. ……………………………………………………………………………………………………12

Figure S1. Flow chart of the Fortran 90 code used for calculating net force between two partners in hydrogen bonded complexes. ……………………………………………………………………………13

Figure S2. Flow chart of the Fortran 90 code used for calculating net force between two partners in Contact ion-pairs case. ………………………………………………………………………………………14

Figure S3. The optimized geometries of planar hydrogen bonded complexes at the COSMO($CHCl_3$)/PBE/TZVP level of theory. ………………………………………………………………………15-17

Figure S4. The optimized geometries of planar hydrogen bonded complexes at the CPCM($CHCl_3$)/M06-2X/6-31G** level of theory. …………………………………………………………….18-21

Figure S5. The optimized geometries of ion-pair complexes at the COSMO($CHCl_3$)/PBE/TZVP level of theory using Turbomole 6.4. ……………………………………………………………………………………22-23

Figure S6. a) A simple model representing the electrostatic interaction between the two partners, each made up of two point charges of the same nature, in a two dimensional plane. ……24-25

Figure S7. The EF vs. E_b Pearson Correlation graph for planar hydrogen bonded molecules for forces calculated along a line perpendicular to the line of direction of hydrogen bonds, by employing Mulliken charges for the geometries obtained at the COSMO($CHCl_3$)/PBE/TZVP level of theory using Turbomole 6.4 package. 26

Figure S8. The optimized geometries of 28 base pairs considered by Popelier *et al.* his QTAIM studies. ……27-31

Figure S9. The optimized geometries of newly designed cationic AAAA-DDDD hydrogen bonded complexes, where C-C bond on the middle region of acceptor partner is replaced with the isoelectronic B-N bonds. ……………………………………………………………………………………………32

Figure S10. The Molecular Surface electrostatic potential in Hartrees, computed on the 0.0004 au contour of the electron density, using the GaussView software at the CPCM($CHCl_3$)/M062X/6-31G**of theory. ……………………………………………………………………..33-35

Figure S11. The optimized geometries of newly designed cationic AAAA-DDDD hydrogen bonded complexes, where hydrogen atoms in frontier lines are replaced with the phenyl groups. ……36

Figure S12. The optimized geometry of hypothetical best case designed cationic AAAA-DDDD hydrogen bonded complex, where hydrogen atoms in frontier lines are replaced with the 2,4,6-trimethylphenyl groups and three C-C bonds in the middle region of acceptor partner is replacer with the isoelectronic B-N bonds. ……………………………………………………………………..37

Figure S13. A schematic picture of some newly designed acceptor-donor planer hydrogen bonded complexes based on our electrostatic force analysis, where central C-C bonds on acceptor moieties are replaced by B-N bonds. ……………………………………………………………..38

Figure S14. A schematic picture of some newly designed acceptor-donor planer hydrogen bonded complexes, where attractive non-directional dispersive force was exploited for improved binding. ………………………………………………………………………………………………………..39

Figure S15. The optimized geometries of Bochmann's anions at the COSMO($CHCl_3$)/PBE/TZVP level of theory using Turbomole 6.4. ………………………………………………………………………………………40

Figure S16. The optimized geometry of the zirconocene complex with newly designed anions. ……41

XYZ coordinates of all the stationary points obtained after full optimization in solvent chloroform ……42-64

Table S1. Electrostatic Forces along the lines making angles 0°, 30°, 60°, 90°, up to 360° from the line connecting center of geometries of frontier atoms of hydrogen bonding partners for a sample set of 16 representative complexes of the planar hydrogen bond family

		0°	30°	60°	90°	120°	150°	180°	210°	240°	270°	300°	330°	360°
**X-1**	a	-163.4	-122.2	-48.3	38.6	115.1	160.8	163.4	122.2	48.3	-38.6	-115.1	-160.8	-163.4
	b	-451.8	-354.8	-162.8	72.9	289.0	427.7	451.8	354.8	162.8	-72.9	-289.0	-427.7	-451.8
	c	-101.3	-77.8	-33.5	19.8	67.8	97.6	101.3	77.8	33.5	-19.8	-67.8	-97.6	-101.3
**X-2**	a	-280.2	-207.8	-79.8	69.6	200.4	277.5	280.2	207.8	79.8	-69.6	-200.4	-277.5	-280.2
	b	-634.9	-524.8	-274.2	49.9	360.7	574.8	634.9	524.8	274.2	-49.9	-360.7	-574.8	-634.9
	c	-152.7	-120.1	-55.3	24.3	97.4	144.4	152.7	120.1	55.3	-24.3	-97.4	-144.4	-152.7
**X-3**	a	-253.6	-190.0	-75.6	59.1	178.0	249.1	253.6	190.0	75.6	-59.1	-178.0	-249.1	-253.6
	b	-541.1	-436.2	-241.5	17.9	272.5	454.1	514.1	436.2	241.5	-17.9	-272.5	-454.1	-514.1
	c	-133.8	-106.6	-51.1	18.2	82.7	125.0	133.8	106.6	51.1	-18.2	-82.7	-125.0	-133.8
**X-4**	a	-135.1	-96.6	-32.3	40.7	102.8	137.4	135.1	96.6	32.3	-40.7	-102.8	-137.4	-135.1
	b	-609.9	-447.7	-165.6	160.9	444.3	608.7	609.9	447.7	165.6	-160.9	-444.3	-608.7	-609.9
	c	-81.8	-59.7	-21.6	22.3	60.2	82.0	81.8	59.7	21.6	-22.3	-60.2	-82.0	-81.8
**X-5**	a	-326.4	-242.7	-93.9	80.0	232.5	322.7	326.4	242.7	93.9	-80.0	-232.5	-322.7	-326.4
	b	-667.5	-562.9	-307.6	30.2	359.9	593.1	667.5	562.9	307.6	-30.2	-359.9	-593.1	-667.5
	c	-161.8	-130.8	-64.8	18.6	97.1	149.5	161.8	130.8	64.8	-18.6	-97.1	-149.5	-161.8
**X-6**	a	-202.8	-148.4	-54.3	54.4	148.5	202.8	202.8	148.4	54.3	-54.4	-148.5	-202.8	-202.8
	b	-487.3	-409.9	-222.7	24.3	264.7	434.2	487.3	409.9	222.7	-24.3	-264.7	-434.2	-487.3
	c	-117.0	-93.1	-44.2	16.5	72.8	109.6	117.0	93.1	44.2	-16.5	-72.8	-109.6	-117.0
**X-7**	a	-267.0	-195.1	-71.0	72.2	196.0	267.3	267.0	195.1	71.0	-72.2	-196.0	-267.3	-267.0
	b	-568.4	-484.9	-271.5	14.7	296.9	499.6	568.4	484.9	271.5	-14.7	-296.9	-499.6	-568.4
	c	-151.3	-120.1	-56.7	21.9	94.7	142.0	151.3	120.1	56.7	-21.9	-94.7	-142.0	-151.3
**X-8**	a	-450.8	-333.7	-127.2	113.4	323.6	447.1	450.8	333.7	127.2	-113.4	-323.6	-447.1	-450.8
	b	-812.1	-680.5	-366.6	45.6	445.5	726.1	812.1	680.5	366.6	-45.6	-445.5	-726.1	-812.1
	c	-245.9	-193.6	-89.3	38.8	156.6	232.4	245.9	193.6	89.3	-38.8	-156.6	-232.4	-245.9
**X-9**	a	-75.8	-51.6	-13.5	28.2	62.3	79.7	75.8	51.6	13.5	-28.2	-62.3	-79.7	-75.8
	b	-321.0	-256.2	-122.7	43.7	198.3	299.8	321.0	256.2	122.7	-43.7	-198.3	-299.8	-321.0
	c	-66.1	-51.1	-22.4	12.3	43.7	63.4	66.1	51.1	22.4	-12.3	-43.7	-63.4	-66.1
**X-10**	a	-95.5	-58.4	-5.7	48.5	89.8	107.0	95.5	58.4	5.7	-48.5	-89.8	-107.0	-95.5
	b	-307.7	-262.0	-146.1	9.0	161.7	271.0	307.7	262.0	146.1	-9.0	-161.7	-271.0	-307.7
	c	-89.3	-70.7	-33.1	13.3	56.2	84.0	89.3	70.7	33.1	-13.3	-56.2	-84.0	-89.3
**X-11**	a	-521.1	-399.3	-170.6	103.9	350.5	503.2	521.1	399.3	170.6	-103.9	-350.5	-503.2	-521.1
	b	-1173.3	-931.5	-440.1	169.2	733.2	1100.7	1173.3	931.5	440.1	-169.2	-733.2	-1100.7	-1173.3
	c	-241.0	-190.4	-88.7	36.7	152.3	227.1	241.0	190.4	88.7	-36.7	-152.3	-227.1	-241.0
**X-12**	a	-273.3	-212.8	-96.2	46.1	176.0	258.8	272.3	212.8	96.2	-46.1	-176.0	-258.8	-273.3
	b	-869.5	-684.0	-315.2	138.1	554.3	822.0	869.5	684.0	315.2	-138.1	-554.3	-822.0	-869.5
	c	-163.2	-129.9	-61.8	22.9	101.4	152.8	163.2	129.9	61.8	-22.9	-101.4	-152.8	-163.2
**X-13**	a	-301.8	-208.6	-59.5	105.5	242.2	314.1	301.8	208.6	59.5	-105.5	-242.2	-314.1	-301.8
	b	-674.3	-455.8	-115.1	256.4	559.2	712.2	674.3	455.8	115.1	-256.4	-559.2	-712.2	-674.3
	c	-160.0	-107.8	-26.8	61.5	133.2	169.3	160.0	107.8	26.8	-61.5	-133.2	-169.3	-160.0
**X-14**	a	-167.9	-135.3	-66.4	20.3	101.6	155.6	167.9	135.3	66.4	-20.3	-101.6	-155.6	-167.9
	b	-680.4	-547.3	-267.6	83.9	412.8	631.2	680.4	547.3	267.6	-83.9	-412.8	-631.2	-680.4
	c	-164.7	-139.6	-77.0	6.1	87.7	145.7	164.7	139.6	77.0	-6.1	-87.7	-145.7	-164.7
**X-15**	a	-243.8	-185.2	-77.0	51.8	166.8	237.0	243.8	185.2	77.0	-51.8	-166.8	-237.0	-243.8
	b	-846.0	-670.8	-315.8	123.8	530.3	794.6	846.0	670.8	315.8	-123.8	-530.3	-794.6	-846.0
	c	-166.2	-126.8	-53.4	34.3	112.8	161.1	166.2	126.8	53.4	-34.3	-112.8	-161.1	-166.2
**X-**	a	-105.0	-74.5	-24.0	32.9	81.0	107.4	105.0	74.5	24.0	-32.9	-81.0	-107.4	-105.0

**16**	b	-621.9	-459.1	-173.4	158.9	448.5	618.0	621.9	459.1	173.4	-158.9	-448.5	-618.0	-621.9
	c	-108.0	-78.5	-28.1	29.9	79.9	108.5	108.0	78.5	28.1	-29.9	-79.9	-108.5	-108.0

a = computed electrostatic force using Mulliken Charges; b = computed electrostatic force using NBO Charges; c = computed electrostatic force using Lowdin Charges; All forces are in pN; Please see the Figure S3 below for the optimized geometry of X-1 to X-16.

Table S2. Binding Energies and Electrostatic Forces for planar hydrogen bonded complexes optimized at the COSMO ($CHCl_3$)/PBE/TZVP level of theory using Turbomole 6.4.

Planar hydrogen bonded complex	Binding Energy	EF (with Mulliken Charges analysis)	EF (with NBO Charges analysis)
**X-1**	-15.4	-163.4	-451.8
**X-2**	-29.2	-280.2	-634.9
**X-3**	-19.3	-253.6	-514.1
**X-4**	-14.9	-135.1	-609.9
**X-5**	-29.4	-326.4	-667.5
**X-6**	-18.0	-202.8	-487.3
**X-7**	-22.3	-267.0	-568.4
**X-8**	-42.9	-450.8	-812.1
**X-9**	-14.3	-75.8	-321.0
**X-10**	-17.3	-167.6	-307.7
**X-11**	-43.4	-521.1	-1173.3
**X-12**	-26.4	-272.3	-869.5
**X-13**	-33.9	-301.8	-674.3
**X-14**	-20.7	-167.9	-680.4
**X-15**	-30.9	-243.8	-846.0
**X-16**	-20.6	-105.0	-621.9

EF=Electrostatic Force

All forces are in pN

All energies are in kcal/mol.

Please see the Figure S3 below for the optimized geometry of X-1 to X-16.

Table S3. Binding Energies and Electrostatic Forces for planar hydrogen bonded complexes optimized at the CPCM($CHCl_3$)/M06-2X/6-31G level of theory using Gaussian 09.**

Planar hydrogen bonded complex	Binding Energy	EF (with Mulliken Charges analysis)	EF (with NBO Charges analysis)
**Y-1**	-15.8	-183.1	-468.1
**Y-2**	-28.3	-418.3	-689.8
**Y-3**	-19.3	-154.1	-242.4
**Y-4**	-16.0	-338.4	-489.7
**Y-5**	-28.3	-360.3	-648.5
**Y-6**	-20.0	-272.8	-526.9
**Y-7**	-23.6	-297.2	-415.1
**Y-8**	-17.3	-125.8	-391.4
**Y-9**	-41.1	-801.4	-1283.8
**Y-10**	-28.3	-446.1	-889.2
**Y-11**	-28.3	-575.7	-635.0
**Y-12**	-30.2	-555.2	-1021.6
**Y-13**	-18.7	-315.1	-613.8
**Y-14**	-28.0	-598.4	-1111.4
**Y-15**	-20.3	-387.9	-699.0
**Y-16**	-18.0	-165.7	-614.4

EF=Electrostatic Force

All forces are in pN

All energies are in kcal/mol.

Please see Figure S4 below for the optimized geometries of Y-1 to Y-16.

Table S4. Binding Energy, Interaction Energy and Electrostatic Forces for Nitrogenous Base Complexes optimized at the COSMO($CHCl_3$)/PBE/TZVP level of theory using Turbomole 6.4.

Molecule Name	Binding Energy	Interaction Energy	EF (with Mulliken Charges analysis)	EF (with NBO Charges analysis)
**AA1**	-11.8	-13.1	-297.2	-275.5
**AA2**	-11.3	-12.5	-154.5	-451.8
**AA3**	-10.0	-11.0	-17.2	-471.1
**AC1**	-13.0	-14.4	-360.2	-448.8
**AC2**	-12.7	-14.0	-230.4	-608.4
**AT (H)**	-11.6	-14.4	-270.3	-772.0
**AT (RH)**	-12.5	-14.0	-289.9	-733.8
**AT (RWC)**	-11.1	-14.1	-330.7	-520.0
**AT (WC)**	-12.7	-14.6	-408.8	-554.5
**CC**	-14.9	-16.6	-409.0	-607.4
**GA1**	-14.0	-15.7	-381.3	-702.2
**GA2**	-10.1	-11.4	-146.9	-424.6
**GA3**	-13.4	-15.0	-244.7	-716.4
**GA4**	-11.2	-12.6	-266.1	-340.7
**GC**	-21.5	-23.8	-577.0	-1110.0
**GC1**	-12.0	-12.7	-316.7	-823.1
**GC2**	-12.6	-14.1	-335.2	-374.4
**GG1**	-18.3	-20.7	-535.3	-1620.0
**GG3**	-15.2	-16.1	-304.3	-707.7
**GG4**	-9.7	-10.9	-227.2	-315.5
**GT1**	-13.2	-14.9	-412.7	-608.4
**GT2**	-11.1	-13.7	-343.8	-462.7
**TC1**	-9.6	-11.1	-272.6	-340.7
**TC2**	-8.9	-10.2	-239.0	-351.4
**TT1**	-8.7	-11.0	-309.1	-438.6
**TT2**	-10.4	-11.8	-343.1	-455.6
**TT3**	-8.2	-10.4	-255.8	-370.8
**U-DAP**	-14.6	-17.1	-440.1	-884.0

EF=Electrostatic Force

All forces are in pN

All energies are in kcal/mol.

Representation of molecular complexes has been taken from the Popelier's paper cited into the manuscript. The optimized geometries are provided into Figure S8 below.

Table S5. Binding Energies and Electrostatic Forces for contact ion-pairs at the COSMO($CHCl_3$)/PBE/TZVP level of theory using Turbomole 6.4.

Anion	Binding Energy	EF (with Mulliken Charges analysis)	EF (with NBO Charges analysis)
**Z-1**	-35.1	-300.5	-400.0
**Z-2**	-35.2	-215.1	-242.4
**Z-3**	-56.9	-393.4	-510.8
**Z-4**	-51.3	-288.3	-332.2
**Z-8**	-56.8	-500.9	-975.2
**Z-6**	-34	-220.0	-268.7
**Z-4**	-30.8	-140.4	-201.0
**Z-5**	-30.7	-94.1	-185.7

EF=Electrostatic Force

All forces are in pN

All energies are in kcal/mol.

Please see the Figure S5 below for the optimized geometries of **Z-1** to **Z-8**.

Table S6. Binding Energies and Electrostatic Forces for hypothetical newly designed cationic AAAA-DDDD complexes obtained at the COSMO($CHCl_3$)/PBE/TZVP level of theory using Turbomole 6.4.

Designed planar AAAA-DDDD cationic complex	Binding Energy	EF (with Mulliken Charges analysis)	EF (with NBO Charges analysis)
**A-1**	-49.6	-675.119	-1501.921
**A-2**	-49.2	-802.126	-1535.602
**A-3**	-55.7	-828.445	-1868.424
**A-4**	-60.2	-1264.118	-2202.395
**B-1**	-50.2	-641.838	-1313.852
**B-2**	-50.8	-524.964	-1127.156
**C**	-69.6	-1411.299	-2795.471

EF=Electrostatic Force

All forces are in pN

All energies are in kcal/mol.

Please refer Figures S9, S11 and S12 below to see the optimized geometries **A-1** to **C**.

Table S7. Binding Energies for hypothetical newly designed planer hydrogen bonded complexes obtained at the COSMO($CHCl_3$)/PBE/TZVP level of theory using Turbomole 6.4.

Designed planar AAAA-DDDD cationic complex	Binding Energy	ΔE [a]
**D-1**	-17.3	1.9
**D-2**	-37.2	8.0
**D-3**	-23.8	4.5
**D-4**	-45.2	11.3

a= increase in binding strength, which was compared with respect to the corresponding synthesized parental complexes wherefrom they are derived.

All energies are in kcal/mol.

Please refer Figure S13 below to for the structures of **D-1** to **D-4**.

Table S8. Binding Energies for hypothetical newly designed planer hydrogen bonded complexes obtained at the COSMO/PBE/TZVP level of theory using Turbomole 6.4.

Designed planar AAAA-DDDD cationic complex	Binding Energy	ΔE [a]
**E-1**	-47.3	3.9
**E-2**	-45.9	2.5
**E-3**	-46.4	3.0
**E-4**	-45.3	1.9

a= increase in binding strength, which was compared with respect to the corresponding synthesized parental complexes wherefrom they are derived.

All energies are in kcal/mol.

Please refer Figure S14 below for the structures of **E-1** to **E-4**.

Table S9. Electrostatic Forces analysis for zirconocne complex with best Bochmann's anion on Mulliken charge analysis for the geometry obtained at the COSMO/PBE/TZVP level of theory using Turbomole 6.4.

Region	1	2	3	4	5	6	7	8	9	10	11
Force	-90.5	-2.1	-9.6	22.6	38.4	-56.4	31.5	6.0	-6.9	-14.9	-12.2

All the Forces are in pN.

Please see the following attached structure to refer the corresponding regions of the molecule.

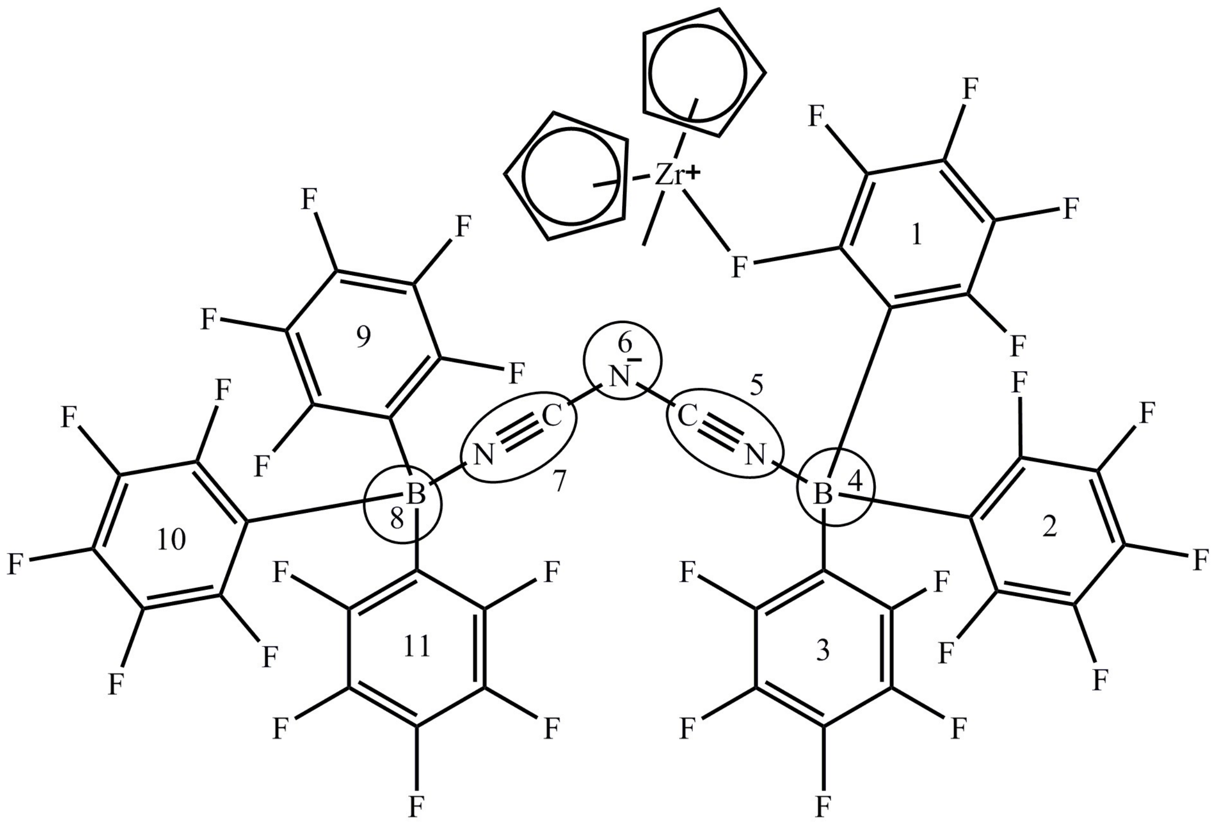

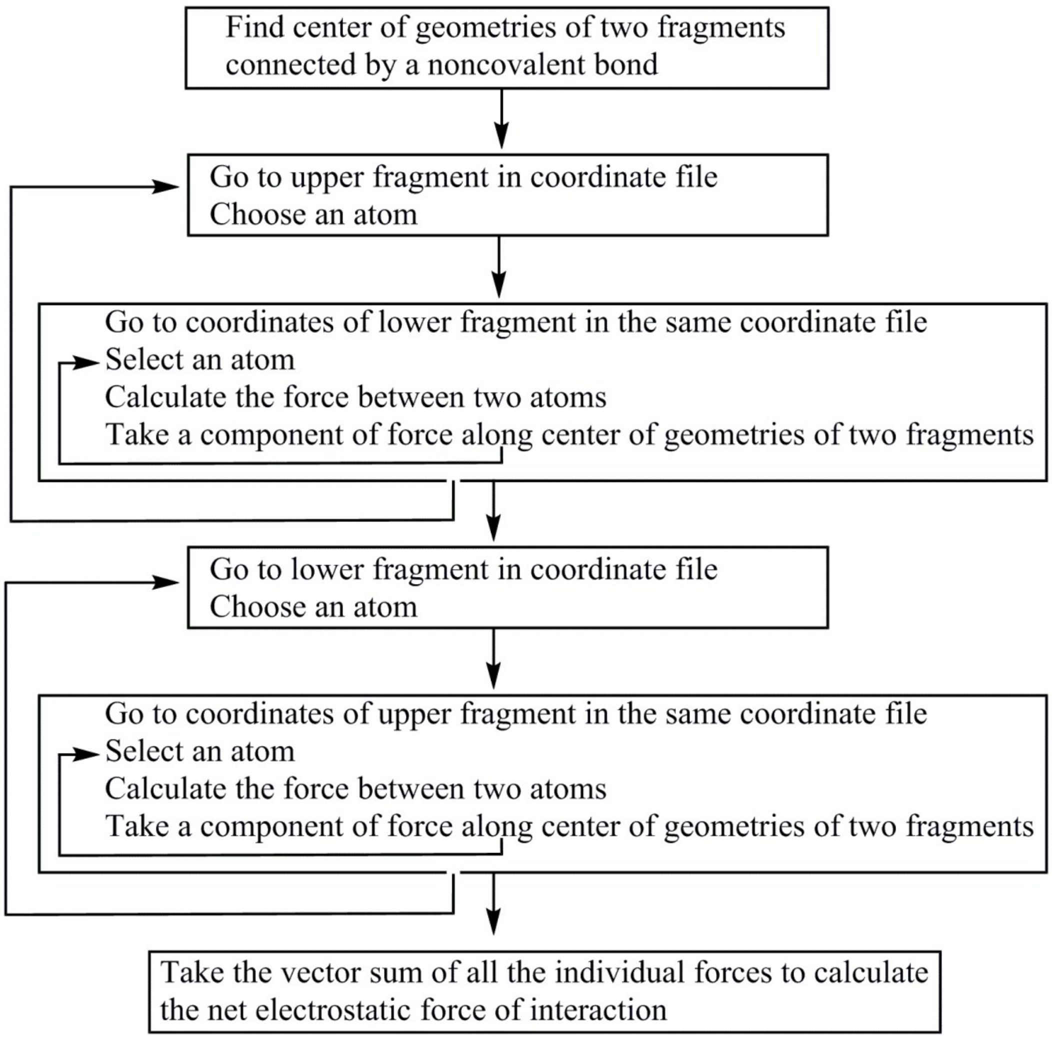

Figure S1. Flow chart of the Fortran 90 code used for calculating net force between two partners in hydrogen bonded complexes.

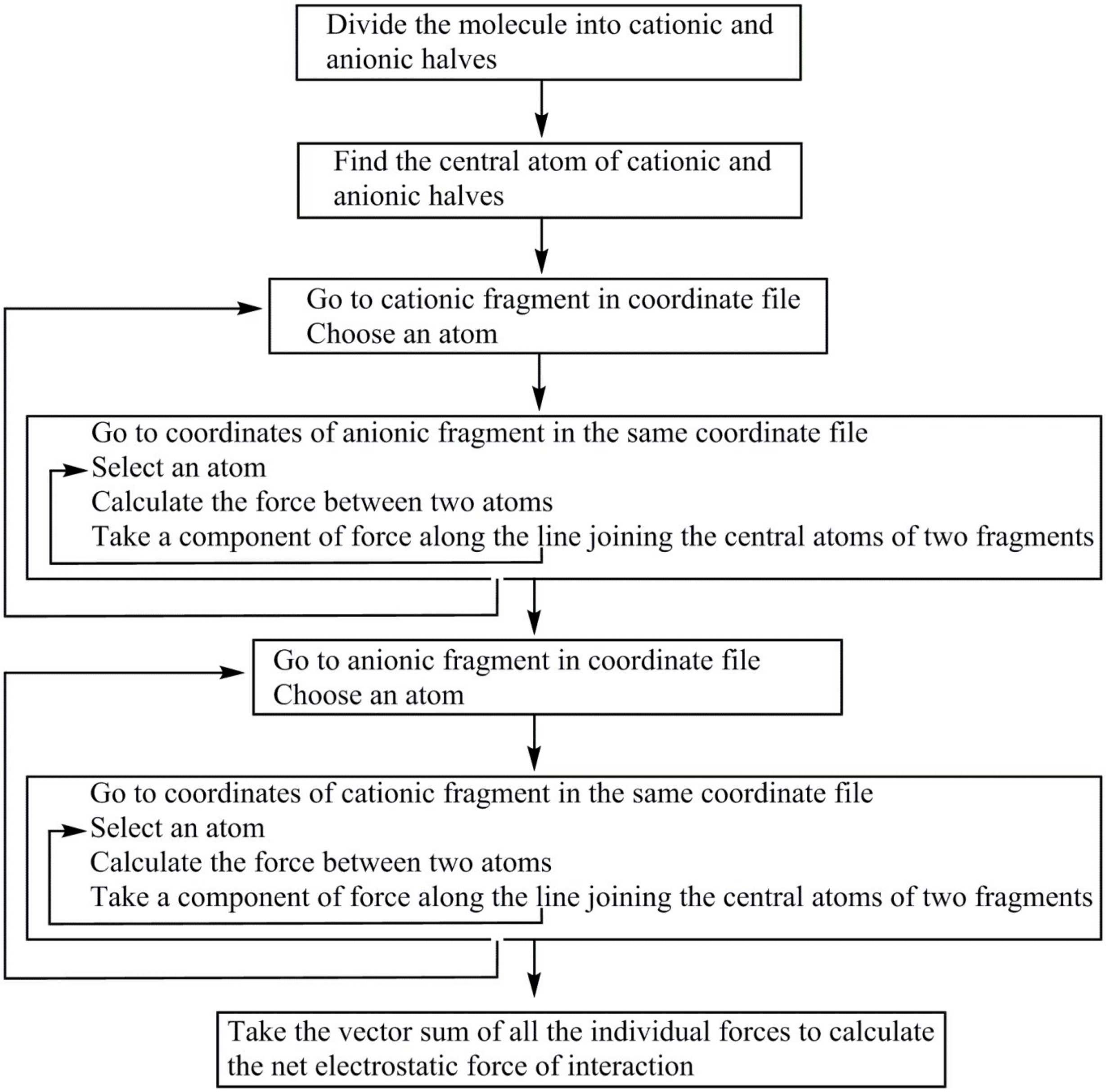

Figure S2. Flow chart of the Fortran 90 code used for calculating net force between two partners in Contact ion-pairs case.

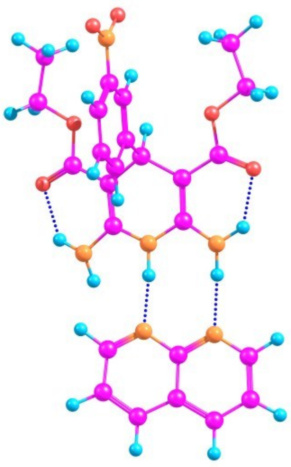

X-1

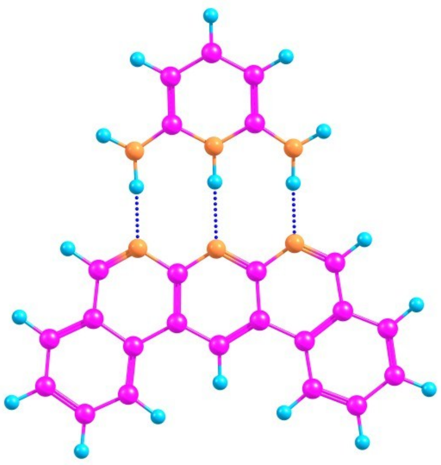

X-2

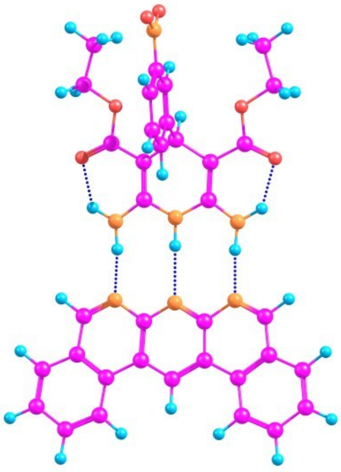

X-3

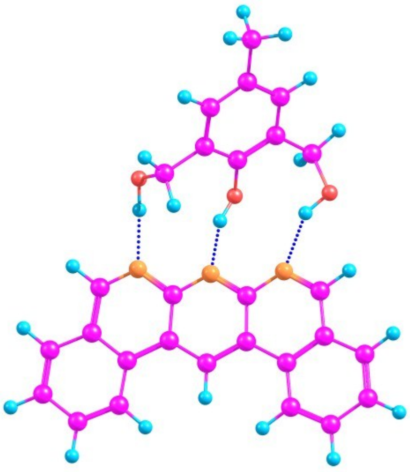

X-4

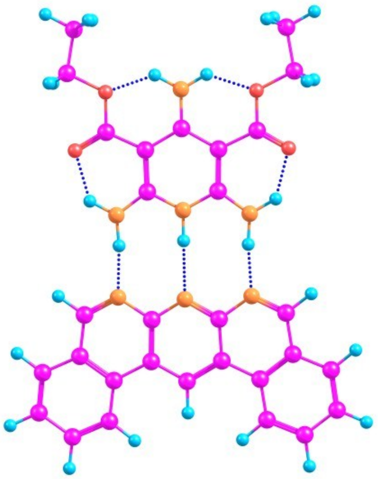

X-5

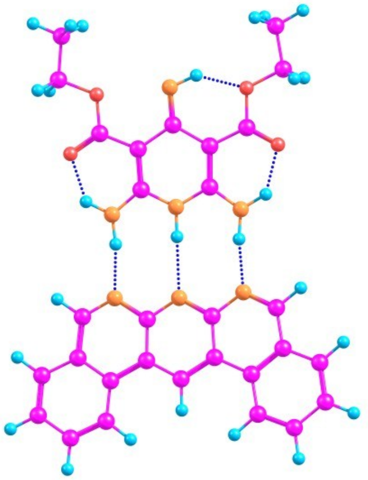

X-6

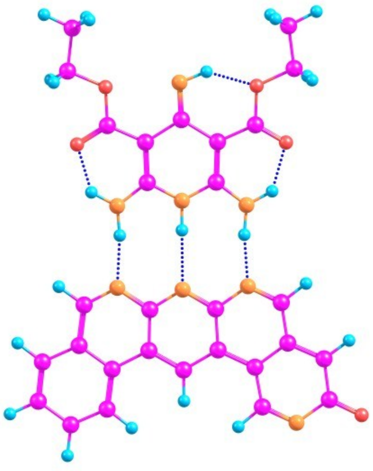

X-7

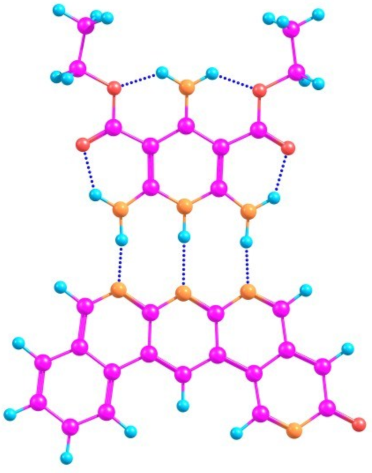

X-8

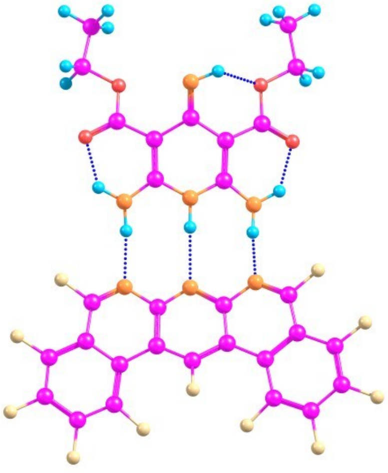

X-9 (continued)

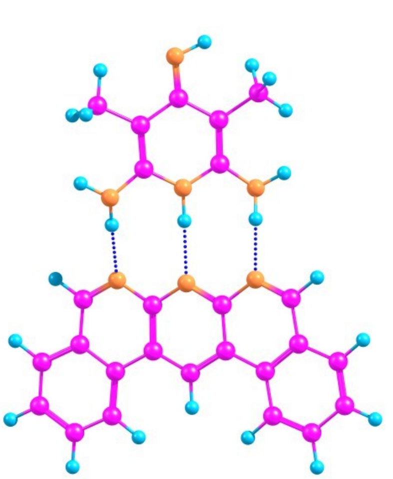

X-10

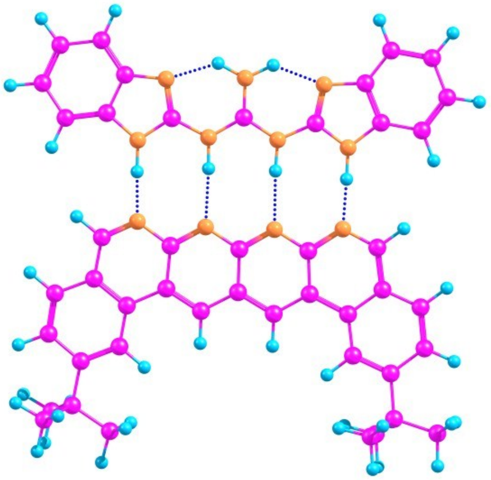

X-11

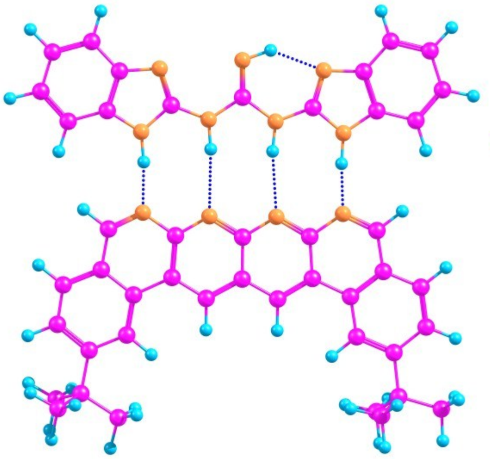

X-12

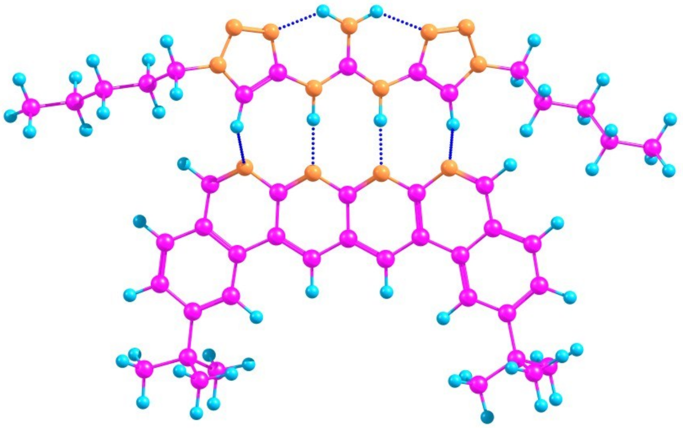

X-13

(Figure continued)

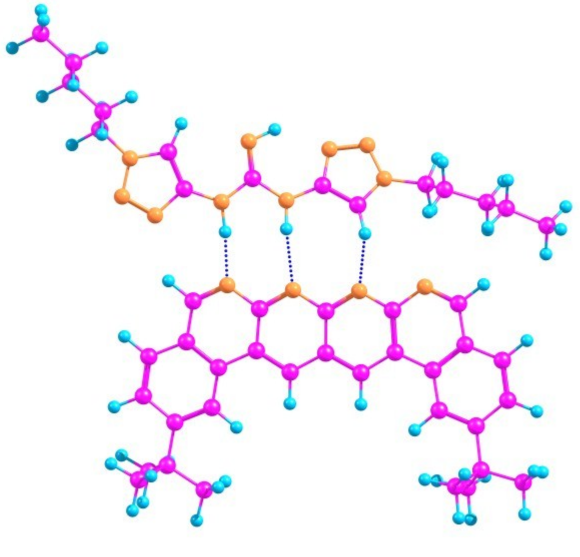

X-14

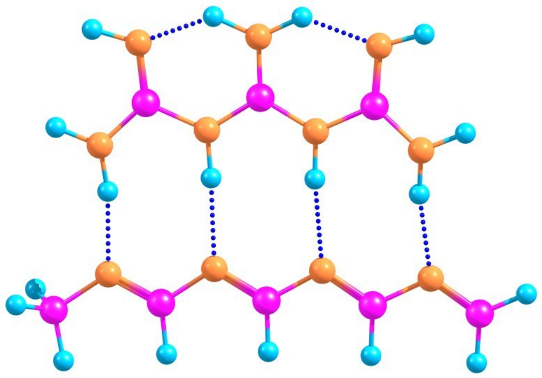

X-15

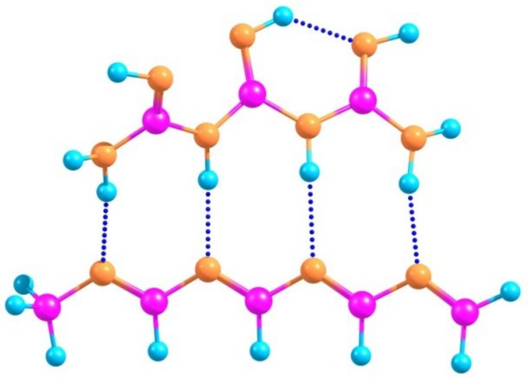

X-16

Figure S3. The optimized geometries of planar hydrogen bonded complexes at the COSMO($CHCl_3$)/PBE/TZVP level of theory. Pink, cyan, brown and white colors represent carbon, hydrogen, nitrogen and fluorine atoms respectively, whereas, dotted blue lines represent hydrogen bonds. **X-5** to **X-10, X-15** and **X-16** are optimized geometries of modeled complexes that are obtained after modification on non-frontier region of corresponding Leigh's complexes.

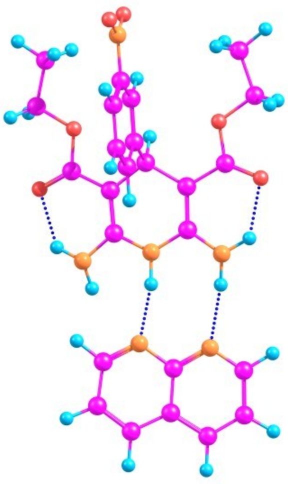

Y-1

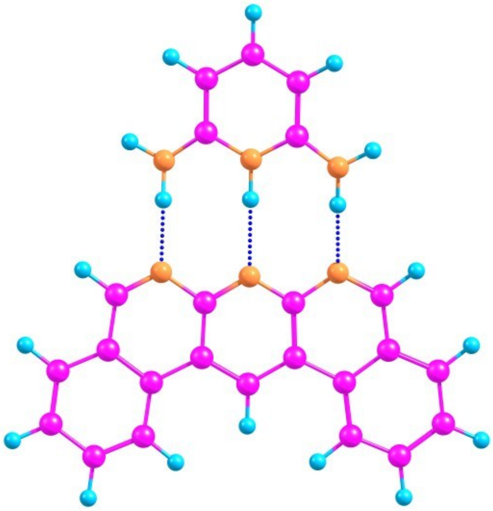

Y-2

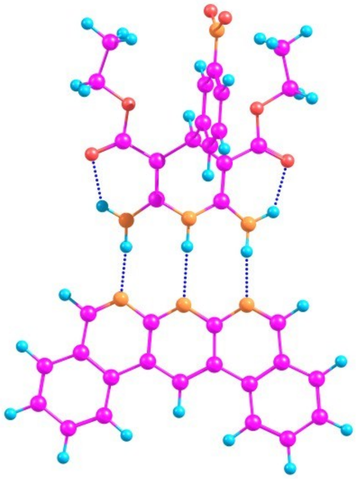

Y-3

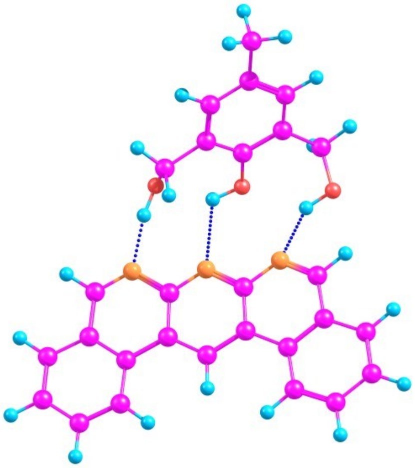

Y-4

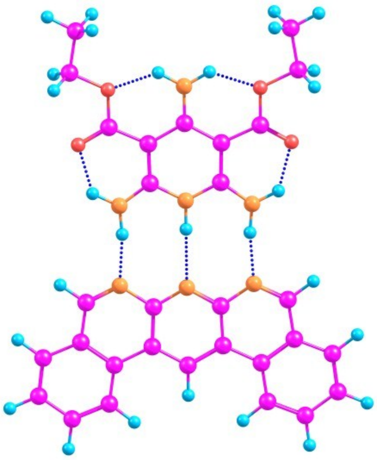

Y-5

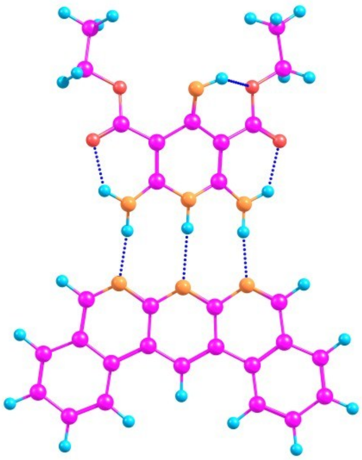

Y-6

(Figure Continued)

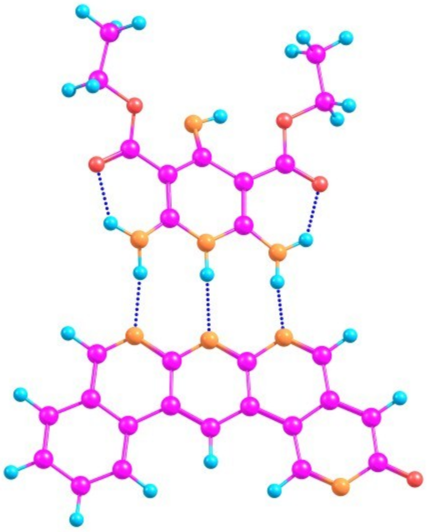

Y-7

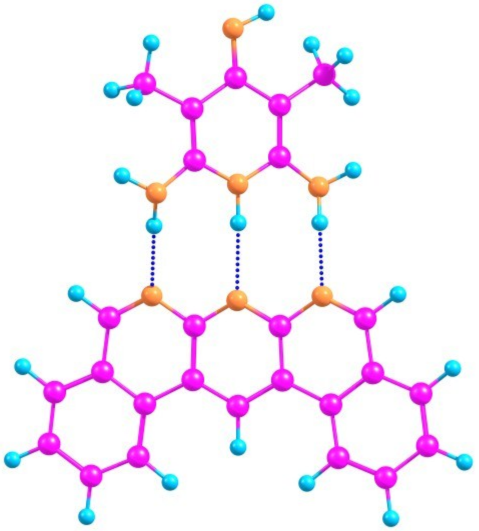

Y-8

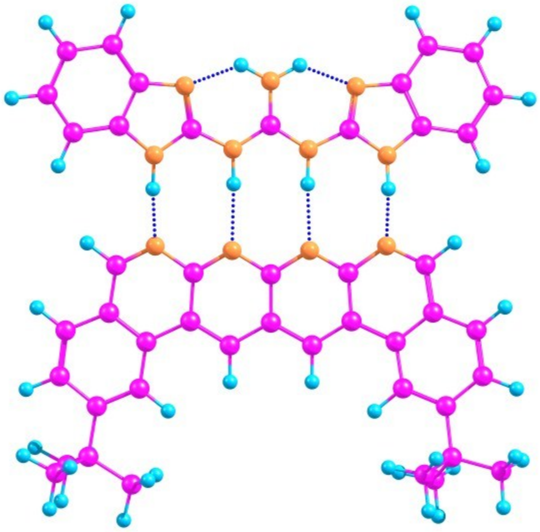

Y-9

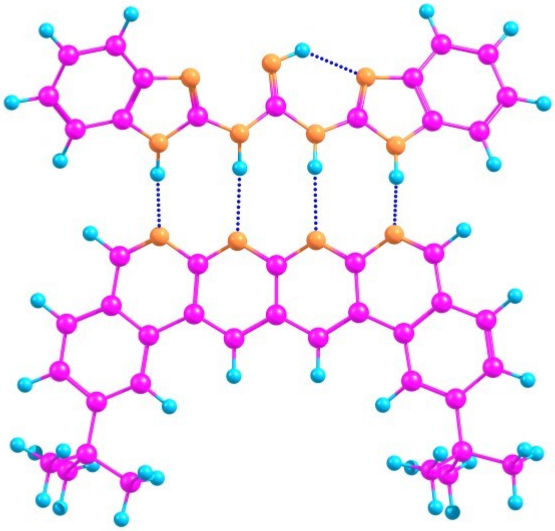

Y-10

(Figure Continued)

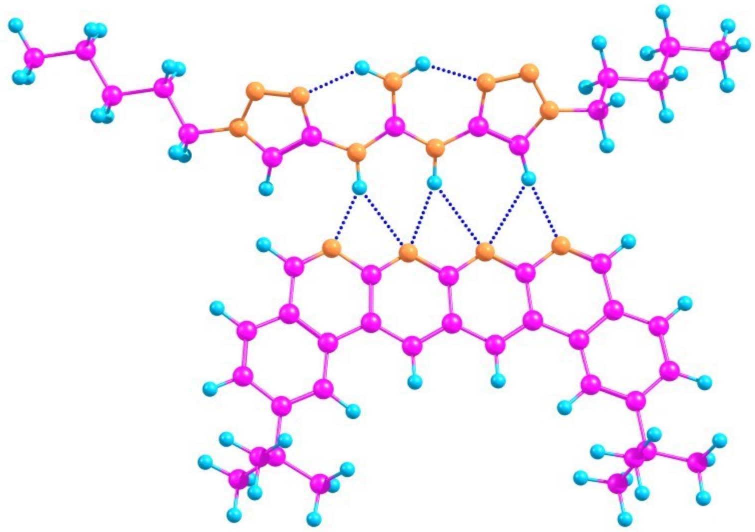

Y-11

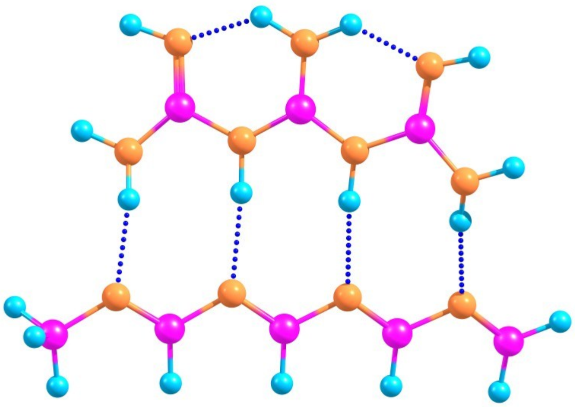

Y-12

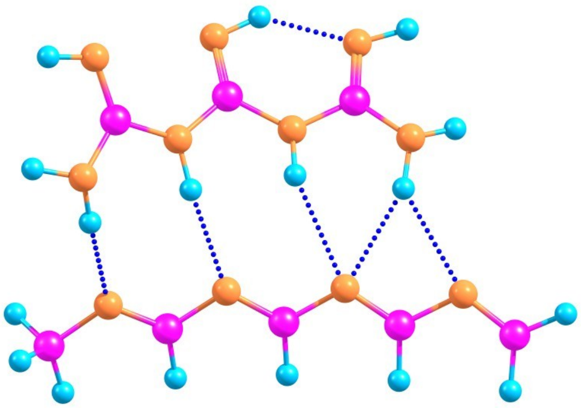

Y-13

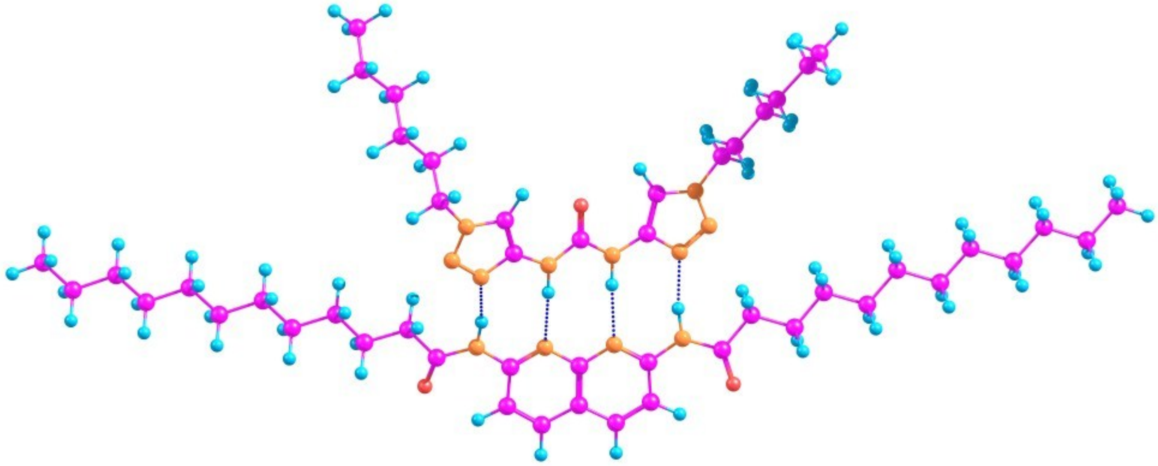

Y-14 (Figure continued)

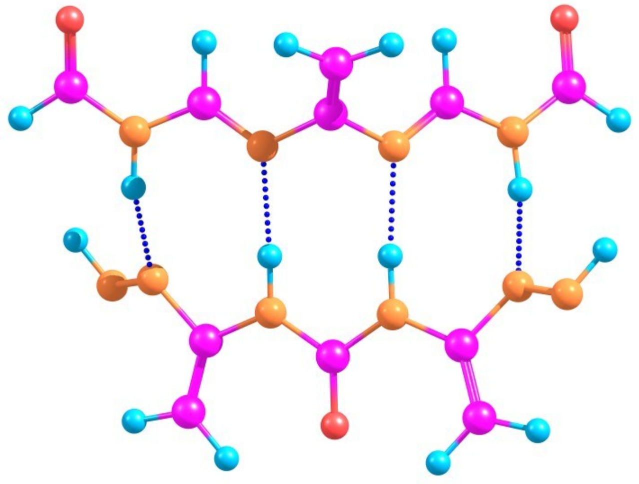

Y-15

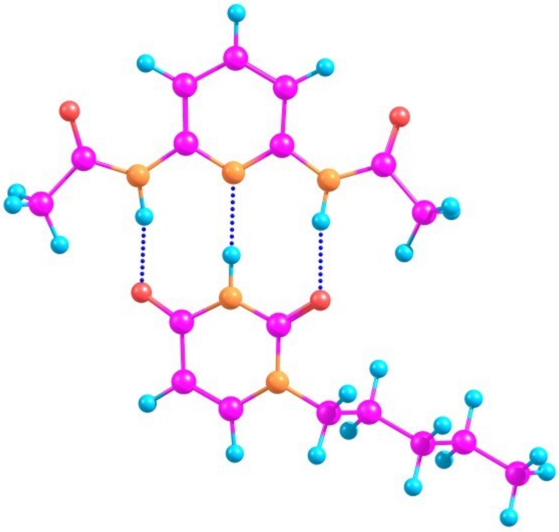

Y-16

Figure S4. The optimized geometries of planar hydrogen bonded complexes at the CPCM($CHCl_3$)/M06-2X/6-31G** level of theory. Pink, cyan, brown and white colors represent carbon, hydrogen, nitrogen and fluorine atoms respectively, whereas, dotted blue lines represent hydrogen bonds. **Y-5** and **Y-12** to **Y-14** are optimized geometries of modeled complexes that are obtained after modification on non-frontier region of corresponding Leigh's or Yosuke complexes.

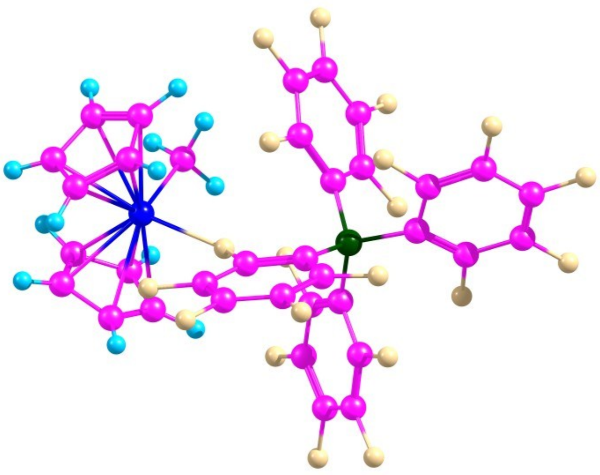

Z-1

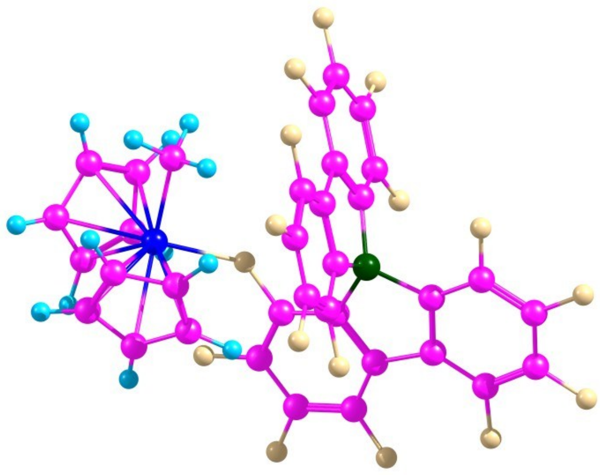

Z-2

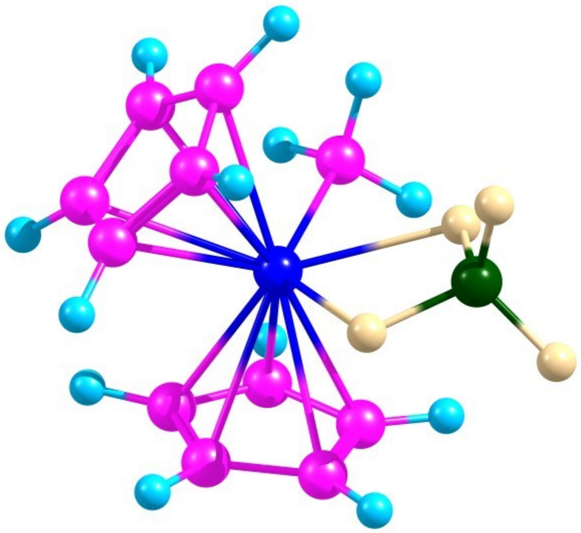

Z-3

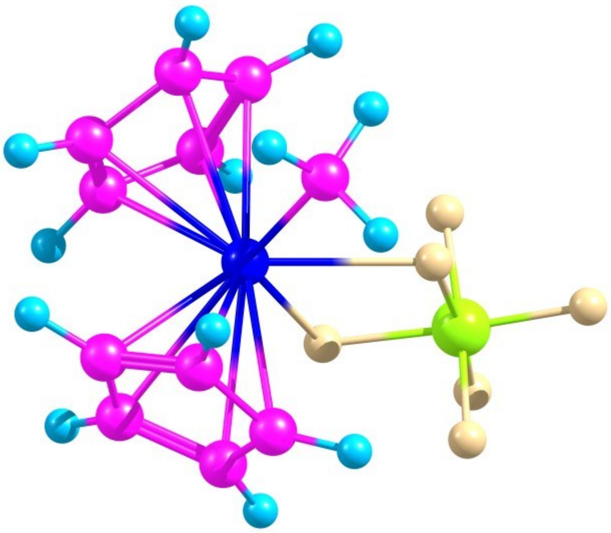

Z-4

(Figure Continued)

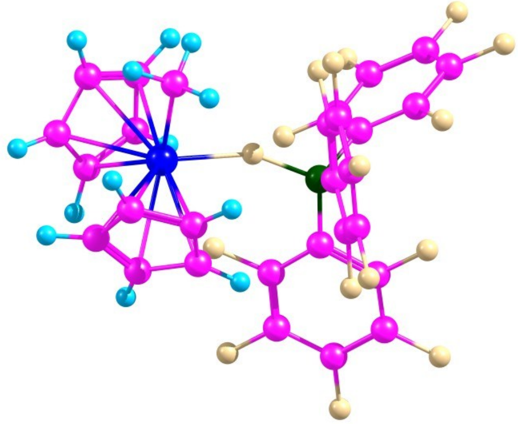

Z-5

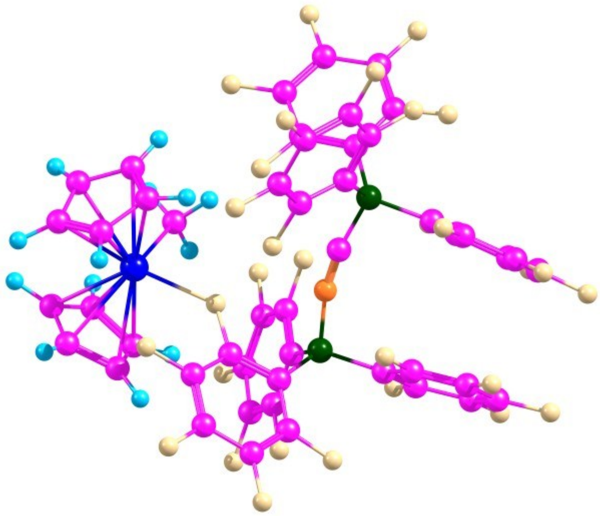

Z-6

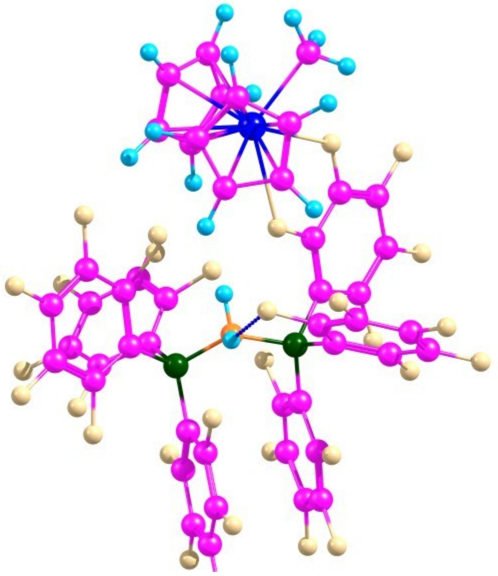

Z-7

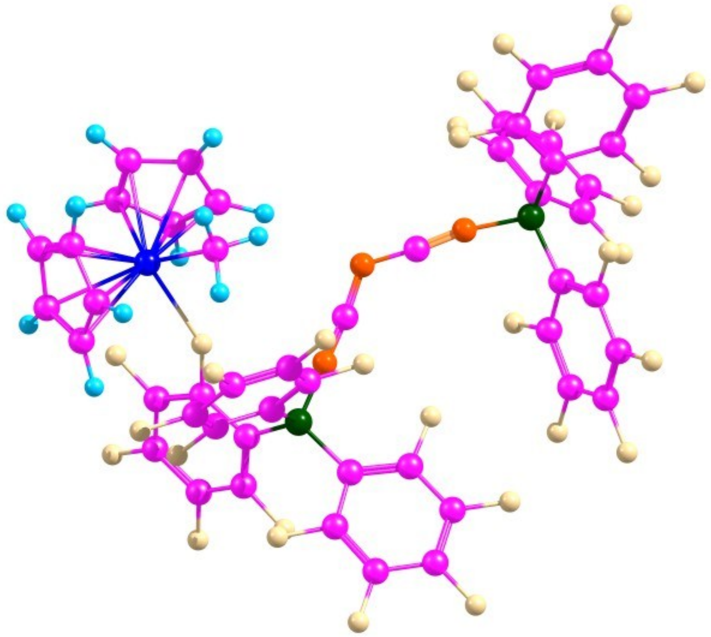

Z-8

Figure S5. The optimized geometries of ion-pair complexes at the COSMO($CHCl_3$)/PBE/TZVP level of theory using Turbomole 6.4. Pink, cyan, brown, green, blue, lime and white colors represent carbon, hydrogen, nitrogen, boron, zirconium, phosphorous and fluorine atoms respectively, whereas, dotted blue lines represent hydrogen bonds.

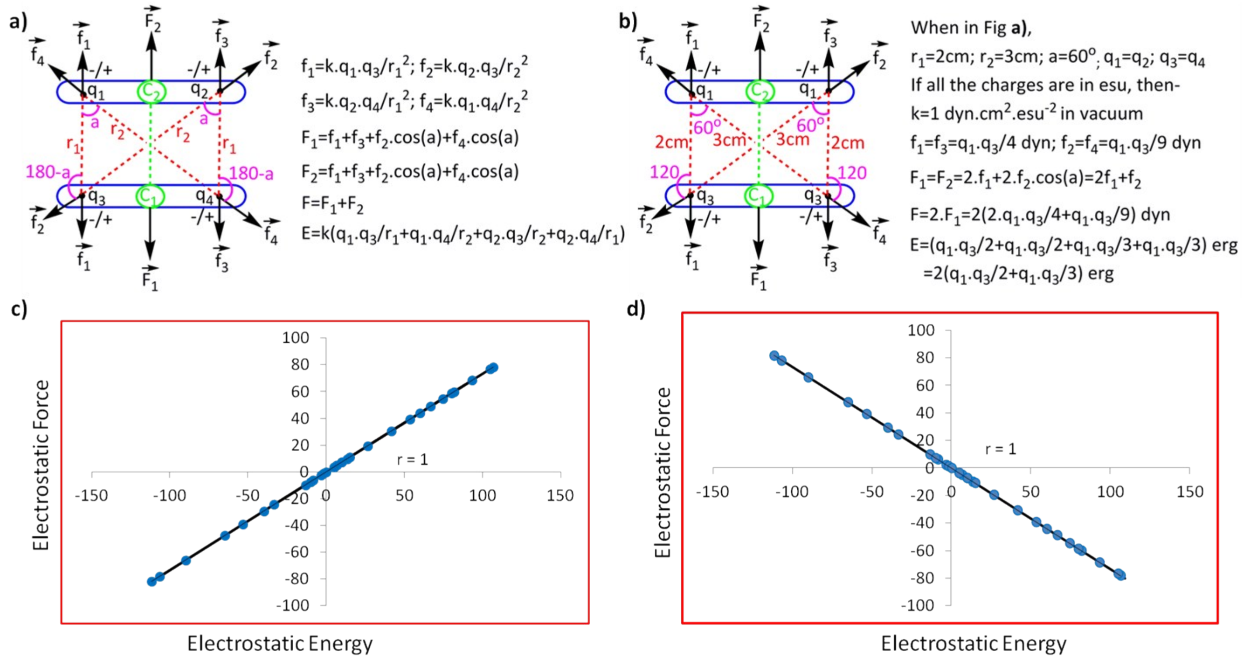

Figure S6. a) A simple model representing the electrostatic interaction between the two partners, each made up of two point charges of the same nature, in a two dimensional plane. f_1, f_2, f_3, f_4 represent the magnitude of forces experienced by the corresponding charged particles due to the charges on the other partner. C_1 and C_2 are the center of geometries of the two particles on the respective partners. The line joining C_1 and C_2 is the line of direction. F_1 and F_2 are the magnitude of forces experienced by the respective partners. F is the magnitude of the force of interaction between two partners. E is the electrostatic energy of interaction. b) A further simplified model when magnitude and nature of both the charges on each fragment are the same. c) The Pearson correlation graph between the electrostatic force of interaction along the direction of approach of the two partners and the electrostatic energy of interaction under the conditions described in b) for a set of 30 different values of q_1 and q_2. The forces are in dyn and the energies are in erg. d) The Pearson correlation graph between the electrostatic force of interaction along the direction opposite to the direction of approach of the two partners and the electrostatic energy of interaction under the conditions described in b) for the same set of 30 different values of q_1 and q_2.

The values of the force and the energies obtained for different values of q_1 and q_2 are provided below:

q1	q3	E	F (at 0°)	F (at 180°)
3	-18	-90.0	-66.0	66.0
2	-4	-13.3	-9.8	9.8
3	-13	-65.0	-47.7	47.66666667
4	4	26.66666667	19.55555556	-19.55555556
5	5	41.66666667	30.55555556	-30.55555556
6	6	60.0	44.0	-44.0
7	7	81.66666667	59.88888889	-59.88888889
8	8	106.6666667	78.22222222	-78.22222222
8	1	13.33333333	9.777777778	-9.777777778
8	-4	-53.33333333	-39.11111111	39.11111111
8	-3	-40.0	-29.33333333	29.33333333
8	4	53.33333333	39.11111111	-39.11111111
8	5	66.66666667	48.88888889	-48.88888889
8	6	80.0	58.66666667	-58.66666667
8	7	93.33333333	68.44444444	-68.44444444
8	-8	-106.6666667	-78.22222222	78.22222222
5	-1	-8.333333333	-6.111111111	6.111111111
-1	6	-10.0	-7.333333333	7.333333333
-1	-3	5.0	3.666666667	-3.666666667
-2	10	-33.33333333	-24.44444444	24.44444444
-2	-2	6.666666667	4.888888889	-4.888888889
-2	-3	10.00000000	7.333333333	-7.333333333
-3	-1	5.0	3.666666667	-3.666666667
-3	-2	10.0	7.333333333	-7.333333333
-3	-3	15.0	11.0	-11.0
1	-2	-3.333333333	-2.444444444	2.444444444
-1	1	-1.666666667	-1.222222222	1.222222222
0.2	0.1	0.033333333	0.024444444	-0.024444444
10.3	6.1	104.7166667	76.79222222	-76.79222222
5.2	8.6	74.53333333	54.65777778	-54.65777778
9.7	-6.9	-111.55	-81.80333333	81.80333333

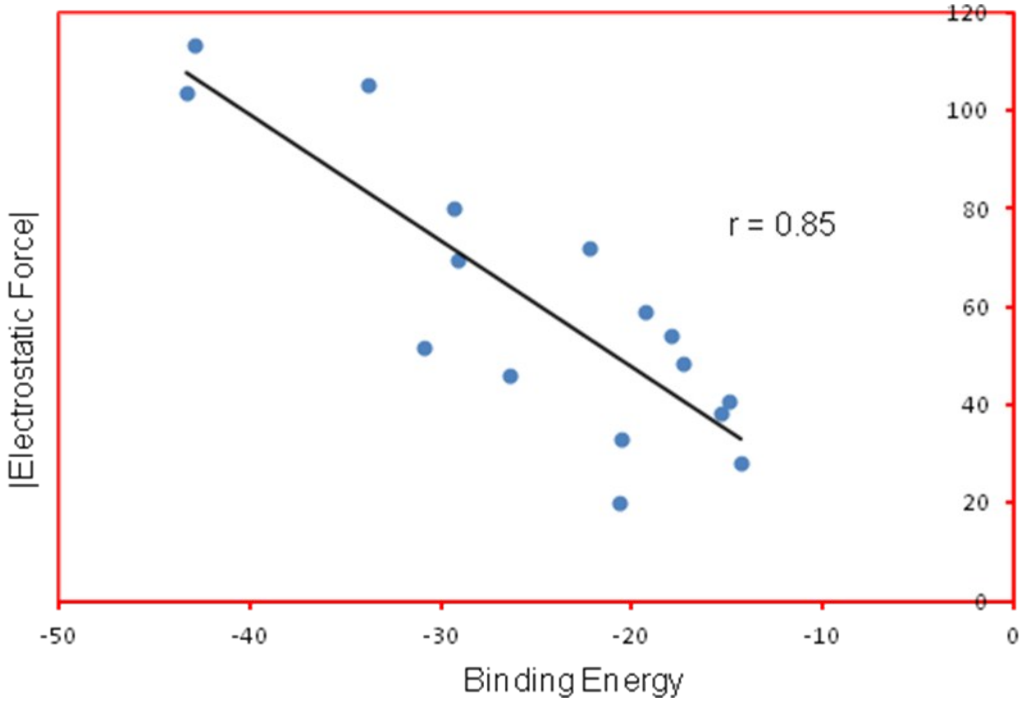

Figure S7. The EF vs. E_b Pearson Correlation graph for planar hydrogen bonded molecules for forces calculated along a line perpendicular to the line of direction of hydrogen bonds, by employing Mulliken charges for the geometries obtained at the COSMO($CHCl_3$)/PBE/TZVP level of theory using Turbomole 6.4 package.

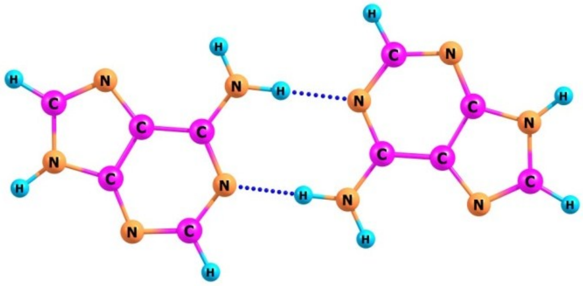

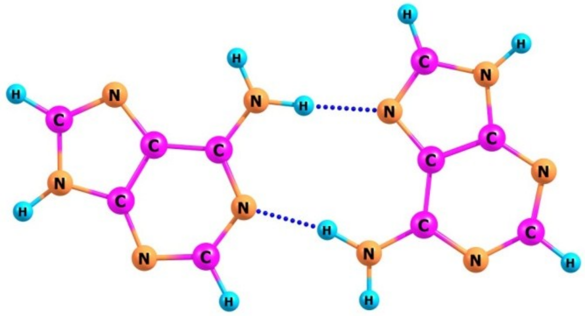

AA1 **AA2**

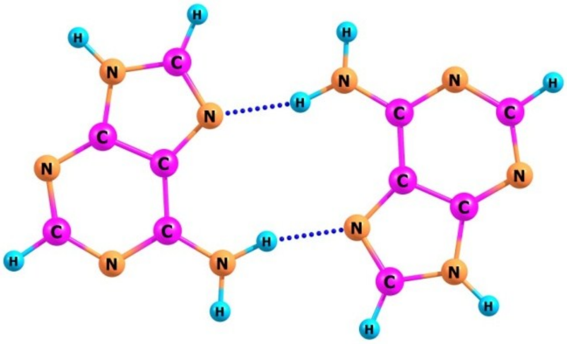

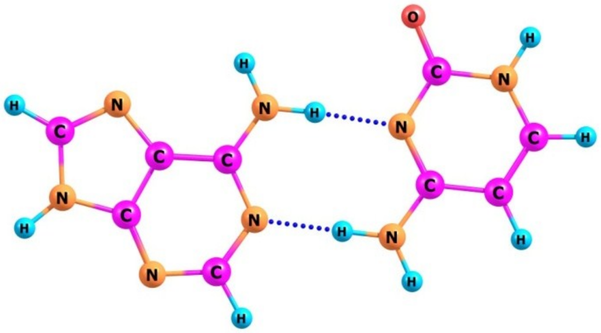

AA3 **AC1**

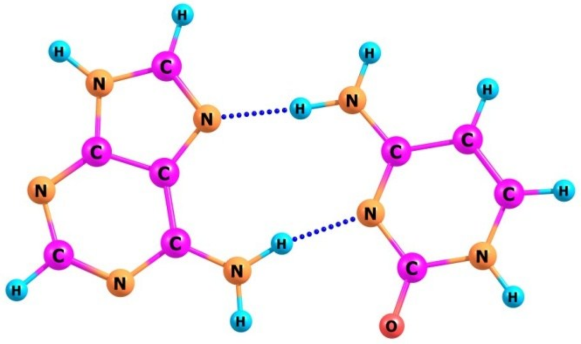

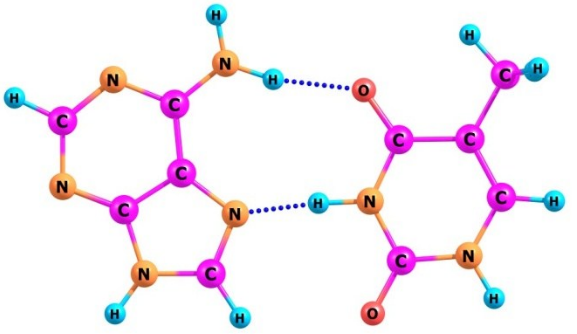

AC2 **AT (H)**

(Figure Continued)

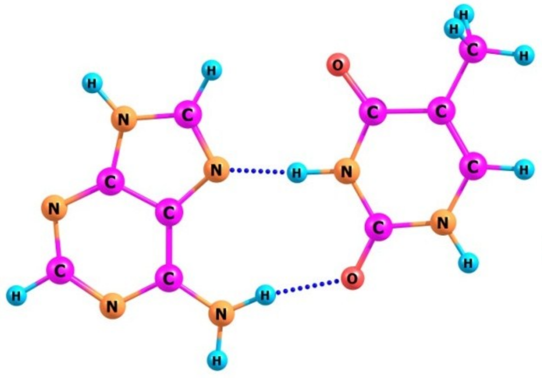

AT (RH)

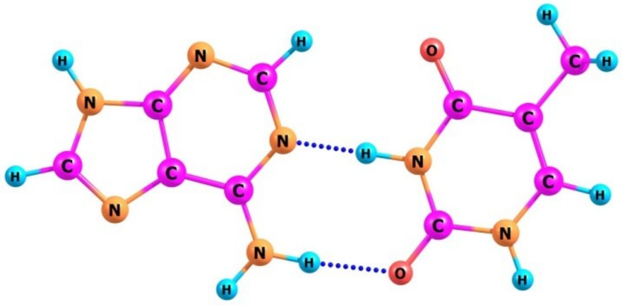

AT (RWC)

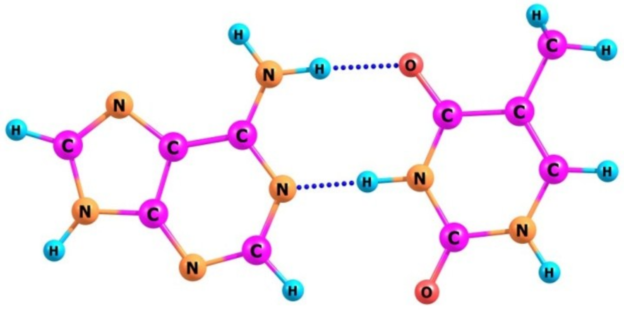

AT (WC)

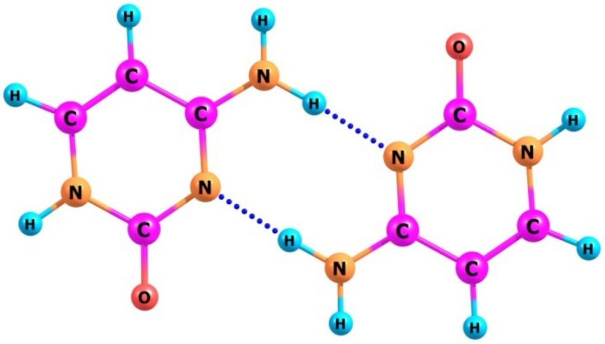

CC

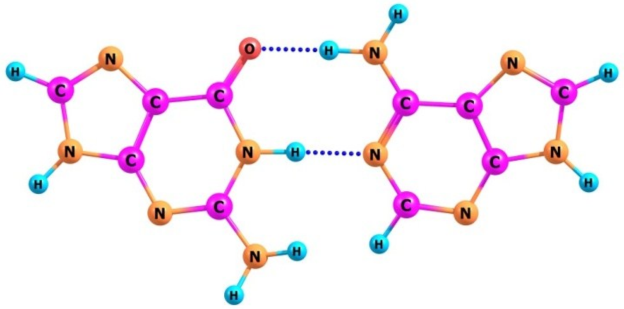

GA1

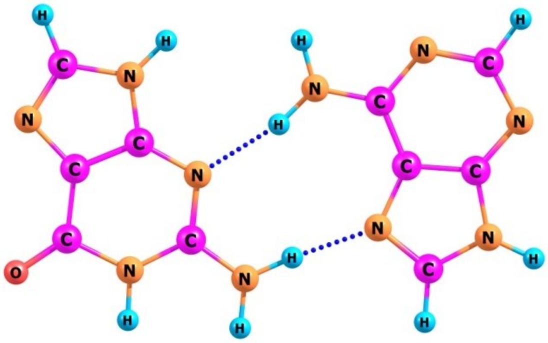

GA2

(Figure Continued)

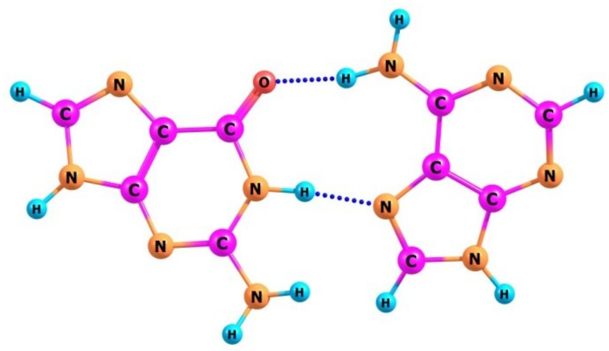

GA3

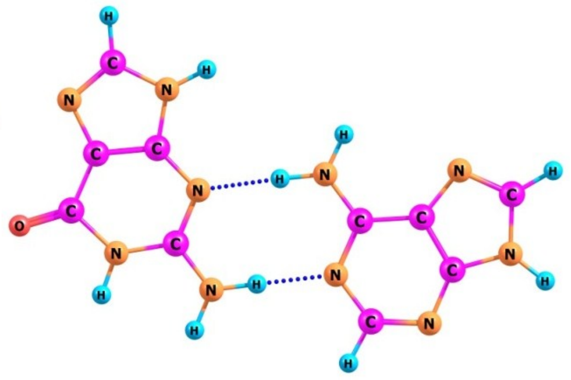

GA4

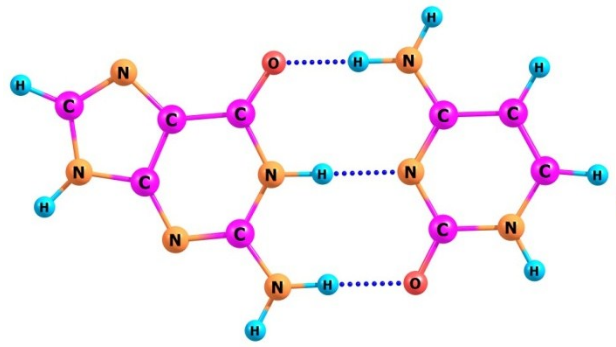

GC

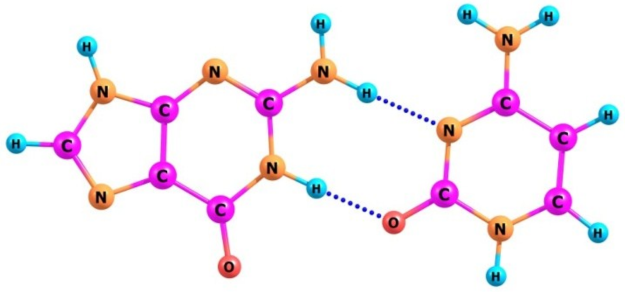

GC1

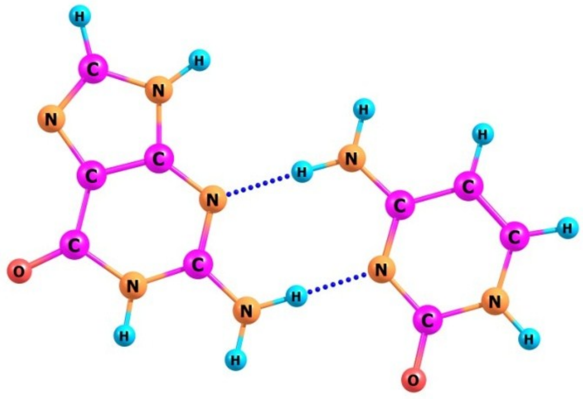

GC2

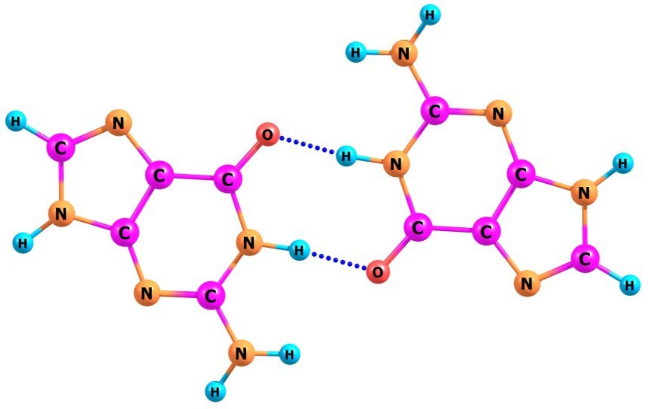

GG1

(Figure Continued)

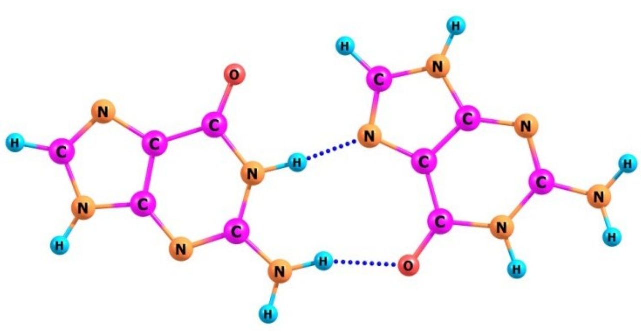

GG3

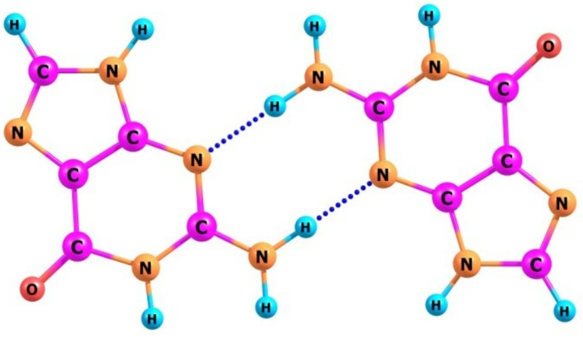

GG4

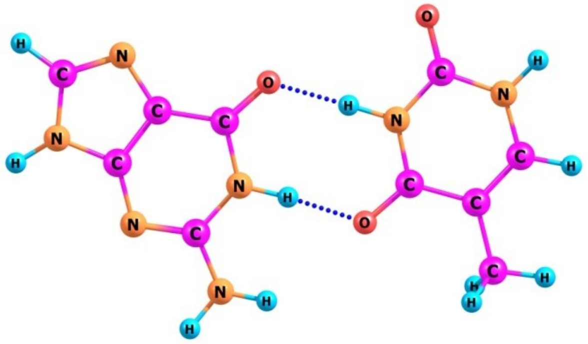

GT1

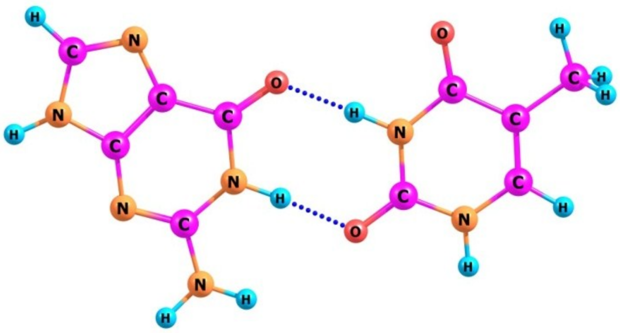

GT2

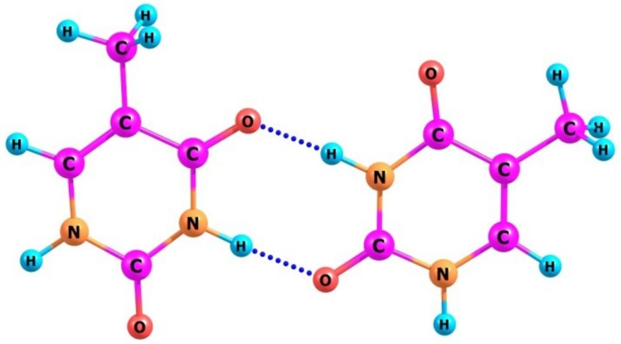

TT1

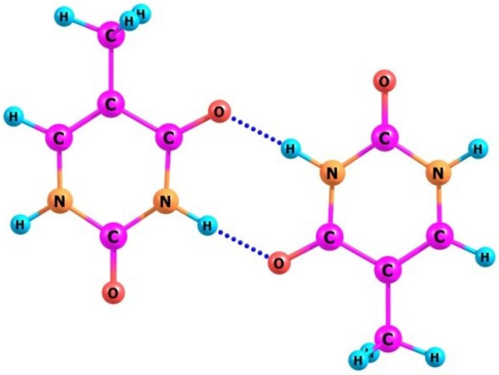

TT2

(Figure Continued)

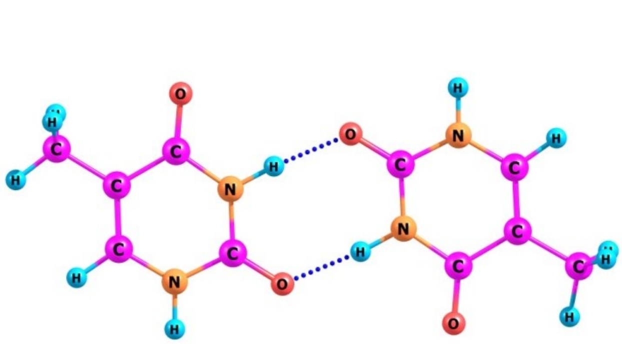

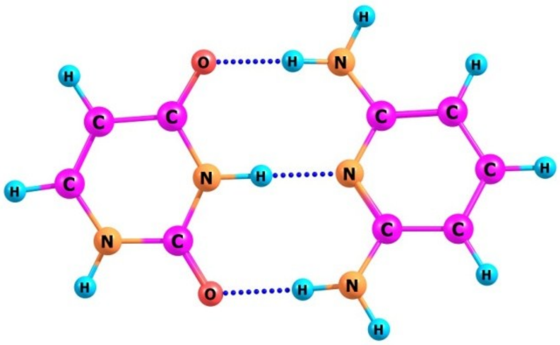

TT3 **U-DAP**

Figure S8. The optimized geometries of 28 base pairs considered by Popelier *et al.* in their QTAIM studies. The same convention of nomenclature of base pairs is followed. Dotted blue lines represent hydrogen bonds.

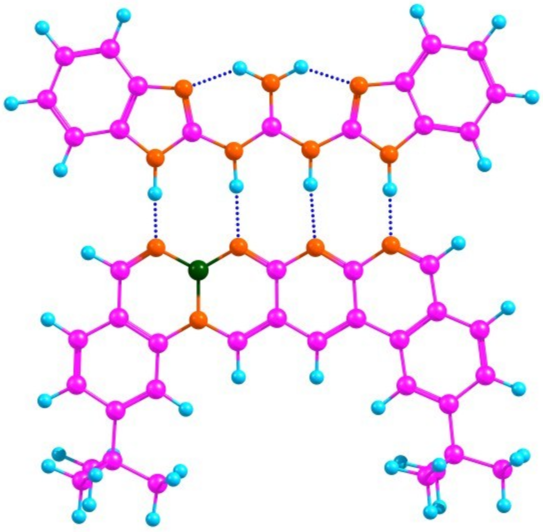

A-1

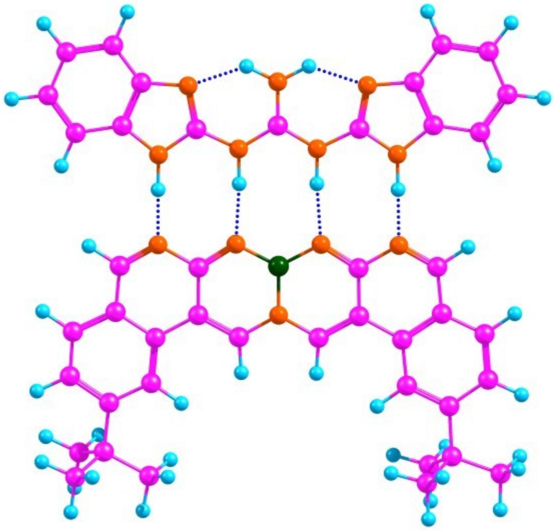

A-2

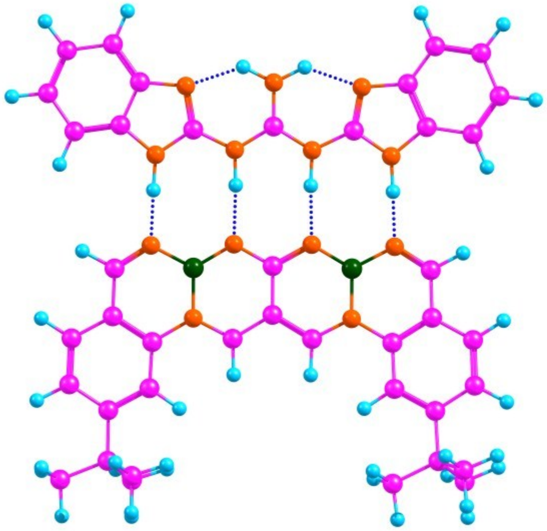

A-3

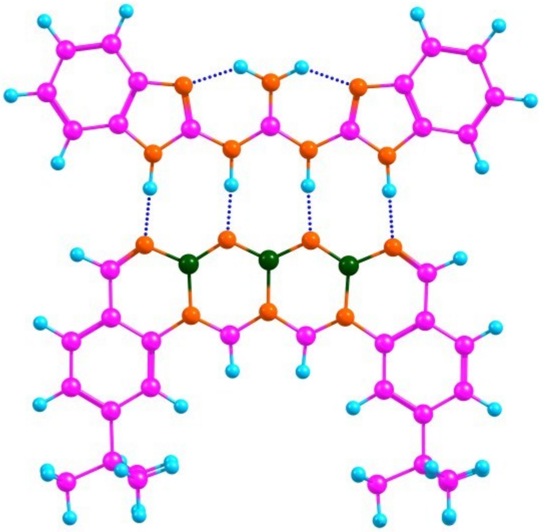

A-4

Figure S9. The optimized geometries of newly designed cationic AAAA-DDDD hydrogen bonded complexes, where C-C bond on the middle region of acceptor partner is replaced with the isoelectronic B-N bonds. Pink, cyan, brown and green colors represent carbon, hydrogen, nitrogen and boron atoms respectively, whereas, dotted blue lines represent hydrogen bonds.

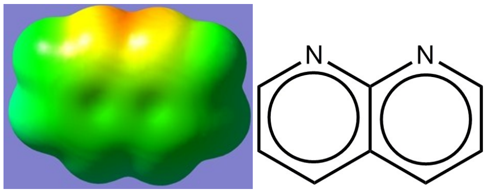

(a)

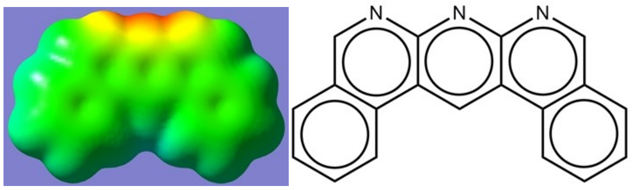

(b)

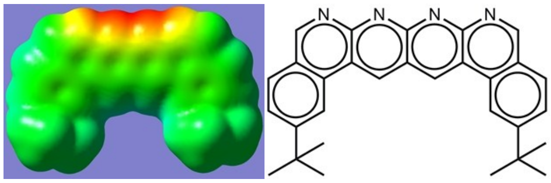

(c)

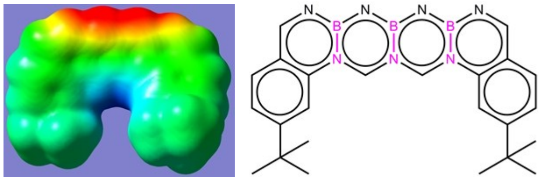

(d)

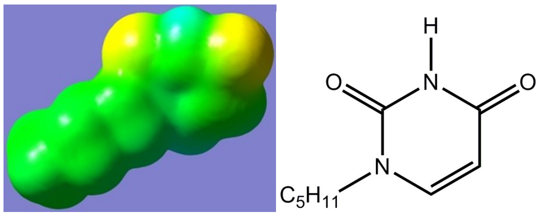

(e)

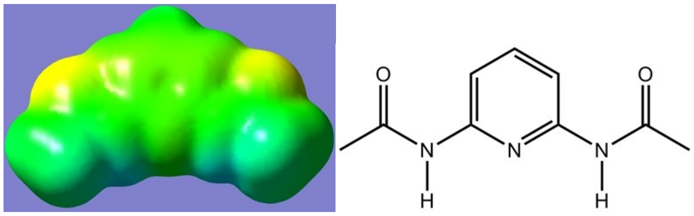

(f)

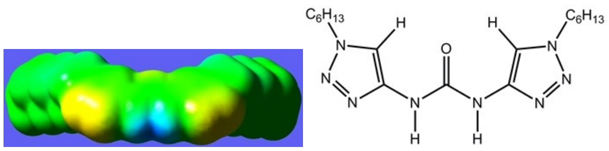

(g)

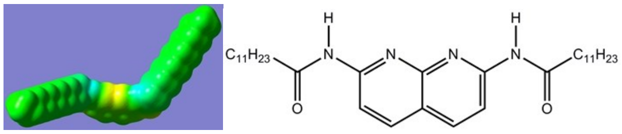

(h)

(Figure continued)

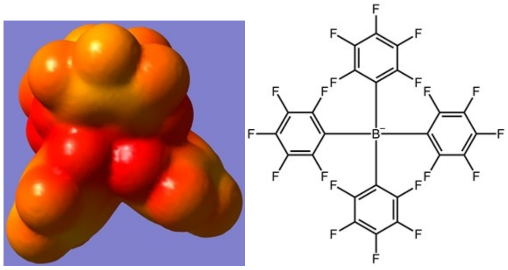

(i)

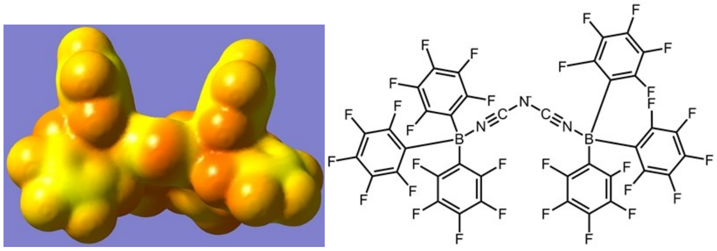

(j)

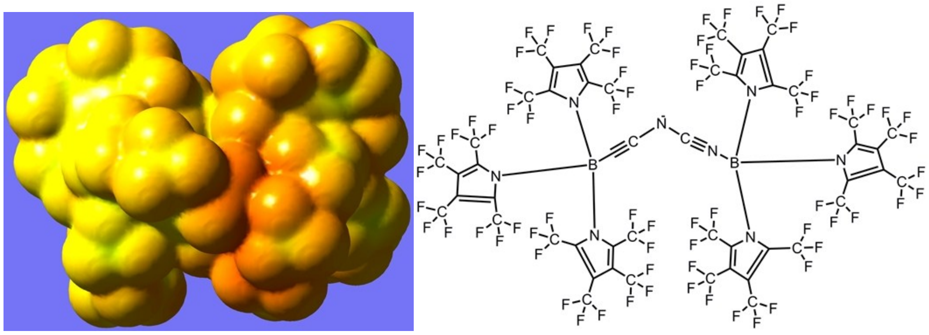

(k)

Figure S10. The Molecular Surface electrostatic potential in Hartrees, computed on the 0.0004 au contour of the electron density, using the GaussView software at the CPCM($CHCl_3$)/M062X/6-31G**of theory. The blue color indicates positive electrostatic potential and the red color indicates negative electrostatic potential, whereas, the intermediate colors indicate intermediate electrostatic potential. The color scale was kept uniform in all the cases.

The Molecular surface electrostatic potential map was constructed for a set of representative noncovalent bonding partners containing nitrogen and fluorine on their interactive sites (Figure S10 above), in order to examine whether they possess σ-holes on their potential surface. The obtained electrostatic potential surfaces reveal that none of the moieties considered in this study possess σ-holes on their surface, suggesting that additional treatment of putting extra point charges on the σ-holes positions is not required for an accurate treatment of electrostatic properties in these molecules, as have been proposed in recent studies (see references 63 and 64 in the main manuscript).

A perusal of Figure S10 (above) suggests that the red region of negative potential becomes intensified from (a) to (c), which indicates that the electrostatic attraction for a specific hydrogen bond donor would become increasingly stronger from (a) to (c), which is what has been reported experimentally by Leigh *et al.* (references 23, 25, 26 in the main manuscript). A

comparison of electrostatic potential surfaces of (c) and (d) further indicates an increase in the negative potential on the binding sites of (d), the newly designed acceptor moiety. This suggests that the binding strength would get stronger for the same donor in (d) in comparison to (c), as obtained in our analysis.

A closer inspection of (i), (j) and (k) in Figure S10 shows increased deterioration of negative potential on the surfaces of these anions on going from (i) to (k), which indicates increasing weaker electrostatic attraction with the cationic zirconocene species. This is reflected in the binding energies of anions with the zirconocene cation. This is what has been observed experimentally by Bochmann et al. (reference 36 in the main manuscript) for (j), and has been obtained by us based on our analysis for (k).

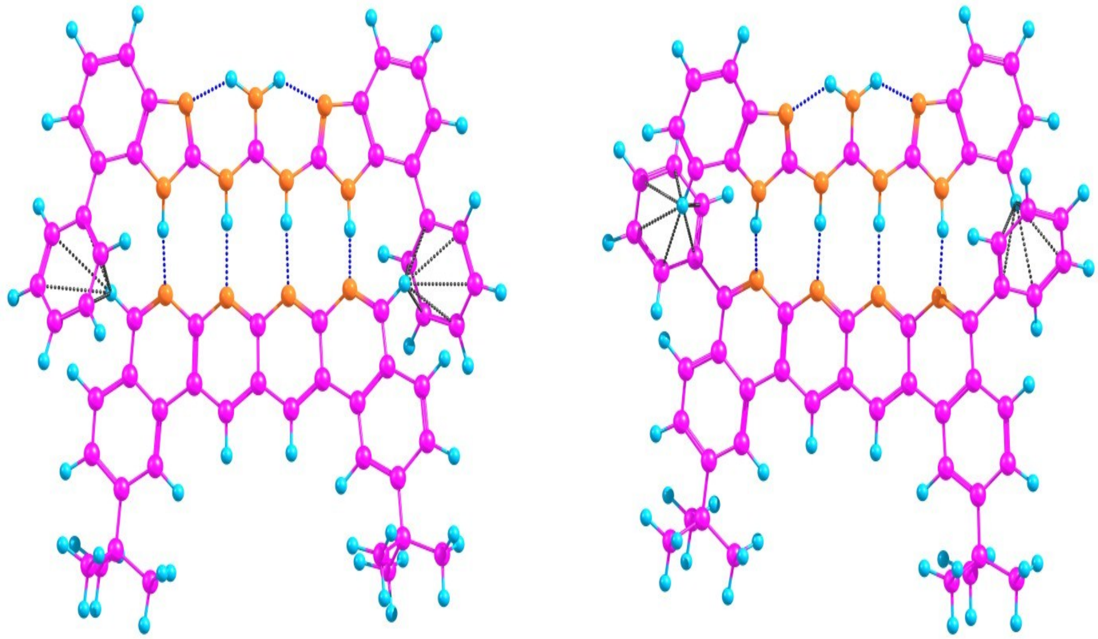

B-1 **B-2**

Figure S11. The optimized geometries of newly designed cationic AAAA-DDDD hydrogen bonded complexes, where hydrogen atoms in frontier lines are replaced with the phenyl groups. Pink, cyan, brown and green colors represent carbon, hydrogen, nitrogen and boron atoms respectively, whereas, dotted blue and black lines represent hydrogen and CH-π bonds respectively.

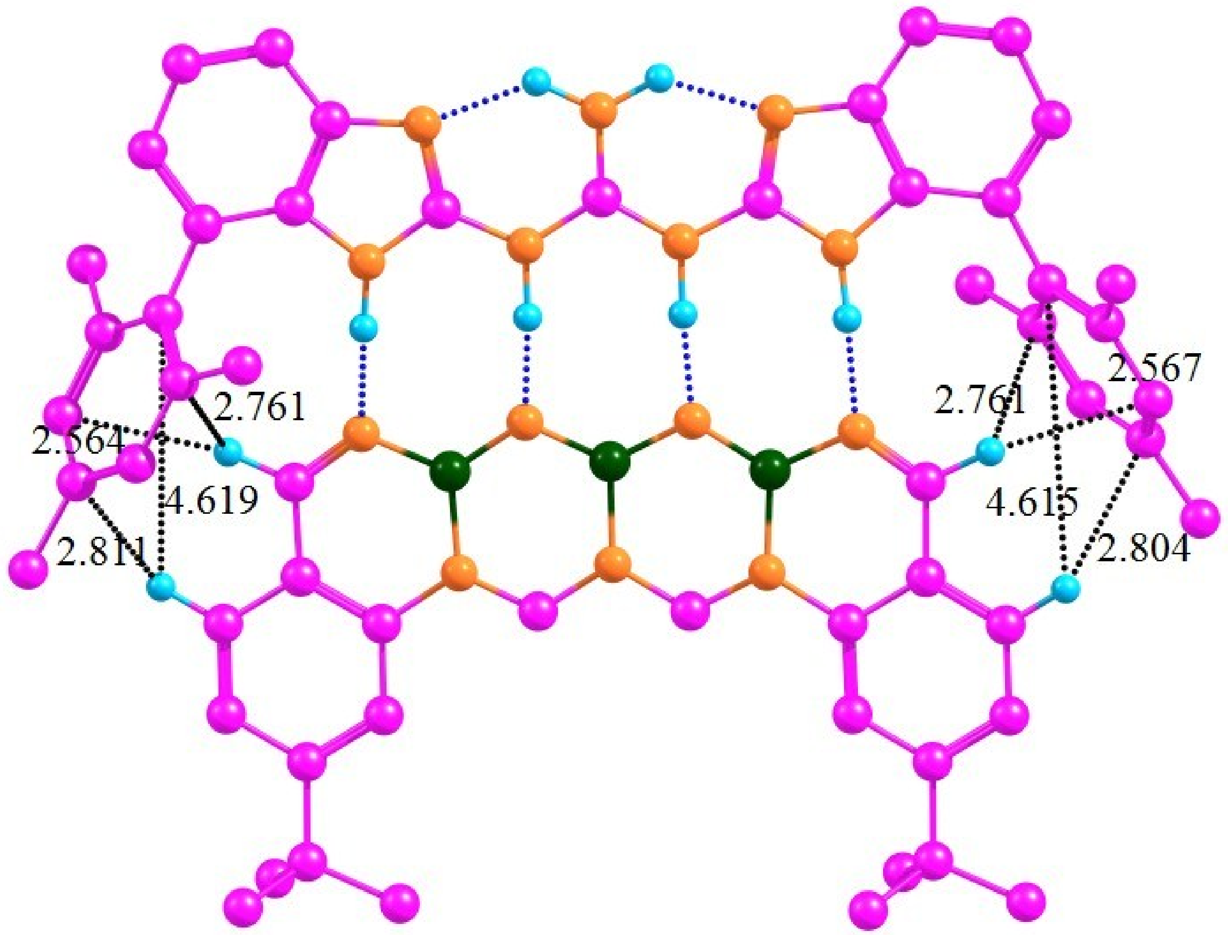

C

Figure S12. The optimized geometry of hypothetical best case designed cationic AAAA-DDDD hydrogen bonded complex, where hydrogen atoms in frontier lines are replaced with the 2,4,6-trimethylphenyl groups and three C-C bonds in the middle region of acceptor partner is replaced with the isoelectronic B-N bonds. Pink, cyan, brown and green colors represent carbon, hydrogen, nitrogen and boron atoms respectively, whereas, dotted blue and black lines represent hydrogen bond and CH-π interactions respectively. Hydrogen atoms other than those involved in hydrogen bonding and CH-π interactions have been deleted for clarity. The distances shown are the maximum and minimum CH-π distances for each hydrogens.

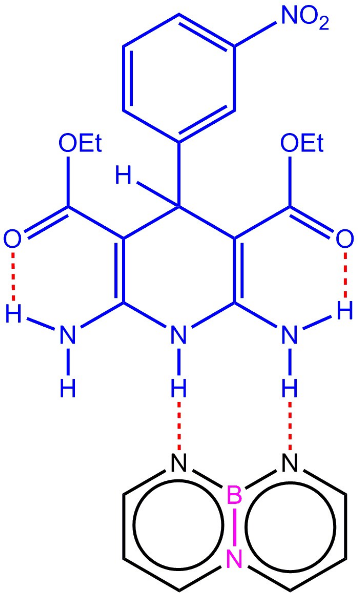

D-1

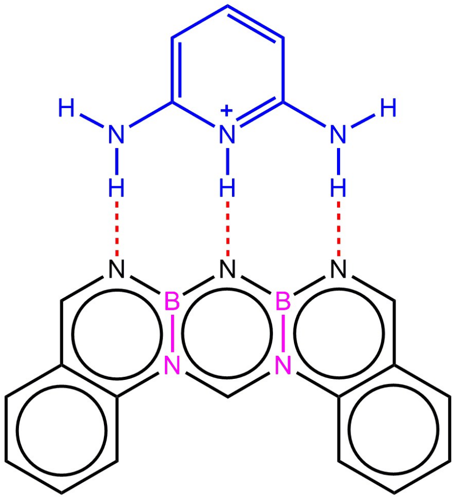

D-2

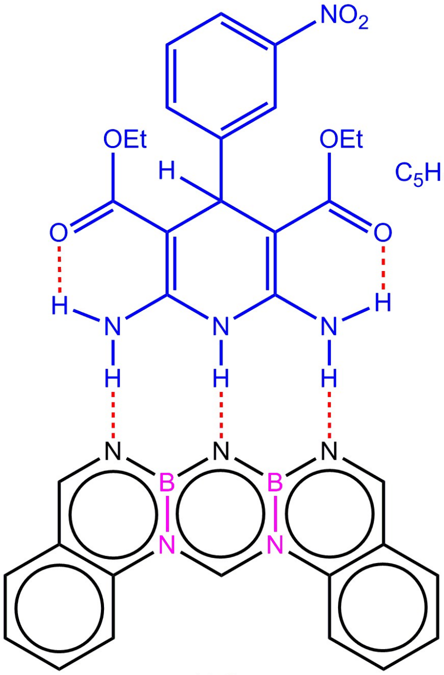

D-3

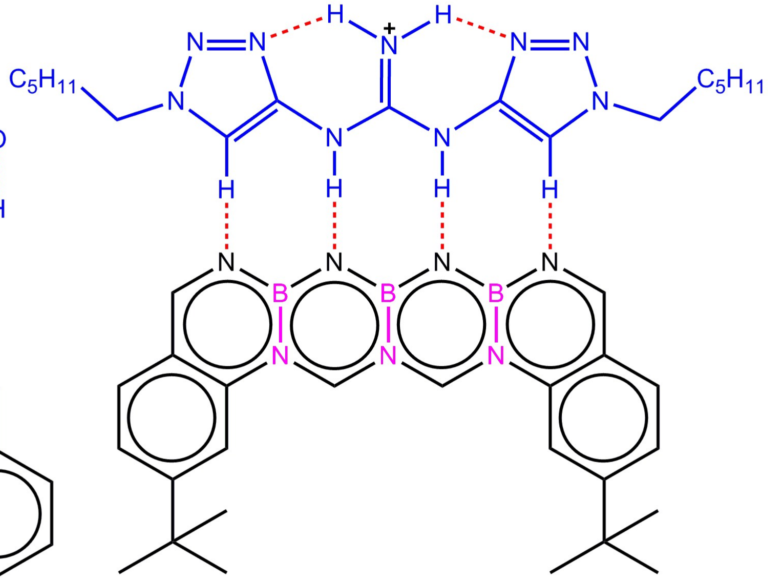

D-4

Figure S13. A schematic picture of some newly designed acceptor-donor planer hydrogen bonded complexes based on our electrostatic force analysis, where central C-C bonds on acceptor moieties are replaced by B-N bonds. These complexes have shown improved binding over their parental complexes from where they are derived. Coordinates of optimized geometries are provided in the corresponding section below.

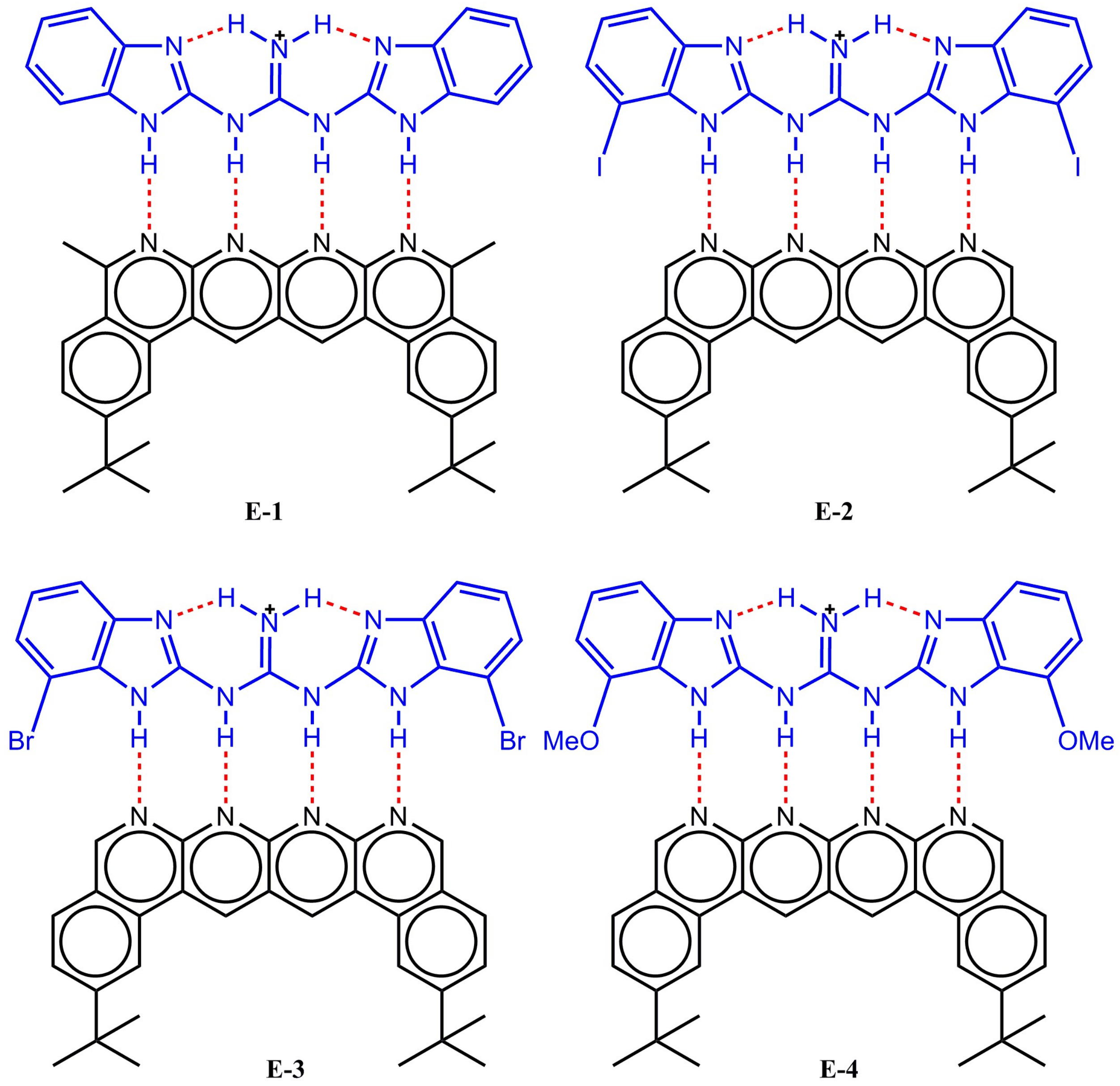

Figure S14. A schematic picture of some newly designed acceptor-donor planer hydrogen bonded complexes, where attractive non-directional dispersive force was exploited for improved binding. These complexes have shown improved binding over their parental complexes from where they are derived. Coordinates of optimized geometries are provided in the corresponding section below.

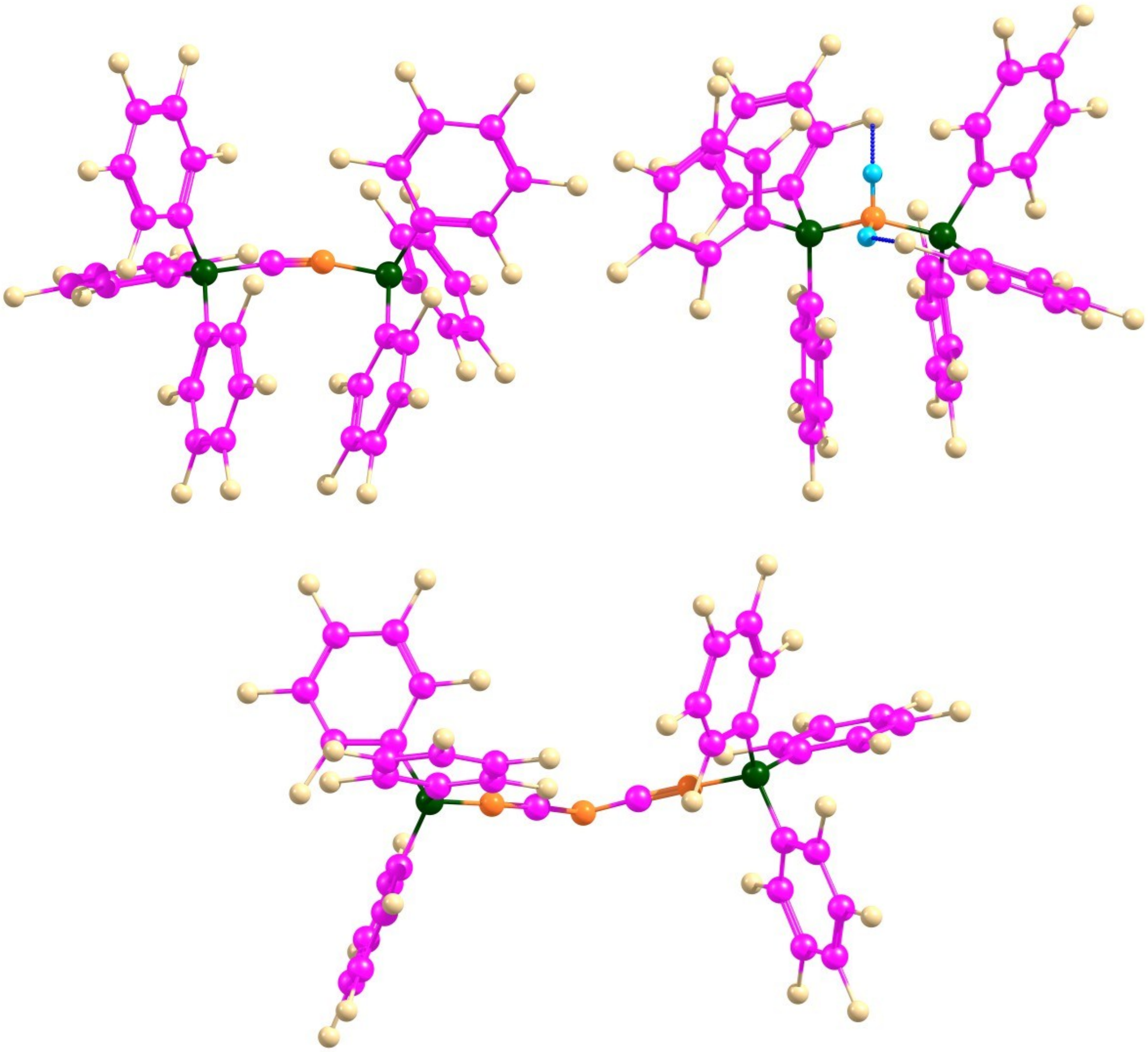

Figure S15. The optimized geometries of Bochmann's anions at the COSMO($CHCl_3$)/PBE/TZVP level of theory using Turbomole 6.4. Pink, cyan, brown, green and white colors represent carbon, hydrogen, nitrogen, boron and fluorine atoms respectively, whereas, dotted blue lines represent hydrogen bonds.

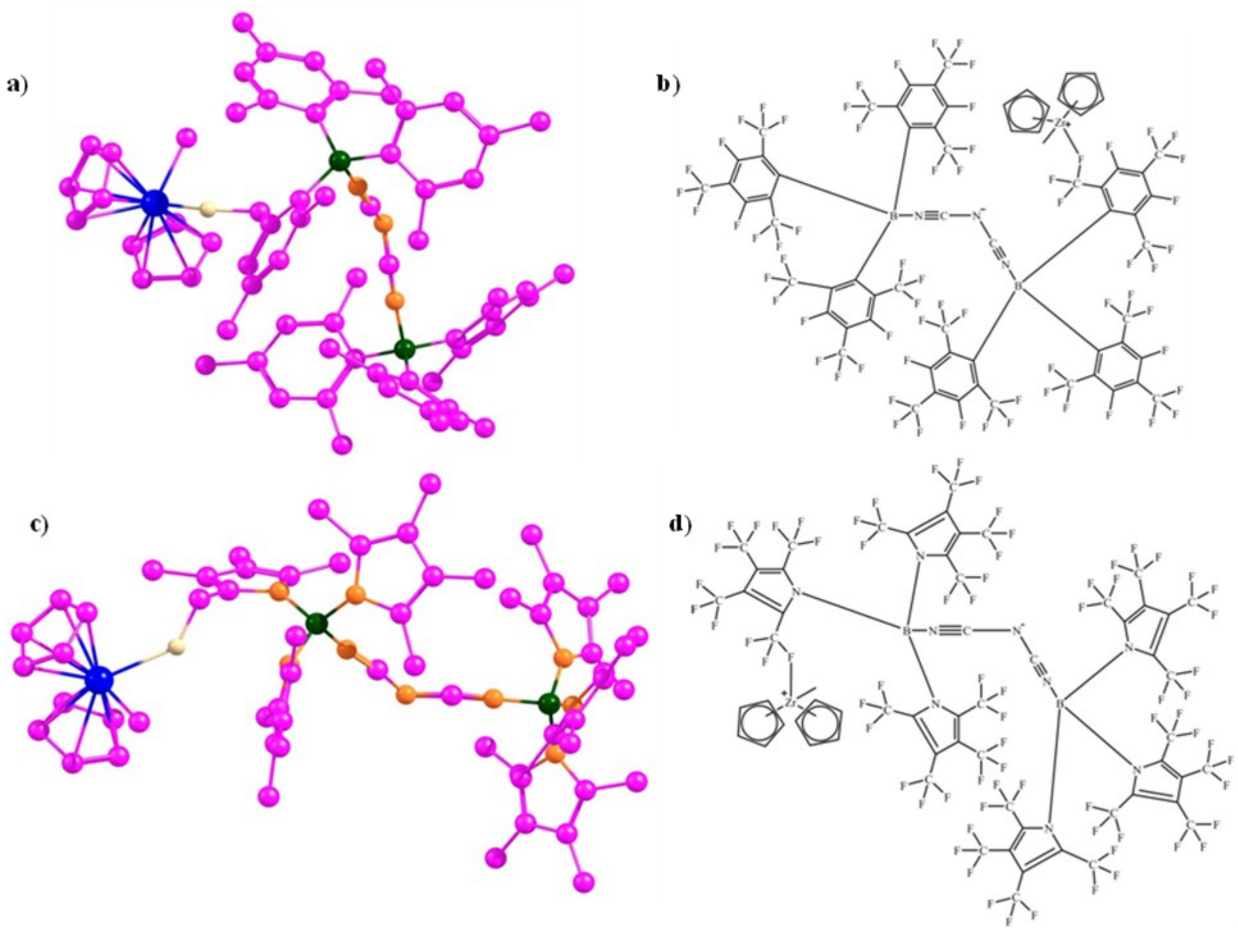

Figure S16. The optimized geometry of the zirconocene complex with newly designed anions. a) anion where *ortho* and *para* fluoro subtstituents are substituted with trifluoro methyl group; b) a schematic picture of a; c) zirconocene complex of best designed anion; d) a schematic picture of c. Pink, white, brown and green colors represent carbon, fluorine, nitrogen and boron atoms respectively. All the hydrogens of the zirconocene and the fluorides of the anions other than the one involved in coordination with the cation have been deleted for clarity.

XYZ coordinates of all the stationary points obtained after full optimization in solvent chloroform.

Turbomole 6.4 geometries

AA-DD (X-1)

C	-3.044305	1.949485	14.204892
C	-1.857839	2.355735	14.815898
C	-0.635427	2.038292	14.204340
C	-0.592268	1.326720	13.000630
C	-1.775243	0.915635	12.383558
C	-2.986178	1.236195	13.004688
C	-1.886314	3.118603	16.138312
C	-1.213443	4.473520	15.990874
C	0.073590	4.657191	16.473404
N	0.696032	3.673552	17.208644
C	0.037003	2.547351	17.668801
C	-1.261689	2.278313	17.241434
N	0.841297	5.767187	16.240311
C	-1.880116	5.532924	15.274963
O	-3.134177	5.165730	14.869785
C	-3.870330	6.135280	14.081147
C	-5.163370	5.470228	13.653364
C	-1.963467	1.134119	17.755411
O	-3.222109	1.018764	17.220542
C	-3.983312	-0.152865	17.605517
C	-5.297203	-0.098469	16.850435
N	0.755740	1.774211	18.512667
N	-4.248798	0.805524	12.373672
O	-4.190336	0.215439	11.283921
O	-1.421871	6.664352	15.016973
O	-1.536372	0.302613	18.582620
O	-5.311845	1.052196	12.963481
N	3.330998	2.819993	19.602276
C	4.114768	3.586253	18.792399
C	5.514210	3.776358	19.038666
C	6.080892	3.145947	20.175087
C	5.271793	2.379656	20.987209
C	3.901147	2.245166	20.653984
N	3.488049	4.160434	17.725693
C	4.210572	4.901899	16.894615
C	5.596544	5.142858	17.053311
C	6.248511	4.580464	18.131088
H	6.122665	5.764408	16.328361
H	5.664346	1.875145	21.870527
H	3.247873	1.632711	21.282468
H	3.671935	5.337842	16.046988
H	1.705762	3.807446	17.449528
H	-2.944719	3.283330	16.382998
H	1.611720	2.148170	18.952840
H	0.245446	0.981208	18.910488
H	-3.403116	-1.054496	17.353050
H	-4.136287	-0.142961	18.696104
H	-5.905429	-0.978938	17.107568
H	-5.865307	0.805314	17.117302
H	-5.126855	-0.096466	15.763607
H	0.314505	6.538311	15.814029
H	1.521595	6.012447	16.955903
H	-4.052097	7.032868	14.693155
H	-3.256343	6.432760	13.216696
H	-5.753865	6.172876	13.046218
H	-4.960501	4.572916	13.050104
H	-5.763224	5.177079	14.527728
H	0.294395	2.355770	14.682899
H	0.367299	1.088373	12.538606
H	-1.770853	0.359253	11.447346
H	-4.004971	2.182993	14.660662
H	7.145024	3.273930	20.386652
H	7.316656	4.739384	18.296912

AAA-DDD, cation, Leigh (X-2)

C	29.643894	11.311791	6.628046
C	30.977919	11.382164	7.081568
C	31.237497	11.156749	8.464138
C	30.179892	10.871996	9.356506
C	28.877654	10.808379	8.886579
C	28.615249	11.029183	7.518391
C	32.591569	11.226685	8.923364
N	33.629354	11.480570	8.164057
C	33.426952	11.702660	6.816850
C	32.116170	11.670752	6.229730
C	32.019512	11.924195	4.859226
C	33.161926	12.192317	4.100914
C	34.425329	12.186948	4.785252
N	34.541343	11.948726	6.104307
N	35.614177	12.429227	4.126592
C	35.581232	12.682542	2.840848
C	34.392526	12.726181	2.044077
C	33.142022	12.470761	2.677119
C	31.972064	12.500488	1.889332
C	32.049965	12.772626	0.528701
C	33.290810	13.025343	-0.092676
C	34.454236	13.003633	0.660136
N	38.169579	11.941342	5.276707
C	38.368453	11.838814	6.601957
N	37.252305	11.807717	7.407372
C	37.316803	11.689711	8.777589
C	38.578433	11.592203	9.387541
C	39.717093	11.622945	8.585674
C	39.635611	11.745214	7.200665
N	36.153866	11.695158	9.452045
H	32.789043	11.057310	9.988342
H	30.404948	10.704008	10.412250
H	28.055546	10.587997	9.569226
H	27.588380	10.976647	7.152387
H	29.406957	11.477241	5.576751
H	31.042533	11.912672	4.376138
H	30.996863	12.310360	2.338525
H	31.136147	12.790192	-0.067867
H	33.330674	13.234989	-1.162560
H	35.425648	13.195895	0.199177
H	36.543504	12.867701	2.349583
H	36.308235	11.873098	6.955391
H	35.231980	11.629291	8.960508
H	36.190290	11.539288	10.453581
H	38.637762	11.495630	10.470511
H	40.699733	11.549701	9.055687
H	40.522881	11.772074	6.570187
H	37.223284	12.132489	4.874278
H	38.986432	12.026306	4.681746

AAA-DDD, neutral, Leigh (X-3)

C	6.272532	4.942383	18.665328
C	5.623274	3.806798	19.156864
C	4.246636	3.620761	18.787377
N	3.575941	4.483753	18.005718
C	4.217618	5.572464	17.549275
C	5.594845	5.853516	17.851068
C	6.252892	2.816537	20.009648
C	5.456174	1.711835	20.425416
C	4.093975	1.646079	19.982421
N	3.512568	2.532715	19.215301
C	6.195865	7.053050	17.298282
C	5.999779	0.708207	21.257100
H	3.482809	0.794707	20.304794
N	3.453160	6.410918	16.762768
C	7.592755	2.881442	20.447682
H	7.317817	5.118956	18.919342
C	4.007657	7.490436	16.272782
H	3.373295	8.133340	15.651216
C	5.369644	7.883532	16.488420
C	5.884809	9.066085	15.912847
C	7.205326	9.425333	16.133536
H	5.228936	9.685181	15.296300
H	7.610173	10.336905	15.690811
C	8.028339	8.606866	16.935105
C	7.536282	7.441025	17.509423
H	9.067663	8.892698	17.106917
H	8.197231	6.829514	18.124734
C	7.318898	0.793780	21.674149
H	5.366153	-0.127758	21.562082
H	7.745114	0.021937	22.316992
C	8.112591	1.885566	21.265218
H	8.231623	3.713491	20.149686
H	9.150873	1.950218	21.595986
N	0.725496	2.087924	18.546716
C	0.020579	2.764289	17.619631
N	0.677023	3.837253	17.034185
C	0.026622	4.760917	16.228804
C	-1.282998	4.524833	15.806304
C	-1.949196	3.192369	16.095972
C	-1.286913	2.451053	17.242301
C	-1.956946	2.323375	14.839370
C	-3.158733	1.962461	14.230069
C	-3.132647	1.164860	13.082800
C	-1.937906	0.708642	12.517052
C	-0.739265	1.075231	13.131722
C	-0.751148	1.874400	14.280216
N	-4.410306	0.793835	12.447043
O	-5.461138	1.223005	12.948546
C	-1.953696	5.488207	14.979608
O	-1.499783	6.579089	14.571400
N	0.743539	5.852675	15.902710
C	-1.952734	1.324815	17.833896
O	-1.497825	0.549105	18.701980
O	-3.222035	5.083209	14.642293
C	-3.954684	5.939438	13.733535
C	-5.265496	5.242446	13.425360
O	-3.216061	1.149224	17.324346
C	-3.937537	-0.022237	17.774807
C	-5.254822	-0.050193	17.023963
O	-4.379256	0.071057	11.438422
H	1.631061	4.063422	17.363565
H	-3.001268	3.375479	16.356152
H	1.712104	2.314585	18.766362
H	0.249040	1.270759	18.939291
H	-3.331322	-0.918450	17.568318
H	-4.088446	0.039959	18.864167
H	-5.832818	-0.936271	17.327739
H	-5.851485	0.847183	17.244930
H	-5.085882	-0.098509	15.937933
H	0.251988	6.532218	15.314582
H	-4.114075	6.920970	14.208232
H	-3.352539	6.097994	12.824944
H	-5.855091	5.858883	12.729903
H	-5.089259	4.261109	12.959760
H	-5.854967	5.092145	14.342228
H	0.190479	2.155313	14.758270
H	0.208092	0.733457	12.711088
H	-1.958259	0.087572	11.622666
H	-4.106860	2.299111	14.645981
H	1.693636	6.023271	16.278912

AAA-DDD, OH, Leigh (**X-4**)

N	1.076790	0.696409	18.874651
C	1.741923	-0.049032	19.829593
C	1.100787	-1.094483	20.575574
C	-0.291394	-1.388634	20.296532

C	-0.937504	-0.600485	19.301001
C	-0.184179	0.428025	18.643476
N	3.030431	0.276807	20.009684
C	3.766912	-0.384913	20.919286
C	3.211735	-1.428285	21.736931
C	1.869368	-1.761533	21.534645
C	4.070948	-2.070129	22.713463
C	5.422807	-1.627134	22.780525
C	5.848907	-0.584863	21.889269
N	5.086967	0.011554	21.007686
C	3.653337	-3.096046	23.587335
C	4.548511	-3.654406	24.491251
C	5.886058	-3.212014	24.554328
C	6.319415	-2.205404	23.705130
C	-2.292760	-0.829658	18.975301
C	-3.001549	-1.830080	19.622349
C	-2.365299	-2.615210	20.606604
C	-1.033825	-2.401009	20.940739
H	6.893660	-0.254166	21.933279
H	1.416270	-2.554647	22.129679
H	-0.687230	1.041446	17.887354
H	-2.766226	-0.211088	18.209481
H	-4.048058	-2.013011	19.373404
H	-2.925530	-3.403428	21.113060
H	-0.568182	-3.025050	21.704243
H	7.351363	-1.848514	23.737160
H	6.574764	-3.662499	25.270977
H	2.624509	-3.457742	23.563443
H	4.211036	-4.446840	25.162055
C	5.077167	2.913666	18.583593
C	6.455819	3.208642	18.533564
C	6.881545	4.532809	18.673280
C	5.970790	5.581598	18.873179
C	4.611308	5.252071	18.962239
C	4.140641	3.937068	18.832414
C	7.427027	2.059052	18.423513
O	7.408919	1.211817	19.591921
C	6.436705	7.012774	18.974184
C	2.666038	3.641178	18.980873
O	2.079168	3.085289	17.794348
O	4.732707	1.608839	18.376248
H	3.879463	6.045759	19.145598
H	7.955069	4.745459	18.641330
H	1.777647	2.172729	18.047843
H	3.964471	1.300634	18.948880
H	6.475766	0.926310	19.730795
H	7.386619	7.085737	19.526174
H	6.605476	7.448660	17.974458
H	5.688835	7.637747	19.484896
H	2.147164	4.582371	19.236495
H	2.512807	2.942349	19.824951
H	8.453907	2.443763	18.338951
H	7.203149	1.458762	17.522479

AAA-DDD, Cation, model (**X-5**)

C	6.192566	4.945133	18.646510
C	5.550390	3.813042	19.133199
C	4.201007	3.564079	18.805824
C	3.523080	4.496860	17.968811
C	4.190076	5.643533	17.483201
C	5.515784	5.866216	17.820083
C	2.154679	4.243213	17.633035
N	1.464367	3.207576	18.042439
C	2.076627	2.280197	18.859318
C	3.447515	2.413666	19.267596
N	1.295067	1.253172	19.238073
C	1.821706	0.305028	20.033542
C	3.180651	0.337719	20.497923
C	3.971613	1.416128	20.093780
C	3.649635	-0.738607	21.350662
C	2.719284	-1.767673	21.676722
C	1.391422	-1.679056	21.149014
N	0.957128	-0.713287	20.377039
C	3.102234	-2.848102	22.502432
C	4.393557	-2.912031	23.001687
C	5.319185	-1.896749	22.681514
C	4.957809	-0.827123	21.871082
H	1.634152	4.959619	16.986955
H	5.007551	1.479832	20.426826
H	0.675590	-2.469658	21.401359
H	2.369136	-3.622976	22.737224
H	4.697014	-3.742888	23.640233
H	6.335384	-1.951225	23.076187
H	5.696942	-0.058910	21.642798
H	3.647019	6.343703	16.844579
H	6.037533	6.749771	17.449188
H	6.102155	3.120858	19.769881
H	7.237002	5.123944	18.908540
N	-1.760436	-0.783881	19.644738
C	-2.397771	0.103093	18.889167
N	-1.637997	1.142600	18.416442
C	-2.117159	2.142700	17.609178
C	-3.495600	2.147399	17.232209
C	-4.338227	1.077813	17.721300
C	-3.785349	0.032916	18.555437
N	-1.230207	3.056212	17.230332
C	-4.539248	-1.106956	19.096135
O	-5.860718	-1.136703	18.764777
C	-6.642942	-2.262108	19.281179
C	-8.069633	-2.059198	18.819802
N	-5.636869	1.052733	17.396562
C	-3.944225	3.255042	16.376113
O	-5.271743	3.245254	16.066219
C	-5.756690	4.329250	15.208998
C	-7.249104	4.135183	15.049721
O	-4.062615	-2.003167	19.805159
O	-3.217216	4.157769	15.941730
H	-0.626355	1.169964	18.682203
H	-0.232050	3.041868	17.556338
H	-1.598180	3.802163	16.629265
H	-5.510105	5.285980	15.690620
H	-5.222620	4.272314	14.249870
H	-7.644573	4.931058	14.401450
H	-7.476503	3.166371	14.580861
H	-7.762127	4.195323	16.020764
H	-2.337511	-1.557457	19.993794
H	-6.205212	-3.191462	18.889788
H	-6.556041	-2.267956	20.377005
H	-8.685853	-2.891451	19.191076
H	-8.483759	-1.120570	19.216680
H	-8.134612	-2.046398	17.721717
H	-0.738024	-0.701984	19.870303
H	-6.012415	1.792478	16.805823
H	-6.218265	0.291467	17.742492

AAA-DDD, anion, model (**X-7**)

C	6.230690	4.991475	18.625927
C	5.568026	3.843772	19.114165
C	4.203166	3.644893	18.754861
C	3.538550	4.579610	17.928755
C	4.211255	5.697977	17.462559
C	5.562632	5.900242	17.815499
C	3.528744	2.481117	19.249541
N	4.081388	1.574429	20.018925
C	5.398021	1.725834	20.390281
C	6.191562	2.845943	19.964210
C	7.518431	2.906573	20.394442
C	8.051810	1.902296	21.216092
C	7.162180	0.825832	21.583668
N	5.886375	0.747787	21.181421
N	7.583576	-0.216309	22.393719
C	8.815919	-0.227088	22.831709
C	9.803130	0.780013	22.537334
C	9.398073	1.871498	21.706272
C	11.100636	0.716503	23.027793
C	12.062722	1.746331	22.709575
N	11.636044	2.812615	21.889331
C	10.393936	2.844502	21.438117
H	9.105077	-1.072126	23.468975
H	8.152089	3.740106	20.091105
H	2.479148	2.325845	18.973307
H	2.492079	4.403499	17.667045
H	3.700774	6.421623	16.824774
H	6.090761	6.782066	17.447257
H	7.275021	5.173484	18.882160
H	11.413789	-0.115554	23.662864
O	13.247806	1.727808	23.130050
H	10.141342	3.706138	20.806768
N	5.924945	-2.399750	23.170586
C	4.673764	-2.660874	22.770686
N	4.116842	-1.702762	21.958464
C	2.833594	-1.763159	21.459763
C	2.001953	-2.870468	21.757667
C	2.527925	-3.972736	22.608772
C	3.925179	-3.814015	23.123141
C	0.656133	-2.839609	21.182304
O	-0.142267	-3.899270	21.461018
C	-1.471274	-3.852111	20.883458
C	-2.174506	-5.130162	21.298452
N	2.483102	-0.712796	20.703102
C	4.611114	-4.780090	23.970859
O	3.898405	-5.911194	24.277827
C	4.567747	-6.886783	25.122001
C	3.607086	-8.044214	25.307826
N	1.763162	-4.998514	22.863624
O	5.767620	-4.666750	24.427322
O	0.208383	-1.913719	20.464563
H	4.696181	-0.876066	21.714066
H	6.457239	-1.562500	22.846744
H	6.344853	-3.121263	23.766413
H	5.501360	-7.202627	24.632498
H	4.828940	-6.410867	26.079517
H	4.076797	-8.807343	25.947105
H	2.677146	-7.709554	25.790815
H	3.356793	-8.506069	24.341218
H	1.525027	-0.759064	20.334605
H	-1.998563	-2.957727	21.251992
H	-1.392023	-3.767212	19.788221
H	-3.193963	-5.139201	20.882966
H	-1.635022	-6.012219	20.923206
H	-2.241029	-5.202246	22.393872
H	3.126399	0.082694	20.513208
H	2.277344	-5.652598	23.457725

AAA-DDD, ion pair, model (**X-8**)

N	4.907276	0.542547	20.933093
C	3.622237	0.157096	20.631208
C	3.064329	-1.079300	21.104640
C	3.898262	-1.936346	21.928631
C	5.222493	-1.493058	22.215736
C	5.654301	-0.238507	21.678972
C	1.751387	-1.382951	20.742155
C	1.005133	-0.503568	19.941480
C	1.665843	0.711305	19.527096
N	2.926868	1.021916	19.860940
C	-0.342399	-0.725136	19.514809
C	-0.979806	0.263485	18.698911
C	-0.213244	1.431399	18.359962
N	1.020147	1.650256	18.740807
C	-2.285040	0.080307	18.264207
C	-3.023029	-1.108978	18.624869
N	-2.371307	-2.066947	19.432336
C	-1.129138	-1.863115	19.830272
C	6.084242	-2.282191	23.011564
C	5.645047	-3.494895	23.518312
C	4.335188	-3.938665	23.237429
C	3.475751	-3.175296	22.457458
H	-0.681077	2.204107	17.737516
H	1.295323	-2.313089	21.080315
H	6.672088	0.105215	21.895366
H	7.094758	-1.920844	23.216971
H	6.307164	-4.107198	24.132721
H	3.991367	-4.894383	23.637551
H	2.469032	-3.543797	22.257486
H	-2.774806	0.832630	17.641446

O	-4.203359	-1.317261	18.252009
H	-0.695995	-2.655774	20.454548
C	6.186965	4.935318	18.651406
C	5.524979	3.768211	19.150767
N	4.213714	3.539047	18.822888
C	3.457362	4.375237	18.041014
C	4.046464	5.562916	17.499969
C	5.431983	5.836871	17.808870
N	6.094681	2.858807	19.931679
N	2.199958	4.002893	17.843607
C	3.184134	6.408147	16.664039
O	1.990913	6.189315	16.418981
N	6.019683	6.935292	17.314341
C	7.590588	5.118674	19.039858
O	8.237221	4.353112	19.767354
O	8.191967	6.236190	18.534592
C	9.590441	6.452211	18.904336
C	10.042844	7.717713	18.207904
O	3.790808	7.510967	16.132717
C	2.958639	8.378187	15.299087
C	3.848011	9.494492	14.795435
H	3.757614	2.668347	19.197336
H	1.805407	3.099051	18.229834
H	1.643041	4.633537	17.257266
H	2.543395	7.775123	14.479256
H	2.127065	8.751964	15.913847
H	3.251183	10.170987	14.165965
H	4.263548	10.078077	15.630328
H	4.674264	9.097314	14.187182
H	7.075265	3.040169	20.170969
H	9.648435	6.535471	19.999202
H	10.171176	5.573438	18.589702
H	11.094101	7.912207	18.467188
H	9.970930	7.614798	17.115053
H	9.443293	8.582475	18.528501
H	5.580192	2.005287	20.284763
H	5.476828	7.559070	16.720169
H	6.995056	7.118132	17.541686

AAA-DDD, neutral, model (**X-6**)

N	4.883885	0.520032	20.939715
C	3.595276	0.125227	20.641281
C	3.046268	-1.113988	21.120104
C	3.878737	-1.970222	21.943677
C	5.200938	-1.521382	22.224133
C	5.624898	-0.262746	21.682293
C	1.731804	-1.417722	20.756902
C	0.993311	-0.540314	19.959015
C	1.639798	0.672145	19.537671
N	2.903013	0.984575	19.874035
C	-0.372803	-0.782461	19.535557
C	-0.989803	0.210123	18.721394
C	-0.232145	1.376922	18.374493
N	1.000122	1.607477	18.749850
C	-2.316885	0.042925	18.268466
C	-3.028853	-1.094344	18.615696
C	-2.423177	-2.080246	19.422165
C	-1.118866	-1.930630	19.876540
C	6.069797	-2.302305	23.017910
C	5.636926	-3.515152	23.531074
C	4.328065	-3.964201	23.257199
C	3.461482	-3.208219	22.477498
H	-0.708971	2.140267	17.748190
H	1.278982	-2.348698	21.097789
H	6.643359	0.080456	21.898796
H	7.079368	-1.935975	23.217361
H	6.303288	-4.122951	24.145190
H	3.990086	-4.919416	23.663210
H	2.455723	-3.582354	22.283454
H	-2.766320	0.819424	17.645248
H	-4.054312	-1.230110	18.268277
H	-0.679454	-2.710837	20.499357
H	-2.987070	-2.974696	19.693704
C	6.236943	5.005787	18.618089
C	5.577217	3.855970	19.109246
N	4.257024	3.620812	18.788456
C	3.491381	4.456362	18.012067
C	4.060670	5.632242	17.463763
C	5.496205	5.958272	17.745656
N	6.129549	2.920653	19.897677
N	2.220416	4.061071	17.845045
C	3.172369	6.440774	16.636891
O	1.969960	6.198665	16.400842
N	6.115462	7.007742	17.282873
C	7.637425	5.165666	19.019137
O	8.260329	4.364774	19.755940
O	8.276358	6.261032	18.547337
C	9.662800	6.403529	18.953373
C	10.181864	7.670250	18.301708
O	3.735204	7.556918	16.079619
C	2.863676	8.377917	15.253827
C	3.701633	9.530533	14.738596
H	3.811828	2.766821	19.166289
H	1.861024	3.163588	18.222032
H	1.654718	4.677481	17.250739
H	2.463897	7.761189	14.434843
H	2.016947	8.724377	15.865733
H	3.076635	10.180832	14.108195
H	4.099320	10.130271	15.570564
H	4.543271	9.163494	14.132729
H	7.113222	3.098448	20.138177
H	9.716350	6.456924	20.052303
H	10.229917	5.516040	18.631425
H	11.234703	7.822436	18.585097
H	10.120509	7.599030	17.205938
H	9.600654	8.544829	18.628575
H	5.603056	2.094024	20.234625
H	5.465197	7.549662	16.709390

AAA-DDD, EWG, model (**X-9**)

C	3.391302	-3.116342	22.765727
C	3.863863	-2.008985	22.034295
C	5.233151	-1.623365	22.240630
C	6.086868	-2.424933	23.025257
C	5.604533	-3.566382	23.652911
C	4.250855	-3.900365	23.531881
C	5.610190	-0.340000	21.712606
N	4.841348	0.491832	21.101217
C	3.554354	0.119212	20.796519
C	3.046660	-1.167717	21.179404
C	1.824391	-1.525149	20.584421
C	1.005278	-0.581515	19.941889
C	1.642871	0.668025	19.641614
N	2.878653	0.994604	20.040880
N	1.044362	1.584360	18.812516
C	-0.082482	1.297560	18.262074
C	-0.879904	0.127929	18.508552
C	-0.347817	-0.801932	19.466119
C	-2.164520	-0.067664	17.964410
C	-2.955022	-1.129549	18.383924
C	-2.485069	-1.982435	19.389612
C	-1.220696	-1.798033	19.944991
F	-0.567427	2.217512	17.416260
F	1.495265	-2.819275	20.512337
F	6.872896	0.068053	21.903154
F	7.372596	-2.108518	23.221054
F	6.407958	-4.324692	24.403494
F	3.776584	-4.955502	24.198291
F	2.090191	-3.436781	22.814234
F	-2.679528	0.766142	17.053001
F	-4.172690	-1.318371	17.868611
F	-0.908243	-2.574237	20.992770
F	-3.279882	-2.952078	19.847697
N	2.160537	4.257251	18.212432
C	3.488647	4.451611	18.094000
N	4.281121	3.504174	18.707654
C	5.632443	3.691909	18.937026
C	6.292146	4.771137	18.332220
C	5.569933	5.549965	17.299767
C	4.086459	5.528602	17.410616
C	7.660823	5.052758	18.749944
O	8.101044	6.291083	18.422996
C	9.455025	6.617205	18.828729
C	9.763060	7.991276	18.268079
N	6.213020	2.781215	19.741416
C	3.203375	6.498881	16.782605
O	3.849909	7.562764	16.221315
C	3.006880	8.567342	15.593180
C	3.927520	9.632059	15.033396
N	6.251533	6.149353	16.369303
O	1.958935	6.436344	16.744605
O	8.378784	4.276070	19.414006
H	3.816083	2.714032	19.180699
H	1.792719	3.345539	18.510470
H	1.586874	4.901767	17.656860
H	2.404811	8.086841	14.806934
H	2.318045	8.974799	16.348962
H	3.325581	10.416502	14.550470
H	4.524521	10.094061	15.833319
H	4.610619	9.207186	14.282965
H	7.209756	2.938519	19.924791
H	9.518474	6.598990	19.928642
H	10.143126	5.852034	18.437094
H	10.786813	8.280051	18.550177
H	9.686914	7.988204	17.171031
H	9.065966	8.743633	18.665866
H	5.664361	2.093709	20.268331
H	5.607592	6.656896	15.753161

AAA-DDD, wd ester, model (**X-10**)

C	6.106308	5.154754	18.934353
C	5.475232	3.961742	19.346462
C	4.091318	3.790144	19.057311
C	3.373911	4.794754	18.371316
C	4.015345	5.958837	17.977444
C	5.385527	6.134020	18.262516
C	3.449916	2.575005	19.472138
N	4.033460	1.600820	20.123041
C	5.372268	1.735951	20.438241
C	6.142494	2.887757	20.056591
C	7.498182	2.897293	20.397482
C	8.065069	1.829027	21.098490
C	7.196818	0.743730	21.462791
N	5.897275	0.709862	21.127270
N	7.636932	-0.346101	22.190045
C	8.898928	-0.392555	22.533950
C	9.878701	0.605174	22.209863
C	9.463412	1.753722	21.477243
C	11.224771	0.460855	22.612584
C	12.152877	1.439992	22.293203
C	11.746956	2.579068	21.566761
C	10.426392	2.737082	21.164577
H	9.220791	-1.264440	23.115704
H	8.119815	3.747211	20.115151
H	2.388799	2.440980	19.229476
H	2.313505	4.638541	18.159785
H	3.465442	6.739191	17.448820
H	5.887334	7.052534	17.952179
H	7.164198	5.319583	19.141910
H	11.517747	-0.428980	23.174575
H	13.194452	1.333414	22.600636
H	10.142912	3.627361	20.602407
H	12.480363	3.347763	21.315982
N	5.548368	-1.781059	23.804853
C	4.498200	-2.326449	23.069133
N	4.217611	-1.640189	21.907256
C	3.174685	-2.008464	21.082861
C	2.376339	-3.088039	21.385839
C	2.642371	-3.870203	22.586118
C	3.732806	-3.410709	23.445207
C	1.250640	-3.468961	20.462783
N	3.057988	-1.250093	19.923195
C	4.003406	-4.163861	24.718937
N	1.895618	-4.934900	22.840418
H	4.807264	-0.829259	21.641150

H	6.272668	-1.319254	23.230581
H	5.961645	-2.433572	24.465389
H	2.159336	-1.360159	19.459174
H	3.335716	-0.260186	20.019455
H	2.203500	-5.371105	23.717351
H	3.069313	-4.294971	25.293517
H	4.398007	-5.178094	24.524476
H	4.710534	-3.650981	25.386137
H	0.654046	-4.261041	20.934775
H	0.585226	-2.612837	20.245902
H	1.617028	-3.850560	19.492362

AAAA-DDDD, cation, Leigh (**X-11**)

N	-1.636518	14.828946	18.555996
C	-1.870216	13.920762	17.542866
C	-2.750687	12.789700	17.756785
C	-3.405837	12.633422	19.046718
C	-3.117287	13.604670	20.045325
C	-2.221713	14.665985	19.718637
C	-2.907014	11.905468	16.702753
C	-2.243083	12.134998	15.485893
C	-1.405627	13.306308	15.353795
N	-1.230947	14.168472	16.387803
C	-3.712748	13.505145	21.318183
C	-4.584580	12.463876	21.598335
C	-4.893688	11.489163	20.620829
C	-4.293804	11.592902	19.359362
N	-0.763822	13.587358	14.191843
C	-0.912853	12.742456	13.157564
C	-1.707437	11.531270	13.206993
C	-2.368956	11.260785	14.394134
N	-0.249127	13.097485	12.001707
C	-0.330338	12.323189	10.945291
C	-1.063911	11.101965	10.877614
C	-1.776459	10.679356	12.030902
C	-1.089536	10.316814	9.703635
C	-1.807071	9.135926	9.680640
C	-2.528397	8.690820	10.819288
C	-2.500605	9.472753	11.975216
C	-5.859124	10.335040	20.895351
C	-7.035367	10.418537	19.898487
C	-3.302337	7.376541	10.738192
C	-4.338357	7.473184	9.597763
C	-5.116343	8.997413	20.690961
C	-6.425547	10.367383	22.321774
C	-2.312081	6.232789	10.427810
C	-4.040879	7.042492	12.041409
H	-1.995312	15.413639	20.488858
H	-3.480015	14.255938	22.076663
H	-5.036717	12.401976	22.587385
H	-4.533565	10.840844	18.606727
H	-3.539392	11.020331	16.792582
H	-2.996211	10.374441	14.505443
H	-3.049814	9.136159	12.853263
H	-1.819416	8.533137	8.771584
H	-0.537036	10.653315	8.823500
H	0.215989	12.654908	10.053789
H	-7.113105	9.518941	22.451895
H	-6.988490	11.294310	22.511420
H	-5.628452	10.278300	23.076187
H	-6.685956	10.343799	18.857734
H	-7.581503	11.366772	20.016379
H	-7.732490	9.588102	20.087707
H	-4.732600	8.903260	19.664100
H	-5.809804	8.162678	20.875889
H	-4.270463	8.910080	21.389664
H	-4.781296	7.816844	12.295770
H	-3.341972	6.929584	12.885087
H	-4.575323	6.089377	11.915149
H	-5.061576	8.279194	9.794469
H	-4.885957	6.520897	9.524586
H	-3.856053	7.665956	8.628227
H	-1.563653	6.135161	11.229327
H	-1.786474	6.402163	9.476542
H	-2.866100	5.284579	10.350346
C	2.267698	15.181647	9.312226
C	2.135245	15.685190	10.608140
C	2.857179	16.825236	11.057686
C	3.741465	17.483759	10.194133
C	3.879087	16.980688	8.898108
C	3.154618	15.850731	8.463638
N	2.531408	17.093671	12.382615
C	1.654568	16.146712	12.700093
N	1.369358	15.281236	11.689126
N	1.041743	15.985421	13.938751
C	1.263059	16.819960	14.991661
N	2.081339	17.852949	14.890931
N	0.605221	16.546897	16.152110
C	0.752704	17.309589	17.306384
N	0.063892	16.972121	18.430867
C	0.422175	17.899782	19.395489
C	1.343528	18.775592	18.757106
N	1.531568	18.378053	17.438233
C	0.046973	18.058534	20.731573
C	0.622082	19.127627	21.425200
C	1.536692	20.003656	20.803126
C	1.909123	19.842456	19.466379
H	0.723548	14.444237	11.788078
H	0.393683	15.171362	14.055931
H	4.303083	18.357606	10.529129
H	4.563081	17.471589	8.203429
H	3.289738	15.490931	7.442179
H	1.707378	14.308811	8.970745
H	2.204131	18.434816	15.741445
H	2.554230	17.985651	13.976888
H	-0.032538	15.717914	16.197298
H	-0.600302	16.146511	18.494369
H	-0.659501	17.383618	21.219129
H	0.357937	19.287552	22.472022
H	1.961748	20.825934	21.381592
H	2.616716	20.519855	18.985213

AAAA-DDDD, cation, 2CH-N, Leigh (**X-13**)

C	1.444828	17.964748	20.655163
C	0.778273	16.730438	20.522992
C	0.447368	15.999789	21.695180
C	0.777673	16.523684	22.963745
C	1.427349	17.740207	23.063081
C	1.778277	18.483961	21.907004
C	-0.216051	14.736417	21.557155
N	-0.562788	14.176317	20.423880
C	-0.270953	14.855215	19.259375
C	0.400371	16.141644	19.248522
C	0.627836	16.736317	18.012676
C	0.225739	16.090477	16.827668
C	-0.423546	14.807911	16.939268
N	-0.664008	14.235024	18.138454
N	-0.825884	14.110445	15.853202
C	-0.639356	14.632586	14.633928
C	-0.017164	15.926029	14.419803
C	0.412882	16.627187	15.539051
N	-1.083475	13.840063	13.594467
C	-0.940606	14.296684	12.374845
C	-0.357763	15.557474	12.017157
C	0.125819	16.404621	13.053208
C	0.701743	17.635836	12.703305
C	0.811979	18.047379	11.369516
C	0.322943	17.189091	10.357730
C	-0.251390	15.966121	10.673876
C	1.454089	19.400256	11.056420
C	2.899066	19.407263	11.598899
C	2.504176	19.817516	22.076796
C	3.828869	19.575246	22.831579
C	1.496165	19.697909	9.551285
C	0.645962	20.515813	11.752971
C	2.823090	20.493477	20.736920
C	1.615370	20.767830	22.907655
N	-2.505042	12.106700	18.271120
C	-2.439679	11.127010	17.338424
N	-1.712000	11.405478	16.228351
C	-1.420216	10.508087	15.205161
C	-0.573769	10.717987	14.126867
N	-0.640522	9.551855	13.436287
N	-1.466678	8.669087	14.026989
N	-1.945889	9.252102	15.112128
C	-0.011141	9.246444	12.152357
C	-0.644633	10.038312	11.007820
C	0.017005	9.717956	9.667921
C	-0.612420	10.486339	8.503075
C	0.055314	10.166932	7.164307
C	-3.271871	12.071962	19.431213
N	-3.876926	10.954441	19.928421
N	-4.485871	11.268181	21.059980
N	-4.264294	12.578517	21.277820
C	-3.519196	13.131690	20.289550
C	-4.727988	13.233356	22.501240
C	-3.554973	13.771796	23.321482
C	-4.021580	14.469148	24.598776
C	-2.855324	15.045946	25.406718
C	-3.320101	15.749112	26.683680
N	-3.061656	9.961238	17.499006
H	-3.213965	14.169126	20.282970
H	-1.810499	12.904814	18.200437
H	-2.984769	9.263463	16.748358
H	-3.536302	9.784946	18.392746
H	-1.392119	12.400863	16.083931
H	0.003732	11.575237	13.806785
H	-1.301299	13.649965	11.564448
H	-0.628776	15.310981	9.884966
H	0.394953	17.487252	9.312225
H	1.075636	18.297860	13.485240
H	0.898995	17.600027	15.443088
H	1.116030	17.710053	17.938446
H	1.709987	18.526593	19.760462
H	1.678114	18.134845	24.048784
H	0.517970	15.955131	23.860159
H	-0.456590	14.179601	22.471506
H	1.966944	20.679600	9.394504
H	0.484648	19.731852	9.117556
H	2.087032	18.945667	9.006066
H	0.624678	20.376639	12.844109
H	-0.390829	20.535129	11.383907
H	1.111229	21.490734	11.540339
H	2.920544	19.249595	12.687611
H	3.366225	20.381156	11.384410
H	3.499636	18.618495	11.120609
H	1.906448	20.720665	20.170065
H	3.477925	19.864952	20.113313
H	3.346133	21.441868	20.929662
H	0.664441	20.965893	22.389267
H	2.140875	21.724388	23.053303
H	1.390664	20.345366	23.898195
H	4.486972	18.902249	22.260730
H	3.653545	19.132823	23.823401
H	4.347858	20.536052	22.972067
H	-0.133415	8.165489	12.001028
H	1.062827	9.468593	12.238607
H	-1.720830	9.803858	10.963798
H	-0.553089	11.118068	11.217287
H	1.094785	9.953698	9.721980
H	-0.058987	8.633893	9.470791
H	-1.687964	10.244154	8.453321
H	-0.543917	11.569975	8.706233
H	-0.413205	10.720722	6.336354
H	1.124901	10.431133	7.188039
H	-0.019243	9.090971	6.938650
H	-5.291800	12.474077	23.059178
H	-5.415992	14.045359	22.221650
H	-2.878682	12.937359	23.570635
H	-2.980168	14.484287	22.706278
H	-4.722856	15.281999	24.340174
H	-4.583148	13.755371	25.226429
H	-2.155481	14.231182	25.661198
H	-2.295033	15.755333	24.772019
H	-2.470529	16.154971	27.253776

H -3.999996 16.582574 26.444711
H -3.864948 15.049365 27.337585

AAAA-DDDD, cation, model (**X-15**)
H 2.943708 15.185635 9.105910
C 2.534584 15.433428 10.099871
N 2.819898 16.526807 10.720104
C 3.672926 17.402847 10.072910
N 4.043325 18.483510 10.686914
C 4.919081 19.286550 9.983277
N 5.415672 20.338249 10.547575
C 6.319161 21.063074 9.766345
N 6.908696 22.074999 10.274545
C 7.857863 22.806213 9.459011
H 4.020129 17.152564 9.050847
H 5.186478 18.996256 8.948833
H 6.501357 20.724259 8.727010
H 1.859410 14.717915 10.579003
H 7.547871 23.862258 9.414182
H 8.841228 22.786613 9.956068
H 7.958596 22.406034 8.434303
N 6.084119 22.981011 12.863563
H 6.623946 23.786437 13.163681
C 5.321721 22.378215 13.809063
N 4.688186 21.192619 13.355090
C 3.809187 20.453528 14.098538
N 3.247378 19.356398 13.498736
C 2.277647 18.519544 14.102173
N 1.771391 17.578630 13.256273
H 1.177081 16.875631 13.685001
N 5.110826 22.793121 15.022732
N 3.509583 20.785367 15.334611
N 1.921059 18.725591 15.332281
H 2.244482 17.282402 12.388806
H 3.523592 19.129908 12.527123
H 3.992721 21.652878 15.676624
H 2.815708 20.155830 15.799991
H 4.901295 20.882597 12.386933
H 6.350480 22.557282 11.949749
H 5.622236 23.656678 15.219311
H 1.210649 18.055191 15.636098

AAAA-DDDD, neutral, Leigh (**X-12**)
C 2.602769 15.019001 9.535724
C 2.380810 15.540344 10.825823
C 3.088314 16.703952 11.223263
C 3.992841 17.317982 10.330625
C 4.192205 16.781940 9.072256
C 3.498893 15.618149 8.650151
C 1.466824 14.953962 11.792534
C 1.339702 15.563457 13.103125
N 2.057274 16.698662 13.425547
C 2.865061 17.220291 12.537738
C 0.693154 13.831649 11.541713
C -0.156331 13.326024 12.540256
C -0.198522 13.993183 13.824836
N 0.545155 15.098378 14.076741
N -0.985908 13.539983 14.832613
C -1.739948 12.454997 14.611139
C -1.778844 11.729358 13.356056
C -0.964397 12.193384 12.335873
C -2.649880 10.568634 13.236913
C -3.407771 10.203355 14.383405
C -3.277332 10.996704 15.566597
N -2.508218 12.048606 15.686279
C -4.263489 9.086094 14.334130
C -4.372653 8.342254 13.168487
C -3.635812 8.688208 12.012487
C -2.784561 9.798469 12.070525
C -3.743523 7.895496 10.707608
C -4.711083 6.708836 10.819505
C 3.767132 15.062706 7.252117
C 2.930614 13.814752 6.940754
C -4.247763 8.833569 9.590171
C -2.349344 7.354723 10.324643
C 3.437839 16.148750 6.205425
C 5.262321 14.692935 7.142628
H -3.862707 10.714261 16.450085
H -4.839009 8.814044 15.222045
H -5.040073 7.481533 13.148698
H -2.216858 10.067691 11.179191
H -0.930654 11.695393 11.365188
H 0.728018 13.322137 10.577219
H 2.067063 14.124264 9.221914
H 4.897478 17.261447 8.391583
H 4.531028 18.214030 10.648346
H 3.411735 18.121305 12.842149
H -4.746420 6.183614 9.853783
H -5.731934 7.041004 11.063146
H -4.383549 5.990449 11.586650
H -3.560488 9.678240 9.433959
H -5.242526 9.235078 9.837907
H -4.323029 8.270889 8.646358
H -1.625533 8.170793 10.180951
H -2.422339 6.791029 9.381544
H -1.963722 6.681064 11.105248
H 1.851725 14.031844 6.983073
H 3.153349 12.994924 7.641587
H 3.168720 13.467300 5.924493
H 2.374581 16.429596 6.254564
H 3.651346 15.760716 5.197260
H 4.044410 17.053293 6.358940
H 5.526242 13.912935 7.873215
H 5.906542 15.566966 7.319713
H 5.471162 14.311820 6.130890
N -2.446628 13.220223 18.207591
C -1.756714 14.325965 18.641543
N -1.960682 14.614437 19.925281
C -2.841675 13.635074 20.364823
C -3.158574 12.748927 19.296881
C -3.406889 13.433775 21.630282
C -4.275818 12.349293 21.798363
C -4.580508 11.479640 20.732277
C -4.025003 11.666589 19.459633
N -0.948152 15.015582 17.765685
C -0.204990 16.163988 18.131725
N -0.173877 16.714427 19.285370
N 0.523149 16.650879 17.037000
C 1.356863 17.753918 17.043221
N 1.600605 18.603108 18.031159
C 2.506263 19.504264 17.487610
C 2.796050 19.168567 16.133626
N 2.041466 18.040500 15.878243
C 3.123630 20.617269 18.074386
C 4.013095 21.367314 17.295706
C 4.287990 21.023330 15.956565
C 3.681225 19.914159 15.351903
H 1.997351 17.497871 14.981872
H 0.481576 16.134829 16.150056
H 2.912257 20.882292 19.112621
H 4.506458 22.237390 17.733792
H 4.986797 21.630879 15.377976
H 3.900033 19.654534 14.313568
H -0.786341 16.184352 19.922146
H -0.906858 14.650180 16.804033
H -2.437691 12.823186 17.232717
H -4.263678 10.987088 18.638459
H -5.262734 10.643886 20.899915
H -4.727161 12.171430 22.776755
H -3.172090 14.107416 22.456880

AAAA-DDDD, neutral, 2CH-N, Leigh (**X-14**)
N -2.974185 14.043763 21.774260
C -3.013989 12.912044 21.000433
C -3.772494 11.951381 21.660262
N -4.148587 12.573780 22.814097
N -3.674385 13.830565 22.877391
N -2.365353 12.898144 19.784117
C -2.213080 11.776057 18.981926
N -2.705892 10.628458 19.315726
C -5.039556 12.062374 23.849578
N -1.474323 12.082593 17.832408
C -1.189672 11.196479 16.804946
C -0.722426 11.528790 15.538268
N -0.562215 10.326039 14.929700
N -0.916152 9.307018 15.734414
N -1.297740 9.835112 16.891301
C -0.095546 10.093731 13.566268
C -1.056541 10.664319 12.523502
C -0.439107 10.638923 11.125214
C -1.373684 11.210806 10.057397
C -0.695832 11.334221 8.690860
N -0.130489 14.009148 12.566621
C 0.244721 14.297104 11.348199
C 0.906659 15.505334 10.937240
C 1.209298 16.485463 11.921626
C 0.830407 16.183301 13.293297
C 0.136017 14.937108 13.562721
C 1.247703 15.739601 9.591876
C 1.874758 16.921954 9.222280
C 2.183261 17.912261 10.182950
C 1.844630 17.671634 11.519135
C 1.085528 17.015418 14.374562
C 0.659034 16.649892 15.666215
C -0.055474 15.402213 15.825065
N -0.290831 14.584001 14.776858
N -0.529224 14.997177 17.029764
C -0.317939 15.777780 18.094389
C 0.423322 17.024786 18.046594
C 0.890719 17.439676 16.806944
C 0.619737 17.765978 19.281621
C 0.023391 17.239266 20.456880
C -0.718505 16.015269 20.368638
N -0.875054 15.314653 19.272188
C 0.175121 17.919220 21.684526
C 0.910697 19.089332 21.742871
C 1.522036 19.631905 20.583517
C 1.362751 18.960490 19.371291
C 2.870894 19.229077 9.812785
C 3.159436 19.340476 8.309359
C 2.334336 20.921211 20.706847
C 2.926341 21.377048 19.366245
C 1.421754 22.042575 21.246885
C 3.493202 20.689270 21.699602
C 1.963578 20.407831 10.224893
C 4.207956 19.329724 10.577238
H -4.031185 10.934644 21.400381
H -1.852164 13.771090 19.536070
H -2.468455 9.932230 18.599732
H -1.264344 13.069364 17.618808
H -0.524424 12.490809 15.066691
H 0.025411 13.547022 10.577198
H 1.009316 14.981461 8.841287
H 2.127650 17.083699 8.174856
H 2.077139 18.435217 12.262868
H 1.618422 17.960455 14.248452
H 1.438226 18.377782 16.694474
H 1.829043 19.366429 18.474479
H 1.024804 19.603203 22.698535
H -0.291721 17.506395 22.581804
H -1.213957 15.614704 21.263050
H 3.649306 20.305222 8.109170
H 2.232685 19.298260 7.716759
H 3.832504 18.539246 7.967133
H 1.766376 20.403517 11.307140
H 0.999618 20.361626 9.695836
H 2.458250 21.358155 9.970107
H 4.048920 19.304182 11.665406
H 4.706459 20.278865 10.324055
H 4.877254 18.499999 10.303157
H 2.137579 21.581659 18.625576
H 3.612814 20.622341 18.951367
H 3.495854 22.305215 19.522185
H 0.589071 22.235636 20.553260
H 2.006497 22.969205 21.358176

H	1.002606	21.780661	22.229560
H	4.163312	19.894106	21.337629
H	3.119295	20.402507	22.693468
H	4.076239	21.617567	21.805477
H	0.023727	9.006866	13.459868
H	0.895011	10.564455	13.464184
H	-1.999214	10.093226	12.542599
H	-1.280720	11.710615	12.784289
H	0.495525	11.228461	11.142315
H	-0.154009	9.608045	10.848494
H	-2.269067	10.571518	9.976710
H	-1.732810	12.200750	10.389434
H	-1.381425	11.739551	7.931161
H	0.181750	12.000957	8.747321
H	-0.339625	10.352378	8.339926
C	-6.501758	12.052996	23.400150
H	-4.707355	11.047719	24.116428
H	-4.903575	12.714606	24.723478
C	-7.429403	11.548151	24.505975
H	-6.789986	13.075663	23.105185
H	-6.602858	11.414377	22.507017
C	-8.897479	11.512957	24.074153
H	-7.116813	10.534768	24.815908
H	-7.326495	12.194569	25.396061
C	-9.826770	11.026222	25.188360
H	-9.201061	12.522968	23.748303
H	-8.995847	10.857104	23.192002
H	-10.876449	11.002013	24.857021
H	-9.549365	10.009961	25.512595
H	-9.762816	11.687740	26.067939

AAAA-DDDD, neutral, model (**X-16**)

C	1.819125	15.890878	9.064685
N	1.746609	15.991853	10.346137
C	2.471493	17.021525	10.930754
N	2.326395	17.235190	12.197869
C	3.094780	18.248029	12.740411
N	2.932084	18.555624	13.983842
C	3.736670	19.572143	14.492159
N	3.557162	19.958016	15.698145
C	4.402787	21.019560	16.204861
H	2.401573	16.589750	8.439123
H	3.156338	17.619486	10.294856
H	3.834057	18.763792	12.094294
H	4.510145	20.015295	13.829461
H	1.275344	15.079760	8.570296
H	4.932769	20.654700	17.099530
H	5.139832	21.394109	15.469455
H	3.764892	21.854873	16.537631
N	0.857095	19.231743	16.868880
C	0.034299	18.211785	16.417319
N	-1.224267	18.075565	16.683059
N	0.708927	17.296167	15.602213
C	0.442151	15.920302	15.525980
N	-0.032936	15.246759	16.504870
N	0.806363	15.427463	14.252560
C	0.716711	14.103858	13.830157
N	0.509039	13.138391	14.674393
N	0.807672	13.960843	12.457282
H	0.384879	20.100604	17.101990
H	1.061926	13.034159	12.129320
H	1.116164	14.733417	11.856989
H	1.099406	16.114883	13.550339
H	-0.126363	14.265337	16.198657
H	1.513471	17.658723	15.070360
H	1.806135	19.347003	16.485226
H	-1.509587	18.807964	17.344121
H	0.413215	12.250123	14.171116

Molecules optimized at CPCM/M06-2X/6-31-G** level of theory using Gaussian 09

AA-DD Np

C	-3.528425	0.051293	0.435646
C	-2.146122	-0.040928	0.561225
C	-1.594303	-0.198800	1.834272
C	-2.399826	-0.266006	2.969509
C	-3.782400	-0.174557	2.853318
C	-4.314103	-0.017465	1.578961
C	-1.246852	0.030633	-0.671726
C	-0.473643	-1.259988	-0.857099
C	0.880645	-1.312670	-0.630825
N	1.587648	-0.173982	-0.326065
C	1.029577	1.085765	-0.382015
C	-0.324929	1.232117	-0.609052
N	1.641318	-2.446436	-0.645236
C	-1.184844	-2.458471	-1.243975
O	-2.497213	-2.216643	-1.445059
C	-3.318035	-3.346353	-1.759710
C	-4.744606	-2.845594	-1.832176
C	-0.884028	2.553938	-0.727073
O	-2.217028	2.516565	-0.959145
C	-2.890824	3.776123	-1.024376
C	-4.368049	3.477401	-1.167680
N	1.905798	2.089022	-0.184879
N	-5.770053	0.081025	1.432445
O	-6.452876	0.047242	2.442160
O	-0.717594	-3.587549	-1.385552
O	-0.287849	3.627821	-0.635460
O	-6.226867	0.192206	0.305731
N	4.879291	1.248481	-0.271372
C	5.307229	0.059158	0.231124
C	6.672306	-0.198329	0.512753
C	7.613036	0.826099	0.243116
C	7.168995	2.014637	-0.269990
C	5.781194	2.175627	-0.505585
N	4.340127	-0.871826	0.450638
C	4.692871	-2.033406	0.955637
C	6.026572	-2.381316	1.279255
C	7.016075	-1.462645	1.051441
H	6.239769	-3.359279	1.693440
H	7.848191	2.828587	-0.492840
H	5.411537	3.117955	-0.903406
H	3.889511	-2.749578	1.118371
H	2.566815	-0.286825	-0.035891
H	-1.917444	0.153193	-1.528600
H	2.901283	1.894788	-0.262126
H	1.540618	3.025096	-0.304492
H	-2.674958	4.345866	-0.115590
H	-2.505937	4.349010	-1.874140
H	-4.936909	4.408843	-1.213431
H	-4.558416	2.909126	-2.081521
H	-4.725931	2.890927	-0.317178
H	1.156283	-3.259943	-1.009170
H	2.604886	-2.326816	-0.927531
H	-2.986702	-3.778783	-2.708881
H	-3.189070	-4.107550	-0.984676
H	-5.420805	-3.672837	-2.059542
H	-5.045588	-2.399502	-0.880439
H	-4.846931	-2.088706	-2.613674
H	-0.512984	-0.268286	1.931484
H	-1.951208	-0.389633	3.949122
H	-4.437113	-0.221588	3.713888
H	-3.988509	0.176261	-0.537994
H	8.665289	0.652264	0.447963
H	8.055781	-1.681293	1.276895

AAA-DDD, cation, Leigh

C	3.677745	-2.694812	0.126958
C	2.284995	-2.506651	0.079197
C	1.449407	-3.642329	0.085705
C	1.997746	-4.936995	0.137951
C	3.365979	-5.100783	0.185313
C	4.203407	-3.971804	0.179330
C	0.025744	-3.445744	0.028028
N	-0.564006	-2.292421	-0.007897
C	0.216751	-1.150548	0.007084
C	1.638248	-1.205851	0.029239
C	2.324507	0.004623	-0.001337
C	1.632312	1.211739	-0.031855
C	0.211148	1.149528	-0.009847
N	-0.464580	-0.002145	-0.001547
N	-0.575129	2.287610	0.005771
C	0.009075	3.443800	-0.028195
C	1.431802	3.647358	-0.084869
C	2.272801	2.515685	-0.080458
C	3.664626	2.710609	-0.128347
C	4.184123	3.990223	-0.178131
C	3.341332	5.115190	-0.181277
C	1.973893	4.944757	-0.134274
N	-3.386613	2.159670	0.787836
C	-4.150060	1.127276	0.401685
N	-3.500561	-0.005894	-0.000178
C	-4.144591	-1.142346	-0.401481
C	-5.541875	-1.153784	-0.409983
C	-6.215223	-0.012804	0.001089
C	-5.547312	1.131665	0.411396
N	-3.376451	-2.171077	-0.787804
H	-0.614766	-4.327469	0.010029
H	1.333396	-5.795895	0.141275
H	3.797934	-6.094334	0.227962
H	5.279449	-4.104024	0.217646
H	4.351519	-1.845958	0.126874
H	3.407880	0.007339	-0.001038
H	4.342452	1.864979	-0.130407
H	5.259504	4.127669	-0.216498
H	3.768540	6.110889	-0.221460
H	1.305368	5.800430	-0.135600
H	-0.635704	4.322404	-0.009318
H	-2.464510	-0.003478	-0.000681
H	-2.381960	-2.204120	-0.532349
H	-3.836162	-3.043371	-0.996785
H	-6.063930	-2.047857	-0.722990
H	-7.300479	-0.015638	0.001773
H	-6.073616	2.023007	0.725008
H	-2.392677	2.196827	0.531034
H	-3.850344	3.030387	0.994717

AAA-DDD, neutral, Leigh

C	-5.724500	-0.130368	0.215814
C	-4.968375	-1.299344	0.215167
C	-3.566917	-1.170033	0.003100
N	-2.964550	0.007397	-0.171689
C	-3.703930	1.117948	-0.161333
C	-5.115789	1.106569	0.023172
C	-5.523328	-2.628368	0.412919
C	-4.625370	-3.713984	0.371041
C	-3.231038	-3.443322	0.131303
N	-2.722501	-2.265904	-0.040057
N	0.031127	-2.041596	-1.213689
C	0.830844	-1.001540	-0.912123
N	0.172692	0.108564	-0.418277
C	0.797853	1.331891	-0.269738
C	2.166270	1.440185	-0.433866
C	3.030759	0.222148	-0.681505
C	2.201439	-0.977291	-1.091323
C	3.886595	-0.096389	0.544917
C	5.275059	-0.054911	0.469283
C	6.020619	-0.347183	1.604117
C	5.442826	-0.681932	2.822915
C	4.054617	-0.720916	2.889794
C	3.289047	-0.431308	1.761425
N	7.483061	-0.300488	1.510013
O	7.980008	-0.000047	0.435864
C	2.794713	2.729987	-0.311954
O	2.254254	3.808300	-0.059132
N	-0.021240	2.347318	0.060672
C	2.870271	-2.152084	-1.587469
O	2.362842	-3.234816	-1.885937
O	4.132164	2.657828	-0.510317
C	4.872173	3.867946	-0.336306
C	6.334615	3.515583	-0.507246
O	4.205594	-1.959536	-1.704588
C	4.980633	-3.088745	-2.114396
C	6.428641	-2.646782	-2.127413

O	8.132329	-0.564430	2.508130
C	-5.830796	2.372501	0.003805
C	-5.082958	-5.031645	0.552459
H	-2.543884	-4.288761	0.086929
N	-2.992299	2.290594	-0.345469
H	-0.847349	0.074871	-0.346816
H	3.733957	0.451713	-1.489310
H	-0.913318	-2.078217	-0.831645
H	0.512749	-2.895835	-1.466095
H	4.812072	-3.912437	-1.413530
H	4.645040	-3.420426	-3.101900
H	7.072159	-3.478895	-2.422278
H	6.572880	-1.827855	-2.836514
H	6.735832	-2.301434	-1.136556
H	0.417700	3.259750	0.092880
H	4.539977	4.605476	-1.073839
H	4.664516	4.276888	0.657296
H	6.953980	4.405652	-0.374334
H	6.637983	2.765243	0.228021
H	6.517318	3.110485	-1.505680
H	2.203193	-0.464667	1.819208
H	3.569758	-0.977851	3.825351
H	6.067537	-0.902190	3.678948
H	5.770706	0.200206	-0.460615
C	-6.886303	-2.890398	0.642821
H	-6.796409	-0.183929	0.365512
C	-3.641971	3.409548	-0.360682
H	-1.024392	2.269762	-0.105426
H	-3.056788	4.318406	-0.504380
C	-5.067224	3.539767	-0.198524
C	-5.682864	4.803844	-0.230784
C	-7.047566	4.907650	-0.062478
H	-5.071470	5.687713	-0.387958
H	-7.530723	5.878162	-0.085971
C	-7.814007	3.747163	0.140518
C	-7.221565	2.499030	0.173454
H	-8.887540	3.831382	0.273326
H	-7.841486	1.623830	0.332065
C	-6.422960	-5.268449	0.776106
H	-4.370455	-5.850388	0.513317
H	-6.785270	-6.280649	0.917146
C	-7.322541	-4.189527	0.820871
H	-7.605605	-2.080424	0.684823
H	-8.376042	-4.378601	0.998187

AAA-DDD, OH, Leigh

C	-3.304021	-1.063359	-0.643437
C	-4.458315	-0.267555	-0.618881
C	-5.411941	-0.484454	0.368643
C	-5.251693	-1.480693	1.336469
C	-4.077297	-2.231890	1.305931
C	-3.086456	-2.035010	0.342260
C	-4.572674	0.826219	-1.652536
O	-3.533685	1.785668	-1.541246
C	-6.320948	-1.745885	2.366547
C	-1.777485	-2.785683	0.432771
O	-1.248504	-3.065734	-0.854599
O	-2.428384	-0.818728	-1.650927
N	1.439182	-2.436726	-0.463381
C	1.539893	-1.061422	-0.296872
C	2.780496	-0.428248	-0.008628
C	3.978382	-1.246232	0.084504
C	3.827456	-2.635541	-0.101884
C	2.510623	-3.155229	-0.367529
N	0.390792	-0.400751	-0.424788
C	0.387468	0.922947	-0.258831
C	1.566660	1.661956	0.056764
C	2.758650	0.952699	0.170133
C	1.461197	3.100659	0.242007
C	0.183487	3.675655	0.093005
C	-0.923861	2.812823	-0.241055
N	-0.847297	1.532854	-0.410789
C	2.545302	3.939847	0.558909
C	2.349147	5.298392	0.718028
C	1.072512	5.866896	0.568021
C	-0.002314	5.059722	0.257516
C	4.939345	-3.494414	-0.030153
C	6.192336	-2.977478	0.223015
C	6.348921	-1.592976	0.408850
C	5.265121	-0.738508	0.341997
H	-3.917683	-2.997177	2.064125
H	-6.303885	0.139284	0.383711
H	-1.911500	3.257925	-0.372354
H	3.677489	1.478472	0.402182
H	2.398534	-4.231142	-0.500787
H	4.795023	-4.560784	-0.177497
H	7.055085	-3.631999	0.277392
H	7.335839	-1.187275	0.605629
H	5.420043	0.324654	0.486547
H	-0.997299	5.477779	0.135870
H	0.937117	6.935241	0.696398
H	3.542311	3.532274	0.681702
H	3.193674	5.934079	0.962912
H	-0.272444	-2.964350	-0.784917
H	-1.675733	-1.433608	-1.543718
H	-2.694923	1.294381	-1.498148
H	-6.864934	-0.831291	2.616440
H	-7.054180	-2.472276	1.999369
H	-5.892959	-2.150128	3.287478
H	-1.933387	-3.715809	0.996680
H	-1.058179	-2.173725	0.996461
H	-5.519075	1.360293	-1.524696
H	-4.573044	0.378483	-2.657282

AAA-DDD, cation, model

C	6.447950	3.983324	-0.005562
C	5.924499	2.704382	-0.003879
C	4.531456	2.512038	-0.002915
C	3.693494	3.646021	-0.003707
C	4.239397	4.942724	-0.005388
C	5.607987	5.110439	-0.006323
C	2.269564	3.444995	-0.002797
N	1.684801	2.288970	-0.001572
C	2.466172	1.148376	-0.000961
C	3.887744	1.208607	-0.001261
N	1.784652	0.000004	-0.000076
C	2.466170	-1.148369	0.000882
C	3.887742	-1.208604	0.001329
C	4.577554	0.000001	0.000070
C	4.531451	-2.512038	0.003033
C	3.693488	-3.646019	0.003712
C	2.269558	-3.444988	0.002673
N	1.684797	-2.288962	0.001414
C	4.239385	-4.942725	0.005411
C	5.607974	-5.110445	0.006485
C	6.447939	-3.983333	0.005849
C	5.924492	-2.704388	0.004144
N	-1.188900	-2.264610	-0.002628
C	-1.980600	-1.207676	-0.001734
N	-1.342332	-0.000022	-0.000301
C	-1.980590	1.207638	0.001244
C	-3.398347	1.248028	0.001758
C	-4.118441	-0.000012	-0.000331
C	-3.398351	-1.248052	-0.002217
N	-1.188867	2.264559	0.002094
C	-4.014462	-2.582949	-0.004221
O	-5.357767	-2.598223	-0.005299
C	-5.984529	-3.899146	-0.006963
C	-7.478346	-3.668515	-0.007585
N	-5.447724	-0.000017	-0.000511
C	-4.014454	2.582931	0.004162
O	-5.357766	2.598235	0.005323
C	-5.984476	3.899182	0.007789
C	-7.478297	3.668626	0.008200
O	-3.395741	-3.638054	-0.005062
O	-3.395716	3.638037	0.005409
H	1.624752	4.323750	-0.003182
H	-0.311566	-0.000024	-0.000292
H	-0.160997	2.180441	0.001210
H	-1.640513	3.171550	0.003668
H	-5.647542	4.442138	0.893804
H	-5.648483	4.445098	-0.876752
H	-7.995475	4.630310	0.010252
H	-7.784614	3.113794	-0.881645
H	-7.783621	3.110555	0.896352
H	-1.640562	-3.171593	-0.003807
H	-5.647582	-4.442699	-0.892608
H	-5.648594	-4.444494	0.877958
H	-7.995591	-4.630164	-0.008872
H	-7.784637	-3.112951	0.881813
H	-7.783621	-3.111131	-0.896190
H	5.660866	-0.000001	0.000125
H	-0.161029	-2.180509	-0.001549
H	1.624745	-4.323742	0.002989
H	3.572780	-5.799901	0.005898
H	6.038108	-6.105711	0.007869
H	7.524259	-4.118648	0.006746
H	6.600319	-1.857178	0.003766
H	3.572794	5.799903	-0.005969
H	6.038126	6.105702	-0.007691
H	6.600323	1.857170	-0.003404
H	7.524271	4.118634	-0.006343
H	-5.947370	0.874746	0.000859
H	-5.947367	-0.874778	-0.002307

AAA-DDD, anion, model

C	-5.542703	3.258919	-0.289910
C	-4.165752	2.962491	-0.277639
C	-3.248341	4.025822	-0.408278
C	-3.701143	5.351192	-0.546735
C	-5.052991	5.619940	-0.556466
C	-5.973032	4.564165	-0.427066
C	-1.840778	3.725293	-0.389290
N	-1.345450	2.535133	-0.274384
C	-2.209859	1.463626	-0.158460
C	-3.621767	1.620782	-0.141478
C	-4.398936	0.473447	0.005313
C	-3.802657	-0.783478	0.119602
C	-2.372462	-0.817369	0.065652
N	-1.615282	0.268063	-0.062172
N	-1.665753	-2.012197	0.142426
C	-2.319184	-3.118781	0.277479
C	-3.761175	-3.228494	0.363863
C	-4.508049	-2.025086	0.278367
C	-4.394876	-4.441782	0.515571
C	-5.834584	-4.516363	0.594131
N	-6.547388	-3.312052	0.507089
C	-5.908959	-2.175067	0.361209
N	1.194550	-2.165264	-0.506174
C	2.073589	-1.164011	-0.412360
N	1.514575	0.079260	-0.246551
C	2.253203	1.200335	0.059449
C	3.644660	1.144714	0.022300
C	4.300162	-0.066736	-0.516466
C	3.466376	-1.294127	-0.490555
C	4.372938	2.329752	0.458879
O	5.670372	2.127094	0.734630
C	6.396758	3.273146	1.184733
C	7.834493	2.838081	1.370725
N	1.518858	2.268620	0.407237
C	4.004171	-2.640046	-0.549865
O	5.352391	-2.707948	-0.464494
C	5.917252	-4.023869	-0.492014
C	7.420870	-3.860313	-0.440988
N	5.493296	0.020146	-0.993435
O	3.351018	-3.680631	-0.634406
O	3.875001	3.442992	0.639302
H	-1.730094	-4.035995	0.327340
H	0.491826	0.143786	-0.191349
H	0.206124	-2.031277	-0.270306
H	1.590023	-3.092992	-0.595628
H	5.539443	-4.593265	0.362484
H	5.591659	-4.536479	-1.401760
H	7.902469	-4.840885	-0.451594
H	7.774655	-3.290071	-1.303386

H	7.719837	-3.334813	0.468795
H	2.035349	3.133584	0.501901
H	6.306654	4.072403	0.442857
H	5.957328	3.640508	2.117756
H	8.441857	3.683716	1.701982
H	7.903432	2.045452	2.119399
H	8.242096	2.460920	0.430054
H	-5.479479	0.554754	0.030559
H	0.517328	2.287315	0.204059
H	-1.134000	4.551214	-0.473950
H	-2.972074	6.150372	-0.645295
H	-5.409837	6.638500	-0.662995
H	-7.036974	4.777152	-0.435289
H	-6.276080	2.466376	-0.193556
H	-3.826523	-5.364836	0.579186
O	-6.448205	-5.588059	0.732801
H	-6.541606	-1.286373	0.304590
H	5.786777	-0.895723	-1.325002

AAA-DDD, neutral, model

C	-3.379951	1.174158	-0.554967
C	-1.992359	1.125587	-0.482691
N	-1.364754	-0.073473	-0.224347
C	-2.040398	-1.208277	0.147364
C	-3.436497	-1.245966	0.066771
C	-4.147393	-0.091268	-0.538718
N	-1.156442	2.161301	-0.658173
N	-1.255984	-2.204891	0.581245
C	-4.093969	-2.457955	0.524701
O	-3.534678	-3.484629	0.909722
N	-5.321537	-0.116757	-1.064829
C	-3.999246	2.491106	-0.646610
O	-3.405231	3.534798	-0.924153
O	-5.300893	2.524957	-0.327044
C	-5.923689	3.811245	-0.382682
C	-7.389163	3.604692	-0.065754
O	-5.442400	-2.387884	0.541636
C	-6.128006	-3.558029	1.005885
C	-7.610257	-3.276846	0.891173
N	1.689397	2.286145	0.242653
C	2.463933	1.143274	0.150671
C	3.886714	1.184956	0.179675
C	4.541412	2.472235	0.346055
C	3.712999	3.608131	0.447764
C	2.285888	3.426554	0.376460
C	4.563877	-0.023080	0.038197
C	3.860597	-1.216123	-0.103819
C	2.439542	-1.142411	-0.079123
N	1.773562	0.007803	0.032326
C	4.486820	-2.517135	-0.271288
C	3.633667	-3.634364	-0.376300
C	2.210948	-3.421747	-0.301932
N	1.639634	-2.268469	-0.166904
C	4.161807	-4.927423	-0.543952
C	5.527277	-5.108317	-0.609969
C	6.382325	-3.997604	-0.507639
C	5.876720	-2.722447	-0.340341
C	4.269300	4.889909	0.610690
C	5.638400	5.041342	0.675226
C	6.468985	3.911781	0.577177
C	5.935446	2.647367	0.414875
H	1.557000	-4.292170	-0.360739
H	-0.343094	-0.071978	-0.153411
H	-0.253241	-2.174956	0.396814
H	-1.722999	-3.083627	0.766086
H	-5.825652	-4.415721	0.398483
H	-5.828670	-3.760000	2.038510
H	-8.180189	-4.140619	1.240970
H	-7.885494	-2.409574	1.495430
H	-7.884549	-3.077808	-0.147631
H	-1.604953	3.055956	-0.813300
H	-5.440854	4.480455	0.336754
H	-5.779424	4.240131	-1.378794
H	-7.918498	4.559547	-0.105149
H	-7.841976	2.922858	-0.789150
H	-7.511290	3.179648	0.933162
H	5.647534	-0.034921	0.038785
H	-0.197835	2.120243	-0.311658
H	1.651560	4.311107	0.437719
H	3.608645	5.748703	0.684120
H	6.076146	6.025266	0.801679
H	7.545783	4.033261	0.630525
H	6.603824	1.796748	0.346545
H	3.482562	-5.771292	-0.620696
H	5.943706	-6.100905	-0.740487
H	6.563637	-1.887005	-0.268828
H	7.456188	-4.142391	-0.561749
H	-5.713764	-1.050590	-0.970489

AAA-DDD, wd ester, model

C	4.392994	-2.667907	-0.244297
C	2.998854	-2.487405	-0.193296
C	2.168127	-3.624738	-0.249050
C	2.723283	-4.913219	-0.350660
C	4.092574	-5.070123	-0.399127
C	4.925290	-3.939085	-0.345806
C	0.740127	-3.437186	-0.194611
N	0.142109	-2.292367	-0.117093
C	0.922216	-1.148885	-0.073724
C	2.345150	-1.193120	-0.090739
C	3.022845	0.019956	-0.002510
C	2.321463	1.219922	0.080507
C	0.900276	1.148045	0.051672
N	0.237254	-0.006592	-0.013633
N	0.097465	2.276107	0.087747
C	0.672266	3.432291	0.171891
C	2.095959	3.647376	0.240312
C	2.949018	2.526371	0.190415
C	2.625144	4.946117	0.350049
C	3.990633	5.129150	0.412581
C	4.845434	4.014528	0.365006
C	4.338907	2.733559	0.255336
H	0.021643	4.306896	0.190977
H	4.106507	0.030388	0.001744
H	0.106786	-4.324212	-0.219009
H	2.061953	-5.773716	-0.390640
H	4.528609	-6.059665	-0.479625
H	6.002144	-4.064919	-0.386139
H	5.062157	-1.815888	-0.209109
H	1.946976	5.793620	0.385748
H	4.406985	6.126580	0.499445
H	5.024651	1.894632	0.224869
H	5.919212	4.160976	0.416339
N	-2.706876	1.978544	-1.204166
C	-3.523392	1.028134	-0.602422
N	-2.830417	0.008573	0.000723
C	-3.481279	-1.042835	0.600635
C	-4.842545	-1.104160	0.642314
C	-5.636276	-0.043142	0.032159
C	-4.890784	1.026326	-0.631328
C	-5.524383	-2.259302	1.322573
N	-2.640945	-1.969232	1.200513
C	-5.666776	2.103922	-1.336329
N	-6.937941	-0.103495	0.099612
H	-1.807788	0.026410	0.002961
H	-1.796335	2.077354	-0.754847
H	-3.152995	2.879071	-1.304051
H	-3.098243	-2.851437	1.385851
H	-1.749901	-2.100723	0.721649
H	-7.343127	0.709932	-0.362840
H	-6.419906	1.657934	-1.996745
H	-6.204994	2.757104	-0.637760
H	-5.037319	2.734329	-1.967239
H	-6.600640	-2.090226	1.302472
H	-5.322300	-3.216272	0.822779
H	-5.209369	-2.366459	2.367955

AAAA-DDDD, Cation, Leigh

N	3.445514	-0.457157	-0.242725
C	2.308709	0.335964	-0.208178
C	2.404745	1.771008	-0.285995
C	3.715661	2.384111	-0.469257
C	4.824461	1.519398	-0.537150
C	4.601624	0.107058	-0.399663
C	1.233340	2.484402	-0.167225
C	0.010058	1.807763	-0.026236
C	0.010931	0.378992	-0.033225
N	1.163546	-0.325301	-0.097589
C	6.113094	2.041259	-0.715741
C	6.297263	3.405648	-0.827148
C	5.204299	4.292550	-0.763851
C	3.927294	3.761750	-0.586532
N	-1.139380	-0.327615	0.023982
C	-2.286560	0.331199	0.140055
C	-2.384267	1.764727	0.234304
C	-1.212784	2.481289	0.122496
N	-3.421937	-0.462123	0.163740
C	-4.578576	0.100583	0.327203
C	-4.800377	1.510143	0.485155
C	-3.693904	2.373665	0.428808
C	-6.092073	2.031064	0.678100
C	-6.268375	3.388779	0.816423
C	-5.170925	4.279803	0.766226
C	-3.899619	3.755615	0.571963
C	5.374200	5.809595	-0.880603
C	4.563706	6.316533	-2.086243
C	-5.425373	5.777901	0.933683
C	-6.385266	6.252199	-0.171772
C	4.852802	6.473408	0.405850
C	6.838633	6.213721	-1.076322
C	-6.065511	6.023620	2.311460
C	-4.136306	6.600276	0.841745
H	5.469776	-0.552175	-0.422617
H	6.961574	1.365149	-0.764290
H	7.300552	3.789570	-0.963729
H	3.082281	4.440480	-0.543213
H	1.226521	3.569575	-0.179727
H	-1.207253	3.566219	0.146662
H	-3.050813	4.426176	0.537835
H	-7.269057	3.780090	0.967279
H	-6.940859	1.354914	0.717544
H	-5.446658	-0.558902	0.341433
H	6.903374	7.302419	-1.153680
H	7.256585	5.789234	-1.994301
H	7.460574	5.902444	-0.231470
H	3.496228	6.105756	-1.976659
H	4.911557	5.849870	-3.012704
H	4.683380	7.400156	-2.181466
H	3.794020	6.257206	0.574251
H	4.966324	7.559421	0.332817
H	5.415164	6.126725	1.277993
H	-3.649278	6.480995	-0.131342
H	-3.424203	6.326272	1.626658
H	-4.375848	7.659840	0.966257
H	-5.953381	6.080512	-1.162301
H	-6.572911	7.324460	-0.059454
H	-7.348636	5.737414	-0.123763
H	-5.403755	5.683553	3.113681
H	-7.022379	5.504234	2.413556
H	-6.248508	7.093998	2.446680
C	-5.841858	-3.829889	-1.196903
C	-4.480003	-4.029288	-0.975798
C	-3.887934	-5.306146	-1.040832
C	-4.663918	-6.430309	-1.331705
C	-6.020722	-6.238110	-1.548639
C	-6.599910	-4.957995	-1.482010
N	-2.527351	-5.207774	-0.784565
C	-2.333414	-3.927875	-0.574013
N	-3.444571	-3.160287	-0.676441
N	-1.111547	-3.331642	-0.276149
C	0.008955	-4.038391	0.001274
N	0.008916	-5.350055	0.022243
N	1.129804	-3.323567	0.255548
C	2.351497	-3.910526	0.572171
N	3.464729	-3.142252	0.640496

C	4.499204	-4.000830	0.971416
C	3.904589	-5.273078	1.087151
N	2.543514	-5.181848	0.830984
C	5.861980	-3.795881	1.181196
C	6.618413	-4.913714	1.508268
C	6.036703	-6.189148	1.626104
C	4.679088	-6.386802	1.419693
H	-3.484575	-2.157626	-0.416519
H	-1.088333	-2.301895	-0.189690
H	-4.211040	-7.414315	-1.385091
H	-6.650425	-7.091156	-1.777676
H	-7.664374	-4.848163	-1.660008
H	-6.289165	-2.843177	-1.152626
H	0.879835	-5.816138	0.290455
H	-0.862958	-5.824375	-0.227803
H	1.106671	-2.296642	0.140317
H	3.505256	-2.147682	0.351266
H	6.311558	-2.812844	1.096893
H	7.683528	-4.799299	1.679346
H	6.665145	-7.034107	1.886293
H	4.224398	-7.367073	1.512030

AAAA-DDDD, cation, 2CH-N, Leigh

C	4.962479	-1.518749	-0.488337
C	4.070409	-2.235332	0.279966
N	4.763587	-3.087025	1.068141
N	6.037265	-2.933341	0.829554
N	6.164485	-1.994141	-0.093725
N	2.678605	-2.128488	0.280000
C	1.821811	-3.082507	0.687684
N	2.251831	-4.219496	1.206365
C	7.489516	-1.537789	-0.528523
C	8.527115	-2.651557	-0.487850
C	9.882657	-2.150235	-0.982944
C	10.962857	-3.228764	-0.913683
C	12.319936	-2.724914	-1.396848
N	0.509980	-2.828496	0.526884
C	-0.527546	-3.720598	0.823228
C	-1.851001	-3.612221	0.458551
N	-2.403407	-4.718879	0.997767
N	-1.511643	-5.464667	1.630879
N	-0.358948	-4.858298	1.534273
C	-3.796102	-5.151696	0.897793
C	-3.937227	-6.406864	0.043745
C	-5.403178	-6.792020	-0.139564
C	-5.577130	-8.086126	-0.932317
C	-7.045759	-8.441648	-1.148146
N	2.671468	0.756531	-0.069035
C	3.616165	1.633044	0.051155
C	3.424987	3.057682	0.126194
C	2.117893	3.570095	0.068222
C	1.031330	2.604001	-0.044853
C	1.357938	1.203255	-0.105552
C	4.517854	3.933218	0.245968
C	4.306244	5.292224	0.304346
C	3.001077	5.833903	0.243914
C	1.927280	4.960995	0.126732
C	-0.306183	2.938825	-0.085958
C	-1.280603	1.930434	-0.181285
C	-0.852744	0.568435	-0.233515
N	0.456605	0.235721	-0.191193
N	-1.725673	-0.455259	-0.328339
C	-3.021063	-0.182037	-0.372649
C	-3.560797	1.155529	-0.305654
C	-2.662222	2.195768	-0.215731
C	-5.009903	1.328932	-0.333676
C	-5.791315	0.164014	-0.448167
C	-5.123881	-1.111949	-0.529215
N	-3.844755	-1.295227	-0.493842
C	-5.654660	2.568059	-0.246639
C	-7.044369	2.673607	-0.268791
C	-7.803494	1.493169	-0.386097
C	-7.188452	0.258471	-0.474592
C	2.823425	7.351458	0.309697
C	3.402247	7.867033	1.639577
C	-7.691220	4.057402	-0.164905
C	-7.254581	4.723507	1.151511
C	-7.230224	4.918661	-1.353719
C	-9.221362	3.983372	-0.183609
C	3.580376	7.996948	-0.864752
C	1.352038	7.768275	0.226389
H	4.836792	-0.768962	-1.252259
H	2.321197	-1.211157	-0.004071
H	1.564204	-4.892660	1.537245
H	3.243367	-4.318300	1.402307
H	0.246294	-1.928748	0.117309
H	-2.416367	-2.866782	-0.083588
H	-5.746594	-2.002828	-0.625917
H	-7.782247	-0.646990	-0.561700
H	-8.885277	1.541616	-0.405929
H	-5.064739	3.473977	-0.153662
H	-2.994016	3.228414	-0.169852
H	-0.632524	3.973402	-0.040825
H	0.922152	5.360143	0.078445
H	5.158574	5.957660	0.396944
H	5.524171	3.526859	0.291341
H	4.637988	1.253305	0.097316
H	-9.631737	4.993633	-0.101395
H	-9.607163	3.397480	0.656544
H	-9.595069	3.546720	-1.114944
H	-6.170238	4.856752	1.199799
H	-7.563525	4.123210	2.012618
H	-7.717654	5.711440	1.237373
H	-6.144376	5.048936	-1.362619
H	-7.687438	5.911149	-1.290888
H	-7.527369	4.462037	-2.302715
H	0.767798	7.356733	1.055963
H	0.895439	7.450051	-0.716114
H	1.282730	8.858441	0.279297
H	2.881639	7.417111	2.490996
H	3.281873	8.953359	1.697376
H	4.468231	7.642023	1.733009
H	3.192070	7.636888	-1.822076
H	4.651383	7.779871	-0.826244
H	3.456955	9.083984	-0.829452
H	-4.344105	-4.311526	0.462631
H	-4.167148	-5.319207	1.912213
H	-3.470767	-6.229805	-0.932604
H	-3.387471	-7.224372	0.523010
H	-5.879810	-6.903141	0.843474
H	-5.932086	-5.976814	-0.651744
H	-5.072897	-7.986367	-1.902440
H	-5.070897	-8.902017	-0.402332
H	-7.153747	-9.383601	-1.692650
H	-7.565451	-8.544978	-0.190263
H	-7.554874	-7.659900	-1.721314
H	7.366367	-1.155010	-1.545160
H	7.786916	-0.704484	0.116402
H	8.619115	-3.024129	0.536861
H	8.184614	-3.489260	-1.106191
H	9.790121	-1.791992	-2.017162
H	10.195495	-1.286297	-0.380635
H	11.046752	-3.585748	0.120214
H	10.651333	-4.091443	-1.515310
H	13.084360	-3.503154	-1.326165
H	12.265946	-2.398487	-2.440103
H	12.652213	-1.871428	-0.797081

AAAA-DDDD, cation, model

H	3.879317	-4.030777	-1.163620
C	4.118653	-2.991449	-0.918379
N	3.243676	-2.150441	-0.521212
C	1.944153	-2.635450	-0.399577
N	1.007788	-1.815609	-0.101629
C	-0.264470	-2.362978	0.026086
N	-1.259413	-1.580052	0.183405
C	-2.512069	-2.198799	0.335889
N	-3.529256	-1.477792	0.543037
C	-4.809273	-2.138876	0.714065
H	1.767229	-3.708280	-0.561265
H	-0.368537	-3.455998	-0.002289
H	-2.551260	-3.295825	0.273048
N	-3.162259	1.198612	-0.683756
H	-4.059952	1.634505	-0.835890
C	-2.103901	2.047887	-0.608080
N	-0.913429	1.385459	-0.239556
C	0.277340	1.993120	-0.007733
N	1.308994	1.174980	0.327147
C	2.613978	1.598756	0.650277
N	3.448613	0.545963	0.889822
H	4.419332	0.793401	1.017085
N	-2.076325	3.301618	-0.876305
N	0.423476	3.288284	-0.099123
N	2.873670	2.848966	0.747655
H	3.311294	-0.324103	0.376179
H	1.147343	0.164112	0.283676
H	-0.411493	3.807886	-0.401696
H	1.351570	3.653035	0.145851
H	-0.976159	0.370557	-0.090604
H	-3.194206	0.343898	-0.121386
H	5.150691	-2.665831	-1.022742
H	-3.001506	3.654804	-1.103087
H	3.853418	3.011766	0.962128
H	-5.542881	-1.662624	0.059594
H	-4.773791	-3.213143	0.499339
H	-5.145612	-1.990563	1.744003

AAAA-DDDD, neutral, Leigh

C	4.284497	3.202241	-0.766611
C	3.899593	1.850835	-0.761446
C	4.870606	0.865081	-1.001134
C	6.204167	1.235778	-1.243479
C	6.557979	2.566097	-1.241452
C	5.600472	3.578351	-0.999593
C	2.537137	1.395412	-0.510971
C	2.266153	-0.020373	-0.507333
N	3.274324	-0.946833	-0.734895
C	4.473367	-0.517900	-0.966197
C	1.475071	2.238514	-0.264658
C	0.194422	1.707397	-0.034930
C	0.027721	0.288452	-0.064319
N	1.067694	-0.542063	-0.291549
N	-1.180633	-0.286523	0.130232
C	-2.226184	0.491965	0.359810
C	-2.160622	1.930439	0.428789
C	-0.929753	2.512435	0.221862
C	-3.383990	2.679332	0.701191
C	-4.561514	1.933877	0.898288
C	-4.497058	0.499782	0.786728
N	-3.428502	-0.182856	0.531532
C	-5.771211	2.588715	1.162994
C	-5.814080	3.968502	1.225414
C	-4.651834	4.738275	1.024406
C	-3.452436	4.075547	0.768915
C	-4.664953	6.268673	1.069375
C	-6.060910	6.826243	1.365961
C	6.050797	5.039692	-0.998230
C	4.894822	6.004041	-0.715523
C	-3.704486	6.752190	2.169629
C	-4.202378	6.814629	-0.292862
C	7.122576	5.231093	0.089719
C	6.646997	5.383839	-2.374174
H	-5.422201	-0.064509	0.913078
H	-6.673251	2.002285	1.312941
H	-6.760740	4.454615	1.426955
H	-2.553812	4.663323	0.612512
H	-0.799758	3.589754	0.252702
H	1.600603	3.316374	-0.244858
H	3.539689	3.965448	-0.580999
H	7.591912	2.838976	-1.426598
H	6.947265	0.464839	-1.424877
H	5.240027	-1.274608	-1.136902
H	-6.014611	7.918614	1.390119
H	-6.434900	6.486389	2.336782
H	-6.782920	6.540309	0.594692

H	-2.676964	6.427598	1.983035
H	-4.011091	6.370956	3.148281
H	-3.709410	7.845966	2.209193
H	-3.187073	6.488955	-0.536504
H	-4.209961	7.908951	-0.274504
H	-4.869684	6.479178	-1.092456
H	4.448023	5.823183	0.267288
H	4.111098	5.928461	-1.475993
H	5.271036	7.030753	-0.726146
H	6.722101	4.981490	1.077023
H	7.450790	6.275064	0.104933
H	8.001115	4.605565	-0.090994
H	5.901660	5.250765	-3.164105
H	7.512061	4.758441	-2.611663
H	6.975144	6.427953	-2.383135
N	-3.770180	-2.788786	-0.699981
C	-2.768305	-3.715514	-0.765160
N	-3.141687	-4.878604	-1.251705
C	-4.493482	-4.716202	-1.520013
C	-4.907427	-3.409957	-1.180492
C	-5.416713	-5.622215	-2.044497
C	-6.729322	-5.195189	-2.214106
C	-7.124557	-3.892046	-1.872740
C	-6.217977	-2.973914	-1.350236
N	-1.513044	-3.365224	-0.336881
C	-0.461745	-4.272656	-0.148119
N	-0.474842	-5.527225	-0.334512
N	0.670261	-3.579531	0.288827
C	1.812565	-4.180938	0.774316
N	1.901879	-5.272044	1.495310
C	3.256069	-5.389770	1.762876
C	3.971526	-4.312761	1.191836
N	3.011416	-3.555000	0.554791
C	3.942651	-6.366539	2.489002
C	5.320404	-6.238407	2.622175
C	6.014670	-5.160378	2.048405
C	5.350838	-4.176321	1.322769
H	3.134805	-2.702455	-0.000151
H	0.767024	-2.602640	0.005707
H	3.404685	-7.197977	2.933460
H	5.873674	-6.985808	3.181502
H	7.090917	-5.093753	2.172164
H	5.884895	-3.342378	0.878940
H	-1.406749	-5.816249	-0.630019
H	-1.383146	-2.396404	-0.039961
H	-3.682946	-1.863389	-0.262507
H	-6.520299	-1.965324	-1.087935
H	-8.157452	-3.593131	-2.019043
H	-7.465032	-5.882286	-2.619377
H	-5.108293	-6.628555	-2.307877

AAAA-DDDD, neutral, model

C	-5.426139	1.285152	0.678116
N	-4.276971	0.744077	0.557619
C	-3.242415	1.609593	0.202006
N	-2.045896	1.159501	0.177542
C	-1.066994	2.063450	-0.221635
N	0.163205	1.739872	-0.128470
C	1.076648	2.706442	-0.578308
N	2.314846	2.530112	-0.392804
C	3.220199	3.557715	-0.869567
H	-5.603948	2.356192	0.531093
H	-3.494487	2.651079	-0.050567
H	-1.393191	3.036829	-0.616671
H	0.671094	3.594629	-1.086974
H	-6.276503	0.661771	0.943798
H	3.962794	3.098026	-1.526651
H	2.713793	4.368299	-1.407442
H	3.757557	3.977575	-0.014577
N	3.475098	0.254843	1.286438
C	3.083560	-0.981077	0.776507
N	3.759305	-2.066474	0.821087
N	1.788167	-0.906495	0.269553
C	1.007848	-1.944162	-0.250611
N	1.485891	-3.002015	-0.758501
N	-0.357709	-1.603221	-0.131831
C	-1.429104	-2.439086	-0.416585
N	-1.268969	-3.542800	-1.054869
N	-2.618363	-1.977359	0.110899
H	4.472522	0.321212	1.435526
H	-3.447551	-2.354059	-0.324863
H	-2.693673	-0.987374	0.313358
H	-0.582382	-0.749994	0.365595
H	0.704361	-3.596347	-1.041064
H	1.324689	-0.008839	0.370324
H	3.106986	1.063598	0.783919
H	4.666213	-1.862435	1.241518
H	-2.160116	-4.028609	-1.132492

ADA-DAD

H	-0.771790	-0.606447	0.109400
N	-0.027664	-1.325076	0.135852
C	1.205731	-0.890897	0.546069
N	2.195912	-1.851576	0.580359
C	1.941095	-3.140468	0.187202
C	0.725923	-3.558457	-0.223359
C	-0.367525	-2.611250	-0.260271
O	1.416645	0.275233	0.865843
C	3.549764	-1.422548	0.959482
H	2.795357	-3.806891	0.235603
H	0.544125	-4.581106	-0.520064
O	-1.519667	-2.862134	-0.604850
H	4.073619	-2.305272	1.334925
C	4.295041	-0.793035	-0.211843
H	3.446528	-0.709284	1.779476
C	5.695574	-0.333092	0.185837
H	4.358120	-1.520504	-1.031000
H	3.711656	0.060169	-0.577558
C	6.444964	0.324267	-0.971964
H	5.623742	0.376801	1.020929
H	6.274769	-1.190553	0.554866
C	7.847692	0.778799	-0.577629
H	6.505013	-0.382374	-1.809212
H	5.863471	1.182207	-1.331602
H	8.363862	1.254802	-1.415262
H	7.806719	1.499119	0.245679
H	8.454531	-0.070316	-0.247403
N	-2.193303	0.779603	0.009467
C	-1.886658	2.085828	0.036512
C	-2.852848	3.093370	-0.037595
C	-4.177428	2.693489	-0.138138
C	-4.523954	1.350417	-0.165912
C	-3.482313	0.419934	-0.091744
N	-0.520346	2.348231	0.168247
H	-2.559772	4.130463	-0.014556
H	-4.957780	3.445737	-0.197088
H	-5.545896	1.016657	-0.243896
N	-3.686374	-0.961596	-0.139280
H	-2.850203	-1.535200	-0.263386
C	-4.884236	-1.631421	-0.075391
C	0.115892	3.558808	0.040562
H	0.068229	1.545699	0.390446
O	-5.969090	-1.086227	0.053400
C	-4.765100	-3.136063	-0.179675
O	-0.450639	4.607568	-0.224233
C	1.612295	3.503608	0.256828
H	-3.734135	-3.486394	-0.215483
H	-5.280793	-3.578413	0.674767
H	-5.290100	-3.456105	-1.082796
H	1.988247	2.494668	0.423880
H	2.098373	3.942423	-0.616634
H	1.855063	4.129767	1.118362

ADDA-DAAD, Yosuke

N	-3.456081	-0.143760	0.642792
C	-2.340339	0.612900	0.556312
C	-2.686303	1.927547	0.797579
N	-4.021916	1.859624	1.022767
N	-4.475724	0.623034	0.925784
N	-1.128056	0.006812	0.273323
C	-0.016699	0.753860	-0.011730
O	-0.016439	1.980415	-0.016207
C	-4.944810	2.954524	1.305006
C	-5.067818	3.923528	0.132858
C	-6.132026	4.984322	0.404737
C	-6.219361	6.031509	-0.704238
C	-7.327573	7.055254	-0.468706
C	-7.372411	8.127951	-1.552844
N	1.094063	0.004199	-0.292481
C	2.305075	0.607962	-0.585621
N	3.420258	-0.149275	-0.675388
N	4.437521	0.615087	-0.973677
N	3.982589	1.850483	-1.077451
C	2.649145	1.920924	-0.839841
C	4.904697	2.946837	-1.355617
C	5.146623	3.817912	-0.127149
C	6.185453	4.900364	-0.410467
C	6.408838	5.828643	0.781534
C	7.477644	6.887499	0.520527
C	7.673810	7.825548	1.708721
N	3.439133	-2.974404	0.063495
C	4.749427	-3.362813	0.264027
O	5.096117	-4.493728	0.560482
C	2.293867	-3.756651	0.090455
N	1.145395	-3.092231	0.032058
C	-0.015374	-3.804876	0.015378
C	-0.014430	-5.222703	0.028615
C	1.225659	-5.892788	0.100601
C	2.385411	-5.178613	0.143916
N	-1.177016	-3.094325	-0.014500
C	-2.324717	-3.761103	-0.060995
C	-2.414345	-5.183985	-0.088011
C	-1.253691	-5.895652	-0.031377
N	-3.470867	-2.980014	-0.048694
C	-4.780653	-3.373332	-0.243208
O	-5.125698	-4.508903	-0.523097
C	5.740453	-2.227466	0.085287
C	7.182977	-2.686100	0.239815
C	8.179791	-1.545421	0.051923
C	9.628262	-2.013216	0.169359
C	10.648844	-0.893466	-0.018460
C	12.091506	-1.382043	0.088144
C	13.124550	-0.273822	-0.100769
C	14.563301	-0.774666	0.002784
C	15.603104	0.327127	-0.185365
C	17.040229	-0.179450	-0.083221
C	18.070841	0.930632	-0.271090
C	-5.773127	-2.236823	-0.079966
C	-7.214829	-2.696499	-0.238508
C	-8.212036	-1.554852	-0.058847
C	-9.660419	-2.020898	-0.184511
C	-10.680106	-0.899395	-0.002682
C	-12.123283	-1.384837	-0.116920
C	-13.154521	-0.274053	0.066466
C	-14.594089	-0.771457	-0.041202
C	-15.631468	0.333472	0.141925
C	-17.069770	-0.169505	0.038315
C	-18.097526	0.943989	0.221438
H	3.314155	-1.982452	-0.169973
H	1.081408	-1.018021	-0.198825
H	-1.114797	-1.016291	0.191648
H	-3.346902	-1.983862	0.167345
H	-1.268260	-6.981719	-0.028581
H	1.241618	-6.978860	0.117946
H	-5.612639	-1.773643	0.901234
H	-5.525246	-1.453842	-0.808117
H	5.574516	-1.772062	-0.898531
H	5.496570	-1.438770	0.808683
H	-7.345565	-3.153267	-1.225955
H	-7.422730	-3.486804	0.492210
H	-8.062278	-1.088578	0.924602
H	-8.013099	-0.771702	-0.803220
H	-9.806451	-2.487976	-1.168079
H	-9.850887	-2.806770	0.559203
H	-10.531656	-0.427894	0.978527

H	-10.498870	-0.115950	-0.751332
H	-12.270118	-1.857604	-1.097698
H	-12.300053	-2.170012	0.631149
H	-13.006633	0.201228	1.045955
H	-12.983312	0.509406	-0.684450
H	-14.740875	-1.247980	-1.020265
H	-14.763008	-1.555413	0.709826
H	-15.484614	0.812261	1.120008
H	-15.466475	1.116760	-0.610876
H	-17.213344	-0.649129	-0.937989
H	-17.232074	-0.950630	0.791692
H	-19.120162	0.565237	0.146332
H	-17.985545	1.418902	1.201406
H	-17.969577	1.720358	-0.539529
H	7.317694	-3.139349	1.228277
H	7.387934	-3.479056	-0.488909
H	8.024451	-1.080452	-0.931295
H	7.986267	-0.761090	0.796412
H	9.779162	-2.480284	1.152018
H	9.813419	-2.799605	-0.575170
H	10.496012	-0.422152	-0.998999
H	10.473067	-0.109551	0.730850
H	12.242222	-1.854732	1.068307
H	12.262802	-2.167882	-0.660384
H	12.974314	0.200450	-1.080371
H	12.957928	0.511076	0.649768
H	14.711931	-1.250127	0.981897
H	14.727636	-1.560235	-0.747624
H	15.455599	0.803961	-1.164331
H	15.441685	1.112645	0.565824
H	17.184234	-0.656974	0.894078
H	17.199039	-0.963027	-0.834779
H	19.092563	0.549313	-0.196365
H	17.958805	1.403031	-1.252246
H	17.946009	1.709548	0.487789
H	-5.902748	2.477285	1.523547
H	-4.599603	3.469704	2.206501
H	5.830385	2.475050	-1.692728
H	4.493213	3.534054	-2.181682
H	-4.099096	4.404967	-0.048465
H	-5.322998	3.358036	-0.771088
H	-7.107963	4.494270	0.523411
H	-5.917341	5.484419	1.359261
H	-5.254512	6.551395	-0.788372
H	-6.386214	5.531687	-1.668645
H	-8.292557	6.536186	-0.421380
H	-7.181906	7.525243	0.511611
H	-8.175430	8.847656	-1.373157
H	-6.427677	8.679931	-1.592046
H	-7.536134	7.679445	-2.538062
H	4.201320	4.278709	0.184940
H	5.482814	3.179376	0.698560
H	7.136698	4.426159	-0.687454
H	5.869724	5.494298	-1.279184
H	5.461855	6.322680	1.038962
H	6.695174	5.232887	1.659089
H	8.425755	6.390063	0.280796
H	7.199919	7.468152	-0.368232
H	8.442889	8.575687	1.507392
H	6.743936	8.352427	1.945299
H	7.974950	7.266831	2.600749
H	3.354568	-5.647133	0.204020
H	-3.382718	-5.654772	-0.140058
H	2.080084	2.832567	-0.865022
H	-2.115876	2.838510	0.820468

ADDA-DAAD, yosuke, model

C	0.157842	-2.889723	-1.770070
C	0.009538	-1.912809	-0.863781
N	1.084422	-1.341394	-0.156527
C	1.908991	-2.119387	0.427842
N	3.036934	-1.614191	1.025043
N	-1.193291	-1.226280	-0.601652
C	-2.270433	-1.911959	-0.594432
N	-3.486380	-1.304566	-0.414041
N	-3.160586	1.416835	0.801077
C	-2.132964	2.411755	0.622749
N	-0.966601	1.784761	0.185089
C	0.209927	2.453706	-0.082351
O	0.312747	3.671291	-0.064121
N	1.256201	1.604566	-0.361438
C	2.541708	2.030483	-0.688815
N	3.461382	0.935804	-0.507834
H	1.789359	-3.202363	0.500473
H	-2.316302	-2.995356	-0.720431
C	-4.664471	-2.016557	-0.406060
C	3.904510	-2.404610	1.743835
C	-2.389512	3.710373	0.810397
N	-3.972854	1.670927	1.699514
N	4.419127	0.929418	-1.292259
H	-3.476845	-0.297817	-0.228914
H	-1.023169	0.774761	0.024760
H	1.138695	0.589710	-0.250472
H	3.245340	-0.629296	0.846964
C	2.972209	3.249153	-1.029810
H	1.134359	-3.314507	-1.966273
H	-0.677672	-3.229729	-2.369364
O	-4.734920	-3.213954	-0.574420
H	-5.542978	-1.376749	-0.234386
O	3.731896	-3.585592	1.949652
H	4.776150	-1.844450	2.113262
H	-4.670684	0.910879	1.695958
H	5.012715	0.129714	-1.022857
H	-1.646200	4.467854	0.625303
H	-3.381865	3.981358	1.145642
H	4.031821	3.381337	-1.204904
H	2.296935	4.086971	-1.099474

Coordinates of Base Pairs

AA1

N	0.138260	1.941355	0.000106
C	0.806830	0.771262	0.000103
C	0.250573	-0.517053	-0.000278
C	-1.164922	-0.600050	-0.000398
N	-1.859417	0.571439	-0.000494
C	-1.186007	1.738560	-0.000272
N	1.234723	-1.490862	-0.000435
C	2.363615	-0.802405	-0.000094
N	2.167963	0.561347	0.000290
N	-1.831615	-1.763199	-0.000499
H	-1.811096	2.636502	-0.000406
H	-2.876145	-1.788372	-0.000243
H	-1.296130	-2.625585	0.000429
H	3.363010	-1.231058	-0.000174
H	2.889011	1.278652	0.000701
N	-4.740101	-1.784098	-0.000213
C	-5.426902	-0.607409	-0.000834
C	-6.843081	-0.679600	-0.001322
C	-7.409057	-1.963516	-0.001521
N	-6.749387	-3.138605	-0.001073
C	-5.423495	-2.945812	-0.000352
N	-4.753380	0.551539	-0.000991
N	-7.819832	0.301577	-0.002013
N	-8.768593	-1.743392	-0.002329
H	-4.806559	-3.849499	0.000217
H	-5.283921	1.417255	-0.001268
H	-3.708002	0.570566	-0.000815
H	-9.494060	-2.456205	-0.002977
C	-8.954078	-0.378128	-0.002589
H	-9.949537	0.059628	-0.003287

AA2

C	-9.055601	-0.686466	-0.002349
N	-7.951229	0.042731	-0.002129
C	-6.919242	-0.884287	-0.001579
C	-7.432418	-2.192079	-0.001420
N	-8.802696	-2.034622	-0.001981
N	-6.731873	-3.338947	-0.000980
C	-5.410349	-3.092765	-0.000693
N	-4.772239	-1.910581	-0.000846
C	-5.503998	-0.763208	-0.001341
N	-4.858261	0.413759	-0.001676
N	-5.785265	3.163099	-0.002195
C	-7.020926	3.732934	-0.002300
C	-7.086886	5.149173	-0.002430
C	-5.863559	5.836873	-0.002701
N	-4.630607	5.293832	-0.002651
C	-4.695578	3.955962	-0.002351
N	-8.157856	6.026409	-0.002448
C	-7.590864	7.221218	-0.002794
N	-6.214100	7.168461	-0.002963
N	-8.108705	2.949299	-0.002223
H	-3.736098	3.429275	-0.002273
H	-8.022532	1.912266	-0.002167
H	-9.023153	3.389673	-0.003506
H	-8.121506	8.170460	-0.002790
H	-5.574844	7.959492	-0.003352
H	-4.762060	-3.974266	-0.000283
H	-5.338997	1.337473	-0.001898
H	-3.842470	0.384848	-0.000891
H	-9.492474	-2.781993	-0.002009
H	-10.069341	-0.293823	-0.003010

AA3

N	0.035508	2.163336	-0.000172
C	0.785584	1.048272	-0.000027
C	0.328152	-0.279945	0.000097
C	-1.079338	-0.459901	0.000071
N	-1.861389	0.652077	-0.000047
C	-1.273961	1.861039	-0.000158
N	1.395050	-1.165141	0.000228
C	2.467649	-0.389909	0.000188
N	2.160927	0.947483	0.000015
N	-1.668241	-1.667145	0.000244
H	-1.959348	2.713719	-0.000256
H	-2.683731	-1.693532	-0.000227
H	-1.138796	-2.556899	0.000092
H	3.497148	-0.739698	0.000272
H	2.820160	1.721986	0.000050
H	1.825560	-3.036014	0.000136
N	2.353968	-3.926527	0.000050
H	3.369459	-3.901473	-0.000449
C	1.765088	-5.133804	-0.000197
N	2.547369	-6.245750	-0.000337
C	1.960432	-7.455042	-0.000446
N	0.651091	-7.757559	-0.000421
C	-0.099076	-6.642460	-0.000305
C	0.357883	-5.314018	-0.000236
H	2.646128	-8.307789	-0.000521
N	-1.474390	-6.541925	-0.000267
N	-0.709049	-4.429044	-0.000160
C	-1.781386	-5.204663	-0.000156
H	-2.133704	-7.316389	-0.000088
H	-2.810914	-4.855044	-0.000118

AC1

N	0.082723	1.936678	0.000155
C	0.775228	0.780325	0.000179
C	0.246989	-0.519573	0.000084
C	-1.167518	-0.634490	-0.000033
N	-1.886584	0.525228	-0.000167
C	-1.236666	1.705047	-0.000047
N	1.252638	-1.471163	-0.000038
C	2.366412	-0.758476	-0.000004
N	2.140500	0.600777	0.000181
N	-1.811359	-1.807430	-0.000085
H	-1.879570	2.590457	-0.000123
H	-2.857171	-1.845760	-0.000259
H	-1.266802	-2.664297	0.000551
H	3.374985	-1.164936	-0.000170

H	2.844159	1.335039	0.000126
N	-4.688789	-1.795211	-0.000863
C	-5.431436	-0.673432	-0.001316
C	-6.870365	-0.711069	-0.001583
C	-7.456586	-1.941533	-0.001544
N	-6.689994	-3.065428	-0.001227
C	-5.273405	-3.028808	-0.000653
N	-4.781627	0.501603	-0.001531
H	-7.465152	0.200020	-0.001855
H	-8.536493	-2.089497	-0.001777
H	-7.121474	-3.987373	-0.001022
O	-4.641761	-4.094396	-0.000048
H	-5.311298	1.366529	-0.001819
H	-3.734077	0.527243	-0.001150

AC2

N	0.041909	2.109639	-0.000264
C	0.776292	0.983637	-0.000165
C	0.302610	-0.338260	-0.000094
C	-1.110059	-0.504214	-0.000019
N	-1.875206	0.622773	-0.000077
C	-1.272045	1.822408	-0.000215
N	1.362556	-1.233938	-0.000008
C	2.444784	-0.472128	-0.000012
N	2.151389	0.867616	-0.000133
N	-1.712307	-1.700296	0.000148
H	-1.946356	2.684309	-0.000287
H	-2.728232	-1.729634	0.000400
H	-1.199146	-2.605118	0.000376
H	3.469704	-0.834548	-0.000122
H	2.818381	1.635309	0.000064
N	-0.649443	-4.392940	0.000576
C	0.573412	-4.950970	0.000421
C	0.764793	-6.377771	0.000033
C	-0.357060	-7.152040	0.000107
N	-1.587522	-6.571832	0.000423
C	-1.774884	-5.167378	0.000418
N	1.634055	-4.126802	0.000717
H	1.759087	-6.820160	-0.000202
H	-0.333331	-8.241907	-0.000051
H	-2.429771	-7.143137	0.000336
O	-2.927576	-4.712220	0.000327
H	2.570640	-4.515373	0.000091
H	1.502103	-3.092327	0.000568

AT (H)

N	0.172672	1.948752	0.000124
C	0.770057	0.744909	0.000010
C	0.137043	-0.507473	0.000026
C	-1.281027	-0.504906	0.000114
N	-1.909314	0.700012	0.000284
C	-1.165850	1.820548	0.000282
N	1.077384	-1.522753	-0.000120
C	2.249044	-0.907023	-0.000206
N	2.121045	0.457842	-0.000197
N	-2.017258	-1.629011	0.000067
H	-1.733049	2.756120	0.000410
H	-3.028874	-1.538847	-0.000231
H	-1.596467	-2.569693	0.000009
H	3.211103	-1.414165	-0.000379
H	2.879109	1.135847	-0.000038
H	1.025326	-3.243661	-0.000312
N	1.087233	-4.306191	-0.000352
C	2.365231	-4.825463	-0.000198
N	2.398090	-6.215462	-0.000143
C	1.269654	-6.997876	-0.000231
C	0.012054	-6.472803	-0.000371
C	-0.110649	-5.018204	-0.000400
O	3.386522	-4.133167	-0.000101
H	3.320348	-6.645114	-0.000105
H	1.454079	-8.072856	-0.000159
C	-1.225707	-7.326537	-0.000411
O	-1.195129	-4.402743	-0.000462
H	-2.115012	-6.682508	-0.000952
H	-1.268938	-7.973209	-0.890453
H	-1.269514	-7.972465	0.890146

AT (RH)

N	0.151448	1.908437	-0.000276
C	0.749493	0.705021	-0.000150
C	0.116884	-0.547690	0.000187
C	-1.300787	-0.545112	0.000231
N	-1.929861	0.658890	0.000141
C	-1.186865	1.779998	-0.000072
N	1.057570	-1.562371	0.000221
C	2.229074	-0.946469	-0.000096
N	2.100468	0.418404	-0.000383
N	-2.037905	-1.669586	0.000393
H	-1.753999	2.715612	-0.000090
H	-3.049485	-1.580272	0.000009
H	-1.618956	-2.608776	0.000264
H	3.190411	-1.455122	-0.000264
H	2.858147	1.096882	-0.000422
H	1.027419	-3.282403	0.000338
N	1.088387	-4.344853	0.000191
C	2.376485	-4.898919	0.000128
C	2.429482	-6.360021	-0.000314
C	1.252108	-7.041877	-0.000700
N	0.031942	-6.404028	-0.000628
C	-0.103327	-5.025480	-0.000184
O	3.368682	-4.156642	0.000420
C	3.767400	-7.032238	-0.000311
H	1.205488	-8.131197	-0.001069
H	-0.829885	-6.944405	-0.000844
O	-1.214419	-4.472017	-0.000172
H	3.656872	-8.125586	-0.000327
H	4.353727	-6.733548	-0.883930
H	4.353704	-6.733533	0.883312

AT (RWC)

N	-0.141568	1.887058	0.000350
C	0.633884	0.785364	0.000329
C	0.205764	-0.552537	-0.000014
C	-1.194153	-0.767911	-0.000364
N	-1.991435	0.333187	-0.000391
C	-1.440582	1.564356	-0.000050
N	1.279842	-1.425346	-0.000114
C	2.336025	-0.629967	0.000170
N	2.008299	0.708041	0.000486
N	-1.757967	-1.985846	-0.000874
H	-2.162083	2.387136	-0.000134
H	-2.784472	-2.085792	-0.000360
H	-1.163073	-2.807527	0.000424
H	3.372254	-0.959561	0.000399
H	2.654537	1.493417	0.000471
H	-3.710203	0.249685	-0.000414
N	-4.779300	0.199397	-0.000313
C	-5.328602	-1.057195	0.000005
N	-6.712953	-1.072426	0.000268
C	-7.478266	0.071326	0.000395
C	-6.931037	1.317396	0.000137
C	-5.469602	1.421830	-0.000296
O	-4.661308	-2.105635	0.000128
H	-7.156876	-1.987763	0.000557
H	-8.555694	-0.097491	0.000749
C	-7.761344	2.570922	0.000351
O	-4.831789	2.483424	-0.000615
H	-7.099218	3.446941	-0.000554
H	-8.407390	2.626970	-0.889499
H	-8.405867	2.627673	0.891266

AT (WC)

C	0.151985	1.935561	0.000130
N	0.710425	0.672246	0.000542
C	0.026535	-0.540826	0.000454
C	-1.428944	-0.458181	-0.000034
C	-1.992833	0.781336	-0.000400
N	-1.239447	1.929759	-0.000278
O	0.657107	-1.617810	0.000755
H	-1.692664	2.840590	-0.000530
O	0.813033	2.976077	0.000079
N	3.531858	-1.728611	-0.000200
C	4.199114	-0.565209	0.000134
C	5.612399	-0.469616	0.000324
C	6.153005	0.826750	0.000408
N	5.474470	1.990932	0.000392
C	4.152543	1.779956	0.000312
N	3.498542	0.600799	0.000179
N	6.608213	-1.431024	0.000167
C	7.728434	-0.728722	0.000172
N	7.516008	0.632589	0.000386
H	3.505268	2.662084	0.000371
H	2.497763	-1.737159	-0.000052
H	4.053711	-2.598648	0.000311
H	8.732824	-1.145445	0.000075
H	8.226939	1.360093	0.000405
H	1.781032	0.636890	0.000528
C	-2.227355	-1.725341	-0.000179
H	-3.072852	0.932369	-0.000820
H	-3.304800	-1.508494	-0.000348
H	-1.987611	-2.337491	-0.883932
H	-1.987863	-2.337550	0.883597

CC

C	-1.035454	1.253460	-0.000213
C	0.225384	1.770504	-0.000122
N	1.305994	0.942589	0.000137
C	1.190254	-0.469155	0.000062
N	-0.072032	-0.985031	0.000109
C	-1.154643	-0.182259	0.000053
O	2.220383	-1.159585	-0.000019
N	-2.360200	-0.767674	0.000268
H	2.250137	1.322291	0.000022
H	-2.440055	-1.813921	0.000420
H	-3.199470	-0.197972	-0.000101
H	-1.912248	1.897715	-0.000380
H	0.434029	2.840574	-0.000213
N	-2.471084	-3.638776	0.000894
C	-1.388289	-4.441340	0.001173
C	-1.506843	-5.876768	0.001258
C	-2.767296	-6.394490	0.001267
N	-3.848417	-5.567140	0.001138
C	-3.733280	-4.155221	0.000741
N	-0.182607	-3.856364	0.001435
H	-0.629287	-6.519928	0.001401
H	-2.974879	-7.464618	0.001402
H	-4.792350	-5.947314	0.000999
O	-4.763662	-3.465395	0.000314
H	0.656337	-4.426490	0.001103
H	-0.102659	-2.810289	0.000988

GA1

N	-0.681627	-1.419846	-0.163641
C	0.547046	-0.877436	-0.005349
C	0.898650	0.477589	0.116851
C	-0.149179	1.451761	0.097032
N	-1.415510	0.846493	-0.036099
C	-1.639516	-0.501018	-0.169369
N	2.269769	0.625454	0.240268
C	2.735944	-0.606851	0.195783
N	1.739923	-1.554222	0.047412
O	-0.054795	2.693070	0.180882
N	-2.949617	-0.889760	-0.262973
H	-2.258277	1.488228	0.030657
H	-3.610920	-0.219370	-0.645940
H	-3.093278	-1.851358	-0.559827
H	3.782897	-0.891723	0.264601
H	1.860008	-2.562360	-0.013644
N	-3.705406	2.519706	0.163073
C	-3.541420	3.852233	-0.076762
C	-4.706575	4.659342	-0.071745
C	-5.923334	4.011847	0.193500
N	-6.103612	2.699897	0.445248
C	-4.941647	2.038967	0.410469

N	-2.313176	4.334645	-0.311048
N	-4.890674	6.016925	-0.264189
N	-6.865767	5.013629	0.154114
H	-4.993900	0.965957	0.621162
H	-2.202641	5.338148	-0.420888
H	-1.473111	3.743564	-0.156572
H	-7.865526	4.906805	0.308254
C	-6.194533	6.184902	-0.121481
H	-6.720246	7.133119	-0.204735

GA2

H	-2.754191	-2.094546	0.003573
N	-2.647178	-1.056534	0.007297
H	-3.474872	-0.471430	0.001818
C	-1.422282	-0.500133	0.003756
N	-1.339782	0.877409	-0.001290
H	-2.202869	1.421279	-0.004661
C	-0.149676	1.666393	-0.003763
O	-0.231109	2.897737	-0.008192
C	1.007083	0.819294	-0.000796
N	2.353369	1.142377	-0.002590
C	2.970458	-0.020767	0.000915
H	4.047253	-0.170930	0.000759
N	2.093207	-1.092151	0.004871
H	2.346961	-2.076389	0.009041
C	0.821968	-0.572107	0.003809
N	-0.335316	-1.275459	0.006368
H	-0.267387	-3.207455	-0.002091
N	0.005018	-4.206445	-0.006979
H	0.989464	-4.458154	-0.005956
C	-0.880875	-5.218297	-0.005039
C	-2.286830	-5.029616	-0.003149
N	-3.092437	-3.901540	-0.002302
C	-4.329187	-4.373505	-0.000834
H	-5.233339	-3.770063	0.000046
N	-4.374518	-5.743881	-0.000759
H	-5.210356	-6.323554	0.000447
C	-3.071231	-6.195215	-0.002196
N	-2.634454	-7.465454	-0.002668
C	-1.291407	-7.511195	-0.004391
H	-0.848153	-8.511459	-0.004837
N	-0.413121	-6.493719	-0.005613

GA3

N	-0.607759	-1.505726	-0.284245
C	0.579623	-0.910322	-0.025347
C	0.849678	0.451753	0.191055
C	-0.247409	1.371055	0.164479
N	-1.467686	0.708038	-0.076583
C	-1.611620	-0.638927	-0.293994
N	2.201757	0.662118	0.400869
C	2.736177	-0.540229	0.315626
N	1.802723	-1.526972	0.058748
O	-0.230466	2.607103	0.322715
N	-2.895503	-1.085899	-0.481219
H	-2.344598	1.291834	-0.001093
H	-3.556296	-0.426937	-0.886243
H	-2.965904	-2.033432	-0.843721
H	3.791757	-0.774520	0.429184
H	1.980913	-2.522397	-0.050032
H	-1.557238	3.773925	-0.143824
N	-2.143540	4.590462	-0.388033
C	-3.476702	4.602818	-0.223076
H	-1.682949	5.489191	-0.505412
N	-4.140382	5.784639	-0.329659
C	-5.470754	5.806730	-0.147199
N	-6.299830	4.790280	0.148961
C	-5.625206	3.632424	0.233378
C	-4.246590	3.439619	0.046902
H	-5.940441	6.789870	-0.247106
N	-6.124660	2.377187	0.516541
N	-3.921703	2.100522	0.205465
C	-5.070472	1.503835	0.487843
H	-7.096481	2.148328	0.711360
H	-5.194674	0.442512	0.689654

GA4

N	-0.600969	-1.378428	0.003701
C	0.599787	-0.754064	0.001896
C	0.875674	0.622213	-0.000928
C	-0.223330	1.543341	-0.002355
N	-1.463318	0.834318	-0.001014
C	-1.637145	-0.534803	0.002510
N	2.240896	0.854846	-0.002111
C	2.779096	-0.347161	-0.000106
N	1.832671	-1.357890	0.002449
O	-0.223978	2.777386	-0.004531
N	-2.894078	-1.013128	0.006222
H	-2.289101	1.433308	-0.001750
H	-3.682561	-0.376114	-0.000212
H	-3.064816	-2.047871	0.003423
H	3.843533	-0.568302	-0.000510
H	2.016131	-2.357997	0.004564
N	-3.373746	-3.845466	-0.001066
C	-2.339438	-4.729941	-0.002180
C	-2.661185	-6.109951	-0.002100
C	-4.026000	-6.438323	-0.000980
N	-5.064129	-5.579634	0.000073
C	-4.639685	-4.309928	0.000009
N	-1.078368	-4.271190	-0.003463
N	-1.869666	-7.244818	-0.002671
N	-4.050981	-7.814700	-0.000971
H	-5.418467	-3.541474	0.000973
H	-0.319221	-4.944801	-0.003514
H	-0.881093	-3.250718	-0.001387
H	-4.881264	-8.402283	-0.000064
C	-2.740565	-8.239994	-0.001970
H	-2.488438	-9.297754	-0.002068

GC

N	-0.626935	-1.483366	-0.000097
C	0.589681	-0.902630	0.000165
C	0.913226	0.465875	-0.000324
C	-0.159330	1.408517	-0.002869
N	-1.414683	0.774278	-0.003713
C	-1.621142	-0.591068	-0.002179
N	2.286770	0.653637	-0.000133
C	2.782404	-0.567197	0.000479
N	1.804014	-1.545878	0.000734
O	-0.084591	2.657902	-0.004665
N	-2.902139	-1.010545	-0.005276
H	-2.255146	1.411270	-0.005925
H	-3.704560	-0.356282	0.004983
H	-3.065446	-2.011135	0.004617
H	3.839295	-0.822927	0.000610
H	1.947730	-2.552417	0.000469
N	-3.692444	2.536406	-0.008399
C	-3.539678	3.878428	-0.020257
C	-4.670021	4.768688	-0.020639
C	-5.907932	4.201669	-0.007354
N	-6.039042	2.845851	0.004160
C	-4.931558	1.978945	0.004418
N	-2.292586	4.362170	-0.031519
H	-4.542024	5.849186	-0.029773
H	-6.832434	4.778130	-0.004724
H	-6.960456	2.413244	0.016477
O	-5.137887	0.747493	0.016524
H	-1.470128	3.718676	-0.024024
H	-2.141559	5.365246	-0.040225

GC1

N	-0.553705	-1.482799	-0.001226
C	0.654079	-0.876374	-0.000237
C	0.952318	0.495264	-0.000172
C	-0.135007	1.433822	-0.000820
N	-1.385710	0.756037	-0.001884
C	-1.562200	-0.609387	-0.002479
N	2.322315	0.705770	0.000644
C	2.840996	-0.506184	0.001083
N	1.879174	-1.500653	0.000617
O	-0.097621	2.670233	-0.000506
N	-2.836601	-1.065399	-0.006201
H	-2.230662	1.364698	-0.001293
H	-3.661620	-0.441739	-0.000642
H	-2.961606	-2.071521	0.000411
H	3.902175	-0.743046	0.001626
H	2.038805	-2.504772	0.000686
O	-3.722388	2.380787	0.002499
C	-4.898467	1.976271	0.001894
N	-5.938793	2.925181	0.000347
C	-7.252055	2.575157	-0.001373
C	-7.609085	1.258671	-0.001383
C	-6.541033	0.300349	0.000832
N	-5.247571	0.657550	0.002641
H	-7.973153	3.392101	-0.002665
H	-8.653345	0.953970	-0.002794
N	-6.826164	-1.016901	0.001333
H	-5.661617	3.905288	-0.000070
H	-6.075543	-1.699824	0.002844
H	-7.784148	-1.347388	-0.001093

GC2

N	-0.608648	-1.381703	0.001278
C	0.598986	-0.771672	0.000581
C	0.892366	0.601040	-0.000264
C	-0.195292	1.535735	-0.000916
N	-1.443533	0.842657	-0.000579
C	-1.636625	-0.524765	0.000920
N	2.260612	0.816863	-0.000666
C	2.784183	-0.391333	-0.000197
N	1.825006	-1.390500	0.000571
O	-0.178911	2.769752	-0.001780
N	-2.896919	-0.987132	0.002845
H	-2.262445	1.451011	-0.000787
H	-3.681019	-0.344439	-0.001023
H	-3.080981	-2.020595	0.001717
H	3.845638	-0.626113	-0.000282
H	1.996468	-2.392568	0.000768
N	-3.357476	-3.805516	0.000534
C	-2.371869	-4.719917	0.000635
C	-2.640220	-6.133109	0.000255
C	-3.949834	-6.513036	0.000216
N	-4.934532	-5.574923	0.000425
C	-4.671171	-4.182915	0.000245
N	-1.105386	-4.269436	0.001081
H	-1.836689	-6.866808	0.000076
H	-4.270685	-7.554771	0.000044
H	-5.913926	-5.852062	0.000210
O	-5.619224	-3.386857	-0.000102
H	-0.338931	-4.933167	0.001098
H	-0.908038	-3.246694	0.001224

GG1

C	-1.064611	-1.468519	0.008267
C	0.258681	-0.938059	0.020257
N	0.256721	0.464322	0.025757
C	-0.862764	1.263029	0.018821
N	-2.105967	0.790907	-0.000457
C	-2.139396	-0.558682	-0.001817
O	1.349543	-1.559877	0.022542
N	-0.639888	2.601932	0.074378
N	4.587906	-2.252055	-0.098386
C	4.809105	-0.913226	-0.033407
N	6.051549	-0.439139	-0.020130
C	6.083354	0.910414	-0.013408
C	5.007791	1.819265	-0.014031
C	3.685303	1.286841	-0.015923
N	3.688695	-0.115711	-0.025675
N	5.461618	3.127807	-0.006968
C	6.774497	3.016158	-0.002295
N	7.204589	1.701572	-0.004632
O	2.593816	1.907455	-0.006820
H	2.753975	-0.602564	-0.021232
H	3.656687	-2.618045	0.082413
H	5.377777	-2.864334	0.076618

H	7.480831	3.842626	0.002243
H	8.167632	1.374270	-0.004322
H	1.191906	0.950684	0.029899
N	-3.261172	-1.349326	-0.016615
N	-1.519296	-2.776624	-0.000012
H	0.293082	2.963828	-0.105094
H	-1.427078	3.213562	-0.114191
C	-2.831957	-2.664322	-0.014067
H	-4.224049	-1.021737	-0.026769
H	-3.538009	-3.491175	-0.021789

GG3

N	-0.633536	-1.464628	0.001445
C	0.585614	-0.882576	0.001573
C	0.915284	0.483938	0.000625
C	-0.154806	1.439698	-0.002861
N	-1.413140	0.789860	-0.003234
C	-1.623092	-0.567900	-0.001471
N	2.289970	0.663548	0.000136
C	2.780877	-0.559952	0.000837
N	1.797316	-1.532492	0.001812
O	-0.099873	2.680744	-0.006056
N	-2.908103	-0.993499	-0.004994
H	-2.241343	1.440338	-0.008434
H	-3.719925	-0.365070	0.000733
H	-3.067642	-1.994474	0.003218
H	3.836488	-0.820739	0.000523
H	1.934524	-2.539909	0.001699
O	-5.539969	0.349344	0.009011
C	-5.783736	1.567073	0.002449
N	-7.123821	2.028984	0.010418
C	-7.523478	3.351347	0.005751
N	-6.673509	4.369340	-0.013214
C	-5.381526	3.973505	-0.014633
C	-4.875979	2.666434	-0.010899
H	-7.831993	1.294760	0.041809
N	-8.861892	3.588740	0.074225
N	-4.266727	4.778104	-0.028739
N	-3.497522	2.678522	-0.021043
H	-9.152952	4.546063	-0.101962
H	-9.515755	2.864306	-0.206435
C	-3.161775	3.954252	-0.031160
H	-4.261687	5.795492	-0.033376
H	-2.137899	4.320908	-0.039256

GG4

C	-6.949052	-4.557544	-0.004607
N	-6.477308	-5.786830	0.014230
C	-5.101972	-5.629308	0.011252
C	-4.751293	-4.271132	-0.010875
N	-5.948506	-3.600291	-0.020051
N	-3.516168	-3.715452	-0.021140
C	-2.530116	-4.614855	-0.009570
N	-2.777154	-5.970427	0.016050
C	-4.055878	-6.610179	0.026578
N	-1.246660	-4.205384	-0.038073
O	-4.122965	-7.841283	0.045750
N	-0.647217	-1.352129	0.027454
C	0.584142	-0.785780	0.015216
C	0.923639	0.575374	0.000956
C	-0.129922	1.548734	-0.003044
N	-1.403319	0.899226	0.007002
C	-1.639891	-0.458559	0.023588
N	2.297293	0.744658	-0.009136
C	2.779457	-0.480624	-0.001706
N	1.787290	-1.446465	0.013344
O	-0.071841	2.780334	-0.013683
N	-2.920302	-0.875622	0.046942
H	-2.199287	1.537463	0.006265
H	-3.675861	-0.202247	-0.006013
H	-3.136991	-1.894425	0.014720
H	3.832624	-0.750611	-0.006369
H	1.926249	-2.453683	0.019989
H	-1.986242	-6.614896	0.019324
H	-1.026169	-3.188215	-0.006680
H	-0.495630	-4.881542	0.042044
H	-6.077552	-2.591802	-0.036654
H	-7.999983	-4.279356	-0.008327

GT1

N	-0.697023	-1.372609	0.026708
C	0.535456	-0.816897	0.011528
C	0.889999	0.544712	0.001377
C	-0.162348	1.511566	0.003987
N	-1.426496	0.895561	0.026150
C	-1.660399	-0.457553	0.040657
N	2.265836	0.699967	-0.014647
C	2.733059	-0.532316	-0.014181
N	1.732877	-1.487463	0.000318
O	-0.080699	2.758684	-0.011401
N	-2.965486	-0.841391	0.120238
H	-2.252595	1.543111	0.028140
H	-3.691491	-0.168714	-0.111469
H	-3.165063	-1.817368	-0.074853
H	3.783754	-0.811897	-0.023754
H	1.852901	-2.497362	0.004760
O	-3.670242	2.485615	-0.006074
C	-3.604679	3.736558	-0.004407
C	-4.778798	4.592908	-0.002852
C	-4.578220	5.940575	-0.001011
N	-3.323859	6.492458	-0.000312
C	-2.148219	5.745797	-0.000826
N	-2.368550	4.374937	-0.002936
C	-6.138198	3.964406	-0.001535
H	-5.403122	6.653624	0.000493
H	-3.207979	7.503447	0.001040
O	-1.034275	6.261802	0.000210
H	-1.503248	3.773196	-0.005407
H	-6.922809	4.734066	-0.001457
H	-6.272577	3.320653	-0.884946
H	-6.271223	3.322191	0.883243

GT2

N	-2.383437	4.392061	-0.004002
C	-3.586821	3.736158	-0.003496
N	-4.690785	4.562001	-0.001491
C	-4.605857	5.936821	-0.000253
C	-3.414852	6.592421	-0.000907
C	-2.187373	5.790980	-0.002308
O	-3.704987	2.494075	-0.004166
H	-5.598990	4.103648	0.000689
O	-1.041821	6.247498	-0.002500
N	-1.449706	0.875216	0.027712
C	-0.186564	1.497658	0.004671
C	0.868732	0.533719	0.001795
C	0.519691	-0.829098	0.013890
N	-0.710899	-1.389323	0.029201
C	-1.678218	-0.479440	0.041792
N	2.243806	0.694334	-0.016368
C	2.715734	-0.536111	-0.015175
N	1.719472	-1.495303	0.001912
O	-0.109067	2.743841	-0.010975
N	-2.981800	-0.870052	0.120000
H	-2.274555	1.517206	0.032726
H	-3.709908	-0.204847	-0.126118
H	-3.173139	-1.848146	-0.073756
H	3.767384	-0.811845	-0.025823
H	1.844266	-2.504693	0.007110
H	-5.564952	6.455604	0.001239
C	-3.312303	8.092189	-0.000324
H	-1.518402	3.792840	-0.006415
H	-2.254646	8.386470	-0.002859
H	-3.794316	8.527634	-0.889047
H	-3.789690	8.526839	0.891290

TT1

C	-1.345643	-0.703451	-0.000379
C	-0.296219	-1.571226	0.000274
N	1.008735	-1.144111	0.000189
C	1.384692	0.195108	-0.000082
N	0.305458	1.065649	-0.000187
C	-1.046657	0.721408	-0.000513
O	2.559288	0.557876	-0.000138
O	-1.922755	1.608959	-0.000685
C	-2.777773	-1.140231	-0.000366
H	1.769054	-1.820148	0.000632
H	0.532464	2.085243	-0.000014
H	-3.304669	-0.747165	0.883422
H	-3.304735	-0.747348	-0.884202
H	-2.852516	-2.236679	-0.000282
H	-0.432417	-2.653220	0.001003
O	0.810227	3.847744	0.000290
C	-0.103017	4.690725	0.000111
N	0.158742	6.047271	-0.000020
C	-0.828599	7.006513	-0.000026
C	-2.152344	6.692343	-0.000019
C	-2.523085	5.274849	-0.000067
N	-1.439319	4.374198	0.000019
H	1.139363	6.318045	0.000277
H	-0.468018	8.035536	-0.000033
C	-3.237906	7.732240	-0.000007
O	-3.679314	4.841724	-0.000140
H	-1.663554	3.351399	-0.000085
H	-4.217432	7.235904	0.000409
H	-3.178022	8.377159	0.890048
H	-3.178545	8.376647	-0.890468

TT2

C	-1.403014	-0.804040	-0.000150
C	-0.347007	-1.663924	0.000558
N	0.954620	-1.227263	0.000535
C	1.320200	0.115748	-0.000137
N	0.234541	0.977521	-0.000720
C	-1.113569	0.622813	-0.000729
O	2.491997	0.486688	-0.000149
O	-1.996131	1.505169	-0.001135
C	-2.831118	-1.253767	-0.000077
H	1.720044	-1.897531	0.001002
H	0.453383	2.002958	-0.000918
H	-3.362566	-0.867021	0.883677
H	-3.362654	-0.867276	-0.883890
H	-2.895167	-2.350871	0.000079
H	-0.475801	-2.746850	0.001294
O	0.746440	3.728748	-0.000828
C	-0.132635	4.614647	-0.000410
C	0.163781	6.039962	0.000242
C	-0.888176	6.904960	0.000654
N	-2.191582	6.474560	0.000335
C	-2.563927	5.133734	-0.000298
N	-1.482729	4.265718	-0.000489
C	1.594444	6.482354	0.000486
H	-0.754542	7.987203	0.001259
H	-2.953761	7.148508	0.000782
O	-3.737797	4.769066	-0.000602
H	-1.706064	3.241450	-0.000697
H	1.664072	7.579312	0.000911
H	2.123473	6.091669	0.884146
H	2.123569	6.092337	-0.883407

TT3

C	-1.215203	-0.538902	0.000078
C	-0.260982	-1.507318	0.000294
N	1.084797	-1.213500	0.000310
C	1.580962	0.076157	0.000118
N	0.612716	1.052206	-0.000106
C	-0.782833	0.858769	-0.000122
O	2.800896	0.309543	0.000175
O	-1.543031	1.831135	-0.000299
C	-2.683461	-0.830916	0.000004
H	1.774479	-1.961539	0.000419
H	0.961365	2.036154	-0.000156
H	-3.167898	-0.385926	0.883541
H	-3.167798	-0.385780	-0.883511
H	-2.868624	-1.914111	-0.000087
H	-0.502610	-2.570531	0.000441

O	1.692352	3.657707	0.000057
C	2.910207	3.900849	0.000414
N	3.392753	5.194911	0.000951
C	4.734176	5.500798	0.001372
C	5.702537	4.545261	0.001262
C	5.283886	3.141569	0.000673
N	3.888432	2.935565	0.000331
H	2.695819	5.936045	0.000737
H	4.961317	6.567167	0.001820
C	7.168722	4.878700	0.001714
O	6.045205	2.169721	0.000457
H	3.550275	1.948908	0.000033
H	7.755782	3.950895	0.001165
H	7.447224	5.464650	-0.887760
H	7.446981	5.463408	0.892080

U-DAP

C	-0.168671	-1.498409	0.290778
C	1.139515	-1.306760	-0.020824
N	1.624602	-0.058166	-0.309035
C	0.836030	1.087783	-0.302150
N	-0.487289	0.873984	0.010392
C	-1.071135	-0.364323	0.313719
O	1.313843	2.200129	-0.558567
O	-2.284678	-0.435429	0.574221
H	-0.560855	-2.486484	0.518055
H	2.607268	0.075714	-0.536839
H	-1.121449	1.744136	0.022505
H	1.869391	-2.114848	-0.060812
N	-2.147686	3.164680	0.026332
C	-1.621690	4.405649	0.171837
C	-2.420725	5.569086	0.149514
C	-3.794784	5.415625	-0.025307
C	-4.353560	4.148294	-0.172697
C	-3.487192	3.032987	-0.142884
N	-0.267214	4.479294	0.392173
H	-1.961166	6.551293	0.265168
H	-4.439194	6.297466	-0.048482
H	-5.425921	4.004838	-0.310089
N	-3.960964	1.760012	-0.332112
H	0.306955	3.695088	0.057409
H	0.153586	5.395859	0.269862
H	-3.383201	0.977554	0.006673
H	-4.963891	1.633019	-0.236569

Coordinates of Contact ion-pair complexes

Z-1

C	8.592736	16.899448	17.149256
C	9.021926	16.875509	15.794116
C	10.201536	17.657629	15.688875
C	10.515477	18.164322	16.981455
C	9.510790	17.699491	17.884336
Zr	10.823316	15.724882	17.180415
F	12.177266	15.148803	15.379670
C	12.936002	15.723645	14.334550
C	13.097262	14.993113	13.167044
C	13.898789	15.648190	12.222685
C	14.455699	16.916984	12.430276
C	14.249280	17.586393	13.635180
C	13.475112	16.969204	14.617301
B	12.564056	13.422657	13.097975
C	10.949061	13.250863	13.400332
C	9.982567	14.256297	13.326475
C	8.606640	14.011664	13.387374
C	8.140497	12.710551	13.550262
C	9.064775	11.668226	13.629417
C	10.424854	11.957905	13.537826
F	14.198652	15.074134	11.040652
F	15.208602	17.495271	11.479266
F	14.790561	18.793939	13.854153
F	13.271726	17.569525	15.812902
F	10.334198	15.564895	13.179756
F	7.718322	15.029489	13.297342
F	6.821164	12.462732	13.628781
F	8.631379	10.401120	13.778907
F	11.267182	10.896479	13.572188
C	12.669047	12.746685	11.596993
C	13.209222	11.494346	11.291385
C	13.136525	10.914947	10.020010
C	12.484651	11.586845	8.989197
C	11.917907	12.835321	9.246701
C	12.014827	13.371378	10.528187
F	13.838008	10.746067	12.231885
F	11.425813	14.581625	10.721528
F	11.278650	13.497574	8.259967
F	12.396354	11.039608	7.762727
F	13.681903	9.702700	9.786444
C	13.555812	12.716969	14.215123
C	13.191890	12.255044	15.480333
C	14.096574	11.708462	16.396316
C	15.450331	11.644736	16.075637
C	15.872863	12.127731	14.836542
C	14.930714	12.654013	13.954435
F	11.903179	12.329434	15.914708
F	13.671681	11.263846	17.600964
F	16.335732	11.135508	16.949362
F	17.180172	12.085009	14.512291
F	15.405885	13.110328	12.769620
C	10.999096	15.426539	19.641317
C	11.949883	16.434442	19.296358
C	12.955745	15.832148	18.500770
C	12.638713	14.447026	18.365826
C	11.442194	14.193631	19.085971
C	9.334553	14.031898	17.093577
H	13.806557	16.342092	18.053454
H	11.899831	17.483597	19.574383
H	10.937117	13.236877	19.172301
H	10.101106	15.571780	20.237541
H	13.214656	13.715219	17.806759
H	9.455760	17.922165	18.946491
H	11.364079	18.794143	17.235386
H	10.775688	17.815487	14.778522
H	8.536683	16.343929	14.981880
H	7.725327	16.383819	17.551984
H	8.654656	13.974665	17.956959
H	8.724920	14.143067	16.183523
H	9.903968	13.089890	17.047520

Z-2

C	13.048223	6.904536	14.792561
C	12.709887	8.216202	14.358919
C	13.891146	9.009874	14.385447
C	14.956157	8.204311	14.864364
C	14.433034	6.896339	15.118604
Zr	13.396564	8.398986	16.784785
F	12.705918	10.563014	16.682620
C	13.137679	11.821849	17.182215
C	14.363603	12.289352	16.719275
C	14.769632	13.547850	17.160895
C	13.954041	14.297055	18.026010
C	12.725142	13.794675	18.467056
C	12.331210	12.489643	18.053452
F	15.140214	11.557052	15.891799
F	15.948597	14.043704	16.745763
F	14.426569	15.504535	18.390756
C	11.671119	14.377623	19.321086
C	10.611321	13.443689	19.483210
C	9.516303	13.778477	20.252668
C	9.410075	15.034455	20.857408
C	10.444258	15.957465	20.689210
C	11.571254	15.634834	19.924556
B	10.943471	12.039925	18.753208
C	11.068505	10.832672	19.830772
C	10.131044	9.802771	19.544891
C	9.970651	8.759142	20.462581
C	10.746910	8.718919	21.629663
C	11.688983	9.716615	21.892061
C	11.831871	10.765541	20.978374
F	12.521489	16.588738	19.806798
F	10.345433	17.173509	21.263275
F	8.332764	15.368570	21.593394
F	8.506147	12.889793	20.438353
C	9.441524	10.093605	18.270495
C	9.844353	11.368148	17.783688
C	9.353136	11.826703	16.578945
C	8.472718	11.058054	15.811030
C	8.091219	9.799032	16.278893
C	8.574086	9.313868	17.500851
F	8.180133	8.071545	17.859736
F	7.261416	9.038789	15.534725
F	8.000620	11.502963	14.629304
F	9.739118	13.035668	16.091428
F	12.748597	11.732072	21.252742
F	12.438572	9.653162	23.009469
F	10.587181	7.710556	22.509422
F	9.068578	7.766162	20.300035
C	14.316978	9.345126	18.932279
C	13.534085	8.222403	19.308776
C	14.142899	7.057269	18.768042
C	15.306798	7.462460	18.053555
C	15.410429	8.882478	18.144466
C	11.495297	7.279670	17.229697
H	11.718125	8.555917	14.066836
H	13.955057	10.066279	14.134680
H	14.999375	6.039197	15.472913
H	15.984931	8.523038	15.013925
H	12.364610	6.065396	14.881043
H	16.001209	6.806088	17.537133
H	16.190131	9.496998	17.699881
H	14.100168	10.381907	19.183280
H	12.610359	8.257966	19.877959
H	13.774098	6.039898	18.870966
H	10.767488	7.491361	16.428865
H	11.612465	6.187582	17.297316
H	11.102474	7.635845	18.194270

Z-3

C	8.037797	9.100873	18.337879
C	9.195793	8.265421	18.264078
C	9.845892	8.537296	17.029396
C	9.122829	9.558333	16.361024
C	7.995484	9.898499	17.162887
Zr	10.017334	10.602805	18.499518
C	9.369511	12.037989	20.473694
C	9.054669	10.690921	20.795970
C	10.280120	9.959751	20.891173
C	11.344594	10.870300	20.651067
C	10.786526	12.143871	20.369936
F	12.200560	9.647650	18.219264
B	12.812452	10.624874	17.302439
F	11.686233	11.566792	17.147787
F	13.870166	11.251336	17.915350
F	13.101410	10.029568	16.097963
C	9.095153	12.516198	17.647476
H	10.380876	8.903002	21.125533
H	12.400453	10.615424	20.607487
H	8.657905	12.842517	20.317718
H	11.341565	13.038576	20.096165
H	8.058328	10.291059	20.960432
H	9.397493	10.022719	15.416492
H	7.246896	10.646973	16.923392
H	9.512558	7.539122	19.008398
H	7.307801	9.111758	19.142021
H	10.782715	8.098517	16.694717
H	9.134621	12.437110	16.549639
H	8.056477	12.709773	17.952351
H	9.725146	13.357055	17.977680

Z-4

C	7.979701	9.137333	18.331438
C	9.134041	8.292899	18.305197
C	9.820948	8.537866	17.087252
C	9.122290	9.549895	16.377106
C	7.970989	9.906241	17.136223
Zr	9.952321	10.639109	18.506942

C	9.356639	12.062664	20.500577
C	9.006960	10.719835	20.805923
C	10.214193	9.956466	20.890315
C	11.300084	10.838903	20.650681
C	10.774630	12.130902	20.384058
F	12.172100	9.788110	18.140551
P	13.022993	10.853154	17.053109
F	11.527037	11.738447	17.085166
F	13.671307	11.938854	16.037595
F	12.412382	9.976103	15.809806
C	8.941512	12.532811	17.710626
H	10.288564	8.895600	21.114564
H	12.348622	10.558425	20.596999
H	8.664889	12.887340	20.361896
H	11.354066	13.011181	20.114278
H	8.000946	10.344311	20.970731
H	9.430628	9.994241	15.433379
H	7.229519	10.648531	16.859106
H	9.424829	7.579028	19.071515
H	7.227712	9.166984	19.114910
H	10.764590	8.090156	16.786653
H	8.948220	12.474120	16.611150
H	7.906588	12.678096	18.052542
H	9.547958	13.392434	18.036568
F	13.494021	11.716505	18.364331
F	14.341736	9.910017	17.136858

Z-5

C	12.931632	10.200220	15.243410
C	11.710512	10.376119	15.946945
C	11.922637	10.006418	17.302249
C	13.271020	9.572438	17.437554
C	13.899541	9.697083	16.168523
Zr	13.381003	12.031338	16.869813
F	10.989244	14.092888	12.122536
C	10.677759	14.819322	13.214165
C	10.960562	14.336947	14.495537
C	10.677860	15.047423	15.661991
C	10.044024	16.278975	15.462786
C	9.741892	16.802121	14.207456
C	10.061344	16.060310	13.068099
F	11.549450	13.106746	14.539320
B	10.888961	14.539191	17.194828
C	11.928707	15.374611	18.125089
C	12.175041	14.898483	19.420437
C	13.106395	15.463876	20.288685
C	13.834888	16.579385	19.871317
C	13.620698	17.096568	18.594265
C	12.686377	16.486505	17.750608
F	9.711524	17.028843	16.542035
F	9.148658	18.003126	14.077840
F	9.780133	16.539667	11.845056
F	11.486093	13.829993	19.887323
F	13.307413	14.954710	21.517876
F	14.734181	17.144256	20.691520
F	14.329672	18.164112	18.181459
F	12.577578	17.022654	16.510528
C	14.587446	13.064083	14.956105
C	15.557984	12.301692	15.672233
C	15.751289	12.913759	16.940317
C	14.909795	14.058965	17.005938
C	14.201349	14.158135	15.780720
F	11.547992	13.132179	17.154571
C	9.413539	14.285813	17.839207
C	8.601678	13.268724	17.324686
C	7.311211	13.004879	17.783824
C	6.775639	13.799936	18.798257
C	7.538522	14.840338	19.327948
C	8.829514	15.063990	18.842583
F	9.056689	12.485527	16.310619
F	6.576261	12.005315	17.255815
F	5.534043	13.568711	19.257522
F	7.023032	15.617947	20.300379
F	9.503511	16.097324	19.402149
C	13.932055	11.898838	19.062944
H	11.191792	10.076327	18.104839
H	10.793984	10.796414	15.540451
H	14.929567	9.437475	15.939080
H	13.100237	10.409036	14.189750
H	13.742180	9.228976	18.353578
H	14.213828	12.853094	13.957551
H	13.463046	14.915756	15.527524
H	14.803902	14.719185	17.862730
H	16.408924	12.562055	17.730391
H	16.061107	11.410709	15.307745
H	13.062700	11.487812	19.601643
H	14.803372	11.262190	19.277143
H	14.152825	12.912333	19.433370

Z-6

C	1.468027	4.911623	10.953332
C	1.688046	5.449521	9.681759
C	1.562276	6.842872	9.595883
C	1.201982	7.654489	10.668761
C	0.961067	7.068296	11.912212
C	1.096903	5.688829	12.054970
B	2.231087	4.604410	8.378849
C	1.490441	4.926615	6.951284
C	2.145564	4.581590	5.767972
C	1.606222	4.764028	4.497570
C	0.326361	5.306341	4.380795
C	-0.376772	5.652120	5.535096
C	0.207069	5.452614	6.790280
F	3.388995	4.022999	5.831461
F	2.299242	4.418132	3.391179
F	-0.224144	5.489069	3.169594
F	-1.614094	6.170983	5.426184
F	-0.538246	5.799352	7.862437
F	1.819325	7.466408	8.418575
F	1.112204	8.991409	10.530201
F	0.629797	7.829094	12.967060
F	0.894776	5.121296	13.257706
F	1.615093	3.586973	11.189385
C	3.686470	5.223831	8.298501
N	4.664523	5.843035	8.363828
B	5.879882	6.671110	8.838216
C	5.281956	7.432617	10.162916
C	5.070986	8.809370	10.282484
C	4.460166	9.393553	11.398107
C	4.032962	8.587949	12.451901
C	4.216822	7.205782	12.373838
C	4.819772	6.665762	11.240832
F	5.451764	9.665902	9.305004
F	4.280421	10.725296	11.462187
F	3.442600	9.132837	13.526204
F	3.801387	6.417599	13.383672
F	4.953414	5.314744	11.208753
C	6.415813	7.670278	7.663539
C	5.697914	8.025854	6.517969
C	6.181787	8.930713	5.567095
C	7.430611	9.524155	5.750782
C	8.179017	9.204478	6.885419
C	7.658491	8.297215	7.808275
F	4.464886	7.521231	6.285326
F	8.404068	8.064007	8.915833
F	9.379015	9.779442	7.078883
F	7.908243	10.394113	4.848117
F	5.452433	9.241005	4.479517
C	7.049381	5.549680	9.157732
C	7.332378	4.541232	8.251322
C	8.280747	3.540697	8.416519
C	9.046283	3.539177	9.580147
C	8.827637	4.539382	10.529504
C	7.853325	5.517497	10.303634
F	6.622377	4.489641	7.034974
Zr	7.223433	4.130224	4.797417
C	5.374432	2.741552	3.790228
C	5.705282	2.154373	5.041509
C	7.035435	1.643072	4.951626
C	7.526275	1.927298	3.650585
C	6.506518	2.623232	2.937358
F	8.485831	2.616009	7.453906
F	9.980812	2.601598	9.773920
F	9.565354	4.555063	11.648424
F	7.721483	6.456635	11.258154
C	2.254386	2.981737	8.533753
C	1.037394	2.305399	8.658879
C	0.933252	0.917694	8.730251
C	2.093859	0.143364	8.659489
C	3.329853	0.773912	8.524631
C	3.384050	2.168629	8.463507
F	-0.110798	3.019521	8.734929
F	-0.264208	0.317777	8.865424
F	2.019216	-1.197003	8.719170
F	4.456195	0.035633	8.446961
F	4.626073	2.713455	8.320583
C	8.660935	5.794792	3.587873
C	8.726367	6.163309	4.959649
C	9.383039	5.119747	5.665943
C	9.699679	4.086341	4.743659
C	9.256347	4.510078	3.452071
C	5.772775	5.618423	3.925525
H	8.317210	7.070054	5.397992
H	8.213240	6.378822	2.789088
H	9.366922	3.953928	2.525327
H	10.196727	3.147964	4.976505
H	7.580880	1.130826	5.739798
H	8.511325	1.668343	3.271331
H	6.577795	2.995627	1.917762
H	4.437455	3.229754	3.542539
H	5.062164	2.111931	5.917240
H	9.590927	5.109501	6.733933
H	4.806680	5.412749	4.418299
H	6.084683	6.647796	4.145615
H	5.614571	5.527305	2.841246

Z-7

C	15.973740	3.361602	6.192910
C	15.279895	2.472522	5.366852
C	16.046872	1.404579	4.892327
C	17.386418	1.207744	5.230162
C	18.022597	2.109458	6.082545
C	17.305253	3.199772	6.571677
B	13.713840	2.731228	4.981662
N	13.624784	3.890365	3.838259
B	14.753659	4.888079	3.214901
C	14.075779	5.456691	1.791571
C	12.780326	5.981424	1.797283
C	12.123789	6.503252	0.685634
C	12.798317	6.557193	-0.532391
C	14.106478	6.080418	-0.595517
C	14.716177	5.553182	0.548601
F	12.063980	6.017293	2.969347
F	10.855837	6.966718	0.779723
F	12.199022	7.062419	-1.622365
F	14.774069	6.133735	-1.762930
F	15.988283	5.130513	0.376115
F	15.523163	0.502858	4.022941
F	18.083675	0.178319	4.708704
F	19.316405	1.944316	6.402160
F	17.908452	4.094322	7.376872
F	15.343431	4.457240	6.684697
C	12.754734	3.112584	6.300680
C	11.443792	3.550361	6.132071
C	10.493107	3.659950	7.130539
C	10.820172	3.363991	8.446419
C	12.122480	2.935584	8.695868
C	13.041108	2.805285	7.643910
F	10.952331	3.885572	4.865525
Zr	8.579062	5.038790	4.573634
C	9.016352	6.955292	6.179705
C	10.061977	6.976361	5.211909
C	9.486587	7.259282	3.946307
C	8.077500	7.412985	4.132191
C	7.792847	7.239724	5.515701

F	14.244182	2.332712	8.017704
F	12.480623	2.630325	9.951503
F	9.917596	3.476093	9.426565
F	9.209306	4.024264	6.801981
C	8.755337	3.037819	3.065278
C	7.365697	3.262119	3.221461
C	7.056166	4.528332	2.647372
C	8.253317	5.065154	2.094425
C	9.309330	4.158122	2.369848
C	12.913737	1.367228	4.449126
C	12.944249	0.214069	5.248229
C	12.235247	-0.954993	4.976144
C	11.418579	-1.013428	3.846515
C	11.338255	0.103662	3.020564
C	12.073001	1.249192	3.338455
F	13.702131	0.200990	6.370501
F	12.320452	-2.020955	5.792675
F	10.718934	-2.122992	3.563607
F	10.547552	0.078232	1.929633
F	11.899242	2.287086	2.456030
C	16.076902	3.976281	2.912804
C	17.360231	4.169491	3.427345
C	18.447583	3.355958	3.098515
C	18.274367	2.285539	2.222833
C	17.006723	2.039759	1.695314
C	15.956219	2.879358	2.053845
F	17.614671	5.152426	4.325490
F	19.656652	3.568947	3.654152
F	19.308776	1.487014	1.913050
F	16.814832	1.001822	0.859458
F	14.740625	2.590505	1.501589
C	14.971990	6.261553	4.078424
C	15.874557	7.220010	3.597518
C	16.070905	8.469857	4.180392
C	15.310571	8.830751	5.293399
C	14.373286	7.929853	5.791312
C	14.218298	6.688935	5.170165
F	16.610494	6.945977	2.492587
F	16.967352	9.338076	3.674481
F	15.473480	10.034481	5.867862
F	13.609982	8.268817	6.851154
F	13.237280	5.897121	5.698823
H	12.822533	4.482212	4.054578
H	13.329806	3.393162	2.993523
C	6.676968	4.607106	5.740083
H	11.115338	6.782673	5.402693
H	9.130380	6.742580	7.240632
H	6.812489	7.296119	5.977726
H	7.353167	7.646661	3.356000
H	10.020618	7.340344	3.005641
H	9.305869	2.167081	3.414609
H	10.351222	4.283978	2.090463
H	8.349548	6.012867	1.572418
H	6.074285	4.994373	2.624330
H	6.664182	2.603870	3.724983
H	6.752801	5.037993	6.748903
H	5.759556	4.980841	5.262397
H	6.600880	3.508913	5.813813

Z-8

C	5.529996	-7.081797	17.130710
C	5.764852	-8.416663	16.690149
C	7.160522	-8.569037	16.472117
C	7.792973	-7.329745	16.797135
C	6.785377	-6.419580	17.215492
Zr	6.367932	-6.941099	14.772221
C	5.405768	-6.730262	12.437461
C	5.966603	-8.033651	12.540646
C	5.240710	-8.753808	13.527021
C	4.215647	-7.890794	14.028028
C	4.317244	-6.644175	13.348608
C	5.924080	-4.738044	14.932918
F	8.384083	-6.346585	13.833602
C	9.656444	-6.804577	13.444015
C	9.809541	-8.180028	13.433776
F	8.784436	-8.993040	13.777412
C	10.617307	-5.858097	13.129589
B	10.198925	-4.260908	13.150293
N	9.023696	-4.193078	12.158481
C	8.102513	-4.087023	11.448508
N	7.047286	-4.072438	10.723614
C	6.822238	-3.256959	9.752039
N	6.521869	-2.558149	8.869473
B	6.394566	-1.571814	7.681552
C	6.158182	-2.472370	6.324453
C	5.174771	-3.467979	6.333717
F	4.467924	-3.712871	7.469362
C	11.839426	-6.417211	12.743358
F	12.876740	-5.639193	12.381626
C	12.066640	-7.801075	12.706445
F	13.259104	-8.282330	12.325729
C	11.049929	-8.693338	13.052906
F	11.255035	-10.015330	13.016260
C	9.605554	-3.755929	14.605887
C	8.884901	-2.556817	14.671742
F	8.677844	-1.828862	13.547724
C	11.385185	-3.224655	12.722696
C	11.420409	-2.417750	11.582087
F	10.470421	-2.514026	10.616801
C	9.791303	-4.399514	15.830588
F	10.504385	-5.553806	15.911478
C	9.271436	-3.922394	17.036269
F	9.465803	-4.599138	18.186600
C	8.539007	-2.735936	17.047744
F	8.028213	-2.267647	18.197386
C	8.344328	-2.043327	15.852042
F	7.638732	-0.899162	15.850518
C	12.442918	-3.033628	13.618739
F	12.513112	-3.803741	14.734792
C	13.459320	-2.100276	13.431744
F	14.449449	-1.960572	14.333671
C	13.442957	-1.302574	12.286162
F	14.406747	-0.389968	12.083383
C	12.419884	-1.466056	11.353860
F	12.400536	-0.704002	10.245121
C	5.052765	-0.645913	7.827537
C	4.062772	-0.761150	8.804339
F	4.176438	-1.652322	9.823241
C	7.821121	-0.760379	7.759345
C	7.979903	0.569401	8.158020
F	6.911901	1.342709	8.471314
C	4.801991	0.309477	6.837076
F	5.714963	0.496187	5.851490
C	3.667901	1.117206	6.808353
F	3.491178	2.036730	5.838853
C	2.704597	0.967869	7.807322
F	1.597402	1.731726	7.802532
C	2.903664	0.021966	8.810241
F	1.974801	-0.130820	9.777024
C	9.017188	-1.452894	7.534336
F	8.977721	-2.768582	7.196134
C	10.281626	-0.875509	7.631714
F	11.394138	-1.593931	7.384781
C	10.387797	0.465760	8.002752
F	11.592770	1.050186	8.106153
C	9.228399	1.191233	8.268172
F	9.320763	2.483349	8.643197
C	6.785978	-2.271943	5.092435
F	7.739963	-1.323718	4.941626
C	6.481329	-3.019373	3.950624
F	7.120445	-2.790211	2.785450
C	5.503997	-4.011365	4.014999
F	5.199020	-4.738358	2.925337
C	4.843071	-4.240695	5.221364
F	3.889687	-5.192425	5.296290
H	8.860219	-7.123876	16.740447
H	7.657646	-9.467995	16.114694
H	5.008359	-9.180418	16.533720
H	4.562073	-6.644753	17.366026
H	6.936859	-5.389483	17.523165
H	3.476244	-8.147410	14.782348
H	5.429012	-9.776846	13.843932
H	6.826819	-8.405652	11.987301
H	5.755021	-5.932278	11.784059
H	3.688052	-5.773702	13.510261
H	5.047469	-4.472560	15.542509
H	6.811765	-4.296291	15.418584
H	5.797054	-4.299880	13.931024

Coordinates of newly designed planar cationic AAAA-DDDD molecules

A-1

C	0.025239	18.039578	20.704515
C	0.426322	17.888001	19.375360
C	1.351575	18.771742	18.754410
C	1.894719	19.841283	19.477514
C	1.495884	19.996588	20.807856
C	0.577416	19.111960	21.411558
N	1.562907	18.378626	17.437183
C	0.792126	17.305373	17.288839
N	0.089715	16.960151	18.404392
N	0.660222	16.546789	16.131644
C	1.318857	16.827715	14.971508
N	2.134955	17.865036	14.882102
N	1.099619	15.997452	13.915537
C	1.711022	16.171133	12.677699
N	1.447646	15.310728	11.653827
C	2.207524	15.747381	10.581712
C	2.905677	16.896287	11.045170
N	2.571720	17.140019	12.371099
C	3.774826	17.585740	10.189950
C	3.922724	17.103314	8.887104
C	3.223386	15.962712	8.438501
C	2.351917	15.263261	9.278884
N	-0.138179	13.114398	11.828352
B	-0.797908	12.844119	13.088411
N	-1.656048	11.603360	13.181504
C	-1.771437	10.722667	12.068895
C	-1.079710	11.085150	10.886739
C	-0.290557	12.291209	10.835861
C	-1.168353	10.242568	9.760539
C	-1.912492	9.077395	9.805519
C	-2.602272	8.702023	10.981575
C	-2.520578	9.536610	12.100431
C	-3.404362	7.401765	10.992669
C	-2.447833	6.226421	10.694115
C	-2.296852	11.375356	14.339344
C	-2.163358	12.236900	15.429618
C	-2.855978	11.963862	16.623823
C	-2.722181	12.810400	17.709225
C	-1.841514	13.951579	17.543721
N	-1.639181	14.835066	18.583656
C	-2.250243	14.639739	19.728761
C	-3.140372	13.561932	20.010642
C	-3.397457	12.611734	18.982777
C	-3.759425	13.424449	21.269086
C	-4.621803	12.365452	21.507828
C	-4.897014	11.409700	20.501848
C	-4.275637	11.552521	19.254382
N	-0.657756	13.707482	14.205402
C	-1.309857	13.415958	15.334653
N	-1.174438	14.237121	16.415513
C	-5.848106	10.234045	20.730017
C	-5.081493	8.912785	20.505843
C	-6.439589	10.219861	22.146619
C	-7.008601	10.323321	19.715058
C	-4.095527	7.140463	12.337722
C	-4.482395	7.470095	9.889643
H	0.811162	14.445952	11.698162
H	0.443939	15.170887	14.039965
H	4.316580	18.468000	10.535653
H	4.594555	17.619358	8.198649
H	3.366082	15.618895	7.412430
H	1.812047	14.381521	8.927345
H	2.252232	18.444275	15.733482

H	2.602671	18.007937	13.966232
H	0.016304	15.718818	16.177189
H	-0.581638	16.141709	18.474744
H	-0.684499	17.356526	21.175758
H	0.291284	19.267920	22.453268
H	1.902928	20.821362	21.395719
H	2.604987	20.526962	19.012278
H	-2.049625	15.372328	20.520353
H	-3.551888	14.159961	22.049691
H	-5.091737	12.273763	22.486244
H	-4.490251	10.814601	18.480286
H	-3.491279	11.077128	16.671020
H	-2.937924	10.499178	14.431776
H	-3.052992	9.238675	13.000802
H	-1.965950	8.438313	8.923445
H	-0.633699	10.528021	8.851867
H	0.211982	12.507404	9.880085
H	-7.110784	9.354090	22.244281
H	-7.025910	11.129232	22.349512
H	-5.653823	10.128598	22.912403
H	-6.640864	10.286395	18.678580
H	-7.576739	11.256104	19.850941
H	-7.690257	9.472781	19.868224
H	-4.679390	8.848147	19.483769
H	-5.767008	8.065129	20.659133
H	-4.246135	8.819510	21.216466
H	-4.810458	7.940062	12.587633
H	-3.365207	7.046728	13.156972
H	-4.653927	6.195112	12.274881
H	-5.181456	8.299393	10.076501
H	-5.051116	6.527622	9.879741
H	-4.033087	7.608541	8.895319
H	-1.666517	6.151561	11.466179
H	-1.961372	6.342943	9.714595
H	-3.020189	5.286107	10.683445

A-2

C	-2.536098	9.523921	12.109291
C	-1.787702	10.716304	12.092154
C	-1.100024	11.069431	10.901922
C	-1.176619	10.235343	9.766204
C	-1.919450	9.070693	9.813432
C	-2.613467	8.691687	10.990312
C	-1.661358	11.626325	13.223845
C	-0.827049	12.824105	13.101742
N	-0.192714	13.094342	11.898019
C	-0.331006	12.273592	10.887295
C	-2.307835	11.399885	14.418961
N	-2.166948	12.257451	15.472489
B	-1.286154	13.464020	15.341250
N	-0.637850	13.687077	14.088038
N	-1.149037	14.319852	16.477461
C	-1.806804	14.027235	17.588164
C	-2.686031	12.863523	17.725067
C	-2.823066	12.017954	16.647357
C	-3.381078	12.628572	18.986556
C	-3.144667	13.558672	20.035787
C	-2.258075	14.653283	19.792973
N	-1.633710	14.884515	18.666151
C	-3.778905	13.393169	21.281689
C	-4.638568	12.324752	21.490466
C	-4.893394	11.388276	20.463635
C	-4.255776	11.559022	19.227375
C	-5.840821	10.203477	20.654267
C	-6.451271	10.159270	22.061773
C	-3.411646	7.389420	10.992008
C	-4.108688	7.121790	12.332964
C	-6.987071	10.309084	19.625024
C	-5.062851	8.891675	20.415022
C	-4.485156	7.456684	9.884637
C	-2.451238	6.216991	10.694781
N	1.426531	15.305147	11.686389
C	2.180907	15.721514	10.602511
C	2.885055	16.874488	11.046627
N	2.559513	17.139615	12.371724
C	1.698342	16.179396	12.696874
C	3.752082	17.547312	10.176323
C	3.891817	17.044479	8.880153
C	3.186472	15.900036	8.452044
C	2.316496	15.216811	9.307199
N	1.088554	16.012542	13.934792
C	1.309203	16.843282	14.991482
N	0.655886	16.569548	16.155089
C	0.800142	17.335671	17.305811
N	0.105501	17.003321	18.431233
C	0.456516	17.937640	19.391104
C	1.380683	18.811393	18.754806
N	1.577590	18.406478	17.439833
C	1.936760	19.884146	19.463186
C	1.551470	20.052907	20.795707
C	0.633941	19.178267	21.415385
C	0.068998	18.102870	20.722930
N	2.128353	17.878034	14.891294
H	0.788334	14.458980	11.756654
H	0.439959	15.180615	14.032065
H	4.298906	18.432603	10.505954
H	4.562293	17.547229	8.180594
H	3.323193	15.539763	7.430807
H	1.771664	14.331772	8.972077
H	2.251880	18.460086	15.740655
H	2.594364	18.017841	13.975501
H	0.000944	15.740287	16.222202
H	-0.569862	16.189160	18.524552
H	-0.640140	17.427829	21.206265
H	0.358861	19.344901	22.458465
H	1.968587	20.880444	21.372576
H	2.646262	20.561656	18.984884
H	-2.078531	15.364875	20.608848
H	-3.586339	14.115290	22.078517
H	-5.121558	12.212162	22.460281
H	-4.459850	10.832211	18.440055
H	-3.449724	11.125354	16.676063
H	-2.956475	10.538108	14.581973
H	-3.067441	9.231146	13.013676
H	-1.971158	8.430557	8.931873
H	-0.641636	10.521170	8.857798
H	0.193583	12.542244	9.961668
H	-7.120614	9.289705	22.134379
H	-7.043309	11.062750	22.273845
H	-5.675166	10.056307	22.835699
H	-6.604292	10.288063	18.593563
H	-7.555930	11.240579	19.767114
H	-7.672157	9.457396	19.754989
H	-4.650517	8.848414	19.395919
H	-5.742445	8.035744	20.547240
H	-4.233215	8.792749	21.131665
H	-4.826999	7.918956	12.580994
H	-3.381702	7.030072	13.155361
H	-4.662966	6.174261	12.264490
H	-5.191091	8.279480	10.074278
H	-5.046848	6.510053	9.866581
H	-4.033586	7.604837	8.892723
H	-1.678883	6.136721	11.475251
H	-1.953494	6.340721	9.721806
H	-3.023030	5.276549	10.670654

A-3

C	0.098173	18.144892	20.721257
C	0.478544	17.969286	19.388160
C	1.402492	18.836162	18.743121
C	1.965879	19.911712	19.441954
C	1.587757	20.090583	20.775313
C	0.670016	19.222843	21.404548
N	1.591341	18.421767	17.431449
C	0.809590	17.350414	17.304506
N	0.118330	17.029103	18.437586
N	0.665789	16.584995	16.153701
C	1.321952	16.860880	14.991095
N	2.142176	17.895662	14.891160
N	1.098922	16.028059	13.935243
C	1.709072	16.193479	12.697779
N	1.440975	15.328251	11.675763
C	2.201894	15.754660	10.600506
C	2.906745	16.901894	11.056874
N	2.575405	17.154981	12.381354
C	3.779259	17.581065	10.196648
C	3.923939	17.089963	8.896377
C	3.217851	15.950776	8.455112
C	2.342596	15.261784	9.300645
N	-0.162299	13.117122	11.795977
B	-0.794106	12.883897	13.078658
N	-1.661675	11.645998	13.222645
C	-1.805128	10.737084	12.134620
C	-1.134980	11.065945	10.931159
C	-0.340306	12.268708	10.831280
C	-1.248372	10.194542	9.830627
C	-1.996974	9.033758	9.920545
C	-2.666994	8.694130	11.117567
C	-2.559454	9.558223	12.212547
C	-3.476976	7.400404	11.178697
C	-2.532088	6.211079	10.899225
C	-2.274454	11.457553	14.397388
C	-2.105189	12.352177	15.459078
C	-2.783948	12.074989	16.650779
N	-2.672112	12.873109	17.718368
B	-1.761243	14.081948	17.594656
N	-1.613525	14.936762	18.755631
C	-2.255866	14.652674	19.845618
C	-3.141127	13.521921	20.007175
C	-3.363268	12.614076	18.939191
C	-3.799813	13.304610	21.228802
C	-4.657050	12.224570	21.397429
C	-4.886946	11.320062	20.342740
C	-4.229442	11.533774	19.120747
N	-0.620146	13.771320	14.163937
C	-1.236036	13.535491	15.331467
N	-1.083017	14.350773	16.385127
C	-5.825193	10.120131	20.475004
C	-5.028981	8.823580	20.213539
C	-6.463192	10.029177	21.868074
C	-6.951018	10.242973	19.425711
C	-4.148067	7.182214	12.541592
C	-4.572557	7.446379	10.091762
H	0.802295	14.471064	11.705075
H	0.438120	15.194837	14.051850
H	4.326424	18.462294	10.536766
H	4.598499	17.597786	8.204385
H	3.358275	15.599631	7.431194
H	1.797506	14.380990	8.954916
H	2.264448	18.475657	15.742386
H	2.608500	18.031930	13.974733
H	0.011577	15.740070	16.203032
H	-0.565336	16.217735	18.570653
H	-0.611151	17.475611	21.212267
H	0.400332	19.397043	22.447812
H	2.010626	20.920184	21.345052
H	2.675525	20.583767	18.956112
H	-2.135056	15.306090	20.724154
H	-3.624744	14.006240	22.047795
H	-5.153529	12.083785	22.356607
H	-4.422866	10.827125	18.314538
H	-3.415476	11.187552	16.710293
H	-2.921138	10.589352	14.528654
H	-3.076450	9.287884	13.130699
H	-2.068648	8.371827	9.056664
H	-0.729613	10.452633	8.904513
H	0.141909	12.454998	9.858660
H	-7.124866	9.151317	21.901030
H	-7.068432	10.920482	22.094218
H	-5.701794	9.910710	22.654226
H	-6.548997	10.253483	18.401311
H	-7.533413	11.163574	19.581677
H	-7.628304	9.380202	19.517798
H	-4.596148	8.812417	19.201925

H	-5.703185	7.958293	20.306277
H	-4.213369	8.710562	20.943820
H	-4.852894	7.994217	12.780381
H	-3.404881	7.105111	13.351094
H	-4.714646	6.240146	12.514381
H	-5.262396	8.285720	10.267989
H	-5.148202	6.508480	10.116229
H	-4.139047	7.555741	9.086871
H	-1.742825	6.150762	11.664569
H	-2.054969	6.299747	9.912272
H	-3.110297	5.274510	10.918785

A-4

C	-4.199655	11.498385	18.983142
C	-3.340576	12.594251	18.871297
C	-3.155030	13.454701	19.982017
C	-3.841955	13.174558	21.173150
C	-4.691459	12.078720	21.274467
C	-4.885165	11.220728	20.177300
C	-2.280272	14.610931	19.906878
N	-1.613050	14.961007	18.858262
B	-1.695032	14.179717	17.631949
N	-1.011711	14.490222	16.470809
B	-1.123574	13.692982	15.316130
N	-0.520956	13.869447	14.056273
B	-0.731274	12.974438	13.023787
N	-1.639040	11.723107	13.256376
C	-2.229369	11.559862	14.424861
N	-2.050867	12.432435	15.451331
C	-2.713644	12.150902	16.604019
N	-2.616841	12.917440	17.672985
C	-5.809521	10.004280	20.233002
C	-6.906343	10.152796	19.157418
N	-0.157518	13.117333	11.693142
C	-0.385953	12.232827	10.780627
C	-1.195828	11.042898	10.968636
C	-1.828208	10.767032	12.202810
C	-2.588199	9.598215	12.350882
C	-2.742739	8.693776	11.294062
C	-2.109212	8.980728	10.066228
C	-1.352938	10.130547	9.909795
C	-3.564383	7.413494	11.430832
C	-4.689214	7.428363	10.373151
C	-2.641673	6.202149	11.173926
C	-4.199553	7.260508	12.819135
C	-4.985370	8.731464	19.943613
C	-6.485949	9.847423	21.601870
N	1.466971	15.374157	11.665983
C	1.738235	16.237501	12.694341
N	2.610930	17.193249	12.374965
C	2.940977	16.940518	11.051118
C	2.230358	15.798362	10.591613
C	3.817719	17.616421	10.192796
C	3.960425	17.127451	8.891263
C	3.248017	15.993473	8.446854
C	2.368467	15.307403	9.290774
N	1.126027	16.071342	13.927074
C	1.350010	16.900378	14.986276
N	2.174994	17.934634	14.886857
N	0.694258	16.629096	16.150847
C	0.841920	17.393928	17.297532
N	1.621031	18.467563	17.428219
C	1.434775	18.880213	18.739871
C	0.514088	18.011814	19.387218
N	0.153244	17.071769	18.437033
C	1.998089	19.956222	19.437818
C	1.623093	20.134165	20.772236
C	0.708278	19.264963	21.403902
C	0.136519	18.186043	20.721287
H	0.831433	14.525398	11.671117
H	0.456424	15.212923	14.014321
H	4.369213	18.493704	10.536274
H	4.638490	17.633184	8.201478
H	3.386375	15.643870	7.421901
H	1.818185	14.430536	8.943370
H	2.298014	18.515323	15.736438
H	2.642608	18.070574	13.972048
H	0.021368	15.773877	16.234611
H	-0.523266	16.270511	18.592390
H	-0.570475	17.515102	21.213222
H	0.440734	19.438391	22.447949
H	2.046442	20.964379	21.340575
H	2.705395	20.629150	18.949337
H	-2.202192	15.215187	20.824835
H	-3.696028	13.840718	22.026661
H	-5.209658	11.890699	22.213897
H	-4.371786	10.824086	18.144708
H	-3.334598	11.254344	16.616274
H	-2.886613	10.707572	14.601362
H	-3.076590	9.360086	13.293571
H	-2.214166	8.287958	9.230289
H	-0.861048	10.346878	8.958839
H	0.056906	12.360275	9.779981
H	-7.135812	8.960535	21.578044
H	-7.110129	10.720351	21.847298
H	-5.746587	9.705621	22.404830
H	-6.475527	10.211972	18.146517
H	-7.508722	11.056698	19.333775
H	-7.570834	9.275965	19.193829
H	-4.520946	8.769953	18.946708
H	-5.648452	7.853352	19.978046
H	-4.191269	8.599589	20.694196
H	-4.886689	8.091805	13.043399
H	-3.434972	7.204253	13.610508
H	-4.779286	6.326672	12.844995
H	-5.360918	8.286323	10.528396
H	-5.277594	6.501892	10.455634
H	-4.281489	7.484852	9.353312
H	-1.828868	6.164193	11.915676
H	-2.195320	6.243989	10.169768
H	-3.229491	5.274512	11.250366

B-1

C	-1.868330	9.159987	12.316024
C	-1.204872	10.402588	12.328653
C	-0.324053	10.720220	11.262095
C	-0.122321	9.793150	10.216158
C	-0.785283	8.580581	10.231045
C	-1.677009	8.242154	11.282342
C	-1.358355	11.392154	13.381654
C	-0.607556	12.628383	13.294907
N	0.174551	12.909999	12.194950
C	0.302500	12.003427	11.251838
C	-2.193217	11.233645	14.477201
C	-2.240490	12.219995	15.476412
C	-1.383162	13.374939	15.351493
N	-0.616049	13.570813	14.251742
N	-1.307195	14.307267	16.332418
C	-2.094596	14.174531	17.411969
C	-3.054336	13.102112	17.576770
C	-3.081490	12.121210	16.598425
C	-3.908493	13.099288	18.754212
C	-3.729238	14.154453	19.690711
C	-2.699435	15.114563	19.449082
N	-1.925850	15.135844	18.386993
C	-4.525393	14.205298	20.851751
C	-5.480632	13.227124	21.084820
C	-5.674524	12.165051	20.170617
C	-4.884593	12.125509	19.015163
C	-6.717583	11.069662	20.398671
C	-7.462081	11.233160	21.731293
C	-2.393872	6.893711	11.240930
C	-3.316708	6.676702	12.448201
C	-7.748161	11.116479	19.250257
C	-6.018610	9.693093	20.395749
C	-3.244346	6.822793	9.954090
C	-1.341584	5.764739	11.217487
N	0.920033	15.513686	11.577036
C	1.524602	16.028536	10.438389
C	2.203648	17.202223	10.860601
N	2.014430	17.388061	12.226442
C	1.269676	16.353417	12.595609
C	2.954238	17.952761	9.947680
C	3.015658	17.490371	8.630489
C	2.336811	16.326393	8.224629
C	1.555115	15.567756	9.112787
N	0.839444	16.078207	13.886553
C	1.192803	16.821999	14.970335
N	0.690296	16.427857	16.171768
C	0.994023	17.054260	17.372576
N	0.430932	16.594844	18.529016
C	0.905515	17.424450	19.535746
C	1.771350	18.350234	18.895260
N	1.815916	18.084247	17.530311
C	2.398736	19.359813	19.635057
C	2.125974	19.418785	21.004306
C	1.270781	18.491045	21.627672
C	0.646892	17.452265	20.915377
N	1.986259	17.874518	14.861819
H	0.578681	14.526427	11.743925
H	0.295216	15.187811	14.035873
H	3.477109	18.857661	10.261125
H	3.594450	18.048494	7.892294
H	2.387551	16.012985	7.179922
C	0.796612	14.379730	8.669318
H	2.226025	18.372904	15.737345
H	2.321722	18.111484	13.911177
H	-0.015734	15.645326	16.201687
H	-0.439635	15.992954	18.540866
C	-0.199808	16.440976	21.582048
H	1.108584	18.552435	22.705665
H	2.595443	20.194865	21.611417
H	3.067406	20.075010	19.153595
H	-2.516745	15.887517	20.203778
H	-4.368911	15.014796	21.566804
H	-6.084210	13.280097	21.990168
H	-5.040468	11.311651	18.306377
H	-3.745365	11.258439	16.679229
H	-2.820304	10.347162	14.587211
H	-2.545034	8.905164	13.130300
H	-0.624851	7.871592	9.417764
H	0.549899	10.056248	9.397648
H	0.927962	12.274208	10.393885
H	-8.184864	10.411099	21.839759
H	-8.017485	12.182552	21.771366
H	-6.770840	11.193691	22.587415
H	-7.267653	10.958046	18.273017
H	-8.266593	12.087281	19.229940
H	-8.496805	10.322977	19.399272
H	-5.515542	9.496352	19.437234
H	-6.769061	8.904018	20.556600
H	-5.269933	9.631985	21.200653
H	-4.108971	7.440647	12.494300
H	-2.754205	6.693031	13.394736
H	-3.799540	5.692627	12.356874
H	-4.007632	7.616605	9.945810
H	-3.753935	5.847899	9.907861
H	-2.622215	6.928844	9.053332
H	-0.723583	5.788784	12.128149
H	-0.679256	5.849501	10.343559
H	-1.852647	4.790931	11.167267
C	-0.548383	14.206551	9.049713
C	-1.279921	13.104170	8.601663
C	-0.679685	12.151822	7.769954
C	0.658305	12.309544	7.390565
C	1.390192	13.415115	7.834317
H	-1.024492	14.953081	9.688575
H	-2.324633	12.990665	8.898496
H	-1.251074	11.289244	7.421950
H	1.136027	11.568257	6.746356
H	2.435416	13.534514	7.540802
C	-0.050628	15.073114	21.280620
C	-0.823650	14.109912	21.932916
C	-1.763038	14.495398	22.896150
C	-1.923615	15.851928	23.201760

C	-1.148150	16.816885	22.551616
H	0.690938	14.765703	20.540187
H	-0.688571	13.053390	21.692023
H	-2.368729	13.742314	23.403853
H	-2.657551	16.161472	23.949085
H	-1.279426	17.875139	22.787030

B-2

C	-2.123032	9.288943	12.153911
C	-1.440877	10.522430	12.161809
C	-0.598300	10.859711	11.065988
C	-0.401071	9.899275	10.044633
C	-1.070664	8.689897	10.073796
C	-1.968249	8.367390	11.120757
C	-1.522061	11.453882	13.272989
C	-0.733965	12.664668	13.214330
N	-0.027354	12.999283	12.086932
C	0.038150	12.162400	11.060404
C	-2.279431	11.241564	14.415400
C	-2.221957	12.155818	15.480875
C	-1.354086	13.301942	15.362349
N	-0.658431	13.546503	14.226939
N	-1.197355	14.177000	16.384216
C	-1.906152	13.996197	17.511836
C	-2.851783	12.917720	17.690021
C	-2.969900	11.996785	16.660589
C	-3.632814	12.878178	18.914963
C	-3.406841	13.896703	19.887154
C	-2.344642	14.855027	19.647534
N	-1.653689	14.896487	18.517241
C	-4.242763	13.940872	21.024644
C	-5.218592	12.975986	21.229578
C	-5.414289	11.926100	20.306617
C	-4.621260	11.910553	19.155787
C	-6.463948	10.832913	20.510406
C	-7.251184	11.009842	21.816477
C	-2.708430	7.031931	11.087590
C	-3.635986	6.839262	12.295307
C	-7.458046	10.856891	19.329883
C	-5.753380	9.462495	20.545911
C	-3.558072	6.970228	9.800006
C	-1.678856	5.882243	11.071362
N	0.355908	15.756833	11.576918
C	0.620177	16.475272	10.419033
C	1.130469	17.728142	10.856777
N	1.181145	17.758735	12.247600
C	0.719188	16.569875	12.613081
C	1.491971	18.705920	9.920855
C	1.338607	18.397022	8.566830
C	0.835400	17.146807	8.148852
C	0.463696	16.162903	9.069017
N	0.570319	16.120822	13.916181
C	1.114845	16.740802	14.996137
N	0.839810	16.187687	16.205960
C	1.374854	16.638288	17.402596
N	0.890069	16.142858	18.580217
C	1.622658	16.762374	19.583434
C	2.545887	17.610249	18.912166
N	2.364810	17.511257	17.534995
C	3.456050	18.379918	19.647896
C	3.413834	18.279783	21.041224
C	2.488171	17.437127	21.692041
C	1.575240	16.660785	20.973366
N	1.870718	17.821858	14.875620
H	0.184975	14.725715	11.686478
H	0.118480	15.172839	14.052025
H	1.878944	19.673417	10.245344
H	1.612352	19.138905	7.813730
H	0.735300	16.941226	7.081337
H	0.086411	15.192483	8.745185
H	2.321092	18.179811	15.732522
H	1.970045	18.223771	13.929849
H	0.098238	15.433190	16.258507
H	-0.030165	15.641229	18.650227
H	0.851064	16.019243	21.476368
H	2.482860	17.391754	22.782769
H	4.113361	18.864503	21.641483
H	4.171349	19.032283	19.143921
C	-1.981633	15.871882	20.660536
H	-4.131547	14.750814	21.744046
H	-5.845487	13.043972	22.118048
H	-4.794375	11.125553	18.419665
H	-3.640289	11.138852	16.738147
H	-2.920229	10.364019	14.517930
H	-2.778730	9.038640	12.986223
H	-0.896271	7.968135	9.274445
H	0.297420	10.108751	9.236057
C	0.826206	12.632971	9.899604
H	-7.980668	10.191678	21.908152
H	-7.803152	11.962173	21.830219
H	-6.589216	10.975014	22.695349
H	-6.950774	10.668150	18.371907
H	-7.969134	11.829771	19.267539
H	-8.216313	10.072320	19.476212
H	-5.207713	9.269488	19.609838
H	-6.501304	8.665706	20.679780
H	-5.038229	9.414004	21.381807
H	-4.405540	7.625704	12.344150
H	-3.072481	6.836938	13.241468
H	-4.146878	5.869574	12.201894
H	-4.304195	7.779943	9.783501
H	-4.087955	6.006009	9.759117
H	-2.930952	7.056258	8.900333
H	-1.067194	5.893447	11.986485
H	-1.008910	5.953265	10.201974
H	-2.209129	4.918976	11.016969
C	2.066958	13.258198	10.125530
C	2.836282	13.704868	9.051714
C	2.369745	13.550160	7.741350
C	1.124561	12.955587	7.508152
C	0.356425	12.494183	8.578974
H	2.423392	13.374347	11.150239
H	3.801056	14.179756	9.237264
H	2.970704	13.906261	6.902811
H	0.745379	12.857604	6.489268
H	-0.623238	12.049795	8.396150
C	-1.791365	17.205990	20.253894
C	-1.464902	18.187026	21.190023
C	-1.306096	17.846574	22.538182
C	-1.462509	16.517177	22.946649
C	-1.802884	15.533659	22.016070
H	-1.918763	17.463538	19.201100
H	-1.330563	19.220615	20.867058
H	-1.046815	18.615268	23.268389
H	-1.312783	16.242949	23.992497
H	-1.906515	14.494558	22.332469

C

C	-4.349202	13.553543	20.918904
C	-3.536938	13.730346	19.789433
C	-3.574717	12.750620	18.766304
C	-4.386590	11.623818	18.916753
C	-5.192293	11.446597	20.053410
C	-5.168353	12.438258	21.050080
C	-2.644123	14.874470	19.721165
N	-1.831362	15.102617	18.742548
B	-1.807880	14.232338	17.581300
N	-1.024777	14.465296	16.466765
B	-1.080008	13.622692	15.343756
N	-0.394202	13.748179	14.123525
B	-0.529984	12.806848	13.121052
N	0.171409	12.877664	11.852545
C	0.052014	11.932078	10.979723
C	-0.796964	10.765310	11.153282
C	-1.589254	10.584908	12.310379
C	-2.427005	9.465740	12.415627
C	-2.497666	8.512014	11.394563
C	-1.691581	8.694404	10.250689
C	-0.862983	9.798531	10.134373
N	-1.474936	11.581744	13.336426
C	-2.156179	11.476113	14.461977
N	-2.040360	12.389237	15.462489
C	-2.803246	12.182888	16.569159
N	-2.753551	12.988969	17.612293
C	-3.416644	7.295077	11.479741
C	-2.561668	6.013247	11.389803
C	-6.048866	10.185222	20.164044
C	-5.128176	8.947619	20.114791
C	-4.227291	7.252050	12.781517
C	-4.400360	7.334679	10.290643
C	-6.854552	10.142888	21.469749
C	-7.033473	10.136886	18.976553
N	0.203148	16.960261	18.483894
C	0.580319	17.891088	19.437742
C	1.475056	18.778077	18.777979
N	1.635974	18.382175	17.458596
C	0.860538	17.305906	17.330043
C	2.044586	19.855966	19.464580
C	1.701281	20.014844	20.809819
C	0.827014	19.120517	21.454334
C	0.247127	18.023252	20.794401
C	-0.606937	17.060826	21.542863
C	-1.820364	17.492988	22.128645
C	-2.557039	16.599011	22.921643
C	-2.122374	15.288915	23.158807
C	-0.936005	14.869539	22.543117
C	-0.171292	15.728653	21.740683
C	-2.341586	18.891595	21.912807
C	1.099797	15.216220	21.115102
C	-2.897266	14.361002	24.058923
N	0.703765	16.549521	16.179827
C	1.336863	16.838262	15.006521
N	1.122997	16.001135	13.950762
C	1.667148	16.213480	12.694499
N	1.384100	15.355501	11.661651
C	2.046731	15.853617	10.552021
C	2.719738	17.024326	10.998329
N	2.458433	17.227511	12.345284
C	3.498816	17.777736	10.113460
C	3.577628	17.335801	8.790060
C	2.890470	16.186632	8.357810
C	2.092767	15.414730	9.220055
C	1.316631	14.257877	8.696015
C	-0.098852	14.297457	8.686380
C	-0.809951	13.245782	8.091372
C	-0.162836	12.151668	7.502879
C	1.236203	12.112566	7.552151
C	1.987530	13.146090	8.134058
C	-0.858794	15.447460	9.293776
C	-0.951334	11.059173	6.827761
C	3.492160	13.044073	8.153226
N	2.131947	17.895911	14.895499
H	0.915017	14.400508	11.717867
H	0.503856	15.095349	14.056815
H	4.018432	18.676745	10.449867
H	4.173764	17.898459	8.069114
H	2.954446	15.889746	7.309064
H	2.253741	18.478087	15.743739
H	2.561734	18.060132	13.967564
H	0.010977	15.696673	16.260098
H	-0.577746	16.245349	18.600644
H	0.602291	19.263578	22.513220
H	2.126714	20.842893	21.380003
H	2.731212	20.540097	18.962925
H	-2.667935	15.565250	20.574813
H	-4.310948	14.311586	21.703770
H	-5.785749	12.331894	21.941146
H	-4.408241	10.837335	18.162524
H	-3.473342	11.321798	16.561754
H	-2.836153	10.639294	14.624945
H	-3.048857	9.310244	13.294639
H	-1.725790	7.959549	9.445134
H	-0.249220	9.947026	9.243830
H	0.610841	11.995505	10.036226
H	-7.444817	9.215292	21.494783

H	-7.551161	10.992292	21.544480
H	-6.195752	10.152473	22.351585
H	-6.502997	10.114593	18.012226
H	-7.700592	11.012601	18.987381
H	-7.648898	9.226978	19.049267
H	-4.570540	8.896612	19.167253
H	-5.736984	8.034692	20.202674
H	-4.404428	8.967297	20.944272
H	-4.879782	8.134376	12.881111
H	-3.571348	7.189168	13.664300
H	-4.868243	6.358306	12.773889
H	-5.028099	8.238202	10.332396
H	-5.054602	6.450222	10.331224
H	-3.869116	7.324413	9.327720
H	-1.858822	5.949941	12.235073
H	-1.985043	5.978324	10.453976
H	-3.222870	5.133676	11.418642
H	-1.902948	13.292781	8.077570
H	1.763818	11.253979	7.125822
H	-0.583802	13.845627	22.700445
H	-3.496775	16.941416	23.365471
H	3.810150	12.003993	7.992104
H	3.944516	13.660245	7.359824
H	3.905151	13.405392	9.106522
H	-0.362851	10.133142	6.745092
H	-1.880624	10.839137	7.375501
H	-1.238301	11.361335	5.806722
H	-1.885337	15.483919	8.902704
H	-0.916022	15.344920	10.390240
H	-0.362304	16.408157	9.091280
H	1.440177	14.301583	21.620320
H	0.945990	14.979475	20.049064
H	1.899804	15.970318	21.159137
H	-3.414031	18.945161	22.149676
H	-1.818237	19.616588	22.556261
H	-2.183933	19.220609	20.875030
H	-3.954620	14.659005	24.126272
H	-2.844957	13.321088	23.702419
H	-2.485186	14.377958	25.081798

D-1

C	6.230013	4.547687	18.181906
N	5.491890	3.773211	19.049736
B	4.038945	3.572818	18.799192
N	3.449482	4.218865	17.657066
C	4.224863	4.937529	16.876074
C	5.625223	5.129348	17.100572
C	6.076342	3.173920	20.145435
C	5.320267	2.398629	20.980658
C	3.926154	2.199738	20.710293
N	3.288528	2.737557	19.695750
N	0.694475	3.673805	17.188892
C	0.066382	4.655266	16.456565
C	-1.227187	4.475460	15.987285
C	-1.896522	3.119678	16.137242
C	-1.265625	2.281545	17.238590
C	0.037342	2.551166	17.659390
C	-1.868386	2.356956	14.814743
C	-3.053791	1.947692	14.203542
C	-2.993489	1.234391	13.003237
C	-1.781566	0.917109	12.382174
C	-0.599711	1.331262	12.999347
C	-0.645167	2.042544	14.203111
N	-4.254226	0.799075	12.372327
O	-5.318051	1.034163	12.965463
C	-1.894989	5.533491	15.273734
O	-1.438624	6.666286	15.015641
N	0.832740	5.761534	16.211583
N	0.754063	1.777537	18.504591
C	-1.967617	1.139345	17.754959
O	-1.542128	0.306730	18.582383
O	-3.150183	5.166130	14.868518
C	-3.882647	6.134379	14.075863
C	-5.178596	5.472901	13.651261
O	-3.227749	1.024060	17.220545
C	-3.987159	-0.148819	17.603288
C	-5.298988	-0.098413	16.843865
O	-4.193813	0.216004	11.278539
H	6.213861	5.738226	16.415266
H	5.785337	1.925216	21.844890
H	3.363987	1.552543	21.398046
H	3.773994	5.423233	15.998630
H	1.715133	3.818677	17.403827
H	-2.954643	3.281028	16.385206
H	1.601488	2.145390	18.976060
H	0.234740	0.987695	18.898323
H	-3.404405	-1.049600	17.353345
H	-4.144397	-0.139171	18.693358
H	-5.906327	-0.979681	17.100760
H	-5.869783	0.804665	17.107438
H	-5.125537	-0.097804	15.757414
H	0.307406	6.539152	15.796974
H	1.554103	5.984449	16.892872
H	-4.061620	7.036047	14.682769
H	-3.268253	6.425167	13.209348
H	-5.765464	6.174542	13.039338
H	-4.979491	4.570949	13.053652
H	-5.780046	5.188226	14.527345
H	0.283446	2.362268	14.682549
H	0.360846	1.095317	12.537902
H	-1.775725	0.359995	11.446377
H	-4.015169	2.178402	14.659279
H	7.143859	3.351224	20.292990
H	7.292936	4.661748	18.405760

D-2

C	39.683097	11.733559	7.225928
C	38.412314	11.828470	6.629320
N	37.293662	11.810437	7.428516
C	37.367277	11.704548	8.797239
C	38.629771	11.604379	9.410221
C	39.769120	11.621303	8.610614
N	38.236068	11.914236	5.296236
N	36.215092	11.727146	9.496427
N	33.622087	11.425072	8.390166
C	32.510373	11.197730	9.009346
C	31.201008	11.166872	8.382444
C	31.054454	11.406861	6.992527
N	32.249904	11.679766	6.253540
B	33.611315	11.683724	6.955669
C	30.064272	10.893571	9.167228
C	28.798364	10.853422	8.597459
C	28.662315	11.089515	7.222049
C	29.772958	11.363154	6.423740
C	32.185937	11.932990	4.942709
N	33.262666	12.187924	4.193069
B	34.648989	12.194052	4.844366
N	34.763445	11.935711	6.210685
N	35.789720	12.473029	3.981393
C	35.600924	12.707259	2.724352
C	34.306755	12.715912	2.065533
C	33.119154	12.449856	2.793043
C	31.889449	12.452873	2.117258
C	31.844692	12.715682	0.748080
C	33.014497	12.980547	0.021744
C	34.235428	12.978336	0.684159
H	32.526287	11.008800	10.094867
H	30.203959	10.712694	10.235500
H	27.920211	10.640677	9.208304
H	27.675152	11.059423	6.758475
H	29.601609	11.535174	5.362297
H	31.212277	11.933096	4.463703
H	30.946585	12.253116	2.623882
H	30.877865	12.711434	0.242159
H	32.963153	13.182686	-1.048817
H	35.164994	13.178037	0.146251
H	36.468908	12.914102	2.077701
H	36.316479	11.872709	6.960639
H	35.266662	11.611513	9.062121
H	36.289210	11.568141	10.495999
H	38.687820	11.515128	10.494129
H	40.752032	11.544861	9.080159
H	40.569117	11.750808	6.592840
H	37.322029	12.154842	4.840701
H	39.074337	11.994572	4.730409

D-3

C	8.216831	2.162123	21.135727
C	7.586210	3.097092	20.315135
C	6.244161	2.917838	19.944115
C	5.546190	1.776323	20.412734
C	6.204341	0.847372	21.240365
C	7.532146	1.031799	21.604408
C	4.155785	1.551145	20.049245
N	3.452862	2.329132	19.296654
B	4.048972	3.541774	18.747236
N	5.516748	3.822849	19.107959
N	3.363380	4.438634	17.932401
B	4.016327	5.577818	17.469640
N	5.489146	5.806723	17.845979
C	6.111144	4.920491	18.629521
C	6.191258	6.955726	17.362027
C	5.459873	7.858728	16.549853
C	4.061235	7.612670	16.234630
N	3.381422	6.591496	16.635057
C	6.092873	9.007444	16.039399
C	7.428048	9.269988	16.319294
C	8.145622	8.371929	17.122568
C	7.540547	7.226725	17.639715
N	0.696176	5.839729	15.877377
C	-0.013207	4.749791	16.225993
N	0.633308	3.836979	17.043789
C	-0.026350	2.766491	17.626241
C	-1.335083	2.447434	17.244492
C	-1.993094	3.183956	16.093001
C	-1.327392	4.516183	15.802414
N	0.660472	2.087359	18.566048
C	-1.995782	5.472932	14.968591
O	-3.266038	5.068010	14.628370
C	-3.992119	5.927041	13.717518
C	-5.308367	5.239709	13.409650
C	-1.988868	2.314379	14.837024
C	-3.184756	1.950330	14.218178
C	-3.147266	1.157670	13.067456
C	-1.946334	0.709293	12.508329
C	-0.753676	1.078828	13.132769
C	-0.777049	1.873242	14.284415
C	-2.001603	1.320813	17.831112
O	-3.263116	1.139443	17.313598
C	-3.980499	-0.033268	17.765222
C	-5.298013	-0.066686	17.014298
N	-4.418275	0.784519	12.420868
O	-4.378264	0.053421	11.417834
O	-5.473772	1.219612	12.907005
O	-1.544002	6.562937	14.552207
O	-1.554115	0.543681	18.703476
H	3.696532	0.638439	20.463180
H	1.608572	4.061878	17.372280
H	-3.047945	3.363595	16.344285
H	1.662125	2.247957	18.784045
H	0.169463	1.267933	18.938051
H	-3.371477	-0.928294	17.560729
H	-4.131803	0.028851	18.854724
H	-5.876856	-0.950686	17.323125
H	-5.894566	0.832290	17.229337
H	-5.128886	-0.121370	15.928582
H	0.190112	6.501827	15.280226
H	-4.144462	6.911220	14.189389
H	-3.388842	6.080149	12.808562
H	-5.894221	5.859887	12.714167
H	-5.140168	4.256797	12.944320
H	-5.898763	5.094428	14.326710
H	0.159294	2.158910	14.770036
H	0.198198	0.743930	12.716615
H	-1.957889	0.092524	11.610777

H	-4.136657	2.282342	14.629089
H	7.149225	5.101064	18.890344
H	1.647383	6.059291	16.230009
H	3.573974	8.373333	15.602406
H	5.507048	9.686919	15.415746
H	7.913256	10.161334	15.919793
H	9.195580	8.561651	17.351313
H	8.151292	6.566114	18.253473
H	5.643846	-0.023608	21.588678
H	8.036696	0.308287	22.245969
H	8.170912	3.954410	19.984430
H	9.260514	2.322574	21.410977

D-4

C	-3.395492	13.248189	20.291363
C	-3.440110	12.410476	19.186512
N	-4.097006	11.261600	19.537634
N	-4.461499	11.346024	20.805172
N	-4.037643	12.541574	21.254302
N	-2.902648	12.665400	17.924423
C	-3.082799	11.858891	16.847915
N	-3.811625	10.744614	16.936600
C	-4.235401	12.930808	22.649228
C	-2.908192	13.066773	23.395096
C	-3.117419	13.476933	24.853015
C	-1.798490	13.621729	25.615866
C	-2.006102	14.034415	27.074541
N	-2.501879	12.219443	15.675387
C	-2.578630	11.466779	14.502684
C	-1.913951	11.728060	13.314158
N	-2.323293	10.729342	12.493613
N	-3.178510	9.890097	13.107131
N	-3.339928	10.341222	14.339718
C	-1.916785	10.509377	11.105897
C	-2.089198	11.767587	10.256630
C	-1.587355	11.556416	8.827957
C	-1.711734	12.818068	7.970222
C	-1.211213	12.606817	6.539922
N	-0.277504	14.247751	12.923570
B	-0.217433	14.924404	14.214554
N	0.628812	16.258071	14.225288
C	1.335729	16.690083	13.051919
C	1.147821	15.926468	11.876892
C	0.325250	14.728773	11.891192
C	0.658672	16.997463	15.313703
N	0.058595	16.609291	16.467060
B	-0.792716	15.278057	16.569364
N	-0.849975	14.518143	15.380092
N	-1.420164	15.071829	17.819043
B	-1.277309	15.967056	18.868935
N	-0.285795	17.179411	18.707186
C	0.225682	17.444898	17.523874
C	0.000014	18.043221	19.812724
C	-0.804505	17.895017	20.961772
C	-1.804015	16.841346	21.034995
N	-2.011963	15.946988	20.129280
C	-0.585066	18.754308	22.052854
C	0.413300	19.715964	22.011271
C	1.254932	19.839233	20.885107
C	1.039405	18.983786	19.798303
C	2.193925	17.793339	13.027038
C	2.849114	18.187082	11.849264
C	2.635095	17.435173	10.680205
C	1.803990	16.320693	10.701190
C	2.369503	20.884988	20.890943
C	1.733297	22.282739	21.052190
C	3.765481	19.410693	11.889090
C	2.930389	20.646037	12.288546
C	4.873620	19.185145	12.938533
C	4.428999	19.686435	10.532925
C	3.308494	20.615630	22.086506
C	3.201702	20.872585	19.602229
H	-2.974799	14.250427	20.423334
H	-2.342817	13.581307	17.838164
H	-3.932681	10.176540	16.085973
H	-4.191027	10.487873	17.858887
H	-1.890812	13.099689	15.610916
H	-1.246359	12.545505	13.022143
H	0.246077	14.200698	10.924067
H	1.652907	15.726111	9.796945
H	3.130878	17.715012	9.751785
H	2.398391	18.372107	13.926695
H	1.176559	17.957971	15.318508
H	0.797619	18.361075	17.361295
H	1.718902	19.046572	18.950198
H	0.561411	20.374685	22.868327
H	-1.213609	18.644468	22.940151
H	-2.396417	16.814370	21.965942
H	5.076095	20.570985	10.626304
H	3.682074	19.892113	9.751085
H	5.053740	18.839398	10.210272
H	2.471355	20.516980	13.280730
H	2.129883	20.829576	11.555812
H	3.582309	21.532461	12.324971
H	4.454896	19.042876	13.946122
H	5.533678	20.065590	12.966103
H	5.479014	18.301861	12.684344
H	2.581868	21.092975	18.718074
H	3.703389	19.903405	19.453320
H	3.978904	21.647727	19.669846
H	1.061860	22.506903	20.209585
H	2.528745	23.043986	21.078221
H	1.157567	22.357053	21.986405
H	3.779387	19.624366	22.000045
H	2.765238	20.660825	23.041766
H	4.101516	21.379402	22.106639
H	-0.865534	10.180503	11.100926
H	-2.538292	9.685143	10.731042
H	-3.153176	12.055560	10.247107
H	-1.534293	12.600077	10.721038
H	-0.530392	11.235347	8.853402
H	-2.152985	10.735702	8.353398
H	-2.767003	13.140928	7.954699
H	-1.143577	13.636019	8.448508
H	-1.308595	13.522977	5.937924
H	-0.150434	12.307542	6.537329
H	-1.784834	11.809750	6.039966
H	-4.863966	12.149531	23.098211
H	-4.794151	13.878513	22.666841
H	-2.370932	12.105420	23.348712
H	-2.277187	13.814848	22.885334
H	-3.669790	14.432801	24.893144
H	-3.750803	12.727143	25.359124
H	-1.251603	12.664345	25.570479
H	-1.165376	14.367325	25.103360
H	-1.047528	14.130903	27.606816
H	-2.529369	15.002510	27.135626
H	-2.617708	13.289253	27.608503

E-1

C	1.249799	19.813781	19.743602
C	0.723097	18.783739	18.931044
C	-0.197998	17.863749	19.499039
C	-0.563395	18.001512	20.853182
C	-0.047921	19.019535	21.652922
C	0.870555	19.926427	21.068490
C	1.070672	18.656387	17.532980
N	0.586556	17.703879	16.757102
C	-0.260777	16.749834	17.274110
C	-0.711894	16.806886	18.645946
C	-1.622474	15.844791	19.057716
C	-2.031088	14.833616	18.171252
C	-1.463093	14.808037	16.845707
N	-0.620737	15.779063	16.417480
N	-1.748723	13.810156	15.973493
C	-2.631180	12.864317	16.335573
C	-3.314678	12.859549	17.608721
C	-2.969607	13.845681	18.520073
C	-4.305354	11.824572	17.856914
C	-4.544711	10.874724	16.824188
C	-3.763300	10.947927	15.608636
N	-2.852365	11.879713	15.397535
C	-5.036669	11.731727	19.051961
C	-5.994168	10.733859	19.260469
C	-6.221885	9.804455	18.222174
C	-5.514578	9.872111	17.030470
C	2.016281	19.633517	16.902971
C	-3.965829	9.934996	14.522832
C	-6.746301	10.681825	20.591100
C	-7.773880	9.543060	20.640951
C	-0.432920	19.189608	23.121009
C	-1.427931	18.123426	23.598503
C	0.841361	19.095834	23.987824
C	-1.073052	20.581613	23.310913
C	-5.725973	10.475733	21.731455
C	-7.485403	12.019089	20.809367
N	0.516089	18.188853	13.985767
C	0.501584	17.111721	13.144268
N	0.843909	17.382982	11.892205
C	1.123587	18.746251	11.905357
C	0.929555	19.267603	13.214681
C	1.130076	20.616700	13.515679
C	1.544548	21.442924	12.465962
C	1.742810	20.937874	11.164169
C	1.533334	19.589256	10.865006
N	0.126187	15.871275	13.636803
C	0.251087	14.711281	12.938963
N	0.790080	14.676143	11.730280
N	-0.208847	13.588887	13.552472
C	-0.114068	12.317815	13.007091
N	0.584228	11.979632	11.931639
C	0.365919	10.611054	11.802814
C	-0.487341	10.156757	12.846692
N	-0.767937	11.281065	13.611901
C	0.854718	9.708656	10.850111
C	0.471946	8.369801	10.965303
C	-0.373062	7.931819	12.006121
C	-0.866223	8.817927	12.969344
H	0.510773	18.087703	15.033969
H	-0.165196	15.821780	14.652528
H	1.681162	19.199437	9.856491
H	2.062763	21.618709	10.373267
H	1.713479	22.503918	12.658907
H	0.963823	21.014940	14.517159
H	0.911292	13.747045	11.295648
H	1.054959	15.579927	11.306318
H	-0.765036	13.690947	14.446970
H	-1.541235	11.430064	14.311573
H	-1.507362	8.472238	13.781011
H	-0.644697	6.876325	12.065356
H	0.838209	7.643548	10.237472
H	1.513606	10.046140	10.048212
H	-5.710902	9.138213	16.248469
H	-6.963249	9.016602	18.351444
H	-4.860560	12.456895	19.846960
H	-3.414310	13.878929	19.516183
H	-2.036619	15.849799	20.067215
H	-1.265188	17.292729	21.289583
H	1.288261	20.731400	21.675107
H	1.958299	20.528185	19.323407
H	-8.280873	9.560961	21.616906
H	-7.293937	8.559121	20.524824
H	-8.538986	9.656305	19.857287
H	-4.993593	11.296601	21.767398
H	-5.181071	9.528134	21.601604
H	-6.255880	10.445699	22.696045
H	-6.785860	12.868094	20.831473
H	-8.016885	11.988805	21.773123
H	-8.220842	12.194605	20.009420
H	-1.006498	17.109845	23.509795
H	-2.370250	18.166674	23.030083
H	-1.662888	18.301056	24.658288
H	1.319144	18.110218	23.875469
H	0.572382	19.235090	25.046334
H	1.572159	19.870914	23.714708

H -1.987057 20.677175 22.705036
H -0.379169 21.385907 23.025448
H -1.339696 20.718740 24.370455
H 1.604140 20.654782 16.939808
H 2.968133 19.654423 17.456141
H 2.222503 19.362233 15.861664
H -3.334528 10.160051 13.655834
H -3.738942 8.920471 14.887883
H -5.018278 9.928585 14.198928

E-2

C 0.000000 0.000000 0.000000
C 0.000000 0.000000 1.394563
C 1.228039 0.000000 2.119485
C 2.455587 -0.033280 1.449375
C 2.433606 -0.059705 0.054466
C 1.224499 -0.040106 -0.669692
N 0.962011 0.044039 3.481059
C -0.360986 0.063354 3.559927
N -1.002935 0.049932 2.349098
N -1.101890 0.125359 4.728127
C -0.557910 0.036966 5.972868
N 0.732255 -0.189837 6.156701
I -1.820437 0.128993 -1.101034
N -1.415557 0.194608 7.018055
C -1.045177 0.049309 8.344528
N -1.950642 0.326932 9.335157
C -1.293011 0.068555 10.526664
C 0.011924 -0.376670 10.162431
N 0.131616 -0.378568 8.779469
C 0.942869 -0.740342 11.141284
C 0.546698 -0.658766 12.476760
C -0.747663 -0.238459 12.843599
C -1.678106 0.118528 11.866026
I -3.663365 0.660871 12.420639
N -4.067530 2.054689 8.618335
C -4.780439 1.736334 7.481692
C -6.177424 2.090998 7.335749
C -6.810722 2.871667 8.385302
C -6.002383 3.246694 9.491263
C -4.652058 2.784400 9.541982
C -6.554944 4.019739 10.536589
C -7.880895 4.407146 10.482124
C -8.711193 4.039403 9.391092
C -8.158358 3.279480 8.358640
N -4.082876 1.088522 6.537007
C -4.709876 0.721835 5.395803
C -6.123481 0.935876 5.207658
C -6.830223 1.645036 6.194905
N -3.945666 0.145739 4.438489
C -4.535625 -0.307919 3.323353
C -5.964716 -0.241434 3.097510
C -6.728281 0.423232 4.045667
C -6.509709 -0.855993 1.897219
C -5.587131 -1.481485 1.012762
C -4.193413 -1.422146 1.321661
N -3.679369 -0.858282 2.391226
C -6.053494 -2.105180 -0.161655
C -7.409520 -2.113841 -0.454579
C -8.346062 -1.500739 0.410318
C -7.874720 -0.881341 1.574918
C -10.171860 4.486853 9.387535
C -10.222078 6.029521 9.419012
C -9.847515 -1.494596 0.118095
C -10.333015 -0.032548 0.015058
C -10.197141 -2.218121 -1.190027
C -10.587954 -2.194730 1.277564
C -10.938264 3.997578 8.150758
C -10.867979 3.929271 10.647770
H -2.796942 0.931391 9.170614
H -2.406185 0.520542 6.820590
H 1.940381 -1.081610 10.861783
H 1.246948 -0.933717 13.267169
H -1.025833 -0.205705 13.897088
H 1.327649 -0.237971 5.313714
H 1.056223 -0.308870 7.130278
H -2.158165 0.151948 4.635641
H -2.014367 -0.227542 2.251923
H 1.248686 -0.042448 -1.759466
H 3.371888 -0.086609 -0.501996
H 3.393551 -0.033062 2.005993
H -3.484113 -1.860893 0.613020
H -5.335988 -2.578000 -0.835844
H -7.752138 -2.600569 -1.366923
H -8.597583 -0.413025 2.243811
H -7.803508 0.556154 3.912842
H -7.893878 1.836445 6.042250
H -8.786406 2.998543 7.514389
H -8.298559 5.003452 11.294391
H -5.924749 4.301065 11.383178
H -4.049308 3.031239 10.421601
H -11.286144 -2.177380 -1.340126
H -9.896541 -3.276590 -1.158611
H -9.716392 -1.740864 -2.057873
H -10.412827 -1.683197 2.235799
H -10.260298 -3.241008 1.375827
H -11.670303 -2.185209 1.076184
H -10.144625 0.521459 0.946959
H -11.416700 -0.021299 -0.178663
H -9.825368 0.490897 -0.809665
H -10.952267 2.897751 8.092509
H -10.502337 4.395417 7.221166
H -11.979272 4.347125 8.214088
H -10.847505 2.828216 10.651503
H -11.918376 4.258636 10.658319
H -10.385930 4.289770 11.568166
H -9.736289 6.454179 8.527002
H -9.722300 6.430451 10.312960
H -11.272272 6.359493 9.435403

E-3

C 1.350550 16.919378 20.993747
C 1.413958 16.960970 19.601544
C 2.348586 17.812840 18.945349
C 3.205913 18.637774 19.682168
C 3.113357 18.592764 21.073969
C 2.199701 17.744404 21.731197
N 2.236309 17.648048 17.571651
C 1.278505 16.742873 17.423315
N 0.747872 16.280102 18.597654
N 0.821237 16.237323 16.217118
C 1.210925 16.718240 15.004629
N 2.012708 17.764235 14.895322
Br 0.144205 15.720277 21.881258
N 0.730766 16.065150 13.910514
C 0.991010 16.461720 12.608390
N 0.582697 15.674950 11.564626
C 0.982432 16.340190 10.418482
C 1.615526 17.537984 10.859584
N 1.600682 17.582636 12.246915
C 2.130018 18.457295 9.938381
C 1.997814 18.159979 8.581203
C 1.361532 16.983833 8.136407
C 0.844234 16.072793 9.057037
Br -0.074649 14.498487 8.458622
N -1.753435 15.009469 18.463205
C -1.962789 14.065861 17.477465
C -2.925914 12.997322 17.646221
C -3.744998 12.976830 18.847861
C -3.533918 14.017441 19.794847
C -2.511548 14.980602 19.535956
C -2.991853 12.038468 16.647035
C -2.194907 12.162534 15.495169
C -1.342804 13.319570 15.368604
N -1.217715 14.219886 16.372760
N -0.630103 13.554686 14.241859
C -0.663569 12.647634 13.253463
C -1.403594 11.404767 13.336836
C -2.189466 11.208647 14.463214
N 0.069672 12.974277 12.131594
C 0.164787 12.092242 11.161939
C -0.467020 10.811966 11.150412
C -1.297821 10.452401 12.244420
C -0.316201 9.926212 10.060276
C -0.978038 8.712639 10.060766
C -1.814880 8.330385 11.141682
C -1.957491 9.208438 12.217655
C -4.298679 14.055662 20.977976
C -5.256534 13.081768 21.219879
C -5.485756 12.038413 20.292478
C -4.723137 12.008446 19.118026
C -2.529409 6.981237 11.083759
C -1.477858 5.858310 10.955632
C -6.536590 10.951855 20.526014
C -5.848279 9.570003 20.505268
C -7.263609 11.113147 21.868369
C -7.580667 11.014660 19.390459
C -3.384094 6.709585 12.329369
C -3.448548 6.959957 9.843383
H 0.330690 14.656708 11.684567
H 0.214271 15.150824 14.048841
H 2.613441 19.374503 10.276911
H 2.388071 18.853473 7.834808
H 1.262998 16.787938 7.068555
H 2.387492 18.164204 15.771812
H 2.215819 18.103620 13.940117
H 0.075143 15.486368 16.253999
H -0.172163 15.764929 18.643953
H 2.161884 17.723338 22.820497
H 3.766851 19.221935 21.680221
H 3.923112 19.287065 19.178655
H -2.317924 15.751502 20.288607
H -4.126269 14.857081 21.699780
H -5.836628 13.125546 22.140887
H -4.904043 11.207166 18.400835
H -3.656952 11.177210 16.732629
H -2.811149 10.317990 14.569938
H -2.592669 8.920224 13.054066
H -0.857773 8.036609 9.213319
H 0.322780 10.214194 9.222590
H 0.764277 12.386944 10.294516
H -7.996006 10.299725 21.977359
H -7.805630 12.069593 21.922788
H -6.563637 11.056279 22.716343
H -7.114861 10.856091 18.406226
H -8.090667 11.990100 19.381361
H -8.334438 10.227313 19.545292
H -5.353215 9.379843 19.541230
H -6.603483 8.784922 20.663675
H -5.094843 9.495939 21.304497
H -4.174151 7.467096 12.450583
H -2.770641 6.688362 13.243659
H -3.868952 5.727951 12.223003
H -4.217089 7.745406 9.913171
H -3.952149 5.982965 9.780811
H -2.877436 7.112189 8.915827
H -0.812523 5.843922 11.832651
H -0.862933 5.981931 10.052372
H -1.990624 4.886247 10.890702

E-4

C -4.430398 11.766700 19.291910
C -3.465481 12.745825 19.010420
C -3.149713 13.701021 20.016238
C -3.794929 13.642820 21.267360
C -4.740987 12.660296 21.518735
C -5.078642 11.704763 20.531891
C -2.168113 14.696986 19.727017
N -1.538843 14.820476 18.581359
C -1.812812 13.936840 17.557493
C -2.756025 12.853078 17.744608
C -2.905298 11.958200 16.697674
C -2.195951 12.150127 15.499411
C -1.337162 13.305788 15.375568
N -1.146323 14.158285 16.413166

C	-2.290052	11.256953	14.419201
C	-1.588331	11.503684	13.249422
C	-0.814348	12.727368	13.190112
N	-0.686338	13.581714	14.218653
C	-1.603637	10.621968	12.093745
C	-0.875865	11.039314	10.948224
C	-0.189657	12.290112	10.993755
N	-0.151627	13.088264	12.036088
C	-0.852482	10.226082	9.793712
C	-1.535259	9.024476	9.781673
C	-2.268553	8.583652	10.914141
C	-2.289605	9.392390	12.051617
C	-2.999830	7.244102	10.846294
C	-4.017855	7.286558	9.686620
C	-6.128911	10.618869	20.771697
C	-7.272647	10.797427	19.750116
C	-1.969050	6.126581	10.575425
C	-3.750542	6.912767	12.143192
C	-5.481455	9.232426	20.568990
C	-6.724117	10.676863	22.185599
N	0.903345	15.585740	11.578180
C	1.245841	16.412586	12.604653
N	1.932938	17.489157	12.237081
C	2.068061	17.358719	10.858581
C	1.431270	16.165709	10.437932
C	2.703455	18.196354	9.927456
C	2.676696	17.797021	8.593018
C	2.042711	16.605604	8.171028
C	1.405166	15.767523	9.092692
N	0.869503	16.089272	13.904096
C	1.273983	16.793965	14.996046
N	2.063905	17.848918	14.884151
O	0.756769	14.594261	8.804861
C	0.712258	14.207936	7.421900
N	0.828723	16.364194	16.208286
C	1.172931	16.976077	17.408994
N	2.051462	17.962154	17.557729
C	2.043455	18.228789	18.923056
C	1.127453	17.358967	19.563186
N	0.591275	16.560635	18.568179
C	2.773272	19.170876	19.666194
C	2.551052	19.204656	21.041135
C	1.635949	18.337959	21.682284
C	0.906999	17.395478	20.948475
O	-0.001977	16.503376	21.455890
C	-0.215021	16.540637	22.875961
H	0.474540	14.624731	11.709696
H	0.307153	15.213190	14.041049
H	3.194949	19.117874	10.241226
H	3.159694	18.418660	7.837007
H	2.054855	16.345133	7.113242
H	2.361473	18.306229	15.764884
H	2.334773	18.130549	13.924555
H	0.141418	15.571668	16.250286
H	-0.219432	15.878590	18.615834
H	1.504713	18.410105	22.761500
H	3.097939	19.921964	21.655714
H	3.482181	19.843647	19.182583
H	-1.891465	15.418314	20.503961
H	-3.543644	14.380147	22.033289
H	-5.229253	12.630313	22.492071
H	-4.694750	11.033152	18.529348
H	-3.564132	11.091886	16.781682
H	-2.925256	10.375412	14.523336
H	-2.845214	9.058422	12.926764
H	-1.509857	8.402238	8.886165
H	-0.288652	10.556853	8.918558
H	0.344096	12.641868	10.103614
H	-7.466763	9.872801	22.293724
H	-7.231794	11.635888	22.371354
H	-5.951007	10.532207	22.955872
H	-6.904694	10.716606	18.716210
H	-7.756799	11.778454	19.872150
H	-8.028355	10.012679	19.909087
H	-5.083295	9.117980	19.549595
H	-6.238817	8.449878	20.730526
H	-4.659676	9.076152	21.284211
H	-4.519215	7.668247	12.368820
H	-3.063733	6.839378	13.000964
H	-4.252435	5.940761	12.027474
H	-4.768006	8.074478	9.854215
H	-4.536556	6.317534	9.622890
H	-3.523574	7.474127	8.722045
H	-1.228883	6.071736	11.388718
H	-1.434966	6.292441	9.628261
H	-2.490079	5.158899	10.511570
H	-0.956276	15.760621	23.088083
H	0.719487	16.323415	23.418451
H	-0.603449	17.523339	23.189733
H	0.149807	13.266917	7.391847
H	0.193969	14.970949	6.818480
H	1.728293	14.050777	7.024054

Coordinates of complexes of designed anions with zirconocene cation

12a

C	-0.055788	0.416429	-0.253904
C	-0.107255	0.433415	1.169367
C	1.152274	0.585162	1.826778
C	2.311769	0.868163	1.082749
C	2.331676	1.010368	-0.300707
C	1.121693	0.760139	-0.941243
B	-1.551467	0.594940	2.046724
N	-1.213458	1.816717	2.961192
C	-0.949523	2.758337	3.600235
N	-0.801088	3.790266	4.332926
C	0.236106	4.418758	4.707573
N	1.111829	5.062648	5.143370
B	2.305277	5.882271	5.729225
C	1.625119	6.430822	7.191384
C	0.272791	6.889243	7.222910
C	-0.434054	6.932011	8.438311
C	0.095926	6.533522	9.663066
C	1.439874	6.178595	9.637381
C	2.219563	6.192151	8.467463
C	-0.617894	7.327450	6.047274
F	-1.655809	6.448592	5.891659
F	-1.720023	7.319684	8.445100
C	-0.799910	6.539941	10.891037
F	-1.907343	5.777134	10.682285
F	2.003926	5.754570	10.785064
C	3.689378	5.884595	8.754840
F	4.147112	6.637704	9.791460
C	1.465641	0.518045	3.324814
F	2.448825	-0.385246	3.570752
F	3.480056	1.067303	1.722818
C	3.628880	1.411397	-0.981663
F	4.597547	0.481254	-0.752696
F	1.075016	0.890807	-2.275544
C	-1.205334	0.092157	-1.211686
F	-1.633924	1.213550	-1.865954
C	-2.893522	1.217300	1.205043
C	-2.893688	2.563871	0.729909
C	-4.103483	3.216572	0.406198
C	-5.349177	2.611476	0.512525
C	-5.329260	1.272744	0.899007
C	-4.157919	0.555682	1.169135
C	-1.694885	3.504178	0.524365
F	-0.468962	2.968509	0.699953
C	-4.422990	-0.921334	1.451265
F	-4.996213	-1.096028	2.682545
F	-6.524911	0.673839	1.047384
C	-6.685019	3.281522	0.245689
F	-7.462101	3.259625	1.364608
F	-4.061496	4.501327	0.020238
C	-1.829642	-0.626204	3.205527
C	-1.163363	-1.889946	3.171204
C	-1.013220	-2.646547	4.339680
C	-1.585813	-2.310782	5.565556
C	-2.375741	-1.167762	5.553810
C	-2.514231	-0.334970	4.423145
C	-0.519288	-2.565009	1.958806
F	-0.858278	-2.045788	0.767997
F	-0.249559	-3.753907	4.320869
C	-1.348309	-3.213910	6.762238
F	-0.012283	-3.384555	6.979635
F	-2.997338	-0.821229	6.694161
C	-3.422560	0.863431	4.722430
F	-3.731091	1.655637	3.677757
F	0.846470	-2.532157	2.043798
F	-0.886549	-3.877103	1.891165
F	-1.877362	-2.745757	7.911342
F	-1.878080	-4.449053	6.544883
F	-4.615073	0.452878	5.227773
F	-2.840577	1.681869	5.665898
F	-5.281929	-1.447008	0.532669
F	-3.334849	-1.710253	1.402509
F	-6.583737	4.568973	-0.143208
F	-7.364469	2.619149	-0.731283
F	-1.689347	4.000348	-0.742694
F	-1.764528	4.570402	1.383016
C	2.541484	7.046339	4.514621
C	2.566053	8.447036	4.781567
C	2.244948	9.368540	3.775827
C	1.982580	9.016499	2.453205
C	2.109309	7.662630	2.163513
C	2.409115	6.689427	3.139104
C	2.859761	9.119467	6.120251
F	3.407674	8.317263	7.052478
F	2.128831	10.672381	4.085281
C	1.586205	10.099536	1.464267
F	2.564851	11.041098	1.364331
F	1.890395	7.261159	0.902250
C	2.520766	5.291174	2.520599
F	1.311563	4.859380	2.039858
C	3.470607	4.650638	5.887093
C	4.780720	4.720231	5.340801
C	5.525815	3.555359	5.080191
C	5.114939	2.282194	5.470426
C	3.889020	2.229461	6.117259
C	3.074282	3.353634	6.345580
C	5.506686	5.994553	4.897481
F	4.990880	7.133365	5.388299
F	6.665479	3.659667	4.387995
C	5.885856	0.990519	5.245421
F	5.170121	0.139531	4.465910
F	3.462856	1.011456	6.512055
C	1.802299	2.974702	7.062059
F	1.055114	2.066388	6.412304
F	3.748369	10.140746	5.963354
F	1.722887	9.650153	6.665780
F	1.347874	9.637135	0.220259
F	0.455640	10.734654	1.882323
F	3.391176	5.292054	1.476980
F	2.961577	4.319230	3.349803
F	6.802757	5.981219	5.317416
F	5.517929	6.100185	3.536693
F	2.210576	2.290935	8.315375
F	0.982960	3.907810	7.518049
F	7.083962	1.172132	4.660026
F	6.108854	0.365217	6.436761
F	-1.149384	8.555116	6.287524
F	-0.024218	7.424397	4.840949
F	-0.195927	6.067434	12.003322
F	-1.222083	7.803498	11.164797
F	3.853826	4.563878	9.115680
F	4.536511	6.119607	7.741894
F	1.915714	1.741471	3.771588
F	0.457315	0.179669	4.159854
F	3.519238	1.552691	-2.317997
F	4.080729	2.596581	-0.486393
F	-0.796051	-0.799962	-2.160015
F	-2.287009	-0.471349	-0.645610

Zr	1.138617	1.099239	9.966361
C	-0.282489	2.777370	10.452926
C	0.518160	-0.431948	8.066301
C	1.771315	1.733834	12.309132
C	1.866105	0.316849	12.192572
C	2.936327	0.016930	11.293473
C	3.489236	1.248188	10.853656
C	2.763838	2.308191	11.469448
H	2.928267	3.371631	11.308776
H	4.305702	1.363502	10.142444
H	3.267971	-0.975857	11.000957
H	1.255221	-0.410454	12.721189
C	0.761432	-1.234247	9.211188
C	-0.249542	-0.936230	10.174959
C	-1.117314	0.044493	9.616333
C	-0.634182	0.366278	8.320298
H	-1.076888	1.092984	7.643538
H	-1.986787	0.481827	10.098393
H	-0.357776	-1.403976	11.149778
H	1.564969	-1.956561	9.326424
H	-0.994655	2.573104	11.265883
H	0.309842	3.658507	10.747235
H	-0.873068	3.000204	9.547777
H	1.126890	-0.415982	7.164370
H	1.052513	2.279184	12.913753

12b

C	0.268884	-3.122669	15.852181
N	0.989816	-4.233170	16.312455
C	0.423631	-4.581861	17.542237
C	-0.649364	-3.737418	17.814915
C	-0.698370	-2.770409	16.780924
B	2.314399	-4.734462	15.593882
N	3.392244	-5.443234	16.498404
C	3.851010	-6.743662	16.329055
C	5.140523	-6.852669	16.833695
C	5.462410	-5.597888	17.421059
C	4.365618	-4.769358	17.223690
C	2.931266	-7.901508	15.996445
F	3.111147	-8.420827	14.756810
C	6.077833	-8.025120	16.683271
F	5.517317	-9.071981	16.034217
C	6.693707	-5.247407	18.220197
F	7.040543	-3.941178	18.060822
C	4.177497	-3.402242	17.841837
F	2.872744	-3.021986	17.868833
C	0.807830	-5.688743	18.520308
F	2.096198	-6.087384	18.494109
C	-1.697699	-3.871509	18.901415
F	-2.894453	-3.394693	18.462314
C	-1.547465	-1.517533	16.783975
F	-2.762672	-1.693283	16.208537
C	0.366566	-2.446333	14.496770
F	0.919579	-3.213866	13.525783
N	2.999189	-3.428338	15.134582
C	3.524273	-2.475361	14.710549
N	4.173811	-1.454952	14.331287
C	4.013359	-0.590146	13.428786
N	3.986840	0.269177	12.629943
B	3.838153	1.102892	11.350971
N	3.577815	2.595982	11.888227
C	4.279405	3.745239	11.540673
C	4.230315	4.675140	12.578097
C	3.439320	4.095984	13.605176
C	3.022157	2.852923	13.138747
C	4.947715	4.018788	10.242454
F	4.672939	3.232346	9.221499
C	4.842101	6.062185	12.559129
F	4.087861	6.928092	11.838037
C	3.206361	4.717308	14.967654
F	2.431553	3.976309	15.782763
C	1.965116	1.992859	13.825025
F	2.454293	1.399508	14.947034
N	5.229527	0.968525	10.572414
C	6.415559	0.712907	11.269676
C	7.326588	0.077145	10.440860
C	6.731077	0.000510	9.151657
C	5.452421	0.537616	9.257948
C	6.743482	1.169460	12.680111
F	6.699314	0.170295	13.596342
C	4.545876	0.704905	8.041038
F	3.319405	1.204745	8.279298
C	7.429608	-0.590116	7.940967
F	8.744395	-0.235035	7.936262
C	8.680613	-0.471984	10.839561
F	8.970735	-1.606717	10.163920
N	2.621209	0.452470	10.590068
C	2.433302	-0.926508	10.450317
C	1.093240	-1.188779	10.184034
C	0.418846	0.060589	10.185084
C	1.380400	1.043324	10.363079
C	3.484616	-2.024223	10.516160
F	3.056164	-3.000948	11.371846
C	0.428323	-2.514463	9.870341
F	-0.540745	-2.341385	8.931626
C	-1.080899	0.264147	10.162951
F	-1.583913	0.410009	8.912361
C	1.111267	2.523325	10.165921
F	0.110801	2.691390	9.270740
N	2.073030	-5.589766	14.270761
C	0.856051	-5.957890	13.692378
C	1.060146	-6.320066	12.366696
C	2.459285	-6.285601	12.132341
C	3.054411	-5.799801	13.291467
C	-0.512841	-6.069362	14.353355
F	-1.264976	-4.946106	14.175319
C	0.000537	-6.556481	11.308618
F	-1.133372	-5.851398	11.557863
C	3.132170	-6.798645	10.873806
F	3.278575	-5.824721	9.937947
C	4.566508	-5.639543	13.404757
F	5.180066	-6.837675	13.574588
F	-1.189534	-7.106078	13.795814
F	-0.492661	-6.324372	15.676057
F	0.437638	-6.128441	10.095358
F	-0.345112	-7.864122	11.185274
F	4.360586	-7.323946	11.107057
F	2.403995	-7.803962	10.317389
F	5.042946	-5.082013	12.259302
F	5.014387	-4.845639	14.409064
F	5.121596	1.559572	7.148400
F	4.374065	-0.492222	7.428658
F	7.361547	-1.947437	7.963544
F	6.929254	-0.186642	6.754723
F	9.684180	0.422360	10.596683
F	8.735273	-0.789205	12.155762
F	8.002199	1.686613	12.706402
F	5.928641	2.157073	13.129176
F	3.698717	-2.579726	9.306487
F	4.708857	-1.646704	10.969147
F	0.748988	3.170464	11.307934
F	2.178256	3.186037	9.653479
F	-1.713524	-0.786086	10.744387
F	-1.437528	1.361612	10.884465
F	1.284762	-3.432104	9.372041
F	-0.151846	-3.061420	10.969563
F	4.778753	5.282033	9.839804
F	6.414703	3.879184	10.414292
F	5.007538	6.589390	13.781119
F	6.099560	6.047562	11.987359
F	4.396334	4.874316	15.611491
F	2.623863	5.940025	14.851681
F	0.903790	2.750136	14.172626
F	1.475982	1.008562	13.034186
F	3.104883	-8.904312	16.893807
F	1.630834	-7.534045	16.098996
F	7.167866	-7.666832	15.949547
F	6.520586	-8.478417	17.886782
F	6.489679	-5.455861	19.551843
F	7.770783	-5.983316	17.866661
F	4.606671	-3.403330	19.124233
F	4.862612	-2.420003	17.187828
F	0.601808	-5.235395	19.787980
F	0.048225	-6.798271	18.345939
F	-1.925316	-5.163156	19.247853
F	-1.375838	-3.189649	20.031092
F	-1.748260	-1.081678	18.056670
F	-0.933029	-0.492064	16.134693
F	-0.859406	-2.097688	14.054145
F	1.114105	-1.293147	14.541616
Zr	8.332024	5.064712	9.706109
C	8.807377	3.224623	8.496182
C	6.981048	6.989630	8.808576
C	9.829773	3.801062	11.327391
H	9.743829	3.254475	7.920101
H	8.875202	2.372590	9.190677
H	7.979758	3.061311	7.785289
C	8.317925	7.436834	8.936963
C	9.120269	6.691649	8.017101
C	8.266514	5.802897	7.305822
C	6.947957	5.968128	7.809556
H	6.066376	5.422938	7.478390
H	8.573001	5.105621	6.532413
H	10.192028	6.801377	7.868831
H	8.672213	8.199517	9.625453
C	10.667636	4.620340	10.521767
C	10.356984	5.980029	10.806817
C	9.304920	6.001546	11.768324
C	8.987912	4.648556	12.093363
H	8.224457	4.324425	12.797282
H	8.836922	6.889801	12.186611
H	10.832745	6.850295	10.364362
H	11.412730	4.267080	9.813655
H	6.135717	7.348702	9.391532
H	9.824212	2.716033	11.332671
